# Unraveling the Effect of Aromatic Groups in Mn(I)NNN Pincer Complexes on Carbon Dioxide Activation Using Density Functional Study

**DOI:** 10.3389/fchem.2021.778718

**Published:** 2021-11-19

**Authors:** Saurabh Vinod Parmar, Vidya Avasare, Sourav Pal

**Affiliations:** ^1^ Department of Chemistry, Sir Parashurambhau College, Pune, India; ^2^ Department of Chemistry, Ashoka University, Sonipat, India; ^3^ Indian Institute of Science Education and Research, Kolkata, India

**Keywords:** Mn pincer complexes, carbon dioxide, hydrogenation, NNN pincer ligands, density functional theory

## Abstract

Carbon dioxide utilization is necessary to reduce carbon footprint and also to synthesize value-added chemicals. The transition metal pincer complexes are attractive catalysts for the hydrogenation of carbon dioxide to formic acid. There is a need to understand the factors affecting the catalytic performance of these pincer complexes through a structure–activity relationship study using computational methods. It is a well-established fact that aromatic functionalities offer stability and selectivity to transition metal catalysts. However, their impact on the performance of the catalysts is lesser known in the case of metal pincer complexes. Hence, it is necessary to investigate the catalytic performance of Mn(I)NNN pincer complexes with variably activated aromatic functionalities. In this context, 15 catalysts are designed by placing different types of aromatic rings at the pincer carbons and two terminal nitrogen of Mn(I)NNN pincer complexes. A benzene moiety, placed at C2–C3 carbons of Mn(I)NNN pincer complex with identical aromatic groups at the terminal nitrogen, is found to be most efficient toward CO_2_ hydrogenation than the rest of the catalysts. On the other hand, when N,N-dimethyl aniline is placed at C2–C3 carbons of Mn(I)NNN pincer complexes, then the catalytic performance is significantly decreased. Thus, the present study unravels the impact of aromatic groups in Mn(I)NNN pincer complexes toward the catalytic hydrogenation of carbon dioxide.

## Introduction

The burning of fossil fuels continuously increases CO_2_ concentration in the atmosphere, leading to a substantial and negative impact on the world climate. About 33,890.8 million tons of CO_2_ was released into the atmosphere in 2018, and the global CO_2_ concentration in the atmosphere reached 407.65 (BP Statistical Review of World Energy, 2019; Global Monitoring Division, 2019) Nearly ∼35 GT of CO_2_ is being added to the atmosphere per year, and there is a considerable gap between the amount of CO_2_ produced and utilized. Therefore, carbon dioxide utilization becomes a necessity to save the world from global warming. CO_2_ utilization would not only help to remove CO_2_ from the atmosphere but also helps to get alternate fuels and to reduce dependence on petrochemicals and, thus, restricts CO_2_ expulsion in the atmosphere (Centi and Perathoner, 2009; Balaraman et al., 2011). Apart from forming fuels from carbon dioxide, many synthetically useful chemicals are being synthesized from carbon dioxide (Aresta, 2010). The conversion of formic acid from carbon dioxide has manyfold benefits to the environment and economy. Formic acid is considered to be potential chemical hydrogen storage material because of its stability, nontoxicity, and easy accessibility (Dörthe et al., 2016; Jörg et al., 2017; Miriam et al., 2019; Ganesh, 2014; Kassem et al., 2020). The global formic acid market is growing at a CAGR of 1.3%, and it is expected to reach US$828.1 million by 2025 due to its wide applications in agriculture, leather, textile, rubber, chemical and pharmaceuticals, and industries (https://dataintelo.com/report/formic-acid-market). Therefore, developing an energy-efficient and environmentally benign protocol to get formic acid from CO_2_ becomes significant.

**GRAPHICAL ABSTRACT:**
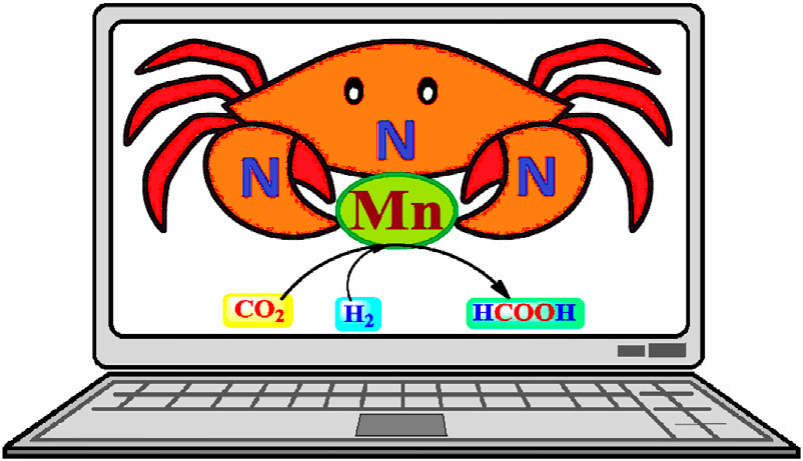
TOC for paper.

**FIGURE 1:**
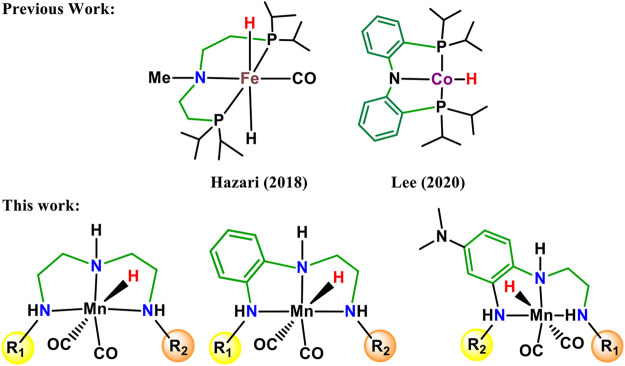
Recently developed NNN pincer ligands with varying electron withdrawing and donating moieties.

**FIGURE 2:**
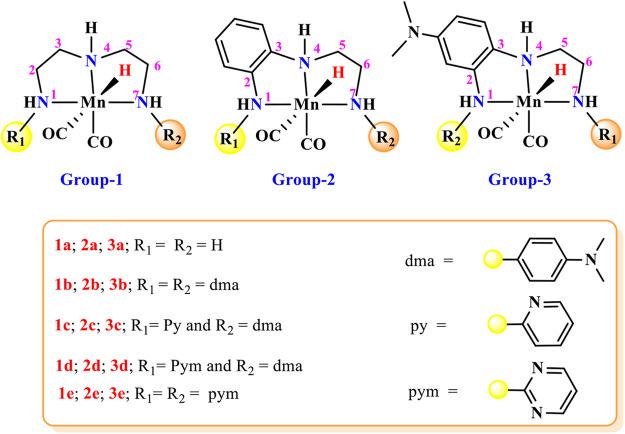
Mn(I)NNN complexes designed for the computational study of the impact of aromatic substituents on carbon dioxide hydrogenation.

In carbon dioxide activation and formation of formic acid or formate derivatives, the use of rhodium, ruthenium, and iridium pincer complexes have made remarkable progress in recent years (Wen, et al., 2021; Wang et al., 2013; Huff and Sanford, 2011, Wesselbaum et al., 2012; Kothandaraman et al., 2016; Wesselbaum et al., 2015; Campos et al., 2014). However, the high price and limited availability of these metals restrict their wide applications to an industrial scale. Therefore, the development of new catalytic technologies based on earth-abundant metals such as Fe, Mn, Ni, and Co is of considerable interest (Figure 1) (Zell and Milstein, 2015; Furstner, 2016; Langer et al., 2011; Zhang et al., 2015; Ge et al., 2016; Choi and Lee., 2020; Curley et al., 2018). The transition metal pincer complexes of these earth-abundant metals are of particular importance due to their thermal stability, cost efficiency, and flexibility for modification. The flexibility in the modification could be useful to fine-tune structural and electronic properties of the metal pincer complexes to make them more reactive as well as more selective (Peris and Crabtree, 2018). In this context, Mn pincer complexes in catalytic carbon dioxide hydrogenation have seen much progress over a much shorter time (Bertini et al., 2017; Garbe et al., 2017; Kar et al., 2017). The PNP, PCP PNN pincer complexes have been commonly used to develop transition metal pincer complexes (Kumar et al., 2019; Bernskoetter and Hazari, 2017; Irina et al., 2016; Bertini et al., 2016; Jan et al., 2020). It is necessary to understand the donor–acceptor strength of the ligand during the rational ligand designing for new catalyst development. In all the cases, the metal–ligand interaction will depend highly on the choice of the transition metal, oxidation state, coordinating sites of the ligands, and the substituents on ligands. Steric bulk is also an extremely important aspect not only for enhancing the stability of the complexes but also for providing stereoselectivity (Garbe et al., 2019; Wen et al., 2021; Jessica et al., 2017). The NNN pincer ligands are of particular importance due to their accessibility, scalability, stability, and affordability. In the recent review, Crabtree mentioned that there is a necessity to explore transition metal NNN pincer complexes and to understand their catalytic performance (Peris and Crabtree, 2018). Herein, 15 Mn(I)NNN pincer complexes are designed to understand steric and electronic factor ligands on the catalytic efficacy toward carbon dioxide hydrogenation (Figure 2). However, it would be difficult to understand the efficacy of these 15 Mn(I)NNN complexes and also to throw light on the impact of aromatic and heterocyclic rings present in the NNN pincer ligands by using experimental methods. Therefore, the use of computational methods to assess a large number of complexes by evaluating the mechanistic pathway and energetics of the reaction is highly desirable.

## Computational Details

The Gaussian-16 program package was used to perform the computational calculations (Frisch et al., 2016). The meta-GGA hybrid M06 functional with dispersion corrections without the imposition of any symmetry constraints were used to optimize reactants and intermediates (Zhao and Truhlar, 2008a). M06 is the accurate, economical functional for transition metals than B3LYP functional (Hehre et al., 1972). The SDD basis set was used for manganese, and Pople’s 6-31G(d,p) basis set was used for all the main group elements (Hariharan and Pople, 1973; Hay and Wadt, 1985). All complexes were treated as neutral species to compute optimized energies. The ground states of intermediates and transition states were confirmed as singlets through the comparison with optimized high-spin analogs. All transition states exhibited a single imaginary frequency, corresponding to the eigenvector along the reaction path. Frequency analysis of all the stationary points was performed at the same level of theory to confirm stationary points as minima or first-order saddle points along with the reaction coordinate (Liu et al., 2012; Jain et al., 2018; Zhao and Truhlar, 2008b). Intrinsic reaction coordinates (IRCs) were carried out on the transition states to endorse that such structures were indeed connecting the two minima, reactant, and product (Fukui, 1970; Fukui, 1981). All the calculations presented in this work were performed in the presence of water (dielectric constant = 78.39) using the integral equation formalism variant-like solvation model based on density (SMD) (Aleksandr et al., 2009; Aleksandr et al., 2009).

NBO analysis was carried out using the NBO 3.1 suite as implemented in Gaussian-16 (Foster and Weinhold, 1980; Reed et al., 1985; Reed and Weinhold, 1985; Glendening et al., 1987; Carpenter et al., 1988; Reed et al., 1988). The second-order perturbative estimation of donor–acceptor stabilization energy (E_s_) was calculated using the following equation,

$$E_{s}=\Delta E_{ij}=q_{i}\frac{F_{ij}^{2}}{\Delta \varepsilon_{ji}}$$ (1)

where *q*_*i*_ is the donor orbital occupancy number, and *F*_*ij*_ is the off-diagonal element of the Fock matrix in the NBO basis. Δ*ε*_*ji*_ = *ε*_*j*_ **− *ε*_*i*_ is the orbital energy difference between the acceptor (*j*) and donor (*i*) NBO.

The solvent-corrected relative Gibbs free energies (ΔG) for the transition states and intermediate as well as overall activation Gibbs free energies (ΔG^⧧^) for the catalytic cycle was calculated at 1 atm pressure and 298.15 K temperature. Visualization of all optimized structures was performed using the CYLview software (CYLview, 2020), and imaging of all NBO structures and orbitals was performed using the Chemcraft Visualization software (Chemcraft, 2021).

## Catalyst Designing

**FIGURE 3:**
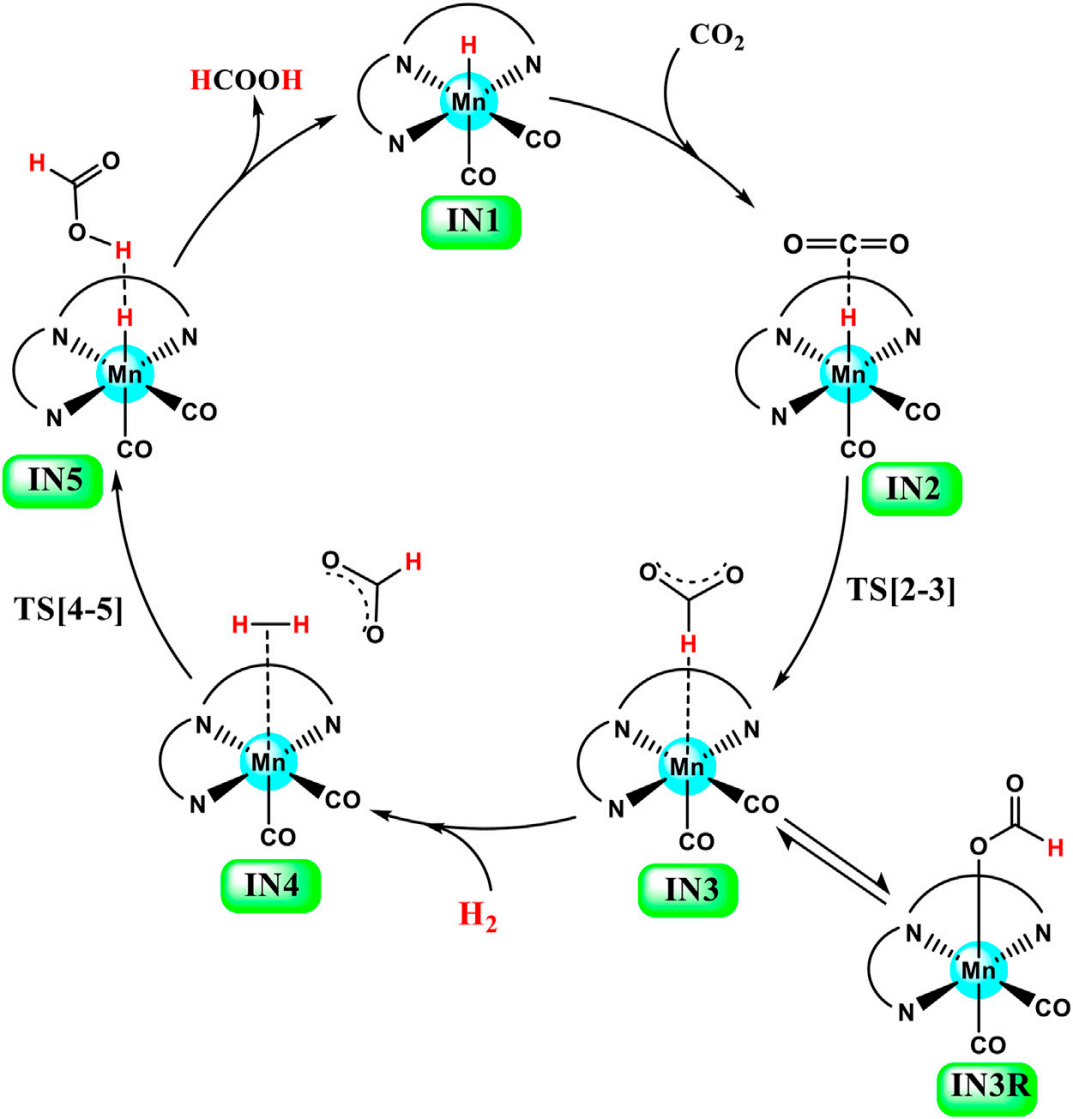
General catalytic cycle of Mn(I)NNN-catalyzed hydrogenation of carbon dioxide to formic acid through the NC pathway for **Groups 1**, **2**, and **3** catalysts.

A large number of pincer catalysts have been developed for various catalytic applications. (Bernskoetter and Hazari, 2017; Irina et al., 2016; Garbe et al., 2019; Kumar et al., 2019; Kar et al., 2020; Wen et al., 2021). However, PNP and PCP pincer complexes have been studied mostly than NNN pincer complexes (Bernskoetter and Hazari, 2017; Bertini et al., 2016; Kar et al., 2019; Tang et al., 2019). Therefore, it is necessary to explore the efficacy of Mn(I)NNN pincer complexes toward the activation of carbon dioxide. In many instances, aromatic systems are commonly used during the designing of metal pincer complexes with limited combinations (Figure 1) (Choi and Lee., 2020; Jessica et al., 2015; Talukdar et al., 2019). The aromatic systems are known to provide better thermal stability to the catalyst complex by offering steric bulk and hydrophobic groups to minimize the leaching of the metals. However, it is very important to understand the impact of the position and the nature of the aromatic rings present in the pincer complexes on their catalytic performances. It would be impossible to study all these parameters using any experimental study, and hence, the DFT (density functional theory) would play an important role to throw light on the effect of aromatic rings on the efficacy of the catalysts. Herein, 15 catalysts were designed by changing the position and reactivity of the aromatic ring attached to pincer ligands. These 15 catalysts were further studied to understand the impact of the position and nature of the aromatic rings on carbon dioxide hydrogenation (Figure 2). The computationally designed catalysts are classified into three groups: 1) **Group 1**: No aromatic ring is placed on pincer ring carbons. 2) **Group 2**: The benzene ring is inserted at the C2—C3 carbons of the pincer ring. 3) **Group 3**: The N,N-dimethyl aniline ring is inserted at the C2—C3 carbons of the pincer ring in such a way that the N,N-dimethyl group is para to the C3 nitrogen. All three groups are further subdivided into **a**, **b**, **c**, **d**, and **e** classes based on the substituents attached to the terminal nitrogens of the pincer ligands. The hydrogens attached to both the terminal nitrogens are replaced with differently activated aromatic rings in all the three groups like N,N-dimethyl aniline, pyridine, and pyrimidine (Figure 2). Catalysts **1a, 2a,** and **3a** contain all unsubstituted pincer nitrogen. The hydrogens of both the terminal nitrogen are replaced by an electron-donating group, N,N-dimethyl aniline, in **1b, 2b,** and **3b** catalysts, whereas catalysts **1c**, **2c**, and **3c** contain pyridine and N,N-dimethyl aniline groups attached to both the terminal nitrogen of the Mn(I)NNN pincer complex. Catalysts **1d**, **2d,** and **3d** are designed to have pyrimidine and N,N-dimethyl aniline groups attached to each terminal nitrogen of the pincer complexes. The two strong electrons withdrawing the pyrimidine groups are attached to the Mn(I)NNN pincer ring nitrogen in catalysts **1e**, **2e**, and **3e** (Figure 2). All these 15 Mn(I)NNN pincer complexes are further used to explore their catalytic performance toward carbon dioxide hydrogenation (Figure 3).

## Result and Discussion

### Hydrogenation of Carbon Dioxide to Formic Acid Catalyzed by Group 1 Catalysts, 1a–1e

**FIGURE 4:**
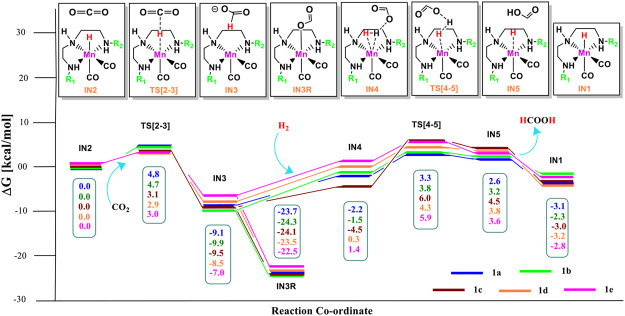
Solvent-corrected relative Gibbs free energy profile for Mn(I)NNN-catalyzed formation of formic acid through the NC pathway for catalysts **1a–1e**. Calculations were carried out at the M06/6-31G(d,p)-SDD(Mn), SMD(H_2_O) level of theory.

Catalysts **1a–1e** are designed to explore the impact of donating and withdrawing groups on the catalytic performance in the absence of any aromatic rings on the pincer ring carbons toward CO_2_ hydrogenation (Figure 2). The intermediate **IN2** is obtained after the addition of CO_2_ to the active catalyst complex **IN1** of the catalysts **1a–1e**. The relative Gibbs free energies of the transition state and the intermediate states are calculated by considering the relative Gibbs free energy of the **IN2** as 0.0 kcal/mol (Figure 4). The **IN2** is further converted into the **IN3** by transferring the hydride from the Mn center to the carbon dioxide carbon through the transition state **TS[2-3]**.

**FIGURE 5:**
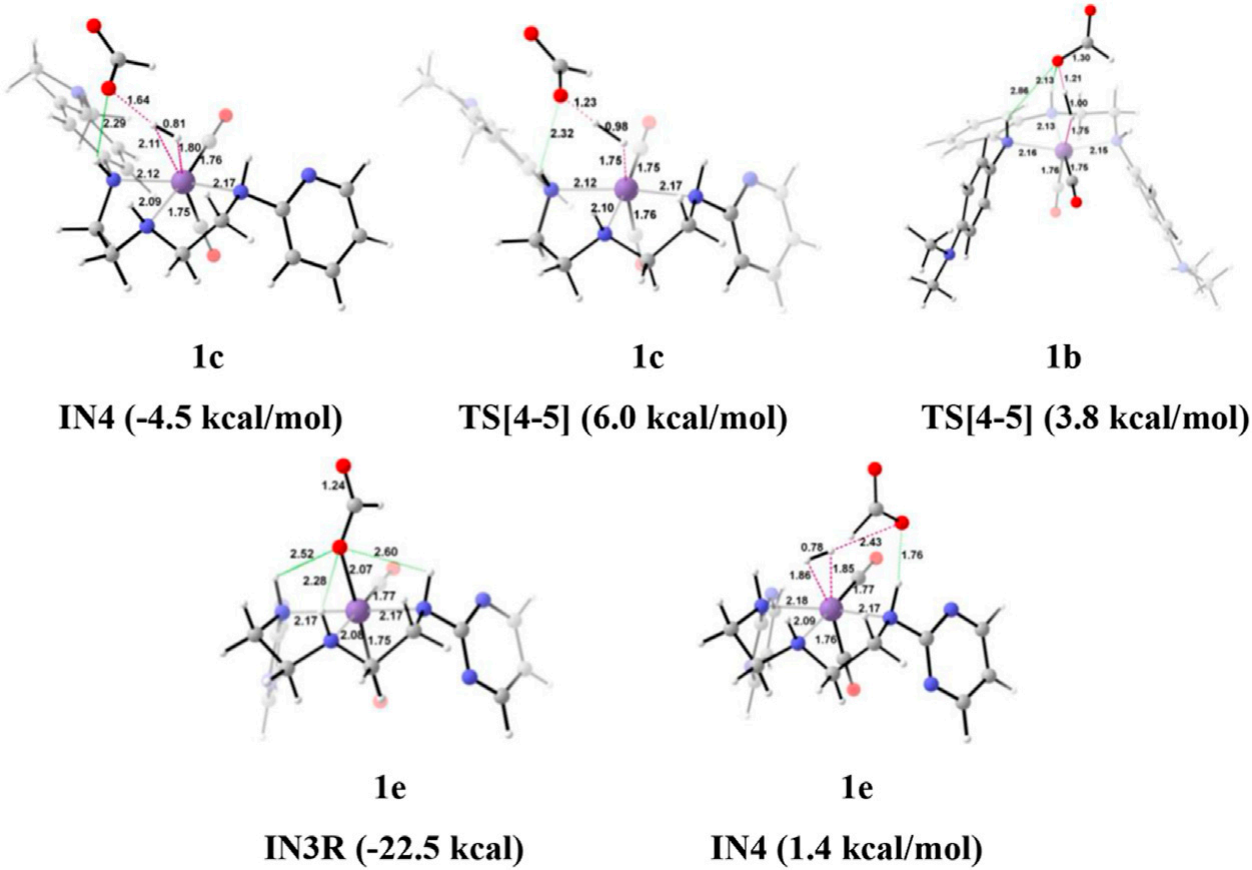
The optimized geometries of the intermediates in the formation of formic acid from carbon dioxide. The bond lengths are in Å, and the relative Gibbs free energies are in parentheses (Figures are shown in Supplementary Data Sheet S1 for all the optimized geometries of all the transition states and intermediates).

The relative Gibbs free energies for the **TS[2-3]** are ∼3.0 kcal/mol for the catalyst complexes **1c**, **1d,** and **1e** and ∼4.8 kcal/mol for the catalyst complexes **1a** and **1b**. The **IN3** of all three catalysts groups are stabilized with the relative Gibbs free energy of approximately ∼−7.0 to −10.0 kcal/mol. The relative Gibbs free energy of **IN3** is minimum for **1b** (−9.9 kcal/mol) and maximum for **1e** (−7.0 kcal/mol). The **IN3** further isomerizes to the **IN3R** (−22.5 to −24.3 kcal/mol) by forming an Mn—OCHO bond. This is the most stable state TDI (Turn-over Determining Intermediate), and hence, it is also a rate-determining state of the reaction. The **IN3R** again isomerizes to **IN3** with an energy barrier of ∼15.0 kcal/mol, and then, the protonation takes place. The hydrogen molecule adds to **IN3** to provide the **IN4** comprising formic acid. The relative Gibbs free energy of the **IN4** is minimum for **1c** (−4.5 kcal/mol) and maximum for **1e** (1.4 kcal/mol) **(** Figure 5 **)**.

**FIGURE 6:**
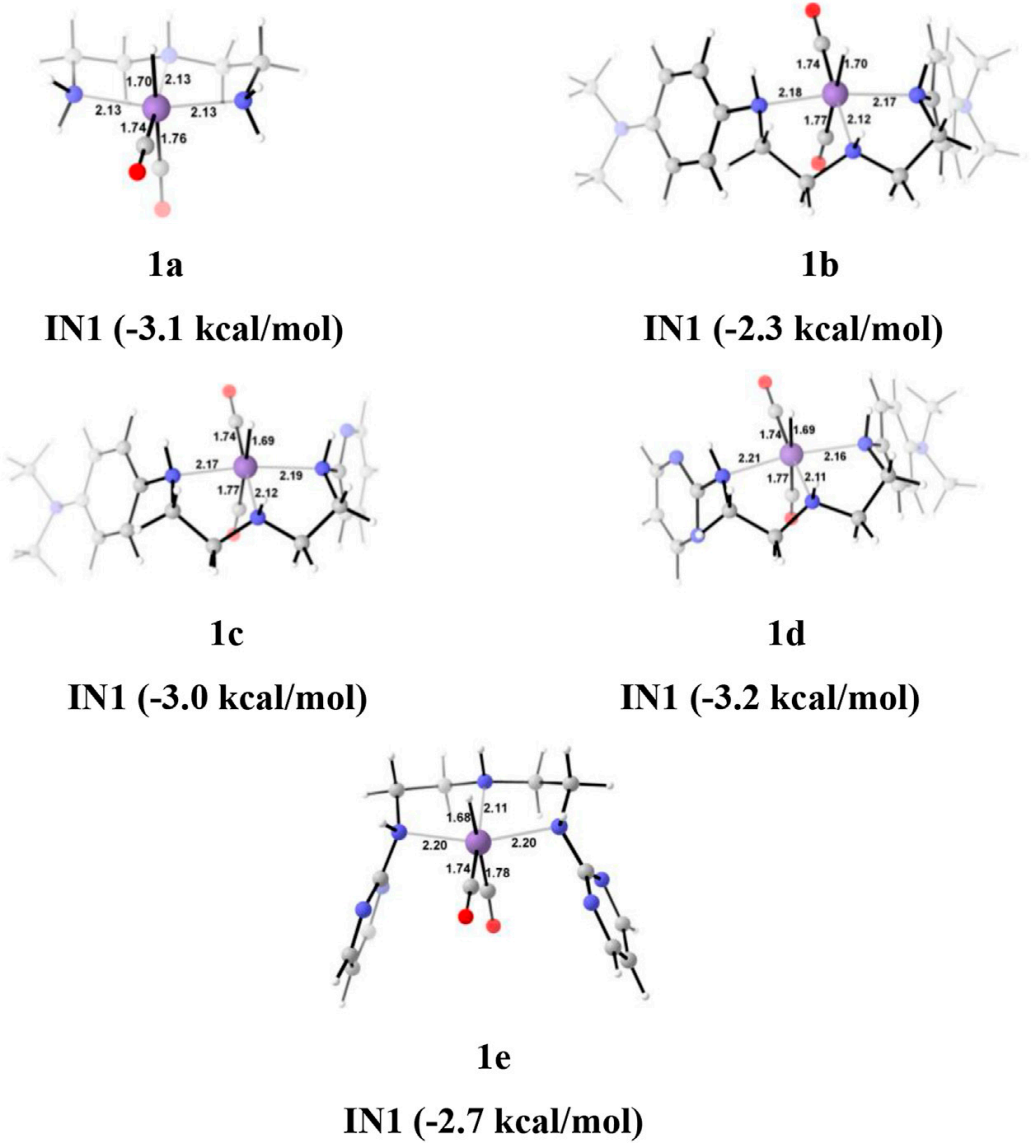
The optimized geometries of the **Group 1 IN1** catalysts. The bond lengths are in Å, and relative Gibbs free energies are in parentheses (The optimized geometries of all the transition states and intermediates are disclosed in Supplementary Data Sheet S1).

Interestingly, we found that in **Group 1**, the catalyst with a shorter Mn—N (**1a**, Mn—N = 2.13 Å) distance performs better than the catalyst with a longer Mn—N bond (**1e**, Mn—N = 2.20 Å) distance (Figure 6). However, there is no significant change in the Mn—N bond distance during the catalytic reaction. The presence of a strong H-bond (HCOO—H_2_ = 1.64 Å) in **1c** than in **1e** (HCOO—H_2_ = 2.43 Å) lowers the relative Gibbs free energy in **1c** (−4.5 kcal/mol) than **1e** (1.36 kcal/mol) in **IN4**. However, **TS[4-5]** of **1c** is found to have higher Gibbs free energy than **1b**, due to strong H_2_ polarization and strong bond-forming interactions in **1b** (1.00 Å) than in **1c** (0.98 Å).

A similar dihydrogen polarization is found to be efficient in the M—H_2_ σ-complex of **1c** (0.81 Å) than in **1e** (0.78 Å). The **IN4** is further converted into **IN5**, the regenerated catalyst and formic acid complex through the **TS[4-5]** with the relative Gibbs free energy of 3.3–6.0 kcal/mol for all the catalysts. However, the activation energy barrier for this step is the lowest for **1a** (3.3 kcal/mol) and the highest for **1c** (6.0 kcal/mol). In this entire mechanism, the dihydrogen dissociation and catalyst regeneration steps are more energy demanding by 1–3 kcal/mol than the hydride transfer transition state for the catalyst complexes **1c**, **1d**, and **1e**. On the other hand, the catalysts complexes **1a** and **1b** show facile protonation via dihydrogen polarization than the hydride transfer from the Mn center to carbon dioxide. In overall CO_2_ to formic acid formation, catalysts **1a** and **1d** have almost the same catalytic efficiency as the rest of the catalysts with ΔG^⧧^ of 23.9 and 24.6 kcal/mol (Table 1), whereas catalyst complex **1c** is most sluggish toward carbon dioxide hydrogenation. This indicates that unsubstituted carbon centers of Mn(I)NNN with unsubstituted nitrogen pincer ligands of **Group 1** show better catalytic performance than the one with electron-withdrawing groups. The donating groups and moderate withdrawing groups at terminal nitrogen make **1b** and **1c** catalysts less reactive than the Mn(I)NNN complex **1a** and **1d**. However, there is no drastic change in the reactivity when we change the N substituents in **Group 1** Mn(I)NNN pincer complexes.

**TABLE 1:** Catalytic performance of **Group 1** catalyst, **1a–1e**.

Catalyst group 1	Activation energy (ΔG^‡^) (kcal/mol)
1a	23.9
1b	25.8
1c	27.1
1d	24.6
1e	25.7

### Hydrogenation of carbon dioxide to formic acid catalyzed by Group 2 catalysts 2a–2e

**FIGURE 7:**
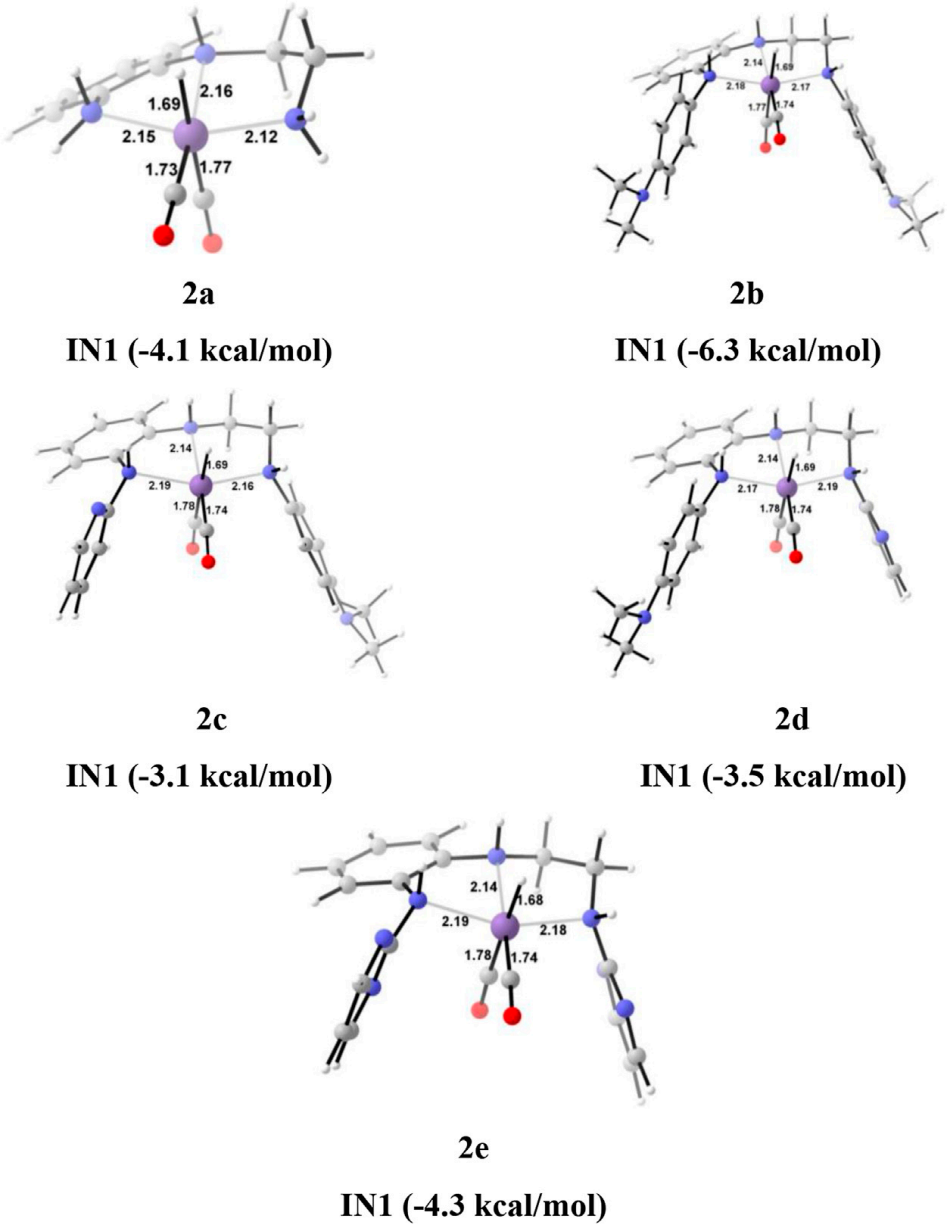
The optimized geometries of the **Group 2 IN1** catalysts. The bond lengths are in Å, and relative Gibbs free energies are in parentheses (The optimized geometries of all the transition states and intermediates are disclosed in Supplementary Data Sheet S1).

**FIGURE 8:**
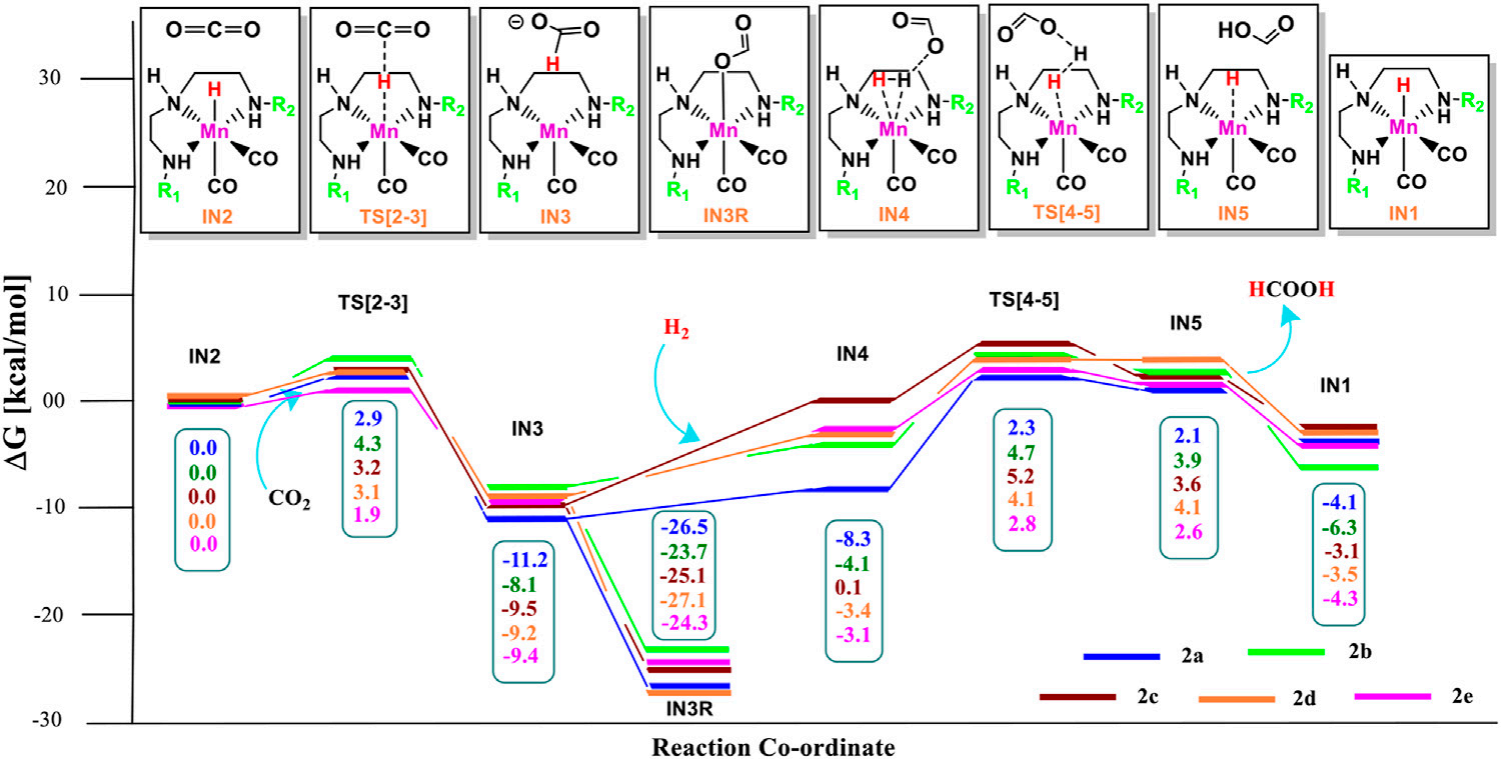
Solvent-corrected relative Gibbs free energy profile for Mn(I)NNN-catalyzed formation of formic acid through the NC pathway for catalysts **2a–2e**. Calculations were carried out at the M06/6-31G(d,p)-SDD(Mn), SMD(H_2_O) level of theory.

**FIGURE 9:**
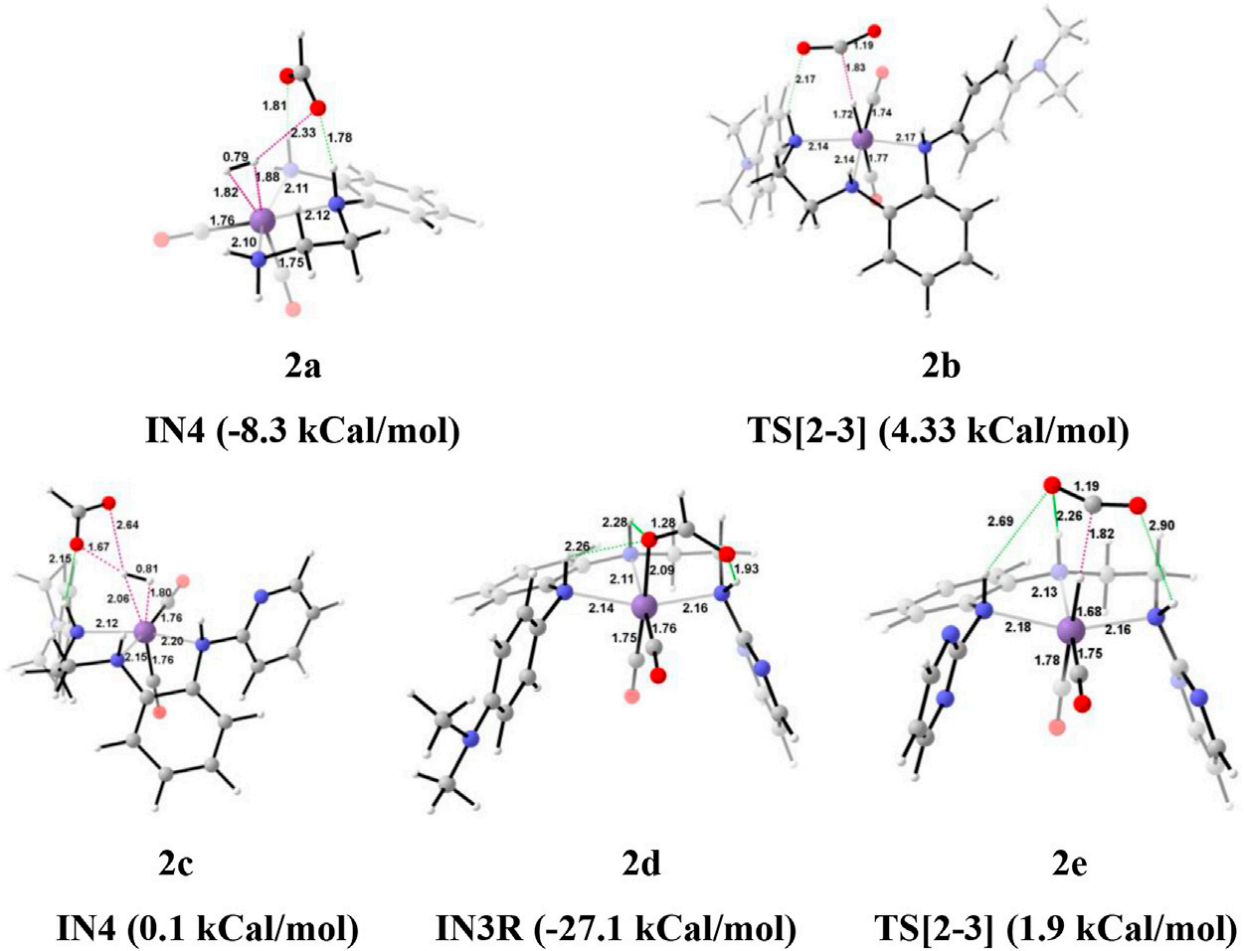
The optimized geometries of the transition state in the formation of formic acid from carbon dioxide for catalysts **2a–2e**. The bond lengths are in Å, and relative Gibbs free energies are in parentheses (Figures are shown in Supplementary Data Sheet S1 for all the optimized geometries of all the transition states and intermediates).

The unsubstituted pincer ring carbons provided exciting results; therefore, we decided to investigate the effect of aromatic substituents of the pincer carbons (C2 and C3) on the catalytic performance (Curley et al., 2018; Kumar et al., 2019; Luca et al., 2020) (Figure 2). Herein, C2 and C3 carbons of catalysts **1a-1e** are used to attach the unsubstituted aromatic ring to form the new pincer complexes **2a-2e** (Figure 7). After the addition of carbon dioxide to these pincer complexes, **IN2** is formed. The relative Gibbs free energy of the **IN2** is considered as 0.0 kcal/mol to calculate the relative Gibbs free energies of the transition state and the intermediate states (Figure 8). The intermediate **IN2** converts into the **IN3** through the transition state **TS[2-3]**. The activation energy barrier for the **TS[2-3]** is found to be maximum for catalyst **2b** (4.3 kcal/mol) and minimum for catalyst **2e** (1.9 kcal/mol). The **TS[2-3]** (1.9 kcal/mol) has the lowest relative Gibbs free energy among all the **Group 2** catalysts. This could be due to a strong H-bond [N(1) H…OCO = 2.7 Å, N(4) H…OCO = 2.3 Å, and N(7) H…OCO = 2.9 Å] among the **Group 2** catalysts (Figure 9). The **IN3** of **2a** (−11.2 kcal/mol) is the most stable, and **2b** (−8.1 kcal/mol) is less stable among all the catalysts. It has been reported in papers that **IN3** isomerizes to a more stable metal formate intermediate, **IN3R** (Boodsarin et al., 2018; Kumar et al., 2019). Herein, all the **IN3R** intermediates are stabilized by three strong H-bonds with a relative Gibbs free energy of ∼ −24.0 to −27.0 kcal/mol (All energies are added in the table in Supplementary Data Sheet S1). However, strong H-bonds [N(1) H…OCHO = 2.26 Å, N(4) H…OCHO = 2.28 Å, and N(7) H…OCHO = 1.93 Å] stabilize the **IN3R** of catalyst **2d** more effectively than the rest of the catalysts (Figure 9). The isomerization energy barrier for **IN3R** to **IN3** is ∼15 kcal/mol for all the catalysts except **2d** (18.0 kcal/mol). When **IN3R** changes to **IN3**, then the hydrogen molecule adds to the **IN3** to provide the intermediate **IN4**. In the **IN4**, there is an increase in the Gibbs free energy by 2–9 kcal/mol than **IN3**.

In the Mn—H_2_ σ-complex, the **IN4** of catalyst **2a** is most stable (−8.3 kcal/mol), whereas **2c** is the least stable (0.1 kcal/mol) (Figure 9) among all the catalysts. The presence of stronger H-bonds [N(1) H…OCHO = 1.8 Å and N(4) H…OCHO = 1.78 Å] offer better stability than all the catalysts (∼0 to −4 kcal/mol). The **IN4** of **2a** shows an Mn-H_2_ σ-complex with an Mn center, and the remaining catalysts show a distorted Mn-H_2_ σ-complex, where one Mn—H_a_ (1.88 Å) is longer than the other Mn—H_b_ (1.82 Å) bond (Structure in Supplementary Data Sheet S1). These distortions in the Mn—H bond could be due to strong dihydrogen polarization. The **IN4** converts into **IN5** through the **TS[4-5]**, where the strong dissociation of a dihydrogen bond is observed in all the catalysts. The H-bond stabilizes the **TS[4-5]** with the relative Gibbs free energy of 2.0 to 5.0 kcal/mol. The **IN5** contains the product, formic acid, and the regenerated catalyst complex. The **IN5** further expels formic acid and provides regenerated catalysts, **2a–2e**. The ΔG for the entire catalytic reaction is found to be minimum for catalysts **2b** (22.1 kcal/mol) and **2e** (22.8 kcal/mol) and maximum for catalysts **2c** (27.2 kcal/mol) and **2d** (27.7 kcal/mol) (Table 2). This indicates that in **Group 2**, catalysts show better catalytic performance when both the terminal nitrogen are attached to either electron-donating groups, N,N-dimethyl aniline, or strong electron-withdrawing groups, pyrimidine. On the other hand, the catalytic performance slows down when these two terminal nitrogens carry mixed electron-withdrawing and donation groups **2a** and **2c**. However, catalyst **2a** with an unsubstituted terminal nitrogen shows average performance toward carbon dioxide hydrogenation.

**TABLE 2:** Catalytic performance of **Group 2** catalyst, **2a–2e**.

Catalyst group 2	Activation energy (ΔG^‡^) (kcal/mol)
2a	24.7
2b	22.1
2c	27.2
2d	27.7
2e	22.8

### Hydrogenation of carbon dioxide to formic acid catalyzed by Group 3 catalysts, 3a–3e

**FIGURE 10:**
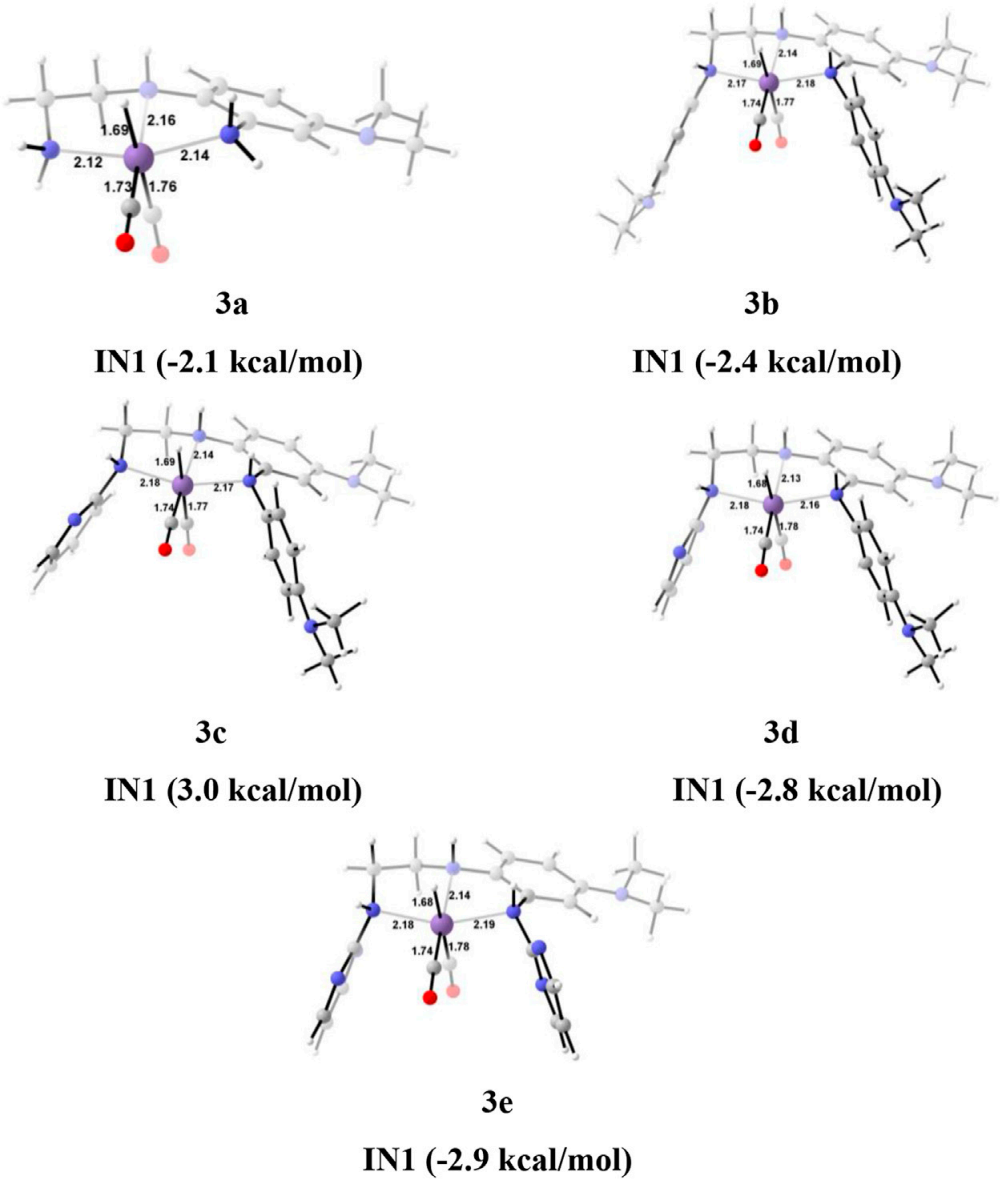
The optimized geometries of the **Group 3 IN1** catalysts. The bond lengths are in Å, and relative Gibbs free energies are in parentheses (The optimized geometries of all the transition states and intermediates are disclosed in Supplementary Data Sheet S1).

**FIGURE 11:**
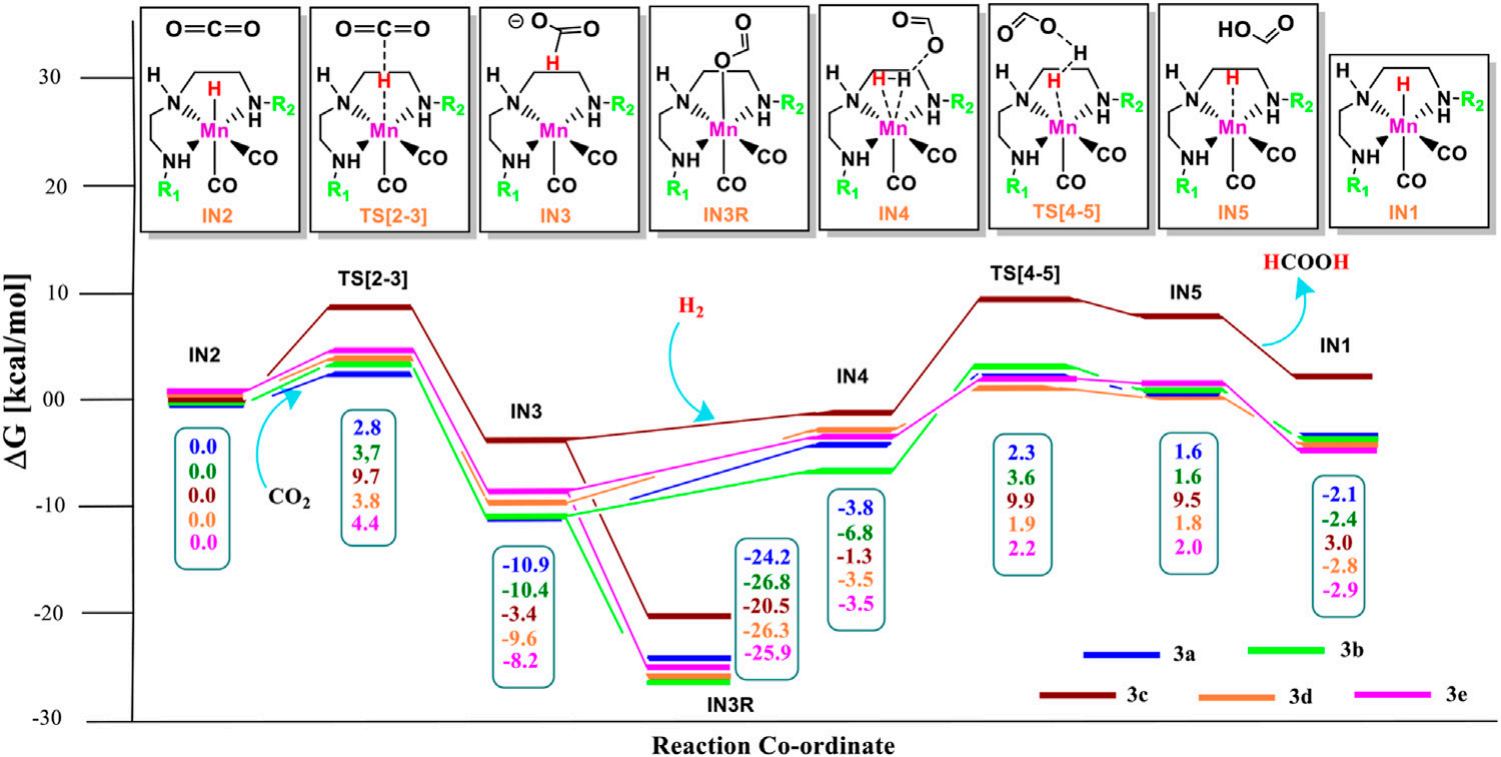
Solvent-corrected relative Gibbs free energy profile for Mn(I)NNN-catalyzed formation of formic acid through the NC pathway for catalysts **3a–3e**. Calculations were carried out at the M06/6-31G(d,p)-SDD(Mn), SMD(H_2_O) level of theory.

**FIGURE 12:**
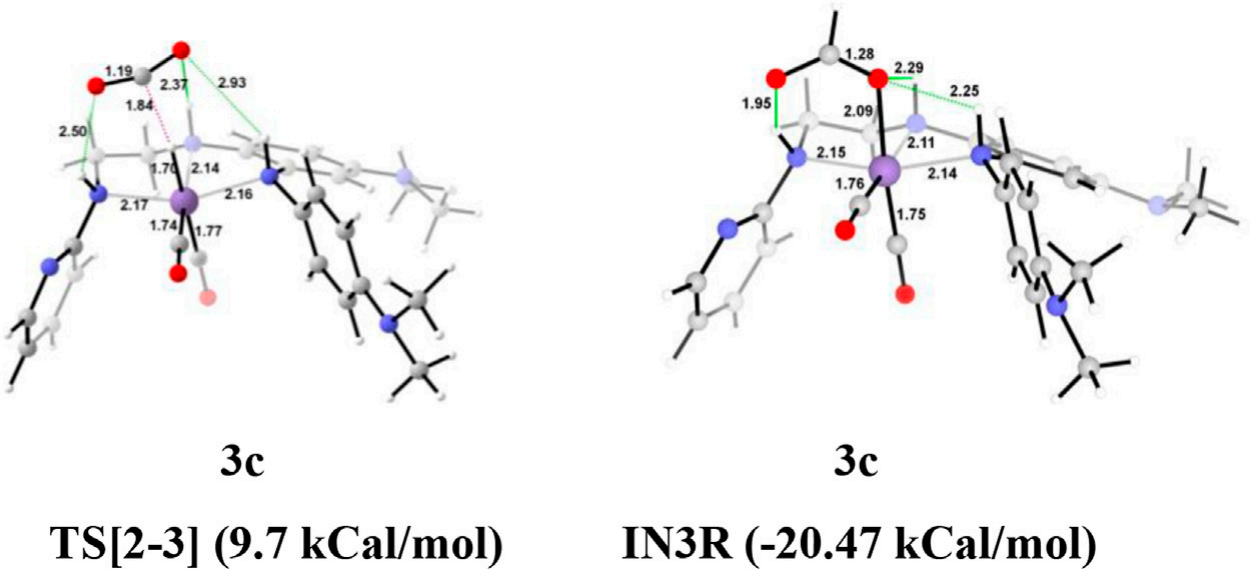
The optimized geometries of the transition states **TS[2-3]** in the formation of formic acid from carbon dioxide for catalysts **3a–3e**. The bond lengths are in Å, and relative Gibbs free energies are in parentheses (The optimized geometries of all the transition states and intermediates are disclosed in Supplementary Data Sheet S1).

The unsubstituted **Group 1**, as well as benzene, substituted **Group 2** manganese pincer complexes provided a deeper insight into their catalytic performances toward carbon dioxide hydrogenation reaction. Therefore, we also investigated the effect of the activated aromatic ring by placing N,N-dimethyl aniline at the C2—C3 of the pincer ligands of the Mn(I)NNN complex in **Group 3** (Figures 2 and 10) (Smith et al., 2018). Complexes **3a–3e** are designed to understand the effect of Mn(I)NNN complexes on carbon dioxide hydrogenation. After the addition of carbon dioxide to these pincer complexes, the **IN2** is formed. The relative Gibbs free energy of the **IN4** is considered as 0.0 kcal/mol to calculate the relative Gibbs free energies of the transition and the intermediate states (Figure 11). The **IN2** converts into the **IN3** through the transition state **TS[2-3]**. The relative Gibbs free energy of the **TS[2-3]** of all the catalysts is in the range of 3.0–4.0 kcal/mol except for catalyst **3c** (9.7 kcal/mol). The **TS[2-3]** [N(1) H…OCHO = 2.9 Å, N(4) H…OCHO = 2.4 Å, and N(7) H…OCHO = 2.5 Å] and **IN3R** [N(1) H…OCHO = 2.29 Å, N(4) H…OCHO = 2.25 Å, and N(7) H…OCHO = 1.95 Å] show moderate H-bonds in all the catalysts (Figure 12). After careful evaluation, it is observed that the **IN1** of **3c** has higher energy than the rest of the catalysts, and hence, the entire catalytic cycle, transitions states, and intermediates associated with this catalyst have higher relative Gibbs free energy than the other catalysts of **Group 3**. The formation of **IN3** is found to be exergonic by 12.0–13.0 kcal/mol in all the catalysts of **Group 3**. The **IN3** isomerizes to the resting state, **IN3R**. The relative Gibbs free energy of the resting state **IN3R** is in the range of −24.0 to −27.0 kcal/mol except for catalyst **3c** (−20.5 kcal/mol). The **IN3R** is the rate-controlling state in the entire catalytic conversion. The **IN3R** again isomerizes to **IN3** to undergo further reaction to form product **IN5**. The dihydrogen molecule adds to the **IN3** to give the intermediate **IN4**. The **IN4** forms a classical σ-complex with an Mn center. Similar to **Group 2**, a strong Mn—H_2_ σ-complex is formed in **IN4** of **3a**, while the remaining catalysts show a distorted Mn-H_2_ σ-complex, where one Mn—H_a_ (1.88 Å) is longer than the other Mn—H_b_ (1.82 Å) bond (Structure in Supplementary Data Sheet S1). A strong polarization must have led to this distortion.

The **IN4** of the **3b** (−6.8 kcal/mol) is the most stable, and **3c** (−1.3 kcal/mol) is the least stable among all the catalysts of **Group 3**. The **IN4** further converts to the **IN5** through the transition state **TS[4-5]** with a maximum relative Gibbs free energy for catalyst **3c** (9.9 kcal/mol) and minimum for catalyst **1a**, **1d**, and **1e** (∼2.0 kcal/mol). The entire CO_2_ hydrogenation is found to be thermodynamically favorable for all the catalysts except for catalyst **3c**. This indicates that electron-donating and weak-withdrawing groups at an Mn center make dihydrogen polarization and catalyst generation sluggish. On the other hand, unsubstituted terminal nitrogen and terminal nitrogen with strong withdrawing groups facilitate the dihydrogen polarization and carbon dioxide to the formic acid formation (Table 3).

**TABLE 3:** The catalytic performance of **Group 3** catalyst, **3a–3e**.

Catalyst group 3	Activation energy (ΔG^‡^) (kcal/mol)
3a	24.4
3b	28.0
3c	33.4
3d	25.4
3e	25.3

## Natural Bond Orbital

**FIGURE 13:**
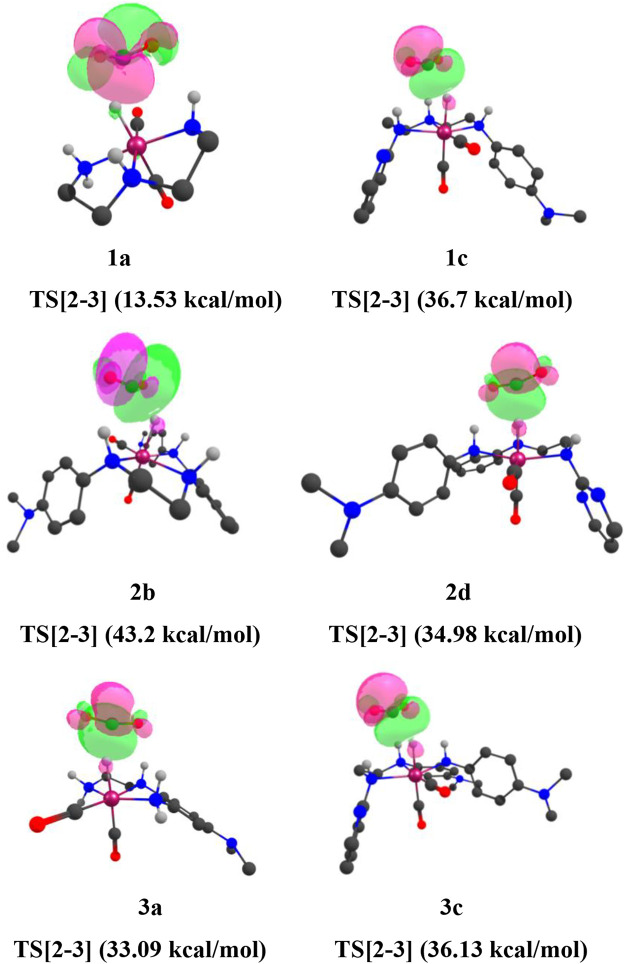
(Mn)H—CO_2_ interaction of natural bond orbitals (NBOs) of **TS[2-3]** of catalysts **1a, 1d, 1e, 2a, 2b, 2e**, and **3a** (isovalue = 0.0174). Interaction energies are indicated in parentheses. The structures are visualized using the Chemcraft software (Chemcraft, 2021) (NBO for all catalysts are given in Supplementary Data Sheet S1).

Natural bond orbital **(**NBO**)** analysis is performed to gain a mechanistic insight into the carbon dioxide hydrogenation reaction mechanism (Chemcraft, 2021). The Lewis acid–base pair present in the chemical species can be predicted from the second-order perturbative estimation of donor–acceptor stabilization energy ( $E_{s}$ ). The NBO analysis of the transition states **TS[2-3]** provides the picture of relevant orbital interactions for the bond formation based on the second-order perturbative interaction energy. At the transition state **TS[2-3]**, prominent bond-forming interactions are observed for the C—H(a) bond. (Figure 13).

**FIGURE 14:**
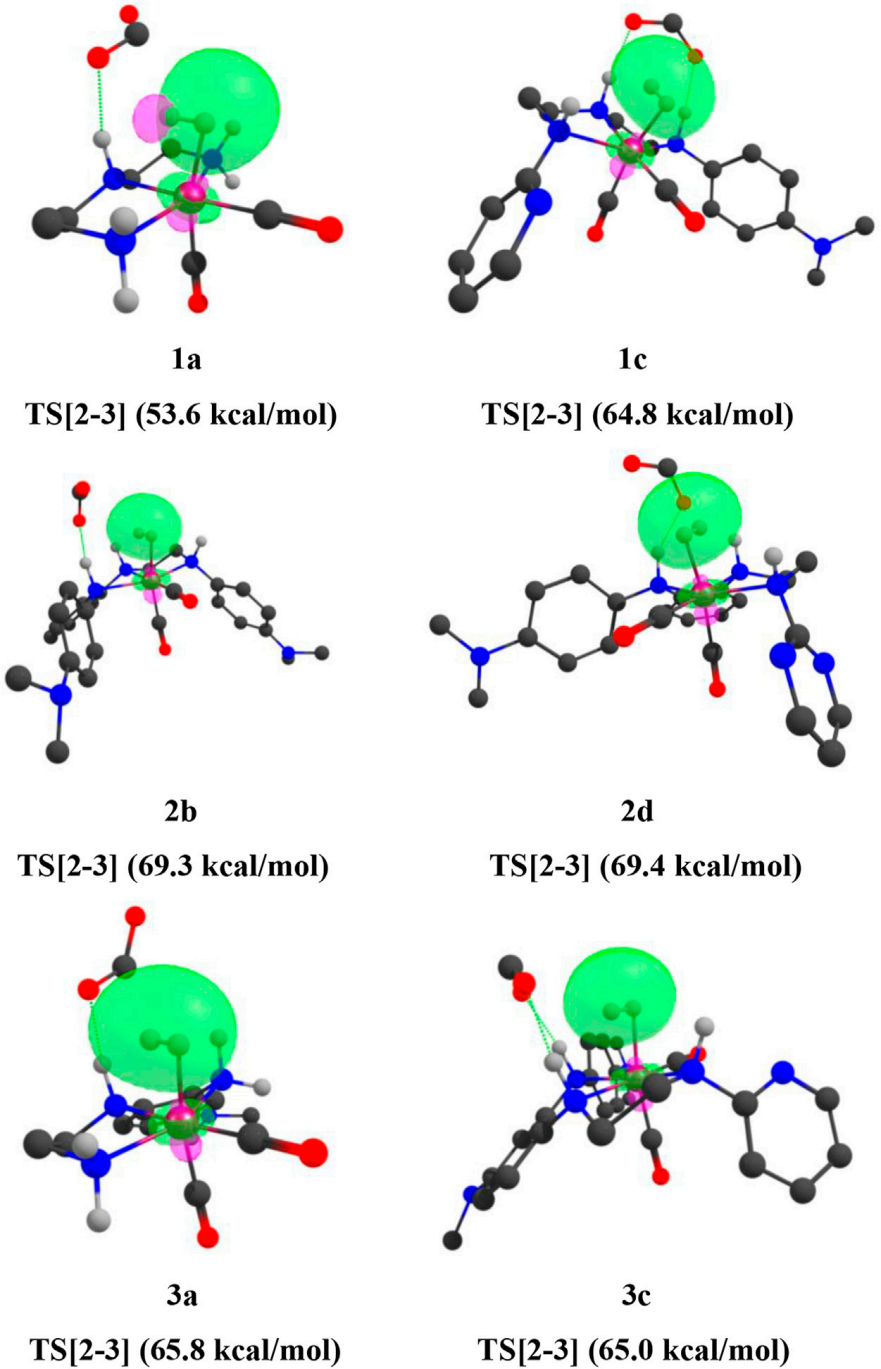
Mn—H_2_ interaction NBOs of **IN4** of catalysts **1a, 1d, 1e, 2a, 2b, 2e**, and **3a** (isovalue = 0.03). Interaction energies are indicated in parentheses. The structures are visualized using the Chemcraft software (Chemcraft, 2021) (NBO for all catalysts are given in Supplementary Data Sheet S1).

Here, the **TS[2-3]** of **1e** has stronger bond-forming interaction energy (45.01 kcal/mol) and lower relative Gibbs free energy(3.0 kcal/mol), whereas the **TS[2-3]** of **1a** shows weaker bond-forming interaction energy of (13.53 kcal/mol) and higher relative Gibbs free energy (4.8 kcal/mol). A similar trend is observed when NBO analysis is performed for the **IN4** of all the catalysts (Figure 14).

## Conclusion

The structure–activity relationship study of computationally modeled Mn(I)NNN pincer complexes emphasize that the position and nature of the aromatic rings attached to the pincer ligands affect the catalytic performance to a considerable amount for carbon dioxide hydrogenation reaction. The **Group 2** Mn(I)NNN pincer complexes with benzene substituent at C2—C3 and identical substituents at both the terminal nitrogen are superior to all the catalysts from the three groups, whereas **Group 1** catalysts without any aromatic substituents at C2—C3 show moderate catalytic performance, and **Group 3** catalysts with N,N-dimethyl aniline at C2—C3 are sluggish toward carbon dioxide hydrogenation.

## Data Availability

The original contributions presented in the study are included in the article/Supplementary Material. Further inquiries can be directed to the corresponding authors.
