# Supplementary material for: Unraveling the Effect of Aromatic Groups in Mn(I)NNN Pincer Complexes on Carbon Dioxide Activation Using Density Functional Study
Source: Front Chem. 2021 Nov 19;9:778718. doi: 10.3389/fchem.2021.778718 (PMC8639700; doi:10.3389/fchem.2021.778718)
Supplement: Supplementary file 1 [file DataSheet1.PDF]

Supplementary Material for

Unraveling the effect of aromatic rings of Mn(I)NNN pincer complexes on carbon dioxide activation using Density Functional Study

Saurabh Vinod Parmar,[a] Vidya Avasare,[ac]* and Sourav Pal[bc]*

[a]Department of Chemistry, Sir Parashurambhau College

Tilak Road, Pune, Maharashtra 411030 India

[b] Indian Institute of Science Education and Research

Mohanpur, Kolkota, West Bengal, 741246 India

[c]Ashoka University Sonipat Haryana, 131029 India

Table of Contents

Sr.no.	List of contents	Page
1.	Title, authors' name, address and tables	S1
2.	CYL-view structures of Intermediates and Transition States	S2 - S22
3.	NBO structures for TS[2-3] and IN4	S23 – S28
4.	Coordinates of all Intermediates and Transition States	S29 – S117
5.	Table of Electronic Energies of the transition states and intermediates in Hartree	S118 - S119

CYL-view structures of Intermediates and Transition States-

GROUP-1

IN1

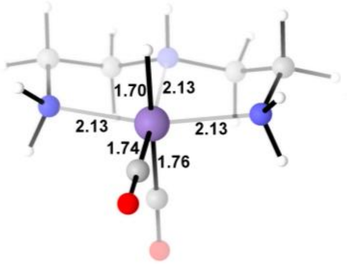

1a-IN1

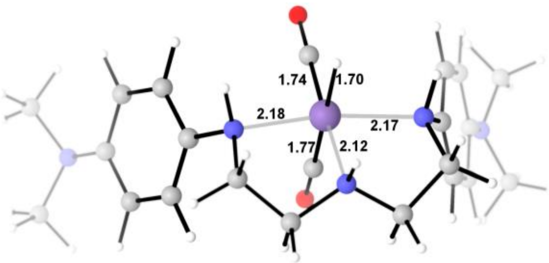

1b-IN1

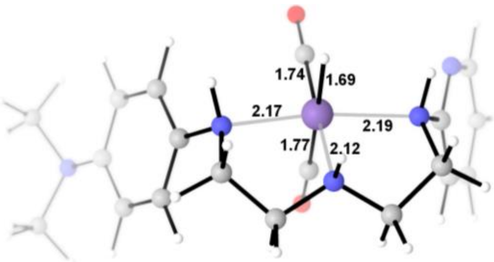

1c-IN1

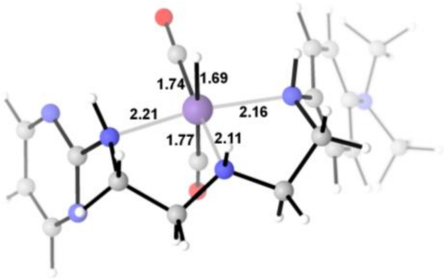

1d-IN1

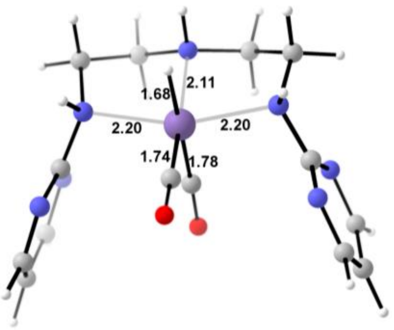

1e-IN1

IN2

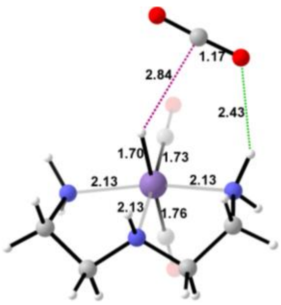

1a-IN2

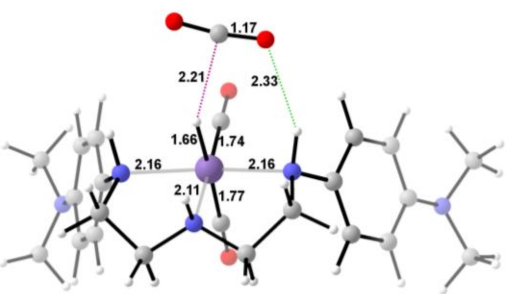

1b-IN2

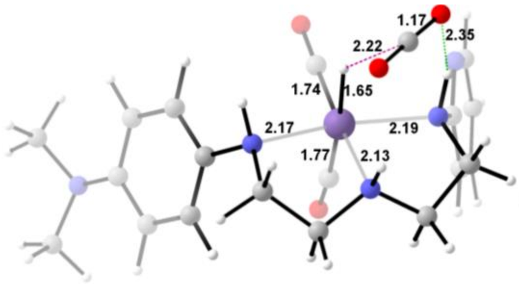

1c-IN2

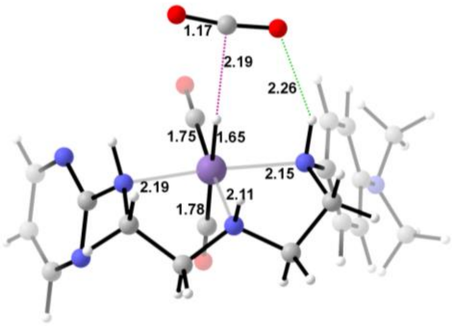

1d-IN2

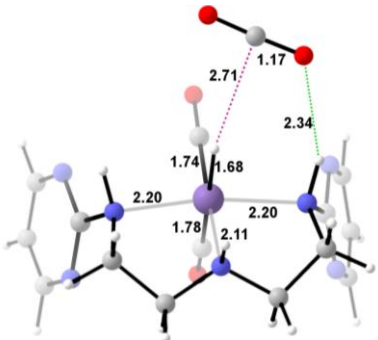

1e-IN2

TS[2-3]

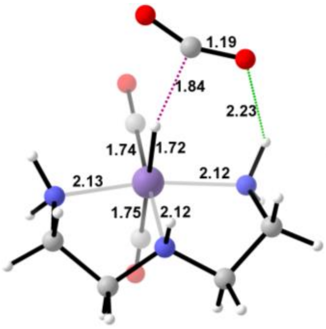

1a-TS[2-3]

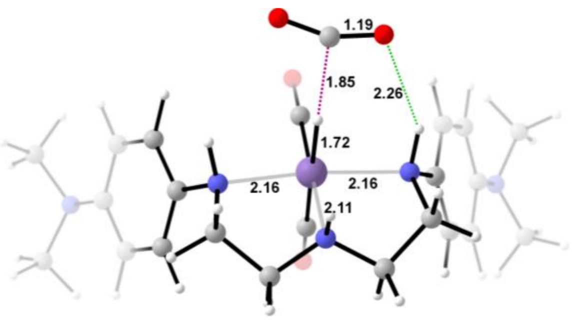

1b-TS[2-3]

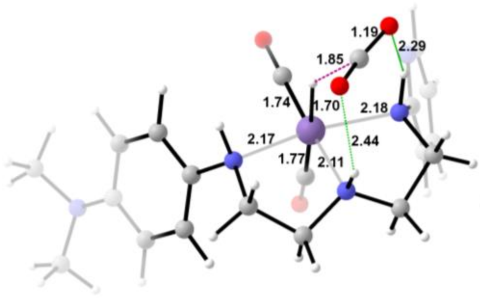

1c-TS[2-3]

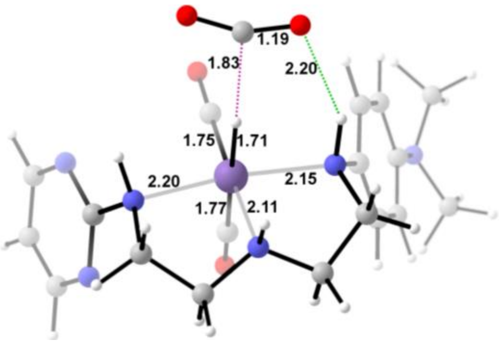

1d-TS[2-3]

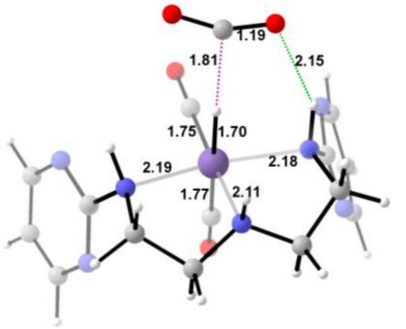

1e-TS[2-3]

IN3

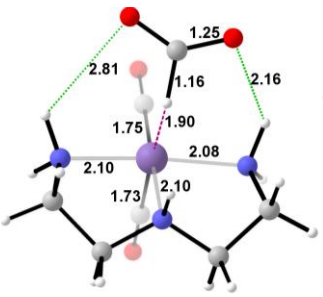

1a-IN3

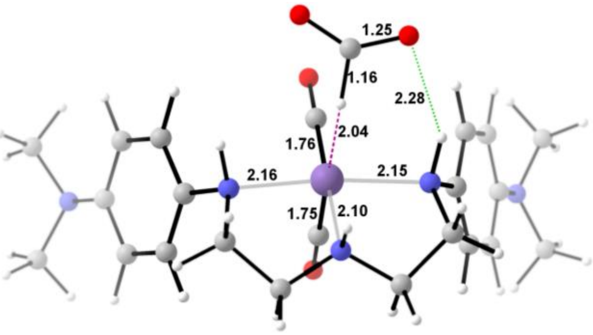

1b-IN3

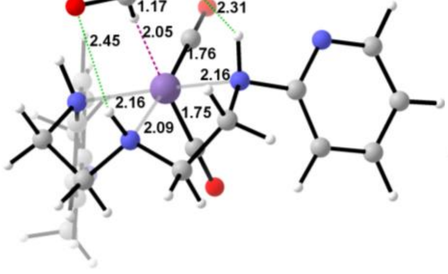

1c-IN3

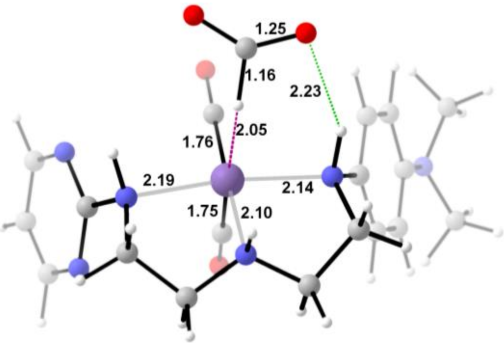

1d-IN3

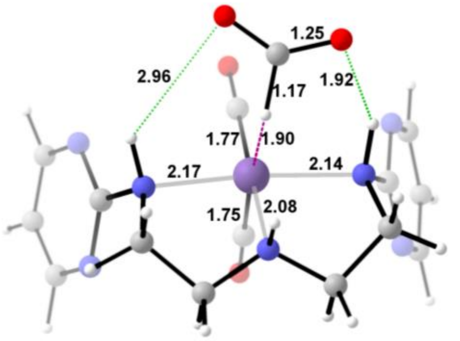

1e-IN3

IN3R

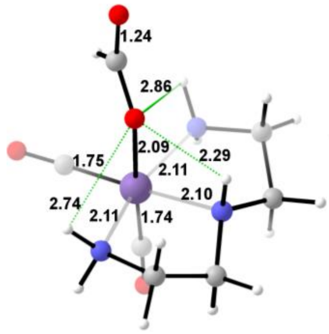

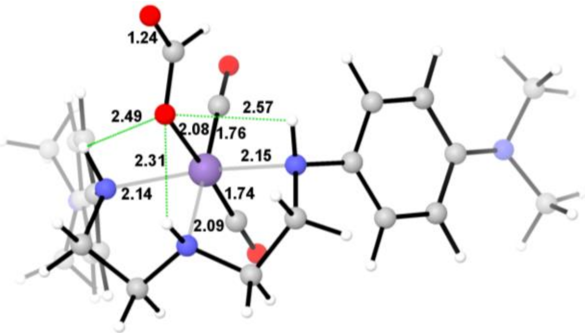

1a-IN3R

1b-IN3R

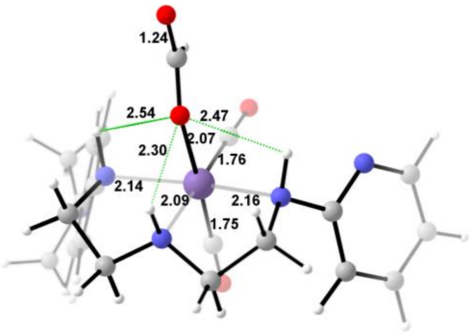

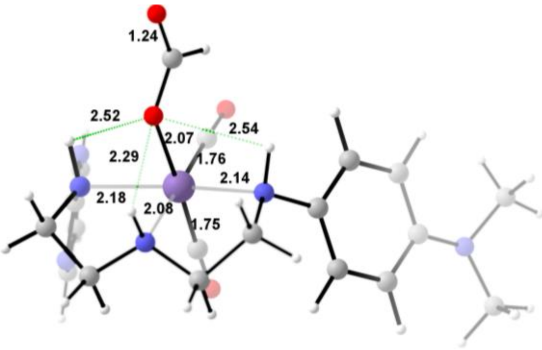

1c-IN3R

1d-IN3R

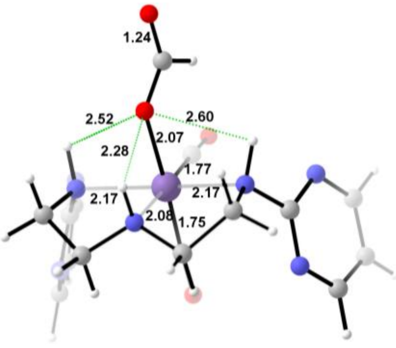

1e-IN3R

IN4

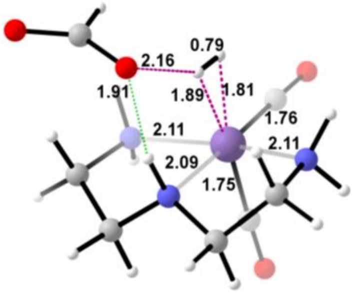

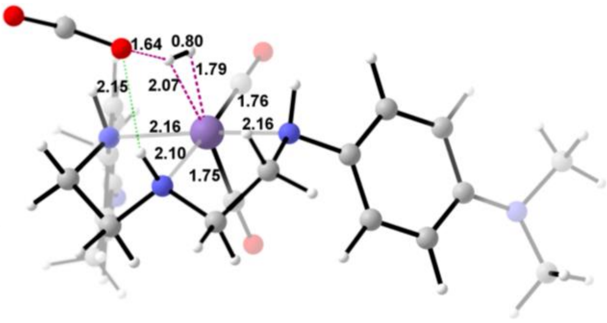

1a-IN4 1b-IN4

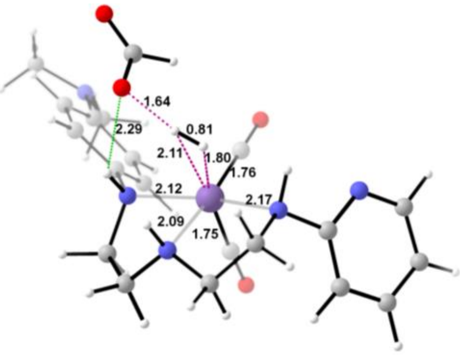

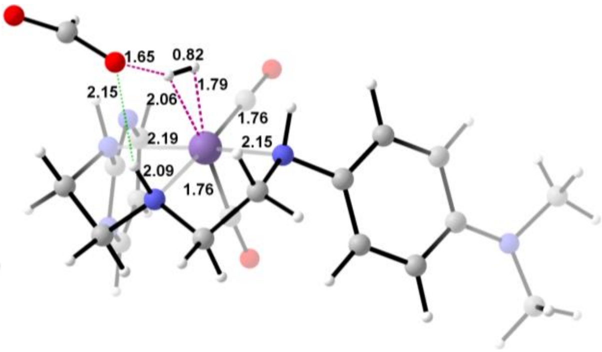

1c-IN4 1d-IN4

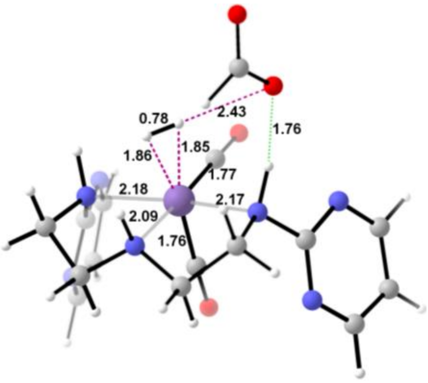

1e-IN4

TS[4-5]

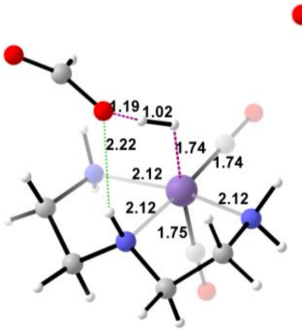

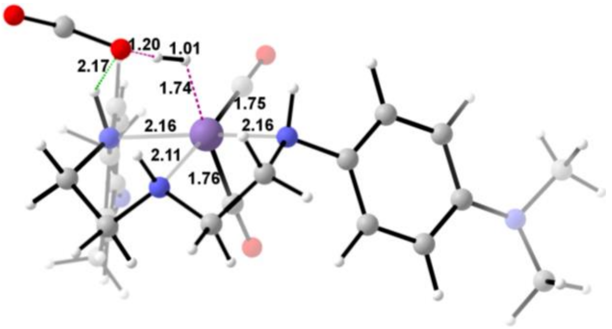

1a-TS[4-5]

1b-TS[4-5]

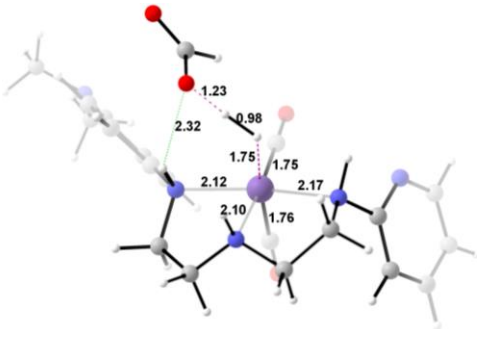

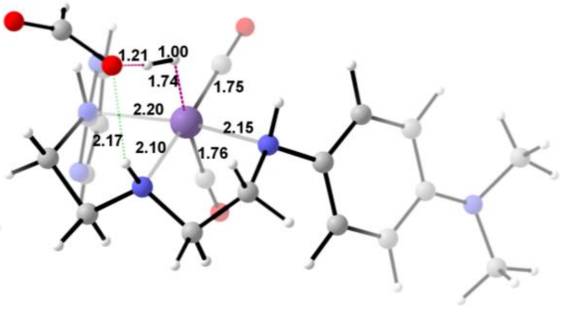

1c-TS[4-5]

1d-TS[4-5]

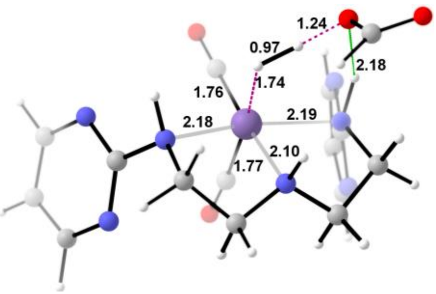

1e-TS[4-5]

IN5

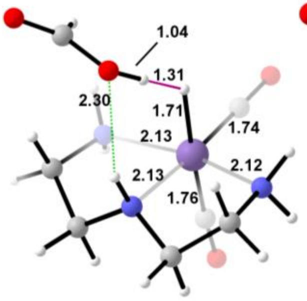

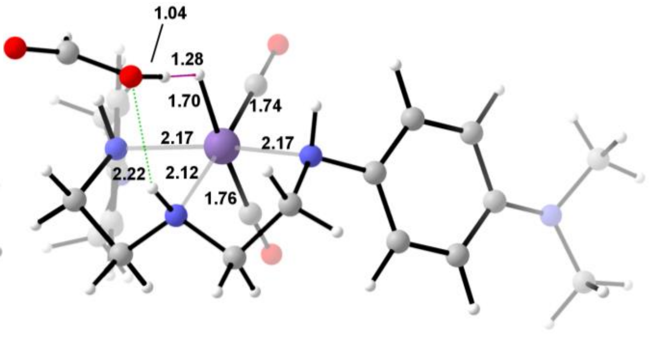

1a-IN5

1b-IN5

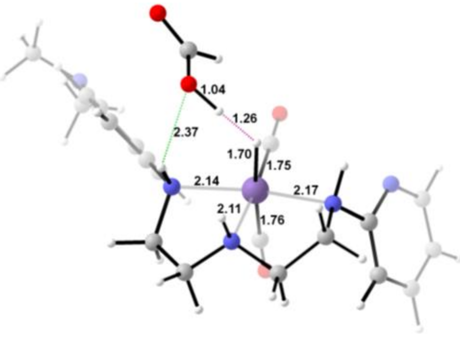

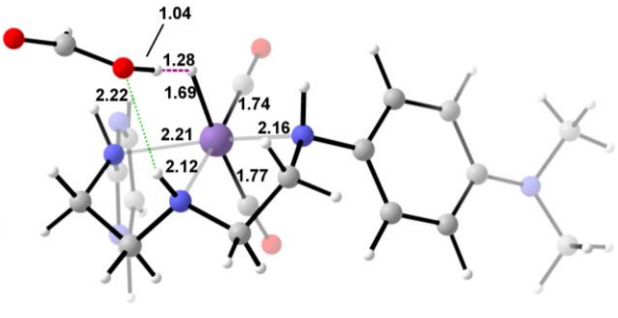

1c-IN5

1d-IN5

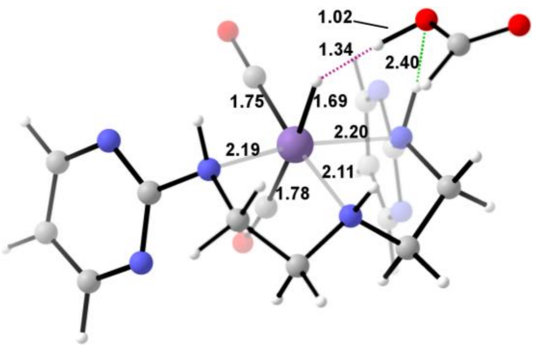

1e-IN5

GROUP-2

IN1

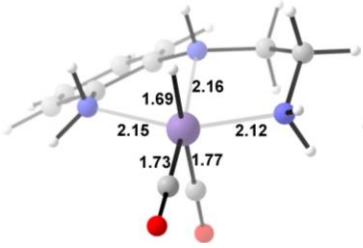

2a-IN1

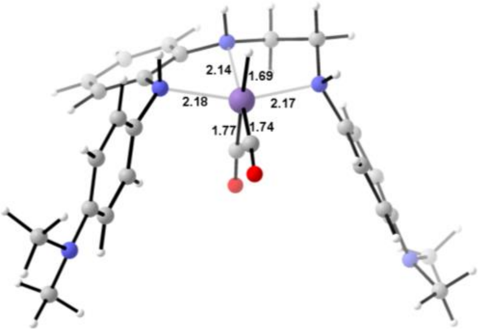

2b-IN1

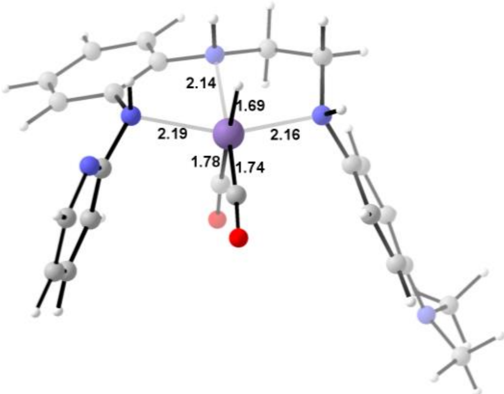

2c-IN1

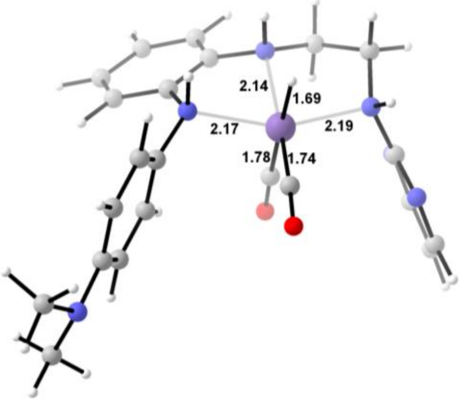

2d-IN1

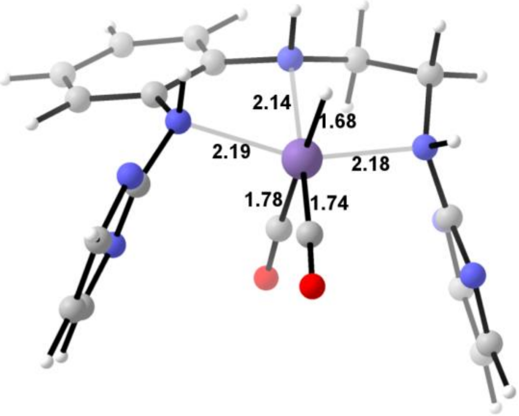

2e-IN1

IN2

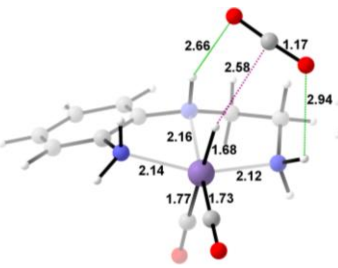

2a-IN2

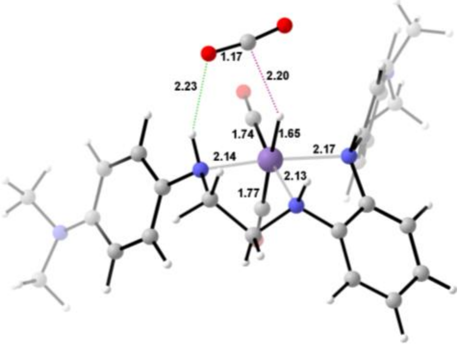

2b-IN2

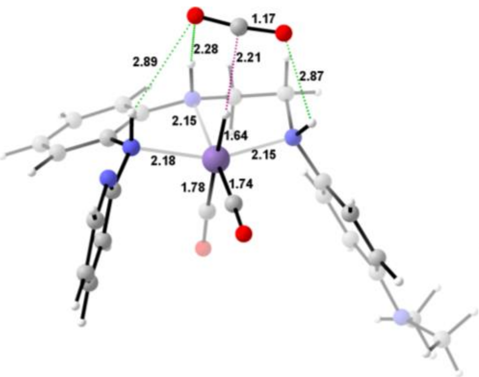

2c-IN2

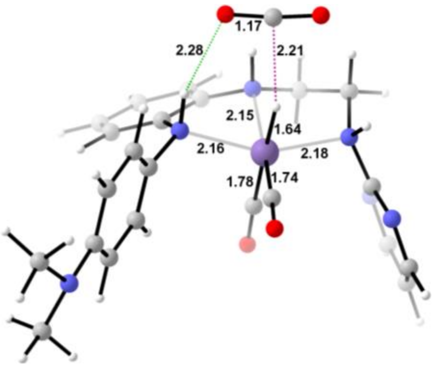

2d-IN2

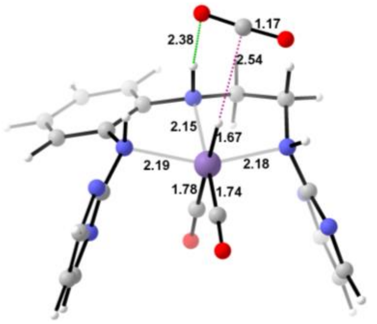

2e-IN5

TS[2-3]

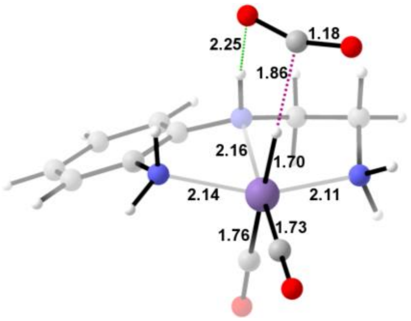

2a-TS[2-3]

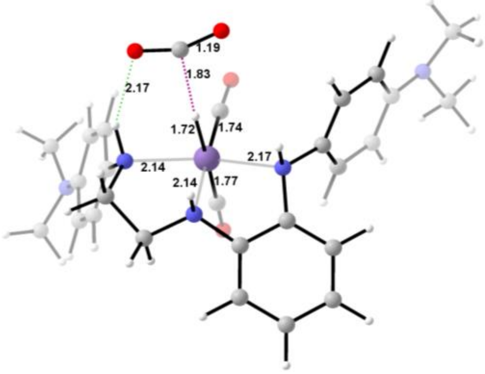

2b-TS[2-3]

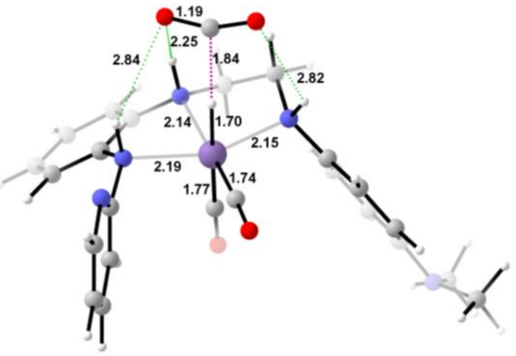

2c-TS[2-3]

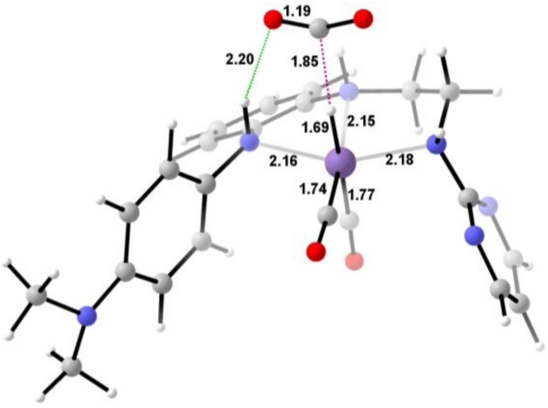

2d-TS[2-3]

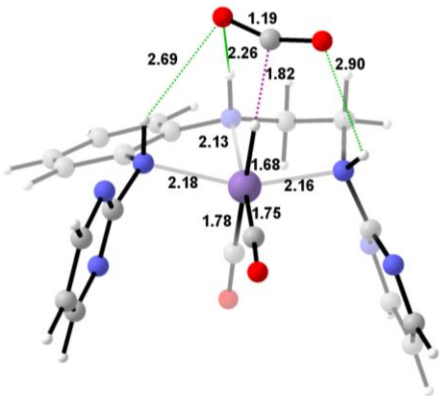

2e-TS[2-3]

IN3

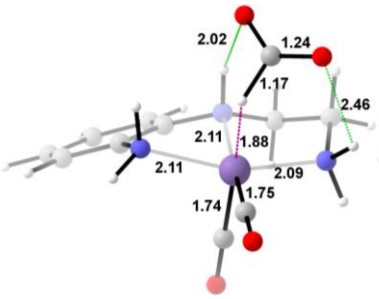

2a-IN3

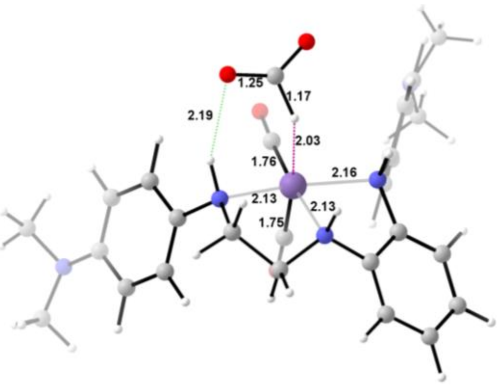

2b-IN3

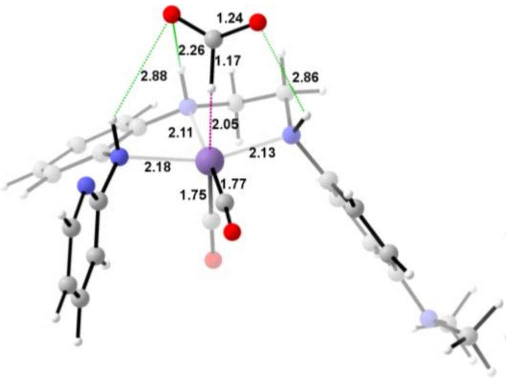

2c-IN3

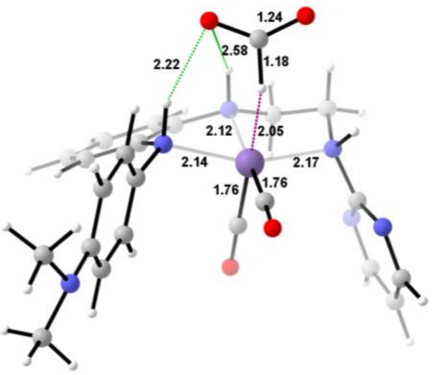

2d-TS[2-3]

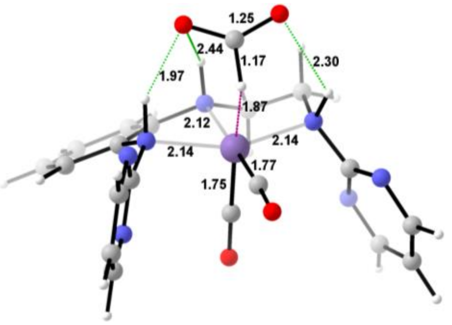

2e-TS[2-3]

IN3R

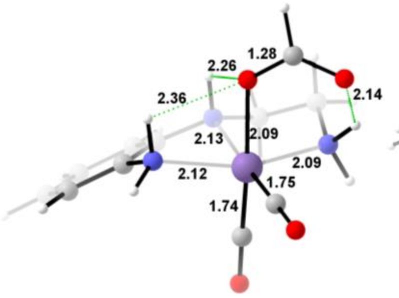

2a-IN3R

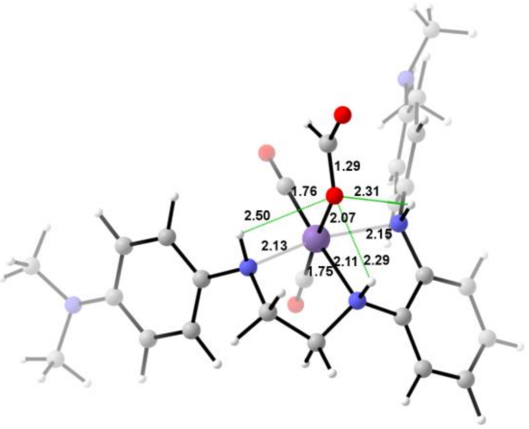

2b-IN3R

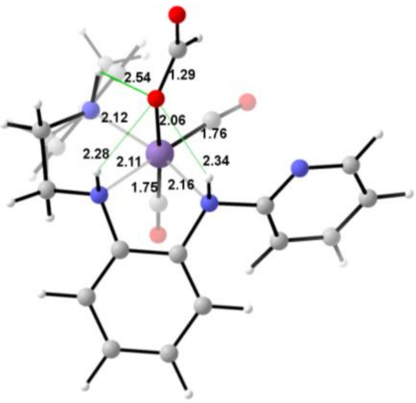

2c-IN3R

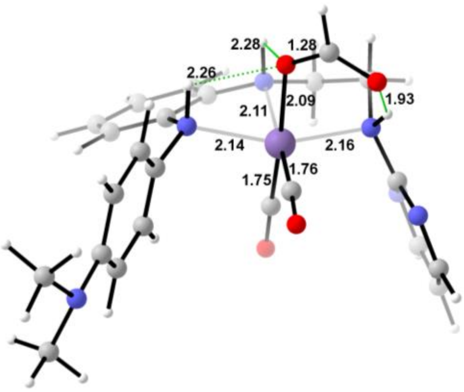

2d-IN3R

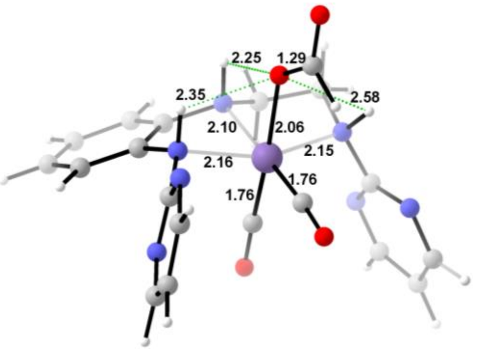

2e-IN3R

IN4

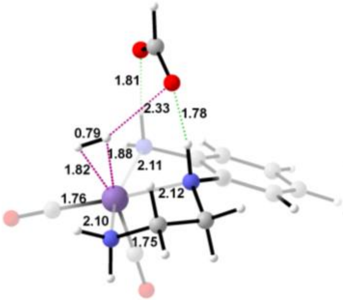

2a-IN4

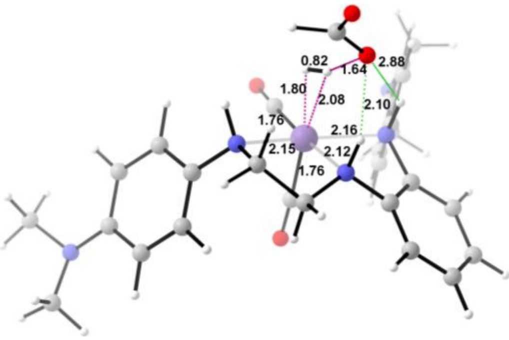

2b-IN4

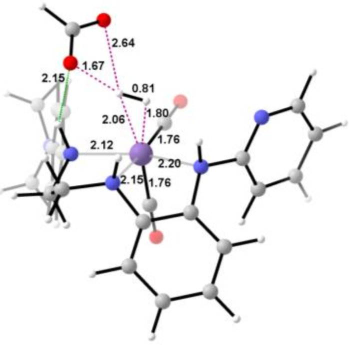

2c-IN4

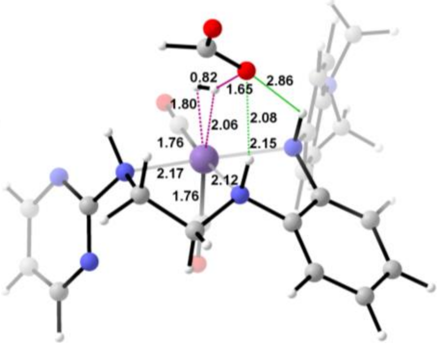

2d-IN4

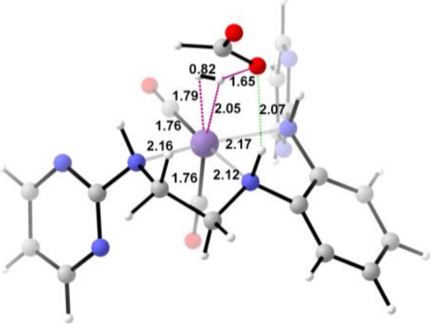

2e-IN4

TS[4-5]

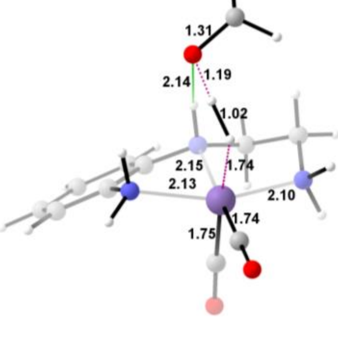

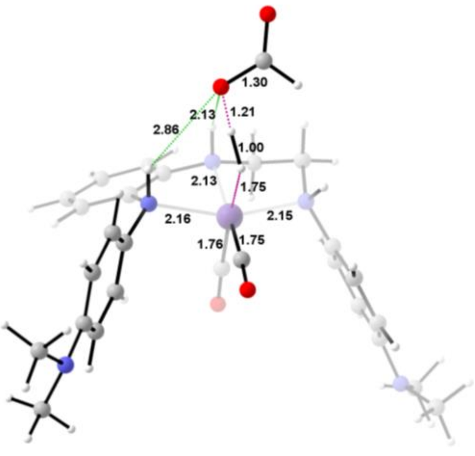

2a-TS[4-5] 2b-TS[4-5]

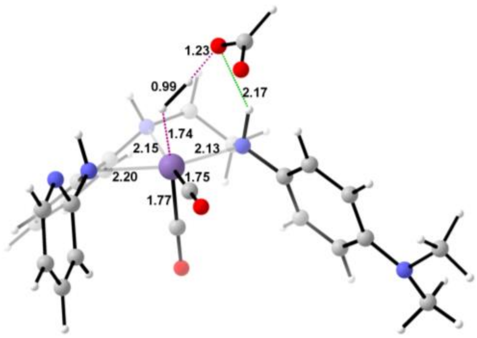

2c-TS[4-5]

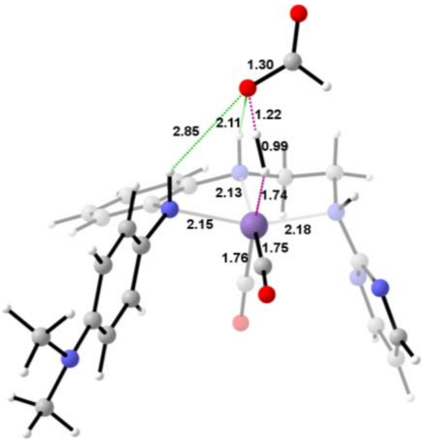

2d-TS[4-5]

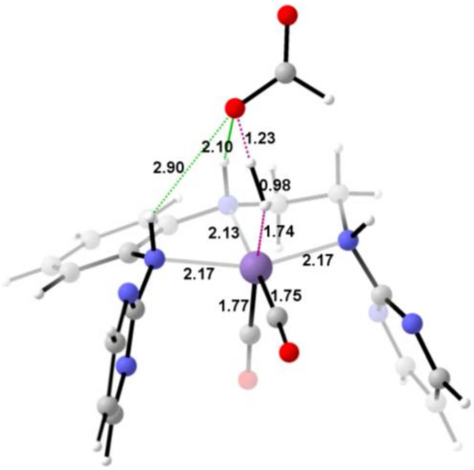

2e-TS[4-5]

IN5

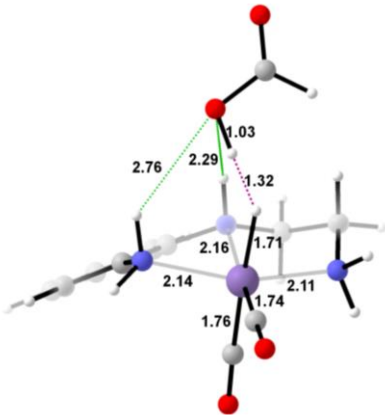

2a-IN5

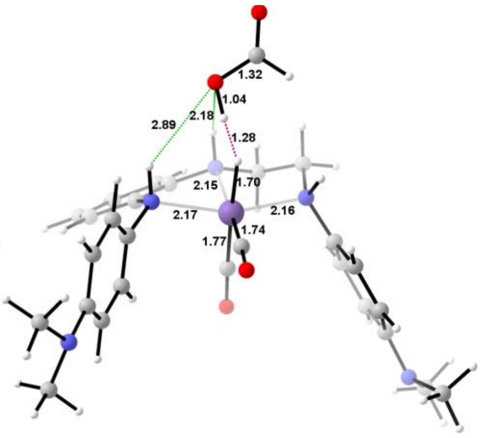

2b-IN5

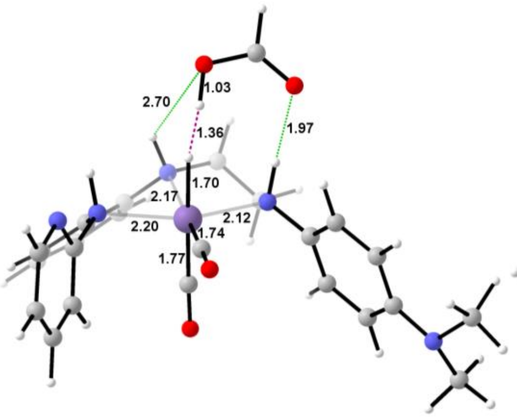

2c-IN5

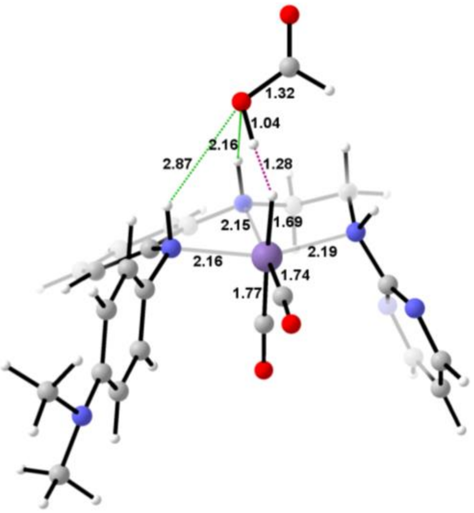

2d-IN5

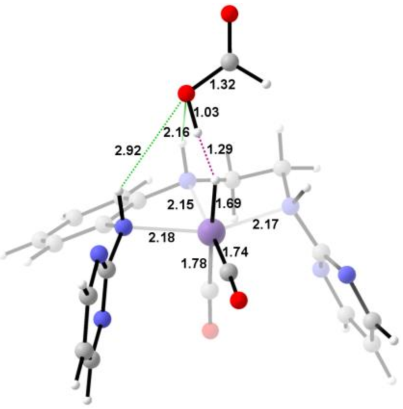

2e-IN5

GROUP-3

IN1

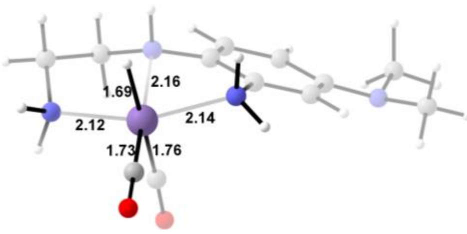

3a-IN1

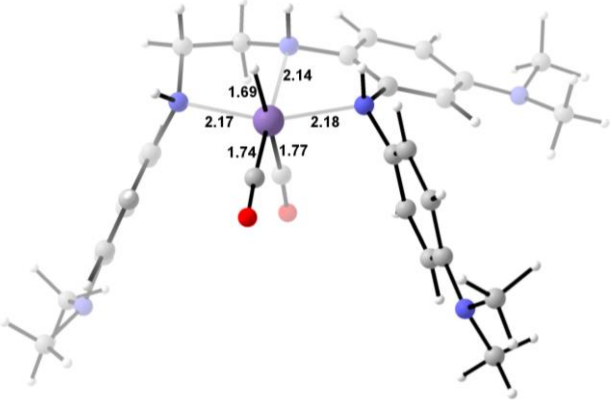

3b-IN1

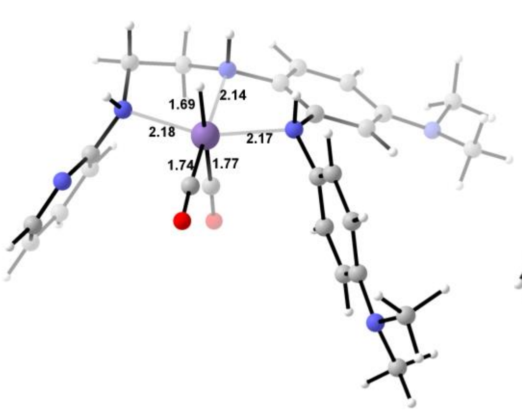

3c-IN1

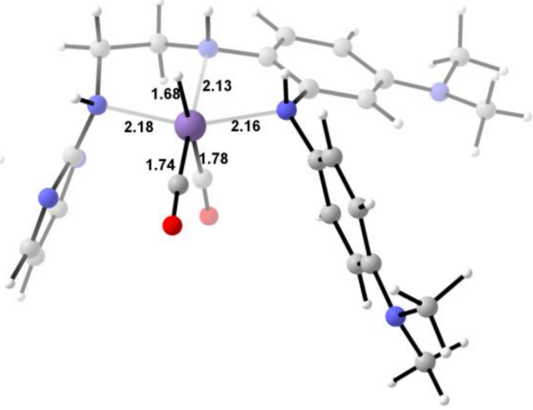

3d-IN1

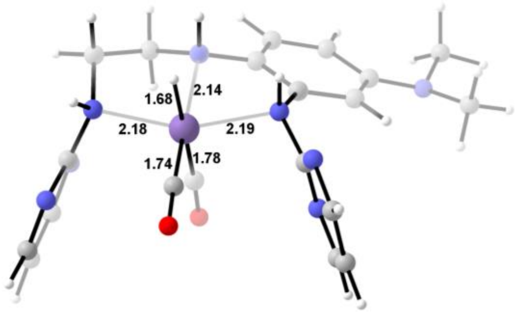

3e-IN1

IN2

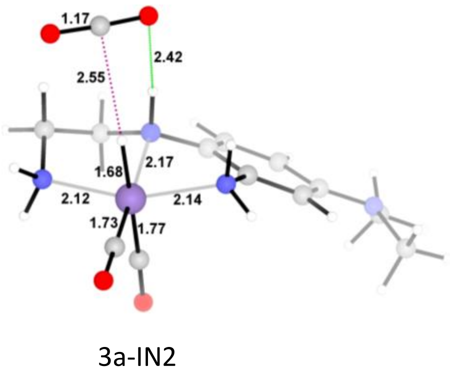

3a-IN2

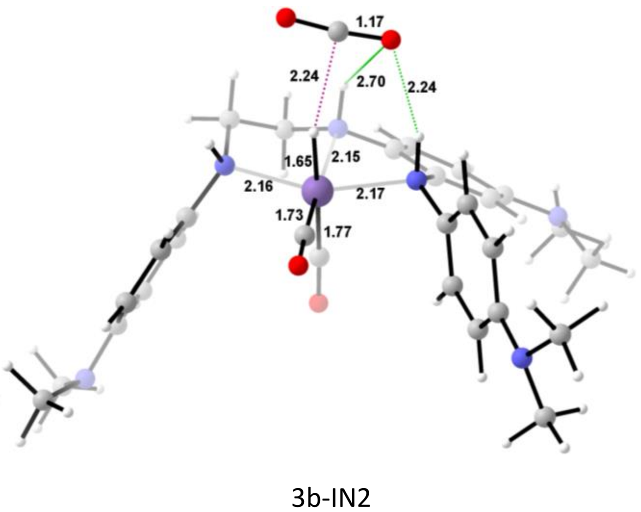

3b-IN2

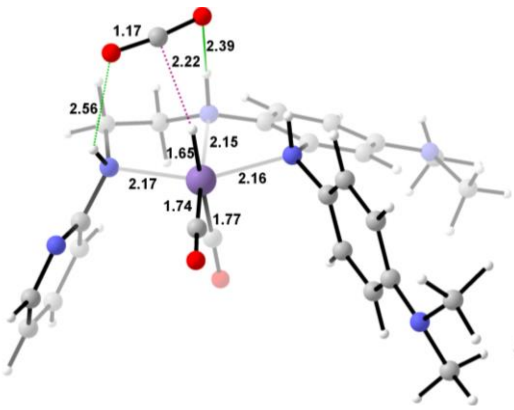

3c-IN2

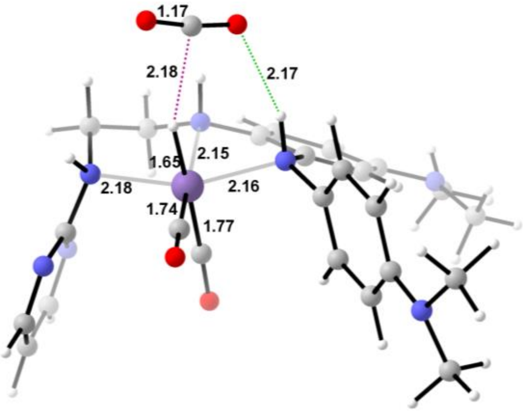

3d-IN2

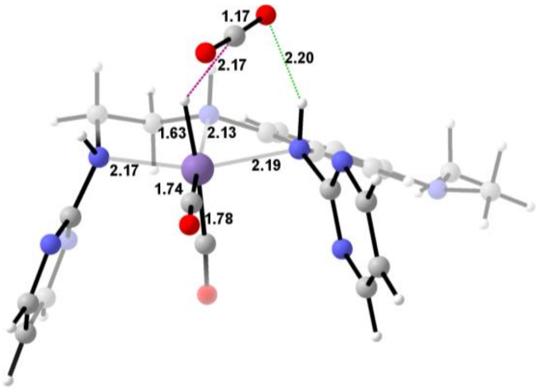

3e-IN2

TS[2-3]

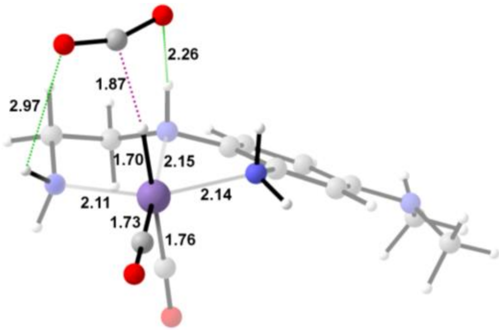

3a-TS[2-3]

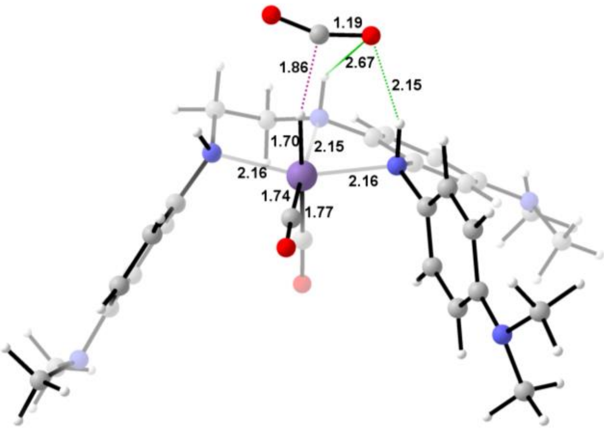

3b-TS[2-3]

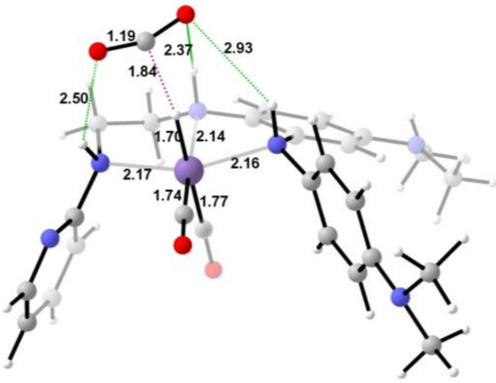

3c-TS[2-3]

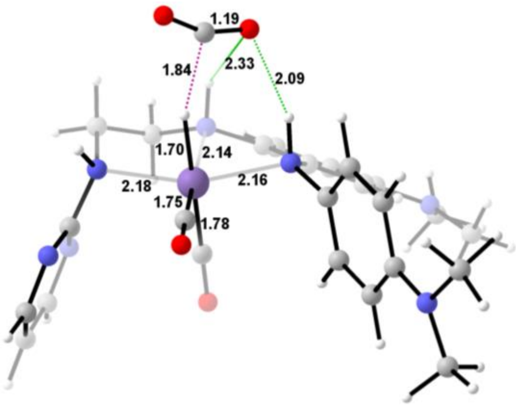

3d-TS[2=3]

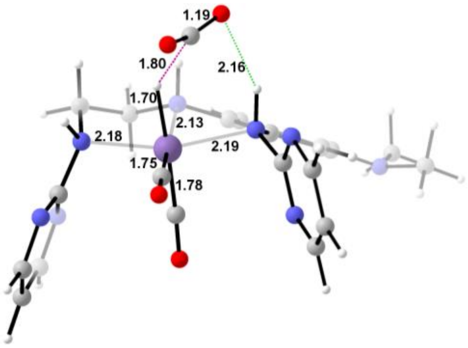

3e-TS[2=3]

IN3

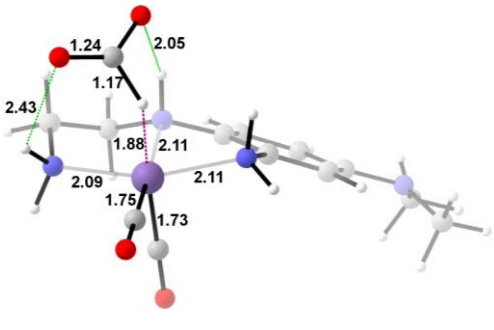

3a-IN3

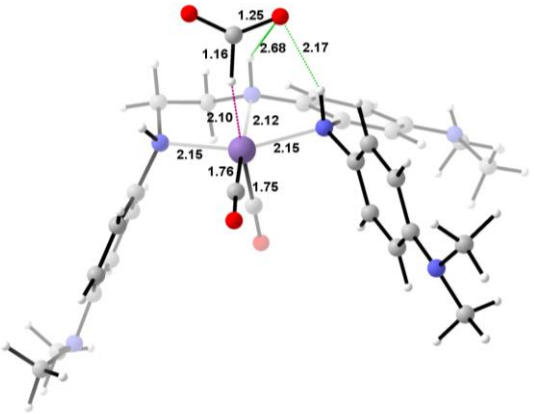

3b-IN3

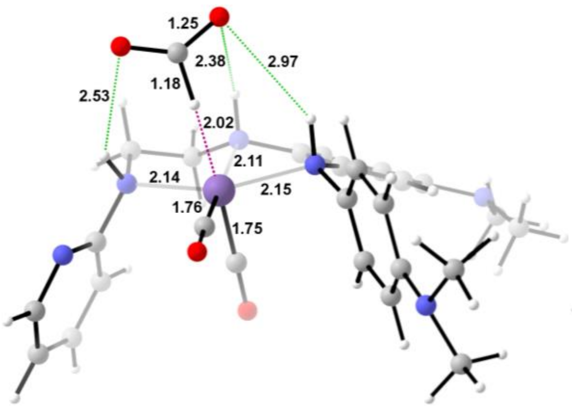

3c-IN3

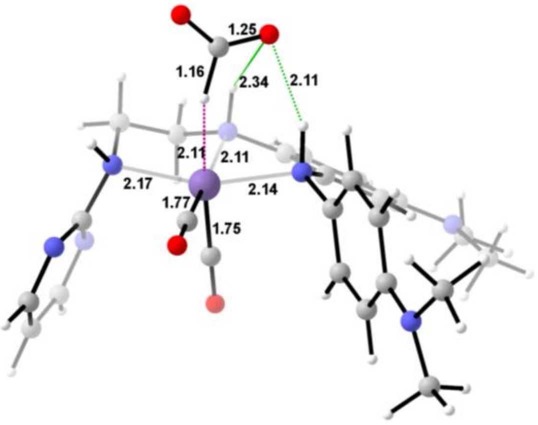

3d-IN3

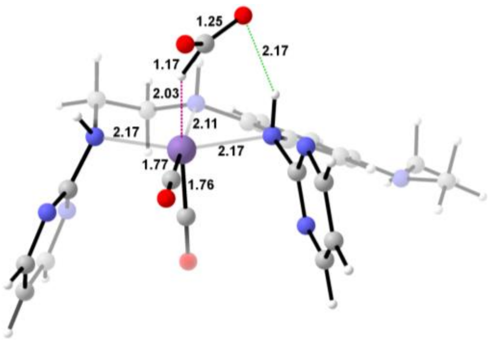

3e-IN3

IN3R

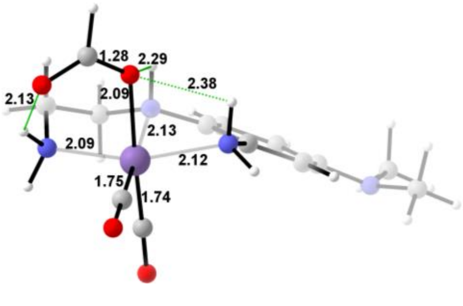

3a-IN3R

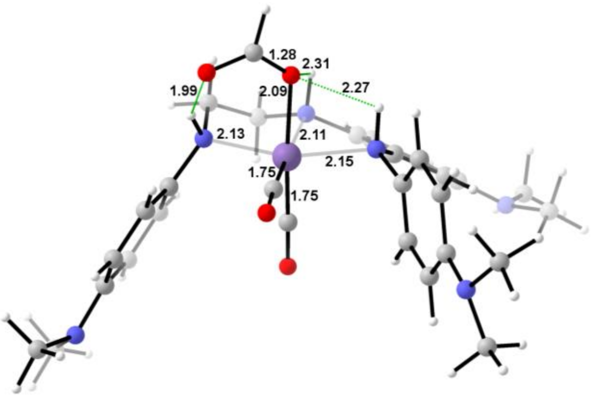

3b-IN3R

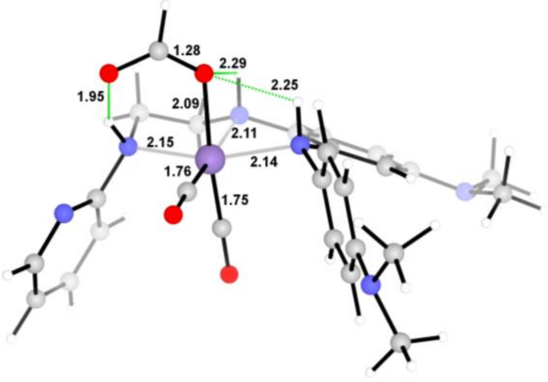

3c-IN3R

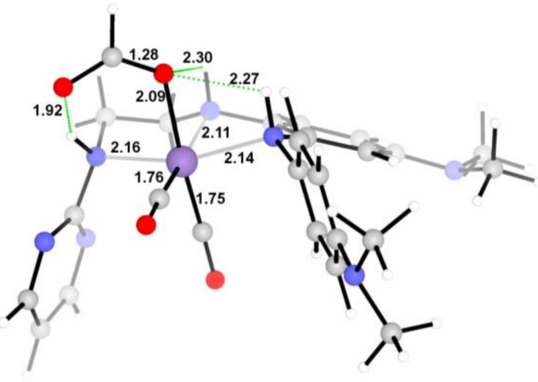

3d-IN3R

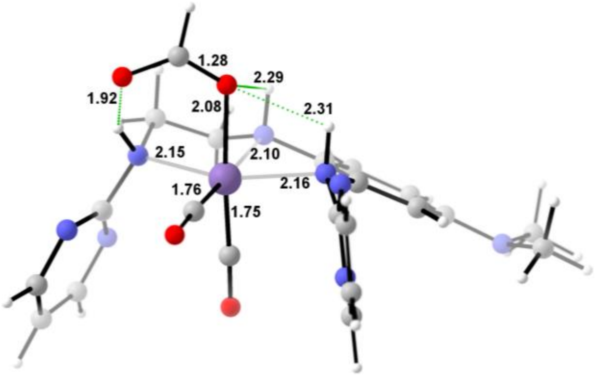

3e-IN3R

IN4

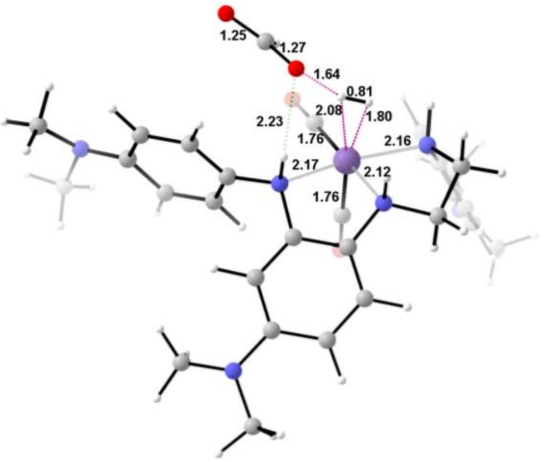

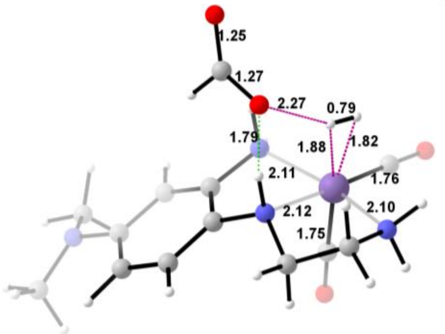

3a-IN4

3b-IN4

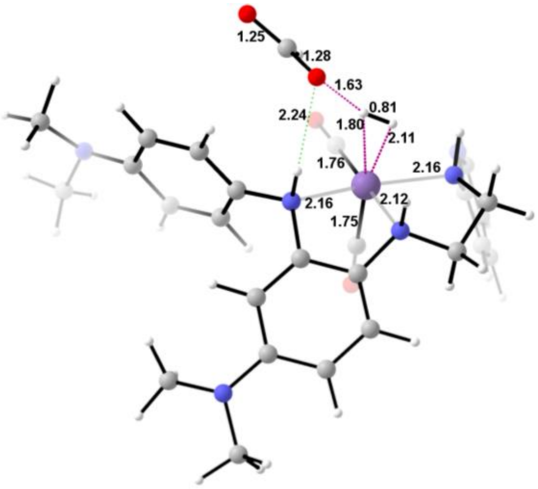

3c-IN4

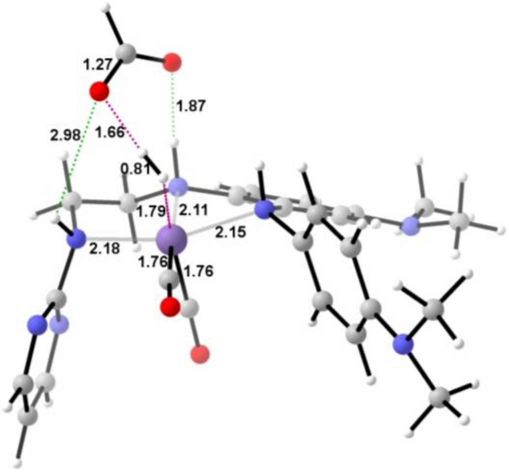

3d-IN4

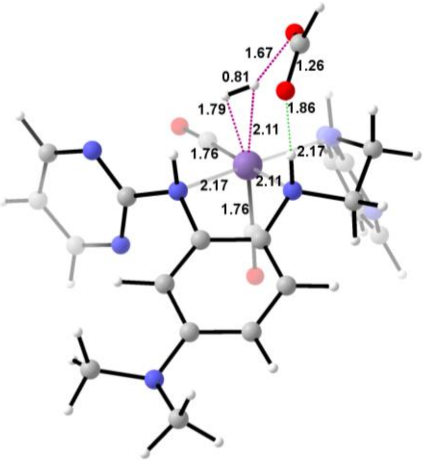

3e-IN4

TS[4-5]

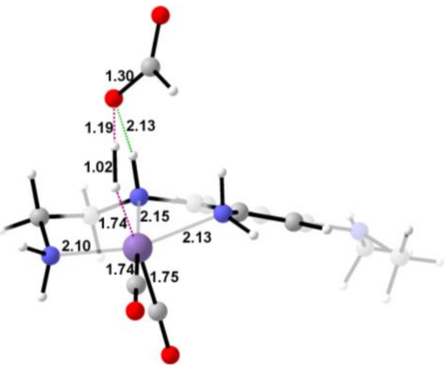

3a-TS[4-5]

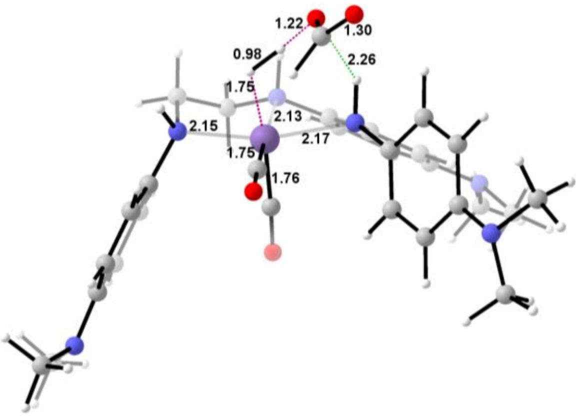

3b-TS[4-5]

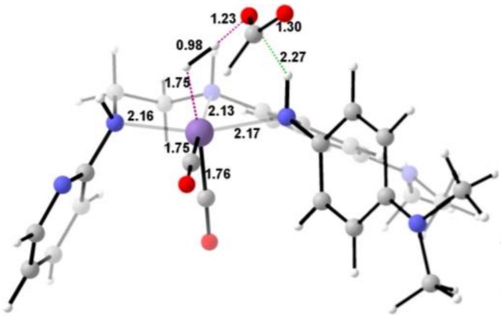

3c-TS[4-5]

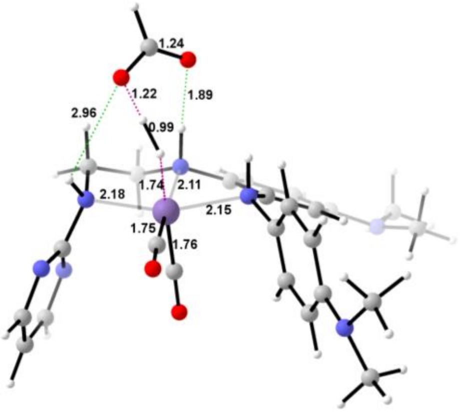

3d-TS[4-5]

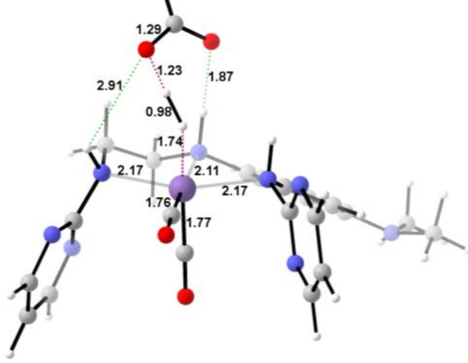

3e-TS[4-5]

IN5

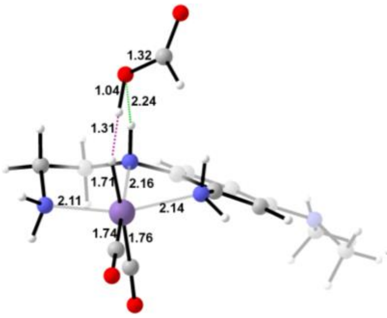

3a-IN5

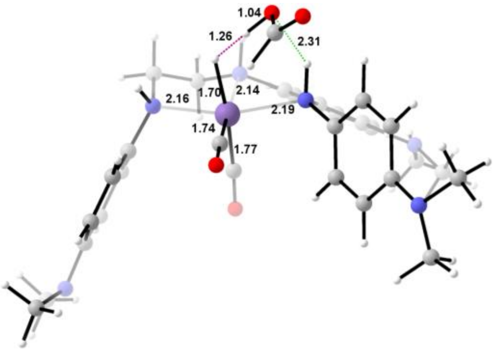

3b-IN5

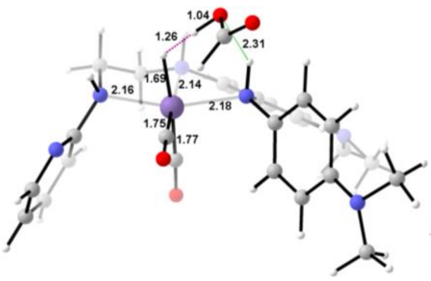

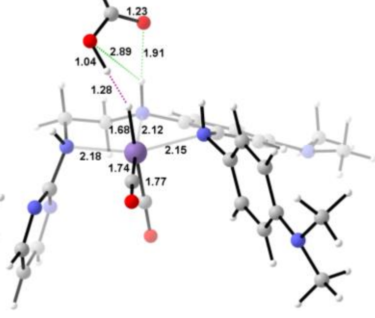

3c-IN5

3d-IN5

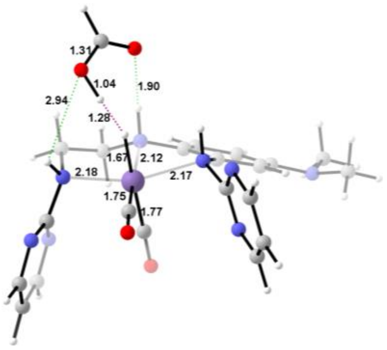

3e-IN5

NBO structures for TS[2-3] and IN4

TS[2-3]

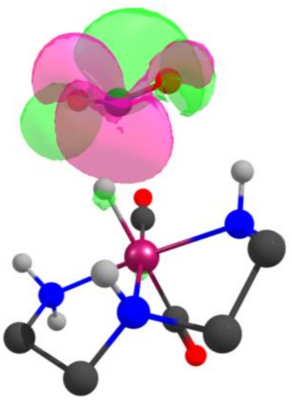

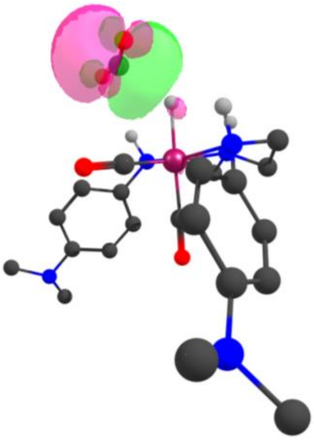

1a-TS[2-3] (13.53 kcal/mol)

1b-TS[2-3] (41.7 kcal/mol)

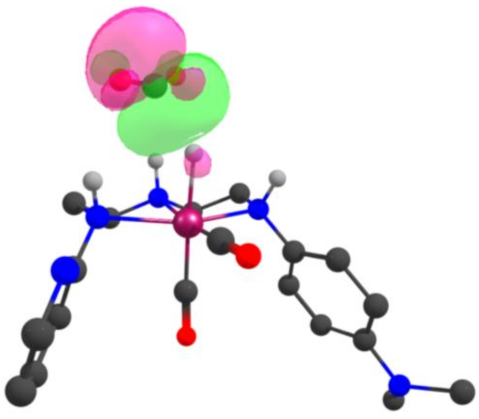

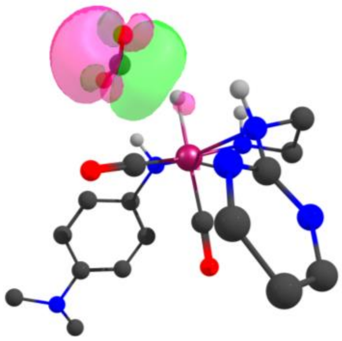

1c-TS[2-3] (36.7 kcal/mol)

1d-TS[2-3] (43.5 kcal/mol)

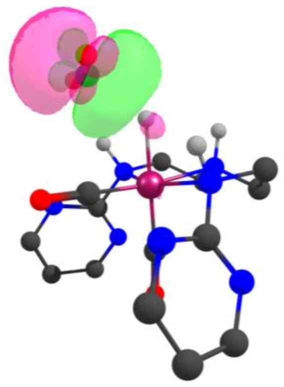

1e-TS[2-3] (45.01 kcal/mol)

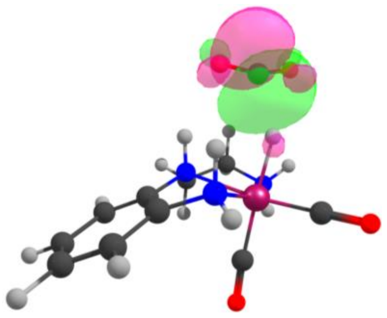

2a-TS[2-3] (33.6 kcal/mol)

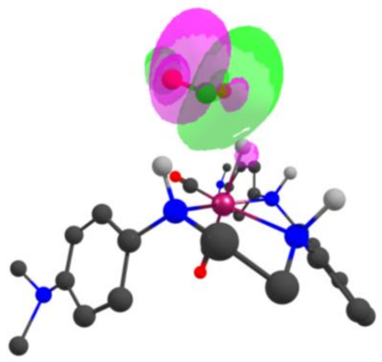

2b-TS[2-3] (43.2 kcal/mol)

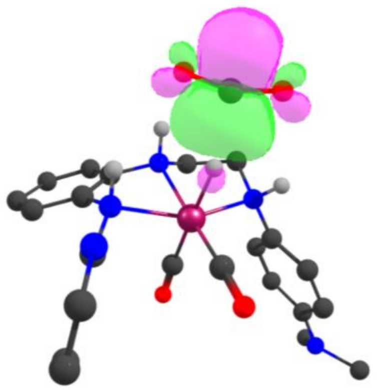

2c-TS[2-3] (36.64 kcal/mol)

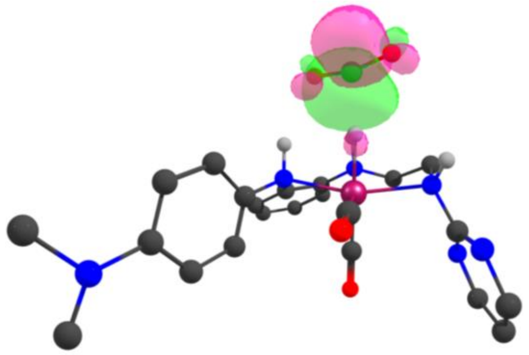

2d-TS[2-3] (34.98 kcal/mol)

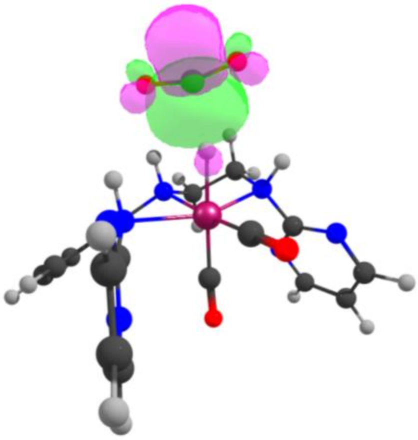

2e-TS[2-3] (37.79 kcal/mol)

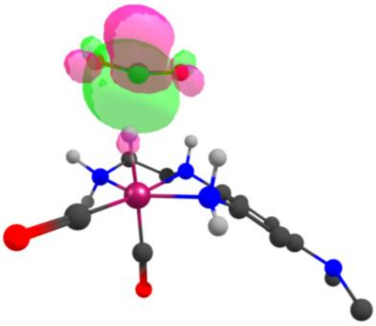

3a-TS[2-3] (33.09 kcal/mol)

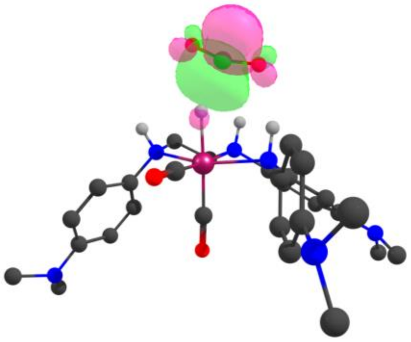

3b-TS[2-3] (33.84 kcal/mol)

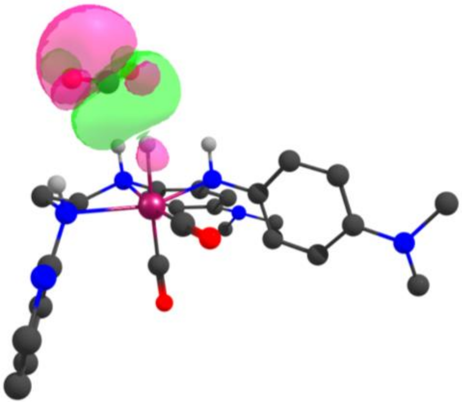

3c-TS[2-3] (36.13 kcal/mol)

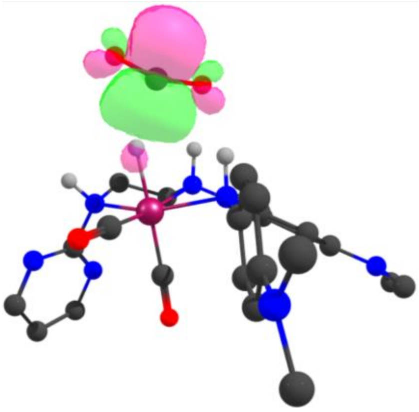

3d-TS[2-3] (37.16 kcal/mol)

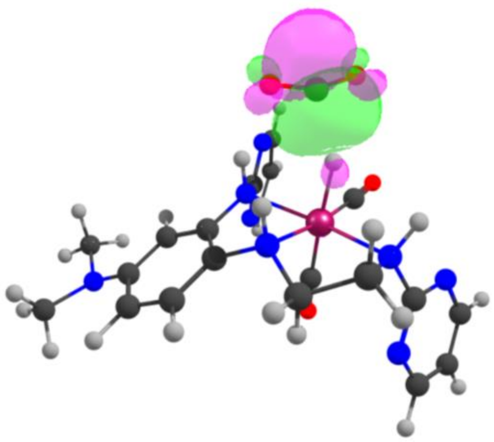

3e-TS[2-3] (42.41 kcal/mol)

IN4

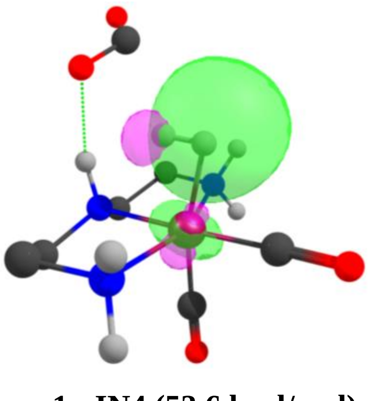

1a-IN4 (53.6 kcal/mol)

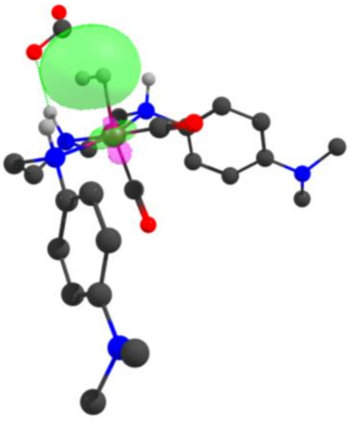

1b-IN4 (62.8 kcal/mol)

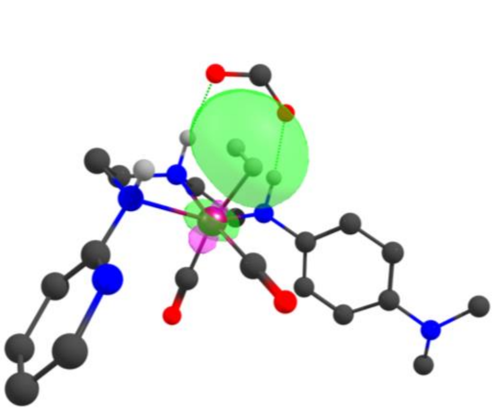

1c-IN4 (64.8 kcal/mol)

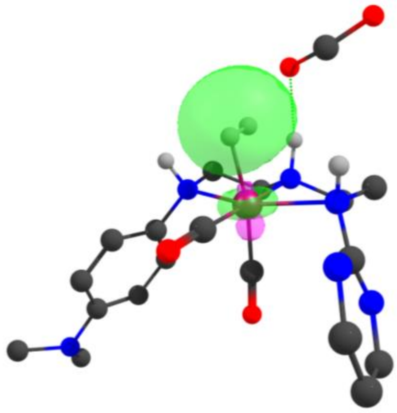

1d-IN4 (62.32 kcal/mol)

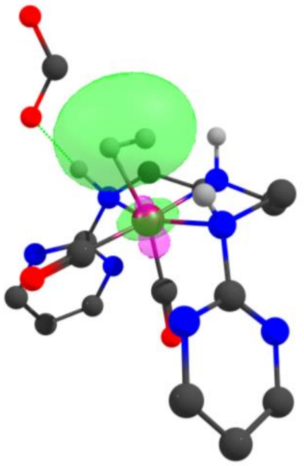

1e-IN4 (61.5 kcal/mol)

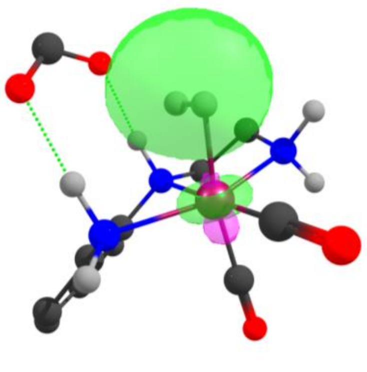

2a-IN4 (50.8 kcal/mol)

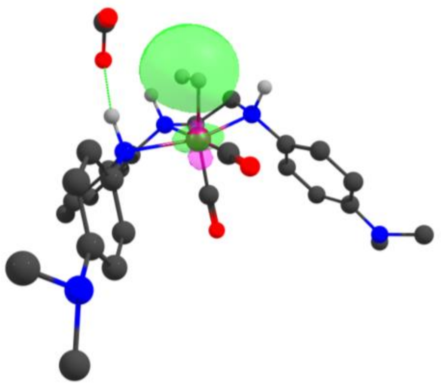

2b-IN4 (69.3 kcal/mol)

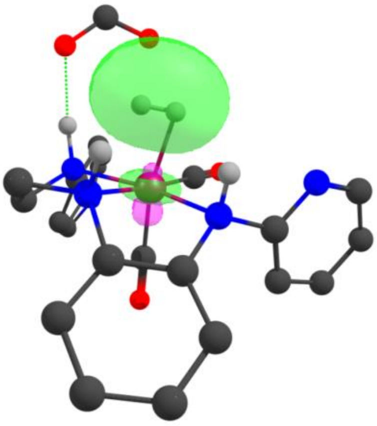

2c-IN4 (69.1 kcal/mol)

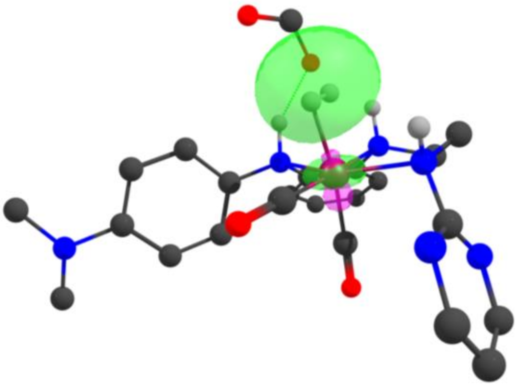

2d-IN4 (69.4 kcal/mol)

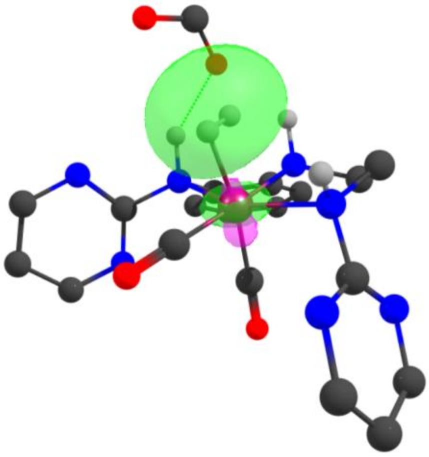

2e-IN4 (68.4 kcal/mol)

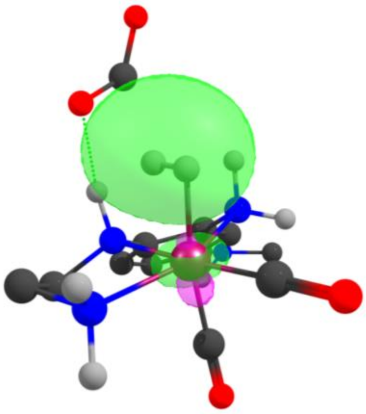

3a-IN4 (65.8 kcal/mol)

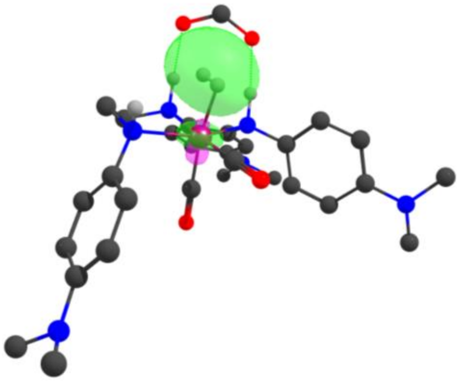

3b-IN4 (66.5 kcal/mol)

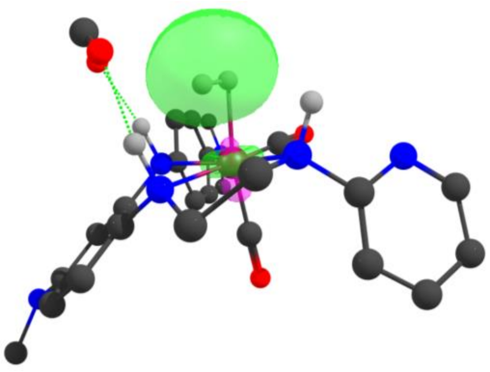

3c-IN4 (65.0 kcal/mol)

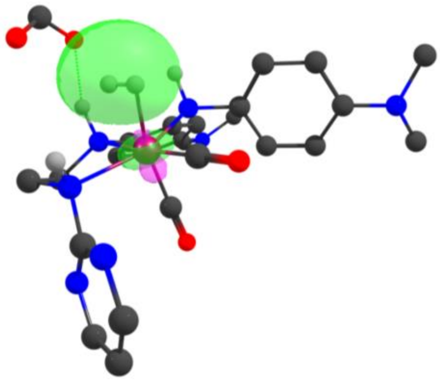

3d-IN4 (65.9 kcal/mol)

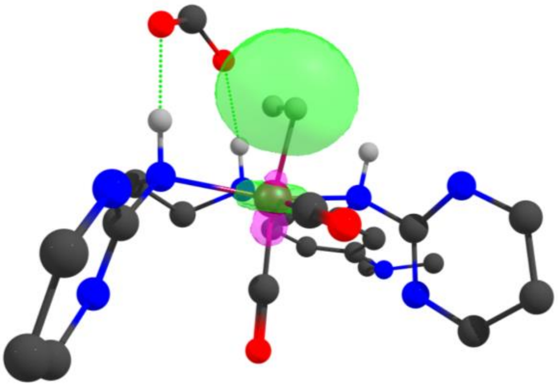

3e-IN4 (67.6 kcal/mol)

Coordinates of all Intermediates and Transition States

Group-1

1a-IN1

Energy: -656.000078 Hartree

N	-1.76787	2.43135	0.37723
N	0.71604	1.94979	-0.76524
Mn	-1.10567	0.78022	-0.76612
H	-2.09855	3.15982	-0.25201
C	-0.5975	-0.45688	-1.8833
O	-0.24269	-1.28993	-2.651
N	-3.23892	0.2577	-0.54937
C	-0.59402	2.94283	1.09016
H	-0.2933	2.18686	1.82464
H	-0.80556	3.87567	1.62981
C	0.47855	3.17468	0.04897
H	0.13726	3.95276	-0.64081
H	1.41044	3.52424	0.50483
H	-3.66749	0.20196	-1.45116
C	-0.59038	-0.21047	0.61282
O	-0.21445	-0.95254	1.45818

H	0.9526	2.20743	-1.70208
H	-1.57463	1.68628	-2.10994
C	-2.9	1.98007	1.19029
H	-3.38183	2.80594	1.73021
H	-2.52414	1.26089	1.92523
C	-3.88776	1.32636	0.25073
H	-4.74795	0.92891	0.79584
H	-4.25271	2.06667	-0.46696
H	1.46008	1.41598	-0.36342
H	-3.32669	-0.61958	-0.07748

1a-IN2

Energy: -844.574929 Hartree

H	1.08058	0.20016	-0.96002
N	-1.3846	0.97154	-0.77065
N	0.35227	1.4107	1.29671
Mn	-0.0507	-0.35792	0.20808
C	-1.22116	-0.92328	1.3872
O	-1.96947	-1.34181	2.20928
H	-0.88095	1.41642	-1.53552
C	2.88545	0.56285	-0.84309
O	3.34592	-0.41546	-1.33179
O	2.88392	1.64328	-0.34914
C	1.18254	-1.38762	0.87326
O	2.03916	-2.08464	1.31531
N	-0.81253	-1.6491	-1.30304
H	1.33848	1.65215	1.26342
C	-1.74525	2.01215	0.19646
H	-2.35241	1.54985	0.98392
H	-2.33301	2.82094	-0.25709
C	-0.44086	2.53817	0.75256
H	0.14052	2.98772	-0.06004
H	-0.61194	3.31451	1.50464
H	-0.08921	-2.05705	-1.88628
C	-1.77663	-0.92046	-2.16206
H	-2.50679	-1.59722	-2.61629
H	-1.2093	-0.45347	-2.9739
C	-2.45764	0.15919	-1.35112
H	-3.13743	0.74904	-1.98
H	-3.04192	-0.27178	-0.52946
H	-1.28747	-2.4292	-0.86001
H	0.11747	1.277	2.275

1a-TS[2-3]

Energy: -844.567253 Hartree

H	-1.55448	-2.1547	0.62351
N	-1.65366	-1.56025	-1.85968
N	0.76399	-1.7636	-0.53092
Mn	-1.02133	-0.67391	-0.05056
C	-0.46991	0.83164	-0.79819
O	-0.07025	1.85876	-1.23026
H	-2.01286	-2.49241	-1.66238
C	-1.12435	-2.83294	2.26607
O	-2.08188	-2.53011	2.89817
O	-0.06828	-3.33367	2.04864
C	-0.52366	-0.07827	1.51539
O	-0.17045	0.3202	2.57443
N	-3.14832	-0.12232	-0.01628
H	0.93822	-2.45171	0.17346
C	-0.45477	-1.70215	-2.6922
H	-0.11382	-0.6972	-2.9662
H	-0.65211	-2.25851	-3.6179
C	0.56787	-2.43324	-1.84976
H	0.19763	-3.44188	-1.64004
H	1.52168	-2.53494	-2.37646
H	-3.59322	-0.54007	0.7759
C	-3.77373	-0.62567	-1.26742
H	-4.60791	0.0158	-1.56207
H	-4.173	-1.61604	-1.03106
C	-2.75631	-0.74657	-2.38017
H	-3.22108	-1.19291	-3.26898
H	-2.35515	0.23363	-2.65738
H	-3.23891	0.87247	0.0305
H	1.53866	-1.13356	-0.58512

1a-IN3

Energy: -844.589418 Hartree

H	1.08058	0.20016	-0.96002
N	-1.3846	0.97154	-0.77065
N	0.35227	1.4107	1.29671
Mn	-0.0507	-0.35792	0.20808
C	-1.22116	-0.92328	1.3872
O	-1.96947	-1.34181	2.20928
H	-0.88095	1.41642	-1.53552
C	2.88545	0.56285	-0.84309
O	3.34592	-0.41546	-1.33179
O	2.88392	1.64328	-0.34914
C	1.18254	-1.38762	0.87326
O	2.03916	-2.08464	1.31531
N	-0.81253	-1.6491	-1.30304
H	1.33848	1.65215	1.26342
C	-1.74525	2.01215	0.19646
H	-2.35241	1.54985	0.98392
H	-2.33301	2.82094	-0.25709
C	-0.44086	2.53817	0.75256
H	0.14052	2.98772	-0.06004
H	-0.61194	3.31451	1.50464
H	-0.08921	-2.05705	-1.88628
C	-1.77663	-0.92046	-2.16206
H	-2.50679	-1.59722	-2.61629
H	-1.2093	-0.45347	-2.9739
C	-2.45764	0.15919	-1.35112
H	-3.13743	0.74904	-1.98
H	-3.04192	-0.27178	-0.52946
H	-1.28747	-2.4292	-0.86001
H	0.11747	1.277	2.275

1a-IN3R

Energy: -844.612632 Hartree

N	-1.64811	-1.64729	-1.61337
N	0.73358	-1.82182	-0.23979
Mn	-0.99864	-0.61253	0.07428
C	-0.37118	0.75037	-0.8239
O	0.07616	1.68926	-1.38586
H	-2.05459	-2.51576	-1.26294
C	-1.5516	-2.6242	2.21622
O	-2.03222	-3.60589	2.79672
O	-1.79622	-2.28443	1.00047
C	-0.45525	0.20538	1.5367
O	-0.05966	0.80257	2.47532
N	-3.07628	0.04382	0.0222
H	0.8758	-2.41895	0.54965
C	-0.46184	-1.97851	-2.40943
H	-0.05922	-1.04682	-2.82219
H	-0.6963	-2.64605	-3.24836
C	0.51063	-2.64906	-1.46381
H	0.07979	-3.59692	-1.12766
H	1.46126	-2.87454	-1.95624
H	-3.54803	-0.26479	0.84816
C	-3.72541	-0.55099	-1.17824
H	-4.51031	0.10955	-1.55342
H	-4.19499	-1.47928	-0.84151
C	-2.70832	-0.86188	-2.2535
H	-3.18574	-1.40302	-3.08031
H	-2.26067	0.05215	-2.65659
H	-0.8381	-1.96007	2.76134
H	-3.11322	1.04154	-0.03424
H	1.53436	-1.23699	-0.36911

1a-IN4

Energy: -845.747504 Hartree

H	-0.6489	-0.87512	-1.15037
N	-0.23835	1.38502	0.34422
N	1.33999	0.75014	-1.80405
Mn	0.71596	-0.43503	-0.16368
C	2.17091	-0.09981	0.74686
O	3.18815	0.06851	1.33203
H	-1.17453	1.39624	-0.06184

C	-3.41892	-0.74471	-0.47072
O	-4.6102	-0.55036	-0.24785
O	-2.63611	0.08771	-1.09917
C	1.37526	-1.97345	-0.64831
O	1.82335	-3.02191	-0.97609
N	-0.36107	-1.04304	1.56158
H	1.12636	0.33139	-2.70375
C	0.53434	2.46946	-0.27266
H	1.50264	2.5326	0.23798
H	0.03566	3.44233	-0.17759
C	0.70971	2.08992	-1.72504
H	-0.27424	2.02209	-2.20153
H	1.28955	2.84273	-2.26768
H	-1.02467	-1.78821	1.37106
H	-2.91649	-1.68035	-0.13836
H	-1.54806	-0.38755	-1.11332
C	-1.08218	0.10459	2.17079
H	-1.16836	-0.00615	3.25544
H	-2.09822	0.11683	1.76292
C	-0.38818	1.39727	1.80422
H	-0.96747	2.26076	2.15617
H	0.61136	1.45497	2.24995
H	0.29582	-1.42092	2.2366
H	2.34935	0.85805	-1.77807

1a-TS[4-5]

Energy: -845.738665 Hartree

H	1.63001	1.41203	1.43116
N	1.64709	1.50038	-1.31843
N	-0.6669	1.86609	0.17306
Mn	0.86985	0.36297	0.26974
C	-0.01623	-0.76496	-0.75362
O	-0.65667	-1.5533	-1.35714
H	2.24107	2.24245	-0.94522
C	4.24617	2.51414	1.37801
O	5.32569	3.09135	1.27633
O	3.11529	2.95978	0.91195
C	0.28187	-0.4943	1.68077
O	-0.12631	-1.07455	2.62579
N	2.81485	-0.61883	-0.00012
H	-0.71886	2.35416	1.04432
C	0.5042	2.12701	-1.99282
H	-0.06712	1.33682	-2.49288
H	0.81935	2.85066	-2.75521
C	-0.30555	2.81627	-0.91762
H	0.30516	3.60199	-0.46212
H	-1.2012	3.29147	-1.33003
H	3.37583	-0.48314	0.81651
H	4.16993	1.53346	1.90024
H	2.2746	2.115	1.14557
C	3.49398	-0.01526	-1.1793
H	4.10593	-0.76268	-1.68936
H	4.16275	0.75744	-0.78845
C	2.49306	0.61104	-2.12317
H	3.01515	1.14983	-2.92425
H	1.85132	-0.14813	-2.58203
H	2.67859	-1.59727	-0.15534
H	-1.54772	1.43775	-0.02864

1a-IN5

Energy: -845.737855 Hartree

H	-0.6489	-0.87512	-1.15037
N	-0.23835	1.38502	0.34422
N	1.33999	0.75014	-1.80405
Mn	0.71596	-0.43503	-0.16368
C	2.17091	-0.09981	0.74686
O	3.18815	0.06851	1.33203
H	-1.17453	1.39624	-0.06184
C	-3.41892	-0.74471	-0.47072
O	-4.6102	-0.55036	-0.24785
O	-2.63611	0.08771	-1.09917
C	1.37526	-1.97345	-0.64831
O	1.82335	-3.02191	-0.97609
N	-0.36107	-1.04304	1.56158

H	1.12636	0.33139	-2.70375
C	0.53434	2.46946	-0.27266
H	1.50264	2.5326	0.23798
H	0.03566	3.44233	-0.17759
C	0.70971	2.08992	-1.72504
H	-0.27424	2.02209	-2.20153
H	1.28955	2.84273	-2.26768
H	-1.02467	-1.78821	1.37106
H	-2.91649	-1.68035	-0.13836
H	-1.54806	-0.38755	-1.11332
C	-1.08218	0.10459	2.17079
H	-1.16836	-0.00615	3.25544
H	-2.09822	0.11683	1.76292
C	-0.38818	1.39727	1.80422
H	-0.96747	2.26076	2.15617
H	0.61136	1.45497	2.24995
H	0.29582	-1.42092	2.2366
H	2.34935	0.85805	-1.77807

1b-IN1

Energy: -1385.675418 Hartree

N	-1.76787	2.43135	0.37723
N	0.71604	1.94979	-0.76524
Mn	-1.10567	0.78022	-0.76612
H	-2.09855	3.15982	-0.25201
C	-0.5975	-0.45688	-1.8833
O	-0.24269	-1.28993	-2.651
N	-3.23892	0.2577	-0.54937
C	1.88324	1.18142	-0.42955
C	3.64876	-0.31628	-1.14745
C	3.45712	0.27932	1.17317
H	4.14137	-0.83012	-1.9652
H	3.7946	0.23471	2.2024
C	-0.59402	2.94283	1.09016
H	-0.2933	2.18686	1.82464
H	-0.80556	3.87567	1.62981
C	0.47855	3.17468	0.04897
H	0.13726	3.95276	-0.64081
H	1.41044	3.52424	0.50483
C	-3.30532	-1.05794	-0.04565
C	-3.17331	-3.2704	-0.48386
C	-3.35811	-2.46249	1.7218
C	-3.20708	-3.55152	0.87699
H	-3.1095	-4.06986	-1.21906
H	-3.44266	-2.59546	2.79854
H	-3.56742	0.2638	-1.50928
C	-0.59038	-0.21047	0.61282
O	-0.21445	-0.95254	1.45818
H	0.79699	2.22691	-1.73818
H	-1.57463	1.68628	-2.10994
C	2.35544	1.07533	0.87698
H	1.86682	1.61163	1.6859
C	4.12362	-0.45367	0.17307
C	2.54972	0.48096	-1.43549
H	2.20376	0.56581	-2.46376
N	5.19259	-1.30008	0.47818
C	6.07679	-1.65409	-0.61931
H	6.88209	-2.28152	-0.23563
H	5.54763	-2.23386	-1.37946
H	6.52625	-0.77813	-1.11268
C	5.87434	-1.05871	1.73843
H	5.20723	-1.23305	2.58624
H	6.70531	-1.75894	1.83038
H	6.27271	-0.03568	1.82563
C	-2.9	1.98007	1.19029
H	-3.38183	2.80594	1.73021
H	-2.52414	1.26089	1.92523
C	-3.88776	1.32636	0.25073
H	-4.74795	0.92891	0.79584
H	-4.25271	2.06667	-0.46696
C	-3.42145	-1.20112	1.27678
H	-3.5555	-0.36596	1.9321
C	-3.2306	-2.02704	-0.9686
H	-3.21759	-1.82242	-2.01877

N	-3.12753	-4.92764	1.38775
C	-4.11946	-5.11158	2.45693
H	-4.24712	-6.15641	2.64911
H	-3.77934	-4.62421	3.3467
H	-5.05393	-4.68801	2.15321
C	-3.40323	-5.87231	0.29575
H	-2.62199	-5.81295	-0.43298
H	-3.45043	-6.86677	0.68783
H	-4.33795	-5.62485	-0.16247

1b-IN2

Energy: -1574.251535 Hartree

H	0.01826	-2.83529	0.96737
N	0.00814	-2.77426	-1.59667
N	2.11648	-1.70019	-0.1446
Mn	-0.01456	-1.40268	0.01264
C	-0.04067	0.018	-1.0329
O	-0.05158	1.00672	-1.68606
H	0.01633	-3.72247	-1.22607
C	0.4176	-2.86681	2.77241
O	-0.67022	-2.88379	3.24614
O	1.60529	-2.88936	2.75015
C	-0.03925	-0.36206	1.41401
O	-0.05568	0.35562	2.35976
N	-2.1383	-1.78374	-0.15126
H	2.31587	-2.19421	0.72246
C	2.86181	-0.47238	-0.13546
C	3.89064	1.30933	1.14534
C	3.87563	1.41856	-1.25576
C	4.22153	2.01598	-0.02892
H	4.16081	1.70354	2.11837
H	4.1295	1.90246	-2.19206
C	1.26635	-2.55192	-2.31586
H	1.21378	-1.56383	-2.78714
H	1.43431	-3.29583	-3.10579
C	2.37374	-2.62384	-1.28544
H	2.40605	-3.6367	-0.8717
H	3.35046	-2.42115	-1.73649
C	-2.92152	-0.58022	-0.11351
C	-4.01486	1.13036	1.20961
C	-3.98199	1.31107	-1.1875
C	-4.35734	1.86056	0.05329
H	-4.3048	1.48753	2.19115
H	-4.24186	1.81587	-2.11103
H	-2.33144	-2.30515	0.69914
C	3.20291	0.20176	-1.3061
H	2.94511	-0.21322	-2.2769
C	3.22119	0.09491	1.08766
H	2.9733	-0.42677	2.01033
N	4.84286	3.26681	0.02518
C	5.54978	3.58916	1.2533
H	6.0164	4.56892	1.14493
H	4.86132	3.6493	2.09978
H	6.33441	2.85863	1.50534
C	5.51737	3.70959	-1.18315
H	4.80602	3.85322	-2.00024
H	5.98997	4.67353	-0.99089
H	6.29229	3.0072	-1.52838
C	-2.36471	-2.68877	-1.31475
H	-3.33902	-2.49246	-1.77353
H	-2.38566	-3.70986	-0.92181
C	-1.24645	-2.57823	-2.32952
H	-1.38586	-3.31966	-3.12737
H	-1.2144	-1.58533	-2.79231
C	-3.30823	-0.06101	1.12213
H	-3.05072	-0.60207	2.03138
C	-3.2715	0.11762	-1.26743
H	-2.9897	-0.26034	-2.24684
N	-5.01748	3.08964	0.13749
C	-5.73673	3.35843	1.37128
H	-5.05204	3.41817	2.2209
H	-6.23336	4.32567	1.28661
H	-6.49861	2.59756	1.60246
C	-5.70253	3.54173	-1.0615

H	-6.20596	4.485	-0.84654
H	-4.99406	3.72847	-1.87228
H	-6.45383	2.82414	-1.42701

1b-TS[2-3]

Energy: -1574.244115 Hartree

H	-1.55448	-2.1547	0.62351
N	-1.65366	-1.56025	-1.85968
N	0.76399	-1.7636	-0.53092
Mn	-1.02133	-0.67391	-0.05056
C	-0.46991	0.83164	-0.79819
O	-0.07025	1.85876	-1.23026
H	-2.01286	-2.49241	-1.66238
C	-1.12435	-2.83294	2.26607
O	-2.08188	-2.53011	2.89817
O	-0.06828	-3.33367	2.04864
C	-0.52366	-0.07827	1.51539
O	-0.17045	0.3202	2.57443
N	-3.14832	-0.12232	-0.01628
H	0.75997	-2.47813	0.19397
C	1.96705	-0.99176	-0.38229
C	3.72228	-0.15877	1.06513
C	3.65348	0.51528	-1.24205
C	4.27813	0.60298	0.01695
H	4.17759	-0.15511	2.04902
H	4.05297	1.0583	-2.09101
C	-0.45477	-1.70215	-2.6922
H	-0.11382	-0.6972	-2.9662
H	-0.65211	-2.25851	-3.6179
C	0.56787	-2.43324	-1.84976
H	0.19763	-3.44188	-1.64004
H	1.52168	-2.53494	-2.37646
C	-3.21962	1.26518	0.23171
C	-3.14253	2.92985	1.75627
C	-3.22246	3.38043	-0.55586
C	-3.12432	3.87399	0.7364
H	-3.12221	3.23298	2.8008
H	-3.26398	4.05177	-1.41119
H	-3.49619	-0.62267	0.79646
C	2.51602	-0.26168	-1.4342
H	2.05964	-0.28913	-2.4203
C	2.58847	-0.93413	0.86535
H	2.17774	-1.50946	1.69295
N	5.38551	1.42854	0.22565
C	6.21297	1.12828	1.38191
H	7.05813	1.8173	1.39956
H	5.65727	1.27219	2.31165
H	6.60559	0.09948	1.37722
C	6.13275	1.82573	-0.95541
H	5.52012	2.43995	-1.6199
H	6.98488	2.43238	-0.64703
H	6.50966	0.96975	-1.53684
C	-3.77373	-0.62567	-1.26742
H	-4.60791	0.0158	-1.56207
H	-4.173	-1.61604	-1.03106
C	-2.75631	-0.74657	-2.38017
H	-3.22108	-1.19291	-3.26898
H	-2.35515	0.23363	-2.65738
C	-3.19919	1.61514	1.52475
H	-3.2262	0.89571	2.31633
C	-3.28368	2.07121	-0.83029
H	-3.37718	1.69563	-1.82784
N	-3.04598	5.3153	1.01458
C	-3.36931	5.55991	2.42757
H	-2.60211	5.14338	3.04629
H	-3.43468	6.61369	2.60124
H	-4.30647	5.1007	2.66368
C	-4.00485	6.03186	0.16135
H	-4.10842	7.03859	0.50872
H	-3.64935	6.03757	-0.84785
H	-4.95474	5.54099	0.20212

1b-IN3

Energy: -1574.267304 Hartree

H	0.01826	-2.83529	0.96737
N	0.00814	-2.77426	-1.59667
N	2.11648	-1.70019	-0.1446
Mn	-0.01456	-1.40268	0.01264
C	-0.04067	0.018	-1.0329
O	-0.05158	1.00672	-1.68606
H	0.01633	-3.72247	-1.22607
C	0.4176	-2.86681	2.77241
O	-0.67022	-2.88379	3.24614
O	1.60529	-2.88936	2.75015
C	-0.03925	-0.36206	1.41401
O	-0.05568	0.35562	2.35976
N	-2.1383	-1.78374	-0.15126
H	2.31587	-2.19421	0.72246
C	2.86181	-0.47238	-0.13546
C	3.89064	1.30933	1.14534
C	3.87563	1.41856	-1.25576
C	4.22153	2.01598	-0.02892
H	4.16081	1.70354	2.11837
H	4.1295	1.90246	-2.19206
C	1.26635	-2.55192	-2.31586
H	1.21378	-1.56383	-2.78714
H	1.43431	-3.29583	-3.10579
C	2.37374	-2.62384	-1.28544
H	2.40605	-3.6367	-0.8717
H	3.35046	-2.42115	-1.73649
C	-2.92152	-0.58022	-0.11351
C	-4.01486	1.13036	1.20961
C	-3.98199	1.31107	-1.1875
C	-4.35734	1.86056	0.05329
H	-4.3048	1.48753	2.19115
H	-4.24186	1.81587	-2.11103
H	-2.33144	-2.30515	0.69914
C	3.20291	0.20176	-1.3061
H	2.94511	-0.21322	-2.2769
C	3.22119	0.09491	1.08766
H	2.9733	-0.42677	2.01033
N	4.84286	3.26681	0.02518
C	5.54978	3.58916	1.2533
H	6.0164	4.56892	1.14493
H	4.86132	3.6493	2.09978
H	6.33441	2.85863	1.50534
C	5.51737	3.70959	-1.18315
H	4.80602	3.85322	-2.00024
H	5.98997	4.67353	-0.99089
H	6.29229	3.0072	-1.52838
C	-2.36471	-2.68877	-1.31475
H	-3.33902	-2.49246	-1.77353
H	-2.38566	-3.70986	-0.92181
C	-1.24645	-2.57823	-2.32952
H	-1.38586	-3.31966	-3.12737
H	-1.2144	-1.58533	-2.79231
C	-3.30823	-0.06101	1.12213
H	-3.05072	-0.60207	2.03138
C	-3.2715	0.11762	-1.26743
H	-2.9897	-0.26034	-2.24684
N	-5.01748	3.08964	0.13749
C	-5.73673	3.35843	1.37128
H	-5.05204	3.41817	2.2209
H	-6.23336	4.32567	1.28661
H	-6.49861	2.59756	1.60246
C	-5.70253	3.54173	-1.0615
H	-6.20596	4.485	-0.84654
H	-4.99406	3.72847	-1.87228
H	-6.45383	2.82414	-1.42701

1b-IN3R

Energy: -1574.290234 Hartree

N	-1.64811	-1.64729	-1.61337
N	0.73358	-1.82182	-0.23979
Mn	-0.99864	-0.61253	0.07428
C	-0.37118	0.75037	-0.8239
O	0.07616	1.68926	-1.38586
H	-2.05459	-2.51576	-1.26294

C	-1.5516	-2.6242	2.21622
O	-2.03222	-3.60589	2.79672
O	-1.79622	-2.28443	1.00047
C	-0.45525	0.20538	1.5367
O	-0.05966	0.80257	2.47532
N	-3.07628	0.04382	0.0222
H	0.70519	-2.44345	0.56551
C	1.97125	-1.09017	-0.18159
C	3.74007	-0.1352	1.16987
C	3.73588	0.20437	-1.21086
C	4.34585	0.44372	0.03559
H	4.1811	-0.01137	2.1523
H	4.17166	0.60276	-2.11982
C	-0.46184	-1.97851	-2.40943
H	-0.05922	-1.04682	-2.82219
H	-0.6963	-2.64605	-3.24836
C	0.51063	-2.64906	-1.46381
H	0.07979	-3.59692	-1.12766
H	1.46126	-2.87454	-1.95624
C	-3.10044	1.45033	0.14505
C	-2.97313	3.2387	1.51793
C	-2.99552	3.48417	-0.82635
C	-2.89667	4.0859	0.41898
H	-2.95531	3.6324	2.53178
H	-2.99226	4.07775	-1.73812
H	-3.4381	-0.37364	0.87587
C	2.56715	-0.54245	-1.31504
H	2.12629	-0.69059	-2.29729
C	2.57472	-0.88004	1.05793
H	2.12386	-1.31161	1.94992
N	5.48678	1.24024	0.14776
C	6.28092	1.07724	1.35385
H	7.15177	1.73086	1.29397
H	5.71472	1.37149	2.24084
H	6.63293	0.04464	1.50308
C	6.26877	1.4338	-1.06157
H	5.69649	1.97706	-1.81772
H	7.14535	2.03684	-0.82253
H	6.61119	0.4883	-1.51021
C	-3.72541	-0.55099	-1.17824
H	-4.51031	0.10955	-1.55342
H	-4.19499	-1.47928	-0.84151
C	-2.70832	-0.86188	-2.2535
H	-3.18574	-1.40302	-3.08031
H	-2.26067	0.05215	-2.65659
H	-0.8381	-1.96007	2.76134
C	-4.14593	6.25423	0.66787
H	-4.04358	7.31054	0.80437
H	-4.68667	6.06204	-0.23521
H	-4.67758	5.83724	1.49755
C	-1.9576	5.91973	1.86102
H	-1.72905	6.96444	1.89636
H	-2.54485	5.654	2.71509
H	-1.04849	5.35545	1.86371
C	-3.08617	1.9123	1.40175
H	-3.15984	1.26753	2.25248
C	-3.10927	2.15901	-0.98526
H	-3.20071	1.6991	-1.94704
N	-2.74978	5.6107	0.57706

1a-IN4

Energy: -1575.422955 Hartree

H	-0.3081	-2.42236	1.4661
N	-0.21467	-2.58147	-1.2806
N	1.98236	-1.79621	0.22787
Mn	-0.08448	-1.16861	0.27491
C	0.17917	0.19931	-0.79625
O	0.37159	1.16531	-1.45168
H	-0.40267	-3.5057	-0.88961
C	-1.99596	-4.71504	1.28948
O	-2.60441	-5.77207	1.14949
O	-0.73899	-4.5366	0.99548
C	-0.01104	-0.088	1.64854
O	0.03352	0.65259	2.571

N	-2.227	-1.18997	-0.01077
H	2.10484	-2.25074	1.12809
C	2.90533	-0.69408	0.16681
C	4.11876	1.04686	1.33682
C	4.26227	0.89205	-1.05742
H	4.38795	1.50153	2.28341
H	4.64194	1.22712	-2.01586
C	1.09913	-2.62149	-1.93323
H	1.2468	-1.66802	-2.45306
H	1.16901	-3.42609	-2.67646
C	2.11969	-2.83018	-0.83616
H	1.93562	-3.79866	-0.36067
H	3.13967	-2.84618	-1.23374
C	-2.79366	0.1321	-0.04107
C	-3.67587	2.03226	1.17576
C	-3.44588	2.14492	-1.21469
C	-3.81154	2.77964	-0.01201
H	-3.96975	2.45613	2.12918
H	-3.55254	2.66105	-2.16192
H	-2.56617	-1.64758	0.83133
H	-2.50342	-3.81152	1.69587
H	-0.47923	-3.38442	1.20647
C	3.41492	-0.21146	-1.03626
H	3.15824	-0.68978	-1.97736
C	4.62341	1.56368	0.12556
C	3.27412	-0.05384	1.35029
H	2.89616	-0.42899	2.29923
N	5.4263	2.70643	0.10037
C	6.10038	3.05739	1.33919
H	6.71787	3.93959	1.16695
H	5.38051	3.31243	2.12065
H	6.74663	2.25253	1.72279
C	6.22908	2.91103	-1.09334
H	5.59652	3.06686	-1.97073
H	6.83156	3.81073	-0.96364
H	6.90574	2.06929	-1.30876
C	-2.5484	-2.06802	-1.1745
H	-3.43308	-1.6987	-1.70231
H	-2.79867	-3.0557	-0.77356
C	-1.36486	-2.19772	-2.10773
H	-1.57859	-2.93695	-2.89055
H	-1.1278	-1.24616	-2.59696
C	-3.17317	0.73858	1.15606
H	-3.0752	0.18558	2.08838
C	-2.93984	0.84933	-1.22629
H	-2.65666	0.40604	-2.17755
N	-4.26475	4.10092	0.00716
C	-4.77561	4.63153	-1.24533
H	-5.13112	5.64892	-1.07909
H	-3.98838	4.68216	-2.0016
H	-5.60569	4.03815	-1.65957
C	-5.01457	4.51625	1.18073
H	-4.39081	4.48703	2.0775
H	-5.33821	5.54891	1.04534
H	-5.90543	3.89627	1.36682

1a-TS[4-5]

Energy: -1575.414517 Hartree

H	1.63001	1.41203	1.43116
N	1.64709	1.50038	-1.31843
N	-0.6669	1.86609	0.17306
Mn	0.86985	0.36297	0.26974
C	-0.01623	-0.76496	-0.75362
O	-0.65667	-1.5533	-1.35714
H	2.24107	2.24245	-0.94522
C	4.24617	2.51414	1.37801
O	5.32569	3.09135	1.27633
O	3.11529	2.95978	0.91195
C	0.28187	-0.4943	1.68077
O	-0.12631	-1.07455	2.62579
N	2.81485	-0.61883	-0.00012
H	-0.5654	2.35151	1.06001
C	-1.99989	1.32333	0.12709
C	-3.88848	0.39435	1.32746

C	-3.93988	0.51671	-1.07288
H	-4.34179	0.1531	2.28216
H	-4.43229	0.36758	-2.027
C	0.5042	2.12701	-1.99282
H	-0.06712	1.33682	-2.49288
H	0.81935	2.85066	-2.75521
C	-0.30555	2.81627	-0.91762
H	0.30516	3.60199	-0.46212
H	-1.2012	3.29147	-1.33003
C	2.57442	-2.01109	-0.03744
C	2.1953	-3.90545	1.13134
C	2.03456	-3.8554	-1.2199
C	1.89673	-4.58073	-0.04601
H	2.16422	-4.41384	2.09247
H	1.86797	-4.32003	-2.18955
H	3.31358	-0.39625	0.85704
H	4.16993	1.53346	1.90024
H	2.2746	2.115	1.14557
C	-2.6749	1.09582	-1.07001
H	-2.22477	1.36642	-2.0211
C	-4.57458	0.13239	0.12344
C	-2.62708	0.972	1.32261
H	-2.12005	1.16101	2.2667
N	-5.81848	-0.50087	0.11834
C	-6.57726	-0.46213	1.35711
H	-7.53388	-0.96178	1.20005
H	-6.05738	-0.99916	2.15417
H	-6.77554	0.56162	1.71073
C	-6.62346	-0.34682	-1.08144
H	-6.13802	-0.81167	-1.94317
H	-7.5777	-0.85356	-0.93372
H	-6.82592	0.70582	-1.33338
C	3.49398	-0.01526	-1.1793
H	4.10593	-0.76268	-1.68936
H	4.16275	0.75744	-0.78845
C	2.49306	0.61104	-2.12317
H	3.01515	1.14983	-2.92425
H	1.85132	-0.14813	-2.58203
C	2.54802	-2.61669	1.15648
H	2.79133	-2.10731	2.06546
C	2.38546	-2.56302	-1.23722
H	2.5063	-2.01046	-2.1455
N	1.49631	-5.99514	-0.049
C	2.26741	-6.72054	-1.06885
H	2.18188	-7.77346	-0.8987
H	1.88548	-6.484	-2.03997
H	3.29615	-6.43218	-1.01023
C	1.75907	-6.58159	1.27309
H	1.13705	-6.10775	2.00347
H	1.5443	-7.6295	1.24751
H	2.78714	-6.4344	1.53062

1b-IN5

Energy: -1575.413492 Hartree

H	-0.3081	-2.42236	1.4661
N	-0.21467	-2.58147	-1.2806
N	1.98236	-1.79621	0.22787
Mn	-0.08448	-1.16861	0.27491
C	0.17917	0.19931	-0.79625
O	0.37159	1.16531	-1.45168
H	-0.40267	-3.5057	-0.88961
C	-1.99596	-4.71504	1.28948
O	-2.60441	-5.77207	1.14949
O	-0.73899	-4.5366	0.99548
C	-0.01104	-0.088	1.64854
O	0.03352	0.65259	2.571
N	-2.227	-1.18997	-0.01077
H	2.10484	-2.25074	1.12809
C	2.90533	-0.69408	0.16681
C	4.11876	1.04686	1.33682
C	4.26227	0.89205	-1.05742
H	4.38795	1.50153	2.28341
H	4.64194	1.22712	-2.01586
C	1.09913	-2.62149	-1.93323

H	1.2468	-1.66802	-2.45306
H	1.16901	-3.42609	-2.67646
C	2.11969	-2.83018	-0.83616
H	1.93562	-3.79866	-0.36067
H	3.13967	-2.84618	-1.23374
C	-2.79366	0.1321	-0.04107
C	-3.67587	2.03226	1.17576
C	-3.44588	2.14492	-1.21469
C	-3.81154	2.77964	-0.01201
H	-3.96975	2.45613	2.12918
H	-3.55254	2.66105	-2.16192
H	-2.56617	-1.64758	0.83133
H	-2.50342	-3.81152	1.69587
H	-0.47923	-3.38442	1.20647
C	3.41492	-0.21146	-1.03626
H	3.15824	-0.68978	-1.97736
C	4.62341	1.56368	0.12556
C	3.27412	-0.05384	1.35029
H	2.89616	-0.42899	2.29923
N	5.4263	2.70643	0.10037
C	6.10038	3.05739	1.33919
H	6.71787	3.93959	1.16695
H	5.38051	3.31243	2.12065
H	6.74663	2.25253	1.72279
C	6.22908	2.91103	-1.09334
H	5.59652	3.06686	-1.97073
H	6.83156	3.81073	-0.96364
H	6.90574	2.06929	-1.30876
C	-2.5484	-2.06802	-1.1745
H	-3.43308	-1.6987	-1.70231
H	-2.79867	-3.0557	-0.77356
C	-1.36486	-2.19772	-2.10773
H	-1.57859	-2.93695	-2.89055
H	-1.1278	-1.24616	-2.59696
C	-3.17317	0.73858	1.15606
H	-3.0752	0.18558	2.08838
C	-2.93984	0.84933	-1.22629
H	-2.65666	0.40604	-2.17755
N	-4.26475	4.10092	0.00716
C	-4.77561	4.63153	-1.24533
H	-5.13112	5.64892	-1.07909
H	-3.98838	4.68216	-2.0016
H	-5.60569	4.03815	-1.65957
C	-5.01457	4.51625	1.18073
H	-4.39081	4.48703	2.0775
H	-5.33821	5.54891	1.04534
H	-5.90543	3.89627	1.36682

1c-IN1

Energy: -1267.846138 Hartree

N	1.59662	1.55248	-1.44501
N	3.24766	-0.4584	-1.20683
Mn	1.30971	-0.28475	-1.13592
H	1.50052	1.70474	-2.41311
C	0.96097	-2.15828	-0.79031
O	0.7609	-3.23324	-0.59202
N	-0.49639	0.36059	-1.36823
C	0.59326	2.36264	-0.7365
C	-0.61824	1.65324	-0.66778
C	3.80347	-0.89598	0.09971
C	4.38461	-2.77319	1.40008
C	4.75528	-0.55785	2.24505
H	4.47559	-3.83943	1.48696
H	5.09701	0.09294	3.02139
C	2.947	1.90875	-0.96229
H	3.01566	1.82829	0.11418
H	3.21993	2.90833	-1.2299
C	3.80496	0.86217	-1.63098
H	3.68264	0.98613	-2.68267
H	4.84245	0.93078	-1.38187
N	3.878	-2.23035	0.28815
C	-1.57855	-0.59358	-0.8583

C	-2.301	-1.39094	-1.77124
C	-1.91522	-0.72807	0.52829
C	-3.25156	-2.35385	-1.33365
H	-2.13693	-1.25535	-2.8117
C	-2.8562	-1.71994	0.96512
H	-1.46414	-0.08586	1.25979
C	-3.47637	-2.58379	0.03588
H	-3.80009	-2.91604	-2.05827
H	-3.0909	-1.81901	2.00641
N	-4.353	-3.72557	0.48345
C	-5.39142	-4.01184	-0.5295
H	-4.92714	-4.33351	-1.43759
H	-6.04185	-4.78587	-0.16773
H	-5.96557	-3.12794	-0.71566
C	-5.00029	-3.40683	1.77028
H	-5.66697	-4.20254	2.04336
H	-4.25354	-3.29284	2.52719
H	-5.55632	-2.49725	1.67728
H	-0.62403	0.50259	-2.36063
C	1.31737	-0.23324	0.78159
O	1.30697	-0.14857	1.90516
H	3.52693	-1.1598	-1.86848
H	1.37342	-0.63313	-2.59104
C	4.27022	-0.04746	1.06895
H	4.26306	1.0103	0.9155
C	4.80022	-1.94503	2.43528
H	5.15723	-2.36483	3.35405
H	0.89369	2.50466	0.27678
H	0.50072	3.32259	-1.21675
H	-1.44318	2.20677	-1.09528
H	-0.8023	1.48488	0.36357

1c-IN2

Energy: -1456.421216 Hartree

H	1.5411	-1.62369	1.34062
N	1.51266	-1.82826	-1.3045
N	2.90714	0.21511	-0.06716
Mn	0.87738	-0.51819	0.22821
C	0.11894	0.64914	-0.85965
O	-0.42909	1.46744	-1.51898
H	2.00801	-2.62423	-0.90398
C	3.01174	-2.71048	1.59012
O	2.59268	-3.74388	1.18633
O	3.77915	-1.93648	2.0586
C	0.42884	0.43617	1.61745
O	0.12502	1.07813	2.56725
N	-0.82572	-1.85631	0.1985
H	3.37141	-0.07265	0.79179
C	2.9354	1.63764	-0.10742
C	3.12365	3.57116	1.09464
C	2.73919	3.72306	-1.25248
C	2.9278	4.36259	-0.02846
H	3.28703	4.02843	2.0682
H	2.58869	4.29701	-2.16194
H	2.92859	5.44382	0.05295
C	2.45155	-1.07605	-2.14378
H	1.88632	-0.28655	-2.65191
H	2.92051	-1.70368	-2.91283
C	3.5108	-0.50704	-1.22537
H	4.10166	-1.3281	-0.80652
H	4.20002	0.14584	-1.76961
N	3.1406	2.23023	1.07232
C	-2.08651	-1.1667	0.14191
C	-2.65196	-0.6934	1.32671
C	-2.75049	-0.91441	-1.05653
C	-3.83977	0.02351	1.31905
H	-2.15306	-0.89531	2.27251
C	-3.94214	-0.19596	-1.07193
H	-2.34848	-1.27413	-1.99959
C	-4.51078	0.30972	0.11216
H	-4.24639	0.35679	2.26703
H	-4.42811	-0.03275	-2.02709
N	-5.6743	1.08278	0.09203
C	-6.41342	1.18062	1.33953

H	-5.82553	1.69045	2.10676
H	-7.31209	1.77508	1.17109
H	-6.71736	0.20093	1.74017
C	-6.51295	0.96567	-1.08876
H	-7.39847	1.58816	-0.95589
H	-5.99215	1.32717	-1.97886
H	-6.84182	-0.06696	-1.28466
H	-0.76319	-2.31169	1.10396
C	2.74177	2.33719	-1.30157
H	2.58061	1.81086	-2.23694
C	0.29914	-2.33695	-1.95424
H	0.5187	-3.12197	-2.6895
H	-0.17354	-1.50111	-2.48236
C	-0.58521	-2.88133	-0.85464
H	-1.53247	-3.26003	-1.25219
H	-0.07286	-3.71858	-0.37053

1c-TS[2-3]

Energy: -1456.416327 Hartree

H	-1.42862	-0.60862	2.10635
N	-1.41413	1.8305	0.93549
N	-3.3771	-0.10553	0.38351
Mn	-1.21532	-0.24613	0.45674
C	-0.94665	0.04792	-1.2661
O	-0.74928	0.16995	-2.428
H	-1.52601	1.93408	1.94465
C	-2.24216	-0.00478	3.64715
O	-1.44522	0.7911	4.02018
O	-3.22748	-0.6635	3.63746
C	-0.98943	-1.95957	0.24157
O	-0.78617	-3.11963	0.11029
N	0.79046	0.31046	1.04322
H	-3.6565	-0.68261	1.1734
C	-3.89771	-0.71511	-0.79339
C	-4.63075	-2.64985	-1.76231
C	-4.52732	-0.67745	-3.09526
C	-4.81512	-2.03705	-2.99398
H	-4.856	-3.70641	-1.63401
H	-4.66362	-0.14928	-4.03417
H	-5.1782	-2.60538	-3.84298
C	-2.65155	2.28108	0.27668
H	-2.46112	2.29585	-0.80196
H	-2.94283	3.29416	0.58037
C	-3.75389	1.31248	0.64675
H	-3.9507	1.38735	1.72079
H	-4.68303	1.5664	0.12667
N	-4.18917	-2.0131	-0.66812
C	1.78065	-0.67315	0.6993
C	2.25933	-1.54092	1.67607
C	2.19756	-0.84899	-0.62078
C	3.12884	-2.575	1.34743
H	1.94442	-1.41206	2.70966
C	3.0749	-1.87027	-0.95371
H	1.83807	-0.17765	-1.39648
C	3.55041	-2.77621	0.0189
H	3.48102	-3.22424	2.14068
H	3.38603	-1.96354	-1.98788
N	4.38124	-3.84078	-0.3284
C	5.13969	-4.45744	0.74632
H	4.47585	-4.92184	1.47979
H	5.76574	-5.24668	0.32904
H	5.78896	-3.74586	1.27962
C	5.09939	-3.73114	-1.58704
H	5.73397	-4.60958	-1.7085
H	4.40921	-3.70903	-2.43392
H	5.73566	-2.83445	-1.64413
H	0.68165	0.32236	2.05725
C	-4.06045	0.00349	-1.98048
H	-3.81731	1.05946	-2.03531
C	-0.18459	2.46054	0.52221
H	-0.22665	3.32233	1.15502
H	-0.0811	2.77084	-0.49657
C	0.97065	1.67278	0.57581
H	1.29356	1.82048	-0.43356

H	1.73933	1.99471	1.24692

1c-IN3

Energy: -1456.436397 Hartree

H	1.5411	-1.62369	1.34062
N	1.51266	-1.82826	-1.3045
N	2.90714	0.21511	-0.06716
Mn	0.87738	-0.51819	0.22821
C	0.11894	0.64914	-0.85965
O	-0.42909	1.46744	-1.51898
H	2.00801	-2.62423	-0.90398
C	3.01174	-2.71048	1.59012
O	2.59268	-3.74388	1.18633
O	3.77915	-1.93648	2.0586
C	0.42884	0.43617	1.61745
O	0.12502	1.07813	2.56725
N	-0.82572	-1.85631	0.1985
H	3.37141	-0.07265	0.79179
C	2.9354	1.63764	-0.10742
C	3.12365	3.57116	1.09464
C	2.73919	3.72306	-1.25248
C	2.9278	4.36259	-0.02846
H	3.28703	4.02843	2.0682
H	2.58869	4.29701	-2.16194
H	2.92859	5.44382	0.05295
C	2.45155	-1.07605	-2.14378
H	1.88632	-0.28655	-2.65191
H	2.92051	-1.70368	-2.91283
C	3.5108	-0.50704	-1.22537
H	4.10166	-1.3281	-0.80652
H	4.20002	0.14584	-1.76961
N	3.1406	2.23023	1.07232
C	-2.08651	-1.1667	0.14191
C	-2.65196	-0.6934	1.32671
C	-2.75049	-0.91441	-1.05653
C	-3.83977	0.02351	1.31905
H	-2.15306	-0.89531	2.27251
C	-3.94214	-0.19596	-1.07193
H	-2.34848	-1.27413	-1.99959
C	-4.51078	0.30972	0.11216
H	-4.24639	0.35679	2.26703
H	-4.42811	-0.03275	-2.02709
N	-5.6743	1.08278	0.09203
C	-6.41342	1.18062	1.33953
H	-5.82553	1.69045	2.10676
H	-7.31209	1.77508	1.17109
H	-6.71736	0.20093	1.74017
C	-6.51295	0.96567	-1.08876
H	-7.39847	1.58816	-0.95589
H	-5.99215	1.32717	-1.97886
H	-6.84182	-0.06696	-1.28466
H	-0.76319	-2.31169	1.10396
C	2.74177	2.33719	-1.30157
H	2.58061	1.81086	-2.23694
C	0.29914	-2.33695	-1.95424
H	0.5187	-3.12197	-2.6895
H	-0.17354	-1.50111	-2.48236
C	-0.58521	-2.88133	-0.85464
H	-1.53247	-3.26003	-1.25219
H	-0.07286	-3.71858	-0.37053

1c-IN3R

Energy: -1456.459586 Hartree

N	-1.41413	1.8305	0.93549
N	-3.3771	-0.10553	0.38351
Mn	-1.21532	-0.24613	0.45674
C	-0.94665	0.04792	-1.2661
O	-0.74928	0.16995	-2.428
H	-1.52601	1.93408	1.94465
C	-2.05593	-1.43265	2.4569
O	-2.08963	-1.72917	3.6794
O	-1.70298	-0.23124	2.08585
C	-0.98943	-1.95957	0.24157
O	-0.78617	-3.11963	0.11029

N	0.79046	0.31046	1.04322
H	-3.6565	-0.68261	1.1734
C	-3.89771	-0.71511	-0.79339
C	-4.63075	-2.64985	-1.76231
C	-4.52732	-0.67745	-3.09526
C	-4.81512	-2.03705	-2.99398
H	-4.856	-3.70641	-1.63401
H	-4.66362	-0.14928	-4.03417
H	-5.1782	-2.60538	-3.84298
C	-2.65155	2.28108	0.27668
H	-2.46112	2.29585	-0.80196
H	-2.94283	3.29416	0.58037
C	-3.75389	1.31248	0.64675
H	-3.9507	1.38735	1.72079
H	-4.68303	1.5664	0.12667
N	-4.18917	-2.0131	-0.66812
C	1.78065	-0.67315	0.6993
C	2.25933	-1.54092	1.67607
C	2.19756	-0.84899	-0.62078
C	3.12884	-2.575	1.34743
H	1.94442	-1.41206	2.70966
C	3.0749	-1.87027	-0.95371
H	1.83807	-0.17765	-1.39648
C	3.55041	-2.77621	0.0189
H	3.48102	-3.22424	2.14068
H	3.38603	-1.96354	-1.98788
N	4.38124	-3.84078	-0.3284
C	5.13969	-4.45744	0.74632
H	4.47585	-4.92184	1.47979
H	5.76574	-5.24668	0.32904
H	5.78896	-3.74586	1.27962
C	5.09939	-3.73114	-1.58704
H	5.73397	-4.60958	-1.7085
H	4.40921	-3.70903	-2.43392
H	5.73566	-2.83445	-1.64413
H	0.68165	0.32236	2.05725
C	-4.06045	0.00349	-1.98048
H	-3.81731	1.05946	-2.03531
C	-0.18459	2.46054	0.52221
H	-0.22665	3.32233	1.15502
H	-0.0811	2.77084	-0.49657
C	0.97065	1.67278	0.57581
H	1.29356	1.82048	-0.43356
H	1.73933	1.99471	1.24692
H	-2.31645	-2.16484	1.72141

1c-IN4

Energy: -1457.597488 Hartree

H	0.91033	-1.77154	1.29618
N	1.68403	-2.1236	-1.16688
N	3.09899	-0.63966	0.75548
Mn	1.04507	-0.54071	0.05437
C	1.34891	0.71313	-1.13555
O	1.55899	1.59919	-1.89125
H	1.46639	-2.98473	-0.66894
C	-1.72559	-2.00952	2.45084
O	-2.81618	-2.30263	2.93922
O	-0.96084	-2.82927	1.79124
C	0.41116	0.66979	1.15135
O	-0.03409	1.50067	1.86563
N	-0.74714	-1.23389	-0.8476
H	2.9717	-0.84079	1.74352
C	3.76793	0.61569	0.66149
C	4.11585	2.64921	1.64421
C	5.11105	2.22677	-0.47799
H	3.93162	3.29105	2.50301
H	5.71965	2.52542	-1.32622
C	3.1462	-2.03104	-1.29516
H	3.35878	-1.18989	-1.96372
H	3.58019	-2.93263	-1.74486
C	3.72591	-1.80873	0.08317
H	3.50957	-2.6775	0.71126
H	4.81413	-1.69485	0.03778
C	-1.97719	-0.4991	-0.66568

C	-3.12511	-1.14496	-0.21432
C	-2.04427	0.86476	-0.94951
C	-4.30825	-0.44414	-0.00997
H	-3.09623	-2.21404	-0.01524
C	-3.22496	1.56937	-0.76826
H	-1.16199	1.38387	-1.31658
C	-4.38552	0.93797	-0.2693
H	-5.17486	-0.98701	0.34957
H	-3.23727	2.62661	-1.00732
N	-5.54964	1.65965	-0.01968
C	-6.78157	0.89963	0.10452
H	-6.74521	0.23073	0.96807
H	-7.60896	1.59176	0.26313
H	-7.0046	0.29196	-0.78591
C	-5.6912	2.94047	-0.69121
H	-6.65696	3.37216	-0.42689
H	-4.92076	3.64326	-0.36507
H	-5.63606	2.86081	-1.7877
H	-0.89519	-2.15154	-0.42459
H	-1.31424	-0.97953	2.55412
H	0.06503	-2.22356	1.48377
C	4.54851	0.958	-0.44465
H	4.70866	0.26057	-1.25948
N	3.5631	1.43013	1.70121
C	4.88814	3.10107	0.58226
H	5.30705	4.10104	0.59212
C	-0.42491	-1.45098	-2.28519
H	-0.42506	-0.46485	-2.75667
H	-1.21776	-2.04231	-2.75853
C	0.94385	-2.12237	-2.44651
H	0.8436	-3.14482	-2.82373
H	1.53947	-1.56411	-3.17396

1c-TS[4-5]

Energy: -1457.580683 Hartree

H	-1.48511	-1.1479	2.18389
N	-1.68555	1.40208	1.57457
N	-3.50189	-0.60287	0.78434
Mn	-1.35647	-0.45226	0.58685
C	-1.4022	0.26219	-1.02073
O	-1.44235	0.70448	-2.11521
H	-1.60357	1.246	2.57965
C	0.76136	-2.77949	2.95683
O	1.72139	-3.37812	3.43979
O	0.27132	-1.66463	3.41485
C	-1.01448	-2.01591	-0.12541
O	-0.75995	-3.05394	-0.62921
N	0.65272	0.24162	1.00717
H	-3.60208	-1.42341	1.37576
C	-4.15046	-0.89716	-0.45181
C	-4.79592	-2.53726	-1.90483
C	-5.19301	-0.26538	-2.50288
H	-4.8573	-3.60412	-2.10826
H	-5.56696	0.4934	-3.18359
C	-3.07104	1.79896	1.27313
H	-3.09939	2.11615	0.22517
H	-3.40557	2.6429	1.88781
C	-3.95934	0.60222	1.52964
H	-3.91319	0.34268	2.59096
H	-5.00231	0.83274	1.28974
C	1.75054	-0.45576	0.3821
C	2.7328	-1.05948	1.16146
C	1.84096	-0.55161	-1.00681
C	3.76505	-1.78401	0.57622
H	2.69669	-0.96021	2.24358
C	2.87853	-1.25373	-1.60025
H	1.09858	-0.06335	-1.63283
C	3.8565	-1.91406	-0.82313
H	4.50479	-2.24118	1.22323
H	2.91971	-1.29573	-2.68258
N	4.8541	-2.6792	-1.4186
C	6.01719	-2.99599	-0.60808
H	5.73994	-3.60852	0.25356
H	6.71836	-3.5767	-1.20794

H	6.53858	-2.10143	-0.23443
C	5.13796	-2.42441	-2.82085
H	5.96072	-3.06649	-3.13641
H	4.27665	-2.66772	-3.44781
H	5.41884	-1.37908	-3.02017
H	0.71616	0.06357	2.01201
H	0.23496	-3.17782	2.06009
H	-0.68787	-1.35935	2.7048
C	-4.62099	0.11082	-1.2954
H	-4.53586	1.15768	-1.02404
N	-4.24364	-2.20019	-0.73124
C	-5.28054	-1.61727	-2.82497
H	-5.71799	-1.95138	-3.75908
C	0.58472	1.68112	0.80671
H	0.69904	2.02066	-0.20153
H	1.41092	2.02341	1.39418
C	-0.63755	2.28384	1.12262
H	-0.63156	2.98332	1.93231
H	-0.78698	2.80642	0.20095

1c-IN5

Energy: -1457.583172 Hartree

H	0.91033	-1.77154	1.29618
N	1.68403	-2.1236	-1.16688
N	3.09899	-0.63966	0.75548
Mn	1.04507	-0.54071	0.05437
C	1.34891	0.71313	-1.13555
O	1.55899	1.59919	-1.89125
H	1.46639	-2.98473	-0.66894
C	-1.72559	-2.00952	2.45084
O	-2.81618	-2.30263	2.93922
O	-0.96084	-2.82927	1.79124
C	0.41116	0.66979	1.15135
O	-0.03409	1.50067	1.86563
N	-0.74714	-1.23389	-0.8476
H	2.9717	-0.84079	1.74352
C	3.76793	0.61569	0.66149
C	4.11585	2.64921	1.64421
C	5.11105	2.22677	-0.47799
H	3.93162	3.29105	2.50301
H	5.71965	2.52542	-1.32622
C	3.1462	-2.03104	-1.29516
H	3.35878	-1.18989	-1.96372
H	3.58019	-2.93263	-1.74486
C	3.72591	-1.80873	0.08317
H	3.50957	-2.6775	0.71126
H	4.81413	-1.69485	0.03778
C	-1.97719	-0.4991	-0.66568
C	-3.12511	-1.14496	-0.21432
C	-2.04427	0.86476	-0.94951
C	-4.30825	-0.44414	-0.00997
H	-3.09623	-2.21404	-0.01524
C	-3.22496	1.56937	-0.76826
H	-1.16199	1.38387	-1.31658
C	-4.38552	0.93797	-0.2693
H	-5.17486	-0.98701	0.34957
H	-3.23727	2.62661	-1.00732
N	-5.54964	1.65965	-0.01968
C	-6.78157	0.89963	0.10452
H	-6.74521	0.23073	0.96807
H	-7.60896	1.59176	0.26313
H	-7.0046	0.29196	-0.78591
C	-5.6912	2.94047	-0.69121
H	-6.65696	3.37216	-0.42689
H	-4.92076	3.64326	-0.36507
H	-5.63606	2.86081	-1.7877
H	-0.89519	-2.15154	-0.42459
H	-1.31424	-0.97953	2.55412
H	0.06503	-2.22356	1.48377
C	4.54851	0.958	-0.44465
H	4.70866	0.26057	-1.25948
N	3.5631	1.43013	1.70121
C	4.88814	3.10107	0.58226
H	5.30705	4.10104	0.59212

C	-0.42491	-1.45098	-2.28519
H	-0.42506	-0.46485	-2.75667
H	-1.21776	-2.04231	-2.75853
C	0.94385	-2.12237	-2.44651
H	0.8436	-3.14482	-2.82373
H	1.53947	-1.56411	-3.17396

1d-IN1

Energy: -1283.905517 Hartree

N	1.4462	1.85658	-1.33613
N	3.48846	-0.07388	-1.06137
Mn	1.32331	-0.24226	-0.93808
H	1.43579	1.99027	-2.34616
C	1.13005	-1.96523	-0.79366
O	0.94371	-3.13381	-0.70208
N	-0.75033	0.31938	-1.22828
C	4.13928	-0.66092	0.05961
C	4.97071	-2.58056	0.97994
C	5.04466	-0.578	2.26775
H	5.17183	-3.64191	0.85039
H	5.29827	-0.02904	3.1697
C	2.75264	2.29518	-0.82292
H	2.70074	2.2687	0.27115
H	2.99948	3.3211	-1.12325
C	3.80017	1.34804	-1.36696
H	3.82286	1.43143	-2.45725
H	4.79494	1.61534	-0.99517
C	-1.67977	-0.68658	-0.79423
C	-3.08581	-2.58248	-1.33005
C	-2.744	-1.95425	0.96761
C	-3.33259	-2.83303	0.03334
H	-3.53169	-3.20937	-2.09354
H	-2.91928	-2.0861	2.0292
H	-0.77811	0.37214	-2.24527
C	1.25438	-0.0341	0.82192
O	1.20086	0.03022	2.00478
H	3.69471	-0.65129	-1.87065
H	1.38712	-0.4504	-2.6148
C	4.45286	0.08225	1.20061
H	4.23294	1.14301	1.25801
C	5.30714	-1.94228	2.16592
C	4.40343	-1.96518	-0.06702
H	4.17078	-2.49388	-0.96772
N	5.92943	-2.69144	3.26701
C	6.57357	-3.9007	2.73438
H	7.17523	-4.34962	3.49686
H	5.82311	-4.59448	2.41756
H	7.19124	-3.63819	1.90103
C	6.93802	-1.84828	3.92483
H	6.4491	-1.08125	4.48833
H	7.53219	-2.44923	4.58112
H	7.5669	-1.40063	3.18388
H	-3.94087	-3.65521	0.34785
N	-2.27397	-1.52729	-1.73083
N	-1.92426	-0.91142	0.56104
C	0.27397	2.45742	-0.75255
H	0.26955	3.34007	-1.35738
H	0.25779	2.7333	0.28115
C	-0.87342	1.66001	-0.68659
H	-1.06942	1.7454	0.36183
H	-1.71732	2.01928	-1.23763

1d-IN2

Energy: -1472.480187 Hartree

H	-1.55448	-2.1547	0.62351
N	-1.65366	-1.56025	-1.85968
N	0.76399	-1.7636	-0.53092
Mn	-1.02133	-0.67391	-0.05056
C	-0.46991	0.83164	-0.79819
O	-0.07025	1.85876	-1.23026
H	-2.01286	-2.49241	-1.66238
C	-1.12435	-2.83294	2.26607
O	-2.08188	-2.53011	2.89817
O	-0.06828	-3.33367	2.04864
C	-0.52366	-0.07827	1.51539
O	-0.17045	0.3202	2.57443
N	-3.14832	-0.12232	-0.01628
H	0.75997	-2.47813	0.19397
C	1.96705	-0.99176	-0.38229
C	3.72228	-0.15877	1.06513
C	3.65348	0.51528	-1.24205
C	4.27813	0.60298	0.01695

H	4.17759	-0.15511	2.04902
H	4.05297	1.0583	-2.09101
C	-0.45477	-1.70215	-2.6922
H	-0.11382	-0.6972	-2.9662
H	-0.65211	-2.25851	-3.6179
C	0.56787	-2.43324	-1.84976
H	0.19763	-3.44188	-1.64004
H	1.52168	-2.53494	-2.37646
C	-3.21962	1.26518	0.23171
C	-3.14253	2.92985	1.75627
C	-3.22246	3.38043	-0.55586
C	-3.12432	3.87399	0.7364
H	-3.12221	3.23298	2.8008
H	-3.26398	4.05177	-1.41119
H	-3.49619	-0.62267	0.79646
C	2.51602	-0.26168	-1.4342
H	2.05964	-0.28913	-2.4203
H	-3.06661	4.93567	0.94131
C	2.58847	-0.93413	0.86535
H	2.17774	-1.50946	1.69295
N	5.38551	1.42854	0.22565
N	-3.28368	2.07121	-0.83029
N	-3.19919	1.61514	1.52475
C	6.21297	1.12828	1.38191
H	7.05813	1.8173	1.39956
H	5.65727	1.27219	2.31165
H	6.60559	0.09948	1.37722
C	6.13275	1.82573	-0.95541
H	5.52012	2.43995	-1.6199
H	6.98488	2.43238	-0.64703
H	6.50966	0.96975	-1.53684
C	-3.77373	-0.62567	-1.26742
H	-4.60791	0.0158	-1.56207
H	-4.173	-1.61604	-1.03106
C	-2.75631	-0.74657	-2.38017
H	-3.22108	-1.19291	-3.26898
H	-2.35515	0.23363	-2.65738

1d-TS2-3

Energy: -1472.473910 Hartree

H	-1.42862	-0.60862	2.10635
N	-1.41413	1.8305	0.93549
N	-3.3771	-0.10553	0.38351
Mn	-1.21532	-0.24613	0.45674
C	-0.94665	0.04792	-1.2661
O	-0.74928	0.16995	-2.428
H	-1.52601	1.93408	1.94465
C	-2.24216	-0.00478	3.64715
O	-1.44522	0.7911	4.02018
O	-3.22748	-0.6635	3.63746
C	-0.98943	-1.95957	0.24157
O	-0.78617	-3.11963	0.11029
N	0.79046	0.31046	1.04322
H	-3.6565	-0.68261	1.1734
C	-3.89771	-0.71511	-0.79339
C	-4.63075	-2.64985	-1.76231
C	-4.52732	-0.67745	-3.09526
C	-4.81512	-2.03705	-2.99398
H	-4.856	-3.70641	-1.63401
H	-4.66362	-0.14928	-4.03417
C	-2.65155	2.28108	0.27668
H	-2.46112	2.29585	-0.80196
H	-2.94283	3.29416	0.58037
C	-3.75389	1.31248	0.64675
H	-3.9507	1.38735	1.72079
H	-4.68303	1.5664	0.12667
C	1.78065	-0.67315	0.6993
C	3.12884	-2.575	1.34743
C	3.0749	-1.87027	-0.95371
C	3.55041	-2.77621	0.0189
H	3.48102	-3.22424	2.14068
H	3.38603	-1.96354	-1.98788
H	0.68165	0.32236	2.05725
C	-4.06045	0.00349	-1.98048
H	-3.81731	1.05946	-2.03531
H	4.2039	-3.58216	-0.24242
C	-4.18917	-2.0131	-0.66812
H	-4.07404	-2.52017	0.26704
N	-5.30737	-2.80757	-4.14501
N	2.19756	-0.84899	-0.62078
N	2.25933	-1.54092	1.67607
C	-5.64964	-4.17011	-3.71231
H	-6.05905	-4.71418	-4.5377
H	-4.76825	-4.66477	-3.36107

H	-6.37075	-4.12317	-2.9232
C	-6.50274	-2.15398	-4.6971
H	-6.21604	-1.25781	-5.2066
H	-6.98585	-2.81625	-5.38479
H	-7.17645	-1.91213	-3.90178
C	0.97065	1.67278	0.57581
H	1.28327	1.84486	-0.43294
H	1.74963	1.97033	1.2463
C	-0.18459	2.46054	0.52221
H	-0.20053	3.32166	1.15713
H	-0.10722	2.77151	-0.49868

1d-IN3

Energy: -1472.493722 Hartree

H	-1.55448	-2.1547	0.62351
N	-1.65366	-1.56025	-1.85968
N	0.76399	-1.7636	-0.53092
Mn	-1.02133	-0.67391	-0.05056
C	-0.46991	0.83164	-0.79819
O	-0.07025	1.85876	-1.23026
H	-2.01286	-2.49241	-1.66238
C	-1.12435	-2.83294	2.26607
O	-2.08188	-2.53011	2.89817
O	-0.06828	-3.33367	2.04864
C	-0.52366	-0.07827	1.51539
O	-0.17045	0.3202	2.57443
N	-3.14832	-0.12232	-0.01628
H	0.75997	-2.47813	0.19397
C	1.96705	-0.99176	-0.38229
C	3.72228	-0.15877	1.06513
C	3.65348	0.51528	-1.24205
C	4.27813	0.60298	0.01695
H	4.17759	-0.15511	2.04902
H	4.05297	1.0583	-2.09101
C	-0.45477	-1.70215	-2.6922
H	-0.11382	-0.6972	-2.9662
H	-0.65211	-2.25851	-3.6179
C	0.56787	-2.43324	-1.84976
H	0.19763	-3.44188	-1.64004
H	1.52168	-2.53494	-2.37646
C	-3.21962	1.26518	0.23171
C	-3.14253	2.92985	1.75627
C	-3.22246	3.38043	-0.55586
C	-3.12432	3.87399	0.7364
H	-3.12221	3.23298	2.8008
H	-3.26398	4.05177	-1.41119
H	-3.49619	-0.62267	0.79646
C	2.51602	-0.26168	-1.4342
H	2.05964	-0.28913	-2.4203
H	-3.06661	4.93567	0.94131
C	2.58847	-0.93413	0.86535
H	2.17774	-1.50946	1.69295
N	5.38551	1.42854	0.22565
N	-3.28368	2.07121	-0.83029
N	-3.19919	1.61514	1.52475
C	6.21297	1.12828	1.38191
H	7.05813	1.8173	1.39956
H	5.65727	1.27219	2.31165
H	6.60559	0.09948	1.37722
C	6.13275	1.82573	-0.95541
H	5.52012	2.43995	-1.6199
H	6.98488	2.43238	-0.64703
H	6.50966	0.96975	-1.53684
C	-3.77373	-0.62567	-1.26742
H	-4.60791	0.0158	-1.56207
H	-4.173	-1.61604	-1.03106
C	-2.75631	-0.74657	-2.38017
H	-3.22108	-1.19291	-3.26898
H	-2.35515	0.23363	-2.65738

1d-IN3R

Energy: -1472.517756 Hartree

N	-1.41413	1.8305	0.93549
N	-3.3771	-0.10553	0.38351
Mn	-1.21532	-0.24613	0.45674
C	-0.94665	0.04792	-1.2661
O	-0.74928	0.16995	-2.428
H	-1.52601	1.93408	1.94465
C	-2.24372	-1.37719	2.39913
O	-2.33304	-1.73088	3.6035
O	-1.70585	-0.23116	2.09543

C	-0.98943	-1.95957	0.24157
O	-0.78617	-3.11963	0.11029
N	0.79046	0.31046	1.04322
H	-3.6565	-0.68261	1.1734
C	-3.89771	-0.71511	-0.79339
C	-4.63075	-2.64985	-1.76231
C	-4.52732	-0.67745	-3.09526
C	-4.81512	-2.03705	-2.99398
H	-4.856	-3.70641	-1.63401
H	-4.66362	-0.14928	-4.03417
C	-2.65155	2.28108	0.27668
H	-2.46112	2.29585	-0.80196
H	-2.94283	3.29416	0.58037
C	-3.75389	1.31248	0.64675
H	-3.9507	1.38735	1.72079
H	-4.68303	1.5664	0.12667
C	1.78065	-0.67315	0.6993
C	3.12884	-2.575	1.34743
C	3.0749	-1.87027	-0.95371
C	3.55041	-2.77621	0.0189
H	3.48102	-3.22424	2.14068
H	3.38603	-1.96354	-1.98788
H	0.68165	0.32236	2.05725
C	-4.06045	0.00349	-1.98048
H	-3.81731	1.05946	-2.03531
H	4.2039	-3.58216	-0.24242
C	-4.18917	-2.0131	-0.66812
H	-4.07404	-2.52017	0.26704
N	-5.30737	-2.80757	-4.14501
N	2.19756	-0.84899	-0.62078
N	2.25933	-1.54092	1.67607
C	-5.64964	-4.17011	-3.71231
H	-6.05905	-4.71418	-4.5377
H	-4.76825	-4.66477	-3.36107
H	-6.37075	-4.12317	-2.9232
C	-6.50274	-2.15398	-4.6971
H	-6.21604	-1.25781	-5.2066
H	-6.98585	-2.81625	-5.38479
H	-7.17645	-1.91213	-3.90178
C	0.97065	1.67278	0.57581
H	1.28327	1.84486	-0.43294
H	1.74963	1.97033	1.2463
C	-0.18459	2.46054	0.52221
H	-0.20053	3.32166	1.15713
H	-0.10722	2.77151	-0.49868
H	-2.60984	-2.01835	1.62468

1d-IN4

Energy: -1473.648705 Hartree

H	1.63001	1.41203	1.43116
N	1.64709	1.50038	-1.31843
N	-0.6669	1.86609	0.17306
Mn	0.86985	0.36297	0.26974
C	-0.01623	-0.76496	-0.75362
O	-0.65667	-1.5533	-1.35714
H	2.24107	2.24245	-0.94522
C	4.24617	2.51414	1.37801
O	5.32569	3.09135	1.27633
O	3.11529	2.95978	0.91195
C	0.28187	-0.4943	1.68077
O	-0.12631	-1.07455	2.62579
N	2.81485	-0.61883	-0.00012
H	-0.5654	2.35151	1.06001
C	-1.99989	1.32333	0.12709
C	-3.88848	0.39435	1.32746
C	-3.93988	0.51671	-1.07288
H	-4.34179	0.1531	2.28216
H	-4.43229	0.36758	-2.027
C	0.5042	2.12701	-1.99282
H	-0.06712	1.33682	-2.49288
H	0.81935	2.85066	-2.75521
C	-0.30555	2.81627	-0.91762
H	0.30516	3.60199	-0.46212
H	-1.2012	3.29147	-1.33003
C	2.57442	-2.01109	-0.03744
C	2.1953	-3.90545	1.13134
C	2.03456	-3.8554	-1.2199
C	1.89673	-4.58073	-0.04601
H	2.16422	-4.41384	2.09247
H	1.86797	-4.32003	-2.18955
H	3.31358	-0.39625	0.85704
H	4.16993	1.53346	1.90024
H	2.2746	2.115	1.14557
C	-2.6749	1.09582	-1.07001

H	-2.22477	1.36642	-2.0211
C	-4.57458	0.13239	0.12344
H	1.60179	-5.62254	-0.04821
C	-2.62708	0.972	1.32261
H	-2.12005	1.16101	2.2667
N	2.54802	-2.61669	1.15648
N	2.38546	-2.56302	-1.23722
N	-5.81848	-0.50087	0.11834
C	-6.57726	-0.46213	1.35711
H	-7.53388	-0.96178	1.20005
H	-6.05738	-0.99916	2.15417
H	-6.77554	0.56162	1.71073
C	-6.62346	-0.34682	-1.08144
H	-6.13802	-0.81167	-1.94317
H	-7.5777	-0.85356	-0.93372
H	-6.82592	0.70582	-1.33338
C	3.49398	-0.01526	-1.1793
H	4.10593	-0.76268	-1.68936
H	4.16275	0.75744	-0.78845
C	2.49306	0.61104	-2.12317
H	3.01515	1.14983	-2.92425
H	1.85132	-0.14813	-2.58203

1d-TS4-5

Energy: -1473.642427 Hartree

H	-1.48511	-1.1479	2.18389
N	-1.68555	1.40208	1.57457
N	-3.50189	-0.60287	0.78434
Mn	-1.35647	-0.45226	0.58685
C	-1.4022	0.26219	-1.02073
O	-1.44235	0.70448	-2.11521
H	-1.60357	1.246	2.57965
C	0.76136	-2.77949	2.95683
O	1.72139	-3.37812	3.43979
O	0.27132	-1.66463	3.41485
C	-1.01448	-2.01591	-0.12541
O	-0.75995	-3.05394	-0.62921
N	0.65272	0.24162	1.00717
H	-3.60208	-1.42341	1.37576
C	-4.15046	-0.89716	-0.45181
C	-4.79592	-2.53726	-1.90483
C	-5.19301	-0.26538	-2.50288
H	-4.8573	-3.60412	-2.10826
H	-5.56696	0.4934	-3.18359
C	-3.07104	1.79896	1.27313
H	-3.09939	2.11615	0.22517
H	-3.40557	2.6429	1.88781
C	-3.95934	0.60222	1.52964
H	-3.91319	0.34268	2.59096
H	-5.00231	0.83274	1.28974
C	1.75054	-0.45576	0.3821
C	3.72954	-1.75909	0.59635
C	2.84578	-1.23157	-1.58152
C	3.8565	-1.91406	-0.82313
H	4.46928	-2.21626	1.24335
H	2.88696	-1.27357	-2.66385
H	0.71616	0.06357	2.01201
H	0.23496	-3.17782	2.06009
H	-0.68787	-1.35935	2.7048
C	-4.62099	0.11082	-1.2954
H	-4.53586	1.15768	-1.02404
C	-5.28054	-1.61727	-2.82497
H	4.63485	-2.48945	-1.27918
C	-4.24364	-2.20019	-0.73124
H	-3.89127	-2.94161	-0.04493
N	2.6981	-1.03816	1.13393
N	1.83755	-0.54799	-0.9544
N	-5.87363	-2.07026	-4.09143
C	-5.96929	-3.53714	-4.09071
H	-6.45627	-3.86325	-4.98592
H	-4.98684	-3.95861	-4.0453
H	-6.53439	-3.8573	-3.24038
C	-7.21701	-1.49168	-4.23801
H	-7.13372	-0.44806	-4.45894
H	-7.73277	-1.98497	-5.03523
H	-7.76239	-1.62088	-3.32654
C	0.58472	1.68112	0.80671
H	0.7037	2.01299	-0.20354
H	1.40627	2.03107	1.39619
C	-0.63755	2.28384	1.12262
H	-0.65801	2.98042	1.93457
H	-0.76054	2.80931	0.19869

1d-IN5

Energy: -1473.641739 Hartree

H	1.63001	1.41203	1.43116
N	1.64709	1.50038	-1.31843
N	-0.6669	1.86609	0.17306
Mn	0.86985	0.36297	0.26974
C	-0.01623	-0.76496	-0.75362
O	-0.65667	-1.5533	-1.35714
H	2.24107	2.24245	-0.94522
C	4.24617	2.51414	1.37801
O	5.32569	3.09135	1.27633
O	3.11529	2.95978	0.91195
C	0.28187	-0.4943	1.68077
O	-0.12631	-1.07455	2.62579
N	2.81485	-0.61883	-0.00012
H	-0.5654	2.35151	1.06001
C	-1.99989	1.32333	0.12709
C	-3.88848	0.39435	1.32746
C	-3.93988	0.51671	-1.07288
H	-4.34179	0.1531	2.28216
H	-4.43229	0.36758	-2.027
C	0.5042	2.12701	-1.99282
H	-0.06712	1.33682	-2.49288
H	0.81935	2.85066	-2.75521
C	-0.30555	2.81627	-0.91762
H	0.30516	3.60199	-0.46212
H	-1.2012	3.29147	-1.33003
C	2.57442	-2.01109	-0.03744
C	2.1953	-3.90545	1.13134
C	2.03456	-3.8554	-1.2199
C	1.89673	-4.58073	-0.04601
H	2.16422	-4.41384	2.09247
H	1.86797	-4.32003	-2.18955
H	3.31358	-0.39625	0.85704
H	4.16993	1.53346	1.90024
H	2.2746	2.115	1.14557
C	-2.6749	1.09582	-1.07001
H	-2.22477	1.36642	-2.0211
C	-4.57458	0.13239	0.12344
H	1.60179	-5.62254	-0.04821
C	-2.62708	0.972	1.32261
H	-2.12005	1.16101	2.2667
N	2.54802	-2.61669	1.15648
N	2.38546	-2.56302	-1.23722
N	-5.81848	-0.50087	0.11834
C	-6.57726	-0.46213	1.35711
H	-7.53388	-0.96178	1.20005
H	-6.05738	-0.99916	2.15417
H	-6.77554	0.56162	1.71073
C	-6.62346	-0.34682	-1.08144
H	-6.13802	-0.81167	-1.94317
H	-7.5777	-0.85356	-0.93372
H	-6.82592	0.70582	-1.33338
C	3.49398	-0.01526	-1.1793
H	4.10593	-0.76268	-1.68936
H	4.16275	0.75744	-0.78845
C	2.49306	0.61104	-2.12317
H	3.01515	1.14983	-2.92425
H	1.85132	-0.14813	-2.58203

1e-IN1

Energy: -1182.134415 Hartree

N	-1.76787	2.43135	0.37723
N	0.71604	1.94979	-0.76524
Mn	-1.10567	0.78022	-0.76612
H	-2.09855	3.15982	-0.25201
C	-0.5975	-0.45688	-1.8833
O	-0.24269	-1.28993	-2.651
N	-3.23892	0.2577	-0.54937
C	1.88324	1.18142	-0.42955
C	3.64876	-0.31628	-1.14745
C	3.45712	0.27932	1.17317
H	4.14137	-0.83012	-1.9652
H	3.7946	0.23471	2.2024
C	-0.59402	2.94283	1.09016
H	-0.2933	2.18686	1.82464
H	-0.80556	3.87567	1.62981
C	0.47855	3.17468	0.04897
H	0.13726	3.95276	-0.64081
H	1.41044	3.52424	0.50483
C	-3.30532	-1.05794	-0.04565
C	-3.17331	-3.2704	-0.48386
C	-3.35811	-2.46249	1.7218
C	-3.20708	-3.55152	0.87699
H	-3.1095	-4.06986	-1.21906

H	-3.44266	-2.59546	2.79854
H	-3.56742	0.2638	-1.50928
C	-0.59038	-0.21047	0.61282
O	-0.21445	-0.95254	1.45818
H	0.79699	2.22691	-1.73818
H	-1.57463	1.68628	-2.10994
C	4.12362	-0.45367	0.17307
H	-3.14848	-4.56517	1.25322
N	-3.2306	-2.02704	-0.9686
N	-3.42145	-1.20112	1.27678
C	-2.9	1.98007	1.19029
H	-3.38183	2.80594	1.73021
H	-2.52414	1.26089	1.92523
C	-3.88776	1.32636	0.25073
H	-4.74795	0.92891	0.79584
H	-4.25271	2.06667	-0.46696
H	4.95414	-1.08725	0.40482
N	2.35544	1.07533	0.87698
N	2.54972	0.48096	-1.43549

1e-IN2

Energy: -1370.709816 Hartree

H	0.1182	2.25413	-0.24796
N	-0.02826	1.29006	2.09878
N	2.12595	0.69951	0.46547
Mn	-0.01933	0.594	0.10786
C	-0.15819	-1.13032	0.50224
O	-0.24201	-2.29797	0.66922
H	0.04557	2.30557	2.08356
C	0.7413	3.06193	-1.74379
O	-0.27309	3.37074	-2.27659
O	1.9181	2.98524	-1.59014
C	-0.00092	0.2092	-1.59999
O	0.01341	-0.04063	-2.75646
N	-2.15246	1.01334	0.34296
H	2.40805	1.42134	-0.19509
C	1.17417	0.7587	2.7475
H	1.07111	-0.32899	2.81838
H	1.31331	1.15986	3.75978
C	2.34333	1.1412	1.86836
H	2.43159	2.23129	1.83338
H	3.28155	0.73659	2.25522
C	-2.86934	-0.13041	-0.07667
C	-3.70161	-1.3252	-1.80272
C	-3.75992	-2.14703	0.40552
C	-4.02072	-2.36568	-0.93882
H	-3.91906	-1.39311	-2.86638
H	-4.02092	-2.89022	1.15603
H	-2.30928	1.76285	-0.32497
H	-4.47331	-3.2836	-1.2924
N	-3.19094	-1.02363	0.86079
N	-3.13092	-0.18982	-1.38842
C	-2.38143	1.48194	1.73627
H	-3.38738	1.2143	2.06813
H	-2.3118	2.57294	1.70591
C	-1.32891	0.93725	2.67521
H	-1.46649	1.35662	3.68033
H	-1.3863	-0.15369	2.74604
C	2.70052	-0.53231	0.08115
N	3.01322	-0.63427	-1.21729
N	2.85434	-1.45595	1.03196
C	3.44418	-1.83834	-1.60477
C	3.28731	-2.6484	0.60297
C	3.57871	-2.90786	-0.72768
H	3.70034	-1.94097	-2.65702
H	3.41021	-3.41597	1.3643
H	3.91907	-3.8805	-1.06023

1e-TS2-3

Energy: -1370.701613 Hartree

H	-1.55448	-2.1547	0.62351
N	-1.65366	-1.56025	-1.85968
N	0.76399	-1.7636	-0.53092
Mn	-1.02133	-0.67391	-0.05056
C	-0.46991	0.83164	-0.79819
O	-0.07025	1.85876	-1.23026
H	-2.01286	-2.49241	-1.66238
C	-1.12435	-2.83294	2.26607
O	-2.08188	-2.53011	2.89817
O	-0.06828	-3.33367	2.04864
C	-0.52366	-0.07827	1.51539
O	-0.17045	0.3202	2.57443
N	-3.14832	-0.12232	-0.01628
H	0.75997	-2.47813	0.19397

C	-0.45477	-1.70215	-2.6922
H	-0.11382	-0.6972	-2.9662
H	-0.65211	-2.25851	-3.6179
C	0.56787	-2.43324	-1.84976
H	0.19763	-3.44188	-1.64004
H	1.52168	-2.53494	-2.37646
C	-3.21962	1.26518	0.23171
C	-3.14253	2.92985	1.75627
C	-3.22246	3.38043	-0.55586
C	-3.12432	3.87399	0.7364
H	-3.12221	3.23298	2.8008
H	-3.26398	4.05177	-1.41119
H	-3.49619	-0.62267	0.79646
H	-3.06661	4.93567	0.94131
N	-3.28368	2.07121	-0.83029
N	-3.19919	1.61514	1.52475
C	-3.77373	-0.62567	-1.26742
H	-4.60791	0.0158	-1.56207
H	-4.173	-1.61604	-1.03106
C	-2.75631	-0.74657	-2.38017
H	-3.22108	-1.19291	-3.26898
H	-2.35515	0.23363	-2.65738
C	1.99462	-0.97408	-0.37889
N	2.50856	-0.88372	0.87821
N	2.50003	-0.41045	-1.51008
C	3.63818	-0.1556	1.00057
C	3.6298	0.31079	-1.35328
C	4.25139	0.47369	-0.10008
H	4.06217	-0.07705	2.01757
H	4.04689	0.7718	-2.26638
H	5.16821	1.06185	0.01322

1e-IN3

Energy: -1370.720911 Hartree

H	0.1182	2.25413	-0.24796
N	-0.02826	1.29006	2.09878
N	2.12595	0.69951	0.46547
Mn	-0.01933	0.594	0.10786
C	-0.15819	-1.13032	0.50224
O	-0.24201	-2.29797	0.66922
H	0.04557	2.30557	2.08356
C	0.7413	3.06193	-1.74379
O	-0.27309	3.37074	-2.27659
O	1.9181	2.98524	-1.59014
C	-0.00092	0.2092	-1.59999
O	0.01341	-0.04063	-2.75646
N	-2.15246	1.01334	0.34296
H	2.40805	1.42134	-0.19509
C	1.17417	0.7587	2.7475
H	1.07111	-0.32899	2.81838
H	1.31331	1.15986	3.75978
C	2.34333	1.1412	1.86836
H	2.43159	2.23129	1.83338
H	3.28155	0.73659	2.25522
C	-2.86934	-0.13041	-0.07667
C	-3.70161	-1.3252	-1.80272
C	-3.75992	-2.14703	0.40552
C	-4.02072	-2.36568	-0.93882
H	-3.91906	-1.39311	-2.86638
H	-4.02092	-2.89022	1.15603
H	-2.30928	1.76285	-0.32497
H	-4.47331	-3.2836	-1.2924
N	-3.19094	-1.02363	0.86079
N	-3.13092	-0.18982	-1.38842
C	-2.38143	1.48194	1.73627
H	-3.38738	1.2143	2.06813
H	-2.3118	2.57294	1.70591
C	-1.32891	0.93725	2.67521
H	-1.46649	1.35662	3.68033
H	-1.3863	-0.15369	2.74604
C	2.70052	-0.53231	0.08115
N	3.01322	-0.63427	-1.21729
N	2.85434	-1.45595	1.03196
C	3.44418	-1.83834	-1.60477
C	3.28731	-2.6484	0.60297
C	3.57871	-2.90786	-0.72768
H	3.70034	-1.94097	-2.65702
H	3.41021	-3.41597	1.3643
H	3.91907	-3.8805	-1.06023

1e-IN3R

Energy: -1370.745761 Hartree

N	-1.64811	-1.64729	-1.61337

N	0.73358	-1.82182	-0.23979
Mn	-0.99864	-0.61253	0.07428
C	-0.37118	0.75037	-0.8239
O	0.07616	1.68926	-1.38586
H	-2.05459	-2.51576	-1.26294
C	-1.5516	-2.6242	2.21622
O	-2.03222	-3.60589	2.79672
O	-1.79622	-2.28443	1.00047
C	-0.45525	0.20538	1.5367
O	-0.05966	0.80257	2.47532
N	-3.07628	0.04382	0.0222
H	0.70519	-2.44345	0.56551
C	-0.46184	-1.97851	-2.40943
H	-0.05922	-1.04682	-2.82219
H	-0.6963	-2.64605	-3.24836
C	0.51063	-2.64906	-1.46381
H	0.07979	-3.59692	-1.12766
H	1.46126	-2.87454	-1.95624
C	-3.10044	1.45033	0.14505
C	-2.97313	3.2387	1.51793
C	-2.99552	3.48417	-0.82635
C	-2.89667	4.0859	0.41898
H	-2.95531	3.6324	2.53178
H	-2.99226	4.07775	-1.73812
H	-3.4381	-0.37364	0.87587
H	-2.79339	5.15802	0.53013
N	-3.10927	2.15901	-0.98526
N	-3.08617	1.9123	1.40175
C	-3.72541	-0.55099	-1.17824
H	-4.51031	0.10955	-1.55342
H	-4.19499	-1.47928	-0.84151
C	-2.70832	-0.86188	-2.2535
H	-3.18574	-1.40302	-3.08031
H	-2.26067	0.05215	-2.65659
H	-0.8381	-1.96007	2.76134
C	1.99797	-1.07437	-0.18034
N	2.51937	-0.85756	1.05811
N	2.52391	-0.67205	-1.36956
C	3.68008	-0.17013	1.0952
C	3.68457	0.01265	-1.29746
C	4.31666	0.29625	-0.0713
H	4.11032	0.01031	2.09647
H	4.11847	0.34301	-2.25809
H	5.25863	0.85307	-0.02697

1e-IN4

Energy: -1371.876698 Hartree

H	0.61576	1.76318	-1.22972
N	0.45004	1.63356	1.38105
N	2.07433	-0.23575	0.18445
Mn	0.0383	0.4242	-0.28118
C	-0.73141	-0.89829	0.60063
O	-1.29038	-1.81228	1.095
H	0.95201	2.46219	1.06207
C	3.63081	3.23429	-1.068
O	4.76063	3.7175	-1.02926
O	3.30694	2.13654	-1.6842
C	-0.21588	-0.4531	-1.78314
O	-0.38556	-1.04002	-2.79186
N	-1.76467	1.65132	-0.3001
H	2.61875	0.09591	-0.61011
C	1.3429	0.8785	2.26842
H	0.78785	0.0176	2.65528
H	1.68391	1.48001	3.12056
C	2.52224	0.44289	1.43168
H	3.0911	1.32277	1.11596
H	3.1937	-0.20638	1.99763
H	-1.71346	2.11268	-1.20383
H	2.78024	3.74052	-0.55569
H	1.45452	1.82465	-1.3604
C	-1.64379	2.6383	0.80551
H	-2.63258	2.9606	1.14092
H	-1.12554	3.50509	0.38462
C	-0.83257	2.06964	1.94571
H	-0.70172	2.82233	2.73308
H	-1.32479	1.19549	2.38319
C	-2.90315	0.80858	-0.28707
N	-3.45952	0.55453	0.89739
N	-3.27684	0.34361	-1.48502
C	-4.4633	-0.33285	0.87689
C	-4.28344	-0.53514	-1.46451
C	-4.91024	-0.93311	-0.28977
H	-4.92438	-0.55721	1.8364
H	-4.60106	-0.92436	-2.4292

H	-5.71834	-1.6539	-0.28944
C	2.08038	-1.65066	0.18962
N	2.27763	-2.22329	-1.00388
N	1.87436	-2.254	1.36111
C	2.17135	-3.55559	-1.0187
C	1.77303	-3.58834	1.30446
C	1.893	-4.3005	0.12076
H	2.32628	-4.03919	-1.98065
H	1.59624	-4.09658	2.25006
H	1.7996	-5.3788	0.08963

1e-TS4-5

Energy: -1371.869510 Hartree

H	-0.3081	-2.42236	1.4661
N	-0.21467	-2.58147	-1.2806
N	1.98236	-1.79621	0.22787
Mn	-0.08448	-1.16861	0.27491
C	0.17917	0.19931	-0.79625
O	0.37159	1.16531	-1.45168
H	-0.40267	-3.5057	-0.88961
C	-2.0052	-4.7536	1.28342
O	-2.61365	-5.81062	1.14343
O	-0.74823	-4.57516	0.98942
C	-0.01104	-0.088	1.64854
O	0.03352	0.65259	2.571
N	-2.227	-1.18997	-0.01077
H	2.10484	-2.25074	1.12809
C	1.09913	-2.62149	-1.93323
H	1.2468	-1.66802	-2.45306
H	1.16901	-3.42609	-2.67646
C	2.11969	-2.83018	-0.83616
H	1.93562	-3.79866	-0.36067
H	3.13967	-2.84618	-1.23374
H	-2.56617	-1.64758	0.83133
H	-2.51266	-3.85008	1.68981
H	-0.47717	-3.37284	1.20959
C	-2.5484	-2.06802	-1.1745
H	-3.43308	-1.6987	-1.70231
H	-2.79867	-3.0557	-0.77356
C	-1.36486	-2.19772	-2.10773
H	-1.57859	-2.93695	-2.89055
H	-1.1278	-1.24616	-2.59696
C	-2.80598	0.16085	-0.04172
N	-3.00533	0.70974	-1.27119
N	-3.08622	0.73089	1.16212
C	-3.53743	1.94987	-1.28209
C	-3.6172	1.97069	1.11612
C	-3.86779	2.63797	-0.09852
H	-3.70261	2.40292	-2.27592
H	-3.84775	2.44082	2.08877
H	-4.29915	3.64432	-0.12163
C	2.92531	-0.67022	0.16549
N	3.20918	-0.04795	1.34227
N	3.42251	-0.3598	-1.06299
C	4.07632	0.98339	1.26769
C	4.28662	0.676	-1.10286
C	4.65455	1.39459	0.05112
H	4.31236	1.49416	2.21828
H	4.69506	0.93474	-2.09607
H	5.35705	2.23344	0.00469

1e-IN5

Energy: -1371.868667 Hartree

N	0.45004	1.63356	1.38105
N	2.07433	-0.23575	0.18445
Mn	0.0383	0.4242	-0.28118
C	-0.73141	-0.89829	0.60063
O	-1.29038	-1.81228	1.095
H	0.95201	2.46219	1.06207
C	3.13776	3.94937	-1.91304
O	4.11294	4.66026	-2.14788
O	2.91777	2.7822	-2.4412
C	-0.21588	-0.4531	-1.78314
O	-0.38556	-1.04002	-2.79186
N	-1.76467	1.65132	-0.3001
H	2.61875	0.09591	-0.61011
C	1.3429	0.8785	2.26842
H	0.78785	0.0176	2.65528
H	1.68391	1.48001	3.12056
C	2.52224	0.44289	1.43168
H	3.0911	1.32277	1.11596
H	3.1937	-0.20638	1.99763
H	-1.71346	2.11268	-1.20383
H	2.34782	4.28765	-1.20328

C	-1.64379	2.6383	0.80551
H	-2.63258	2.9606	1.14092
H	-1.12554	3.50509	0.38462
C	-0.83257	2.06964	1.94571
H	-0.70172	2.82233	2.73308
H	-1.32479	1.19549	2.38319
C	-2.90315	0.80858	-0.28707
N	-3.45952	0.55453	0.89739
N	-3.27684	0.34361	-1.48502
C	-4.4633	-0.33285	0.87689
C	-4.28344	-0.53514	-1.46451
C	-4.91024	-0.93311	-0.28977
H	-4.92438	-0.55721	1.8364
H	-4.60106	-0.92436	-2.4292
H	-5.71834	-1.6539	-0.28944
C	2.08038	-1.65066	0.18962
N	2.27763	-2.22329	-1.00388
N	1.87436	-2.254	1.36111
C	2.17135	-3.55559	-1.0187
C	1.77303	-3.58834	1.30446
C	1.893	-4.3005	0.12076
H	2.32628	-4.03919	-1.98065
H	1.59624	-4.09658	2.25006
H	1.7996	-5.3788	0.08963
H	0.61227	1.33677	-1.28051
H	2.0602	2.363	-2.33898

Group-2

2a-IN1

Energy: -808.3919 Hartree

N	1.84058	1.82009	1.02176
N	-0.76447	1.28171	0.90015
Mn	0.90435	-0.102	0.92721
H	1.76775	2.1253	1.99119
C	0.03061	-1.60243	1.04555
O	-0.61549	-2.59322	1.12756
N	2.9608	-0.76307	1.26417
H	3.00217	-1.3544	2.06954
C	0.99137	-0.24489	-0.84124
O	1.02029	-0.43937	-2.01
H	-0.99118	1.56936	1.83067
H	0.81927	-0.01214	2.60886
C	3.25979	1.63163	0.68554
H	3.86283	2.52042	0.90746
H	3.32442	1.42951	-0.38757
C	3.77256	0.45754	1.48916
H	4.8244	0.26761	1.25787
H	3.6936	0.68241	2.55642
C	-0.30924	2.41567	0.1209
C	1.06466	2.69054	0.18191
C	-1.14081	3.19828	-0.67051
C	1.60336	3.7273	-0.57016
C	-0.59629	4.23631	-1.42716
H	-2.20423	2.98324	-0.71194
C	0.76976	4.49534	-1.38404
H	2.67002	3.92699	-0.52834
H	-1.24693	4.8376	-2.05456
H	1.1934	5.2969	-1.98112
H	3.29782	-1.25453	0.46112
H	-1.55866	0.85823	0.46435

2a-IN2

Energy: -996.965204 Hartree

N	-0.30929	-1.02458	0.50552
N	-0.59781	0.48715	-1.64163
Mn	0.78873	0.75041	-0.0334
H	0.08216	-1.78617	-0.05026
C	1.77666	2.05799	-0.6007
O	2.4643	2.94176	-0.99864
N	1.91382	0.16597	1.6512
H	2.9073	0.11722	1.44629
C	-0.24359	1.85657	0.86803
O	-0.8865	2.66193	1.45689
H	-0.19396	-0.22442	-2.24935
H	1.8285	-0.25161	-0.92493
C	-0.04109	-1.28123	1.92906
H	-0.39205	-2.26851	2.25201
H	-0.57563	-0.52175	2.51122
C	1.45254	-1.15915	2.1255
H	1.71901	-1.32785	3.17346
H	1.9626	-1.92463	1.53095
C	-1.81569	0.00658	-1.04131
C	-1.66699	-0.78515	0.10477

C	-3.08207	0.35454	-1.4905
C	-2.78511	-1.22508	0.80124
C	-4.20624	-0.08084	-0.78557
H	-3.18426	0.98126	-2.37237
C	-4.05823	-0.86526	0.35379
H	-2.66383	-1.8289	1.69591
H	-5.19677	0.20281	-1.1277
H	-4.93297	-1.19763	0.90434
C	2.8114	-2.75936	-1.3294
O	1.84126	-3.31411	-1.72778
O	3.93611	-2.59191	-0.99979
H	1.81137	0.84122	2.40273
H	-0.79275	1.2916	-2.23044

2a-TS2-3

Energy: -996.960621 Hartree

N	1.77693	1.81774	0.303
N	-0.81133	1.26204	0.32579
Mn	0.84478	-0.10864	0.48977
H	1.72545	2.29478	1.20281
C	-0.0176	-1.58425	0.81955
O	-0.65216	-2.55674	1.05133
N	2.89702	-0.69144	0.95235
H	2.93236	-1.13178	1.84949
C	0.91887	-0.49415	-1.23742
O	0.92145	-0.84321	-2.36916
H	-0.99283	1.69388	1.20928
H	0.70303	0.15237	2.15734
C	3.19219	1.57344	-0.01916
H	3.79784	2.48541	0.04265
H	3.24077	1.19382	-1.04368
C	3.71168	0.54975	0.96532
H	4.76173	0.32113	0.76385
H	3.63918	0.94992	1.98105
C	-0.3896	2.2545	-0.64116
C	0.98336	2.54121	-0.65466
C	-1.24221	2.8895	-1.53481
C	1.50167	3.4421	-1.57511
C	-0.71773	3.79087	-2.46245
H	-2.30452	2.66575	-1.52009
C	0.64641	4.06133	-2.48827
H	2.56754	3.64966	-1.58687
H	-1.38383	4.2769	-3.16856
H	1.05201	4.75606	-3.21714
C	0.88739	1.36236	3.54254
O	0.1183	2.20021	3.20276
O	1.69303	0.79691	4.19963
H	3.23994	-1.31697	0.25155
H	-1.62631	0.78366	-0.00125

2a-IN3

Energy: -996.982986 Hartree

N	-0.30929	-1.02458	0.50552
N	-0.59781	0.48715	-1.64163
Mn	0.78873	0.75041	-0.0334
H	0.08216	-1.78617	-0.05026
C	1.77666	2.05799	-0.6007
O	2.4643	2.94176	-0.99864
N	1.91382	0.16597	1.6512
H	2.9073	0.11722	1.44629
C	-0.24359	1.85657	0.86803
O	-0.8865	2.66193	1.45689
H	-0.19396	-0.22442	-2.24935
H	1.8285	-0.25161	-0.92493
C	-0.04109	-1.28123	1.92906
H	-0.39205	-2.26851	2.25201
H	-0.57563	-0.52175	2.51122
C	1.45254	-1.15915	2.1255
H	1.71901	-1.32785	3.17346
H	1.9626	-1.92463	1.53095
C	-1.81569	0.00658	-1.04131
C	-1.66699	-0.78515	0.10477
C	-3.08207	0.35454	-1.4905
C	-2.78511	-1.22508	0.80124
C	-4.20624	-0.08084	-0.78557
H	-3.18426	0.98126	-2.37237
C	-4.05823	-0.86526	0.35379
H	-2.66383	-1.8289	1.69591
H	-5.19677	0.20281	-1.1277
H	-4.93297	-1.19763	0.90434
C	2.50073	-1.96672	-1.20155
O	1.53059	-2.52146	-1.59993
O	3.62543	-1.79926	-0.87195
H	1.81137	0.84122	2.40273

H	-0.79275	1.2916	-2.23044

2a-IN3R

Energy: -997.007483 Hartree

N	1.73526	1.82025	0.81128
N	-0.84661	1.24135	0.76759
Mn	0.83205	-0.06731	0.55975
H	1.67059	1.96886	1.82056
C	0.00151	-1.61461	0.5036
O	-0.60248	-2.62531	0.45039
N	2.87726	-0.73593	0.74619
H	2.94796	-1.42056	1.47165
C	0.83256	-0.03217	-1.18916
O	0.8286	-0.05619	-2.37082
H	-1.03686	1.4084	1.73501
C	3.15332	1.70779	0.43526
H	3.73847	2.57784	0.75531
H	3.20734	1.63672	-0.65476
C	3.69001	0.45337	1.09196
H	4.73706	0.2969	0.81986
H	3.634	0.55471	2.18025
C	-0.46203	2.47807	0.11099
C	0.90867	2.7749	0.12745
C	-1.35175	3.33341	-0.52483
C	1.38869	3.90709	-0.51695
C	-0.8673	4.47069	-1.17287
H	-2.41231	3.10124	-0.53329
C	0.49497	4.75203	-1.17697
H	2.4532	4.12181	-0.51068
H	-1.56268	5.13193	-1.68034
H	0.8694	5.6315	-1.69147
O	0.78601	0.03793	2.64279
C	1.1767	-0.90491	3.4145
O	1.7481	-1.96269	3.09092
H	0.97042	-0.71735	4.48938
H	3.19037	-1.12319	-0.12098
H	-1.64935	0.8475	0.31982

2a-IN4

Energy: -998.147463 Hartree

H	1.91752	0.32253	-0.87994
N	-0.06692	-0.79125	0.68302
N	-0.68092	0.22905	-1.68216
Mn	0.53408	1.08998	-0.16044
C	-0.78055	1.99701	0.56372
O	-1.63069	2.66836	1.04022
H	0.53537	-1.50581	0.26784
C	3.24786	-2.19388	-0.71242
O	3.70183	-3.33346	-0.71516
O	2.07417	-1.86056	-1.17596
C	1.11618	2.53912	-0.92896
O	1.51379	3.52601	-1.45001
N	1.70758	1.12604	1.58646
H	-0.10239	-0.42444	-2.20986
H	2.68265	1.33704	1.39674
H	3.83409	-1.34177	-0.30377
H	1.94409	-0.68892	-1.01039
C	1.61629	-0.18686	2.26663
H	1.90443	-0.1164	3.31983
H	2.3192	-0.86734	1.77401
C	0.21352	-0.73029	2.12703
H	0.12902	-1.71043	2.61029
H	-0.51947	-0.05739	2.58678
C	-1.73937	-0.47658	-1.00064
C	-1.42241	-1.00455	0.25698
C	-3.03194	-0.57988	-1.49597
C	-2.39897	-1.6326	1.0194
C	-4.01548	-1.20487	-0.72717
H	-3.26927	-0.15413	-2.46715
C	-3.70073	-1.72689	0.52356
H	-2.14999	-2.0305	1.99884
H	-5.02989	-1.27408	-1.10752
H	-4.46835	-2.20669	1.12291
H	1.37981	1.84991	2.21927
H	-1.07608	0.87058	-2.36374

2a-TS4-5

Energy: -998.130637 Hartree

H	0.86851	0.49003	2.07922
N	1.85161	1.57832	-0.28533
N	-0.77082	1.33794	0.10434
Mn	0.78248	-0.10919	0.44555
C	0.62095	-0.87143	-1.13641
O	0.47958	-1.45004	-2.15587

H	1.96442	2.22689	0.49864
C	2.56634	2.35999	3.40229
O	3.21189	3.27198	3.91073
O	1.59113	2.52138	2.55279
C	-0.1702	-1.39743	1.15033
O	-0.85744	-2.24124	1.60705
N	2.80203	-0.82721	0.81634
H	-0.84427	1.95738	0.88593
H	2.91247	-1.04763	1.78548
H	2.78324	1.29674	3.65033
H	1.1936	1.41437	2.2428
C	3.73516	0.26567	0.43631
H	4.71398	-0.14234	0.17115
H	3.85801	0.88697	1.32792
C	3.18511	1.10426	-0.69507
H	3.87219	1.93211	-0.90577
H	3.06764	0.51148	-1.60672
C	-0.36438	2.07157	-1.08188
C	1.01726	2.18591	-1.28785
C	-1.25396	2.62961	-1.99094
C	1.50399	2.8323	-2.4172
C	-0.76246	3.27571	-3.12539
H	-2.32314	2.54129	-1.82399
C	0.60854	3.3699	-3.34218
H	2.57538	2.90744	-2.57722
H	-1.45909	3.70021	-3.84151
H	0.98839	3.86437	-4.23075
H	2.98266	-1.63811	0.25974
H	-1.64372	0.87716	-0.05606

2a-IN5

Energy: -998.127869 Hartree

H	1.91752	0.32253	-0.87994
N	-0.06692	-0.79125	0.68302
N	-0.68092	0.22905	-1.68216
Mn	0.53408	1.08998	-0.16044
C	-0.78055	1.99701	0.56372
O	-1.63069	2.66836	1.04022
H	0.53537	-1.50581	0.26784
C	3.24786	-2.19388	-0.71242
O	3.70183	-3.33346	-0.71516
O	2.07417	-1.86056	-1.17596
C	1.11618	2.53912	-0.92896
O	1.51379	3.52601	-1.45001
N	1.70758	1.12604	1.58646
H	-0.10239	-0.42444	-2.20986
H	2.68265	1.33704	1.39674
H	3.83409	-1.34177	-0.30377
H	1.94409	-0.68892	-1.01039
C	1.61629	-0.18686	2.26663
H	1.90443	-0.1164	3.31983
H	2.3192	-0.86734	1.77401
C	0.21352	-0.73029	2.12703
H	0.12902	-1.71043	2.61029
H	-0.51947	-0.05739	2.58678
C	-1.73937	-0.47658	-1.00064
C	-1.42241	-1.00455	0.25698
C	-3.03194	-0.57988	-1.49597
C	-2.39897	-1.6326	1.0194
C	-4.01548	-1.20487	-0.72717
H	-3.26927	-0.15413	-2.46715
C	-3.70073	-1.72689	0.52356
H	-2.14999	-2.0305	1.99884
H	-5.02989	-1.27408	-1.10752
H	-4.46835	-2.20669	1.12291
H	1.37981	1.84991	2.21927
H	-1.07608	0.87058	-2.36374

2b-IN1

Energy: -1538.066303 Hartree

N	0.00021	-3.25725	-0.59253
N	2.14451	-1.93523	0.58784
Mn	0.00025	-1.59636	0.72173
H	0.00019	-4.11837	-0.04995
C	0.00045	-0.34165	1.92855
O	0.00059	0.51662	2.75157
N	-2.14421	-1.93522	0.58778
C	2.88623	-0.73706	0.31012
C	3.91075	1.29688	1.14163
C	3.90841	0.83928	-1.21733
H	4.17506	1.9082	1.99704
H	4.16705	1.08926	-2.23998
C	-2.88604	-0.73712	0.31013

C	-3.91151	1.29634	1.14174
C	-3.90822	0.83924	-1.21733
C	-4.24986	1.70774	-0.16372
H	-4.17621	1.90743	1.9972
H	-4.16644	1.08955	-2.24002
H	-2.37819	-2.23476	1.52894
C	0.0002	-0.38888	-0.57074
O	0.00029	0.48258	-1.37673
H	2.37862	-2.2347	1.52901
H	0.00018	-2.74277	1.97301
C	3.23506	-0.35598	-0.98386
H	2.98329	-0.98673	-1.83204
C	4.2493	1.70815	-0.16383
C	3.23986	0.10319	1.36672
H	2.98735	-0.18641	2.38496
N	4.8722	2.93693	-0.40168
C	5.56632	3.5423	0.7224
H	6.0353	4.46919	0.39016
H	4.86893	3.80064	1.52297
H	6.34713	2.89349	1.14939
C	5.56307	3.07873	-1.67205
H	4.86319	3.02014	-2.50922
H	6.03338	4.06187	-1.71081
H	6.34218	2.31604	-1.82805
C	-1.25218	-3.19434	-1.35098
H	-1.40422	-4.07896	-1.98385
H	-1.20469	-2.31521	-2.00391
C	-2.36932	-3.07956	-0.33683
H	-3.34595	-2.99898	-0.82566
H	-2.38227	-3.98542	0.27714
C	-3.23454	-0.35582	-0.98392
H	-2.98211	-0.98626	-1.83215
C	-3.24028	0.1028	1.36679
H	-2.98794	-0.18688	2.38505
N	-4.87331	2.93618	-0.40161
C	-5.56402	3.0778	-1.67209
H	-6.03551	4.06038	-1.71049
H	-4.86386	3.0204	-2.50914
H	-6.34216	2.31425	-1.82867
C	-5.56712	3.54178	0.72248
H	-4.86951	3.80031	1.5228
H	-6.03624	4.46858	0.39018
H	-6.3478	2.89307	1.14991
C	2.36692	-3.06554	-0.32531
C	1.28133	-3.68773	-0.94244
C	3.66321	-3.51596	-0.5749
C	1.49204	-4.76053	-1.80842
C	3.87422	-4.58844	-1.44188
H	4.51895	-3.02557	-0.08873
C	2.78891	-5.21084	-2.05853
H	0.63637	-5.2514	-2.2944
H	4.89643	-4.9431	-1.6388
H	2.9548	-6.05669	-2.74141

2b-IN2

Energy: -1726.642985 Hartree

H	0.27413	-1.04023	2.40939
N	-0.18408	-2.85053	0.60496
N	1.98086	-1.30747	0.38415
Mn	-0.08008	-0.72141	0.75423
C	-0.48105	-0.46785	-0.9477
O	-0.76725	-0.24593	-2.07486
H	0.11701	-3.22162	1.50537
C	0.2078	0.17151	3.77858
O	-0.95254	0.17439	4.03855
O	1.35345	0.45919	3.8906
C	0.13062	0.99574	0.97205
O	0.32585	2.15824	1.10115
N	-2.11379	-0.95208	1.39226
H	2.25085	-1.63897	1.31002
C	2.81587	-0.1918	0.03296
C	4.36462	1.5335	0.72428
C	3.45361	1.5604	-1.50354
C	4.27845	2.16567	-0.53125
H	5.00133	1.939	1.50207
H	3.36711	1.9921	-2.49401

C	-3.05138	-0.14143	0.66406
C	-4.04296	2.04251	0.31681
C	-4.64166	0.21514	-1.12506
C	-4.7775	1.57589	-0.79327
H	-4.13253	3.07292	0.64132
H	-5.20475	-0.20727	-1.94946
H	-2.06634	-0.60022	2.34644
C	2.72733	0.41268	-1.22123
H	2.08457	-0.01615	-1.98609
C	3.64387	0.37613	0.99651
H	3.72152	-0.08719	1.97792
N	4.95771	3.35472	-0.7964
C	6.07448	3.67774	0.07503
H	6.53003	4.60857	-0.26406
H	5.73805	3.83582	1.10266
H	6.85084	2.89705	0.08898
C	5.16721	3.69321	-2.19428
H	4.21701	3.8721	-2.70344
H	5.74383	4.61696	-2.25119
H	5.70874	2.91266	-2.7507
C	-2.39016	-2.41185	1.44904
H	-3.46261	-2.61095	1.35069
H	-2.08169	-2.7507	2.44321
C	-1.60472	-3.17813	0.40537
H	-1.80559	-4.2518	0.50362
H	-1.87171	-2.87413	-0.61278
C	-3.19572	1.20031	1.02144
H	-2.63689	1.58858	1.87124
C	-3.79008	-0.62489	-0.41295
H	-3.71671	-1.66654	-0.7127
N	-5.58426	2.43456	-1.5438
C	-6.01258	3.66152	-0.89304
H	-5.15924	4.30499	-0.6653
H	-6.66217	4.21407	-1.57288
H	-6.56339	3.48436	0.0439
C	-6.60261	1.81097	-2.37135
H	-7.17801	2.59047	-2.87188
H	-6.15062	1.19034	-3.14898
H	-7.29982	1.18058	-1.79738
C	1.89935	-2.43325	-0.52552
C	2.86033	-2.72583	-1.48576
C	0.75351	-3.2351	-0.41414
C	2.66223	-3.79811	-2.35599
H	3.74695	-2.10434	-1.56713
C	0.56012	-4.30364	-1.28063
C	1.51448	-4.57852	-2.26059
H	3.40895	-4.01526	-3.11345
H	-0.33624	-4.91081	-1.19583
H	1.35745	-5.40556	-2.94596

2b-TS2-3

Energy: -1726.636086 Hartree

H	0.01826	-2.83529	0.96737
N	0.00814	-2.77426	-1.59667
N	2.11648	-1.70019	-0.1446
Mn	-0.01456	-1.40268	0.01264
C	-0.04067	0.018	-1.0329
O	-0.05158	1.00672	-1.68606
H	0.01633	-3.72247	-1.22607
C	0.4176	-2.86681	2.77241
O	-0.67022	-2.88379	3.24614
O	1.60529	-2.88936	2.75015
C	-0.03925	-0.36206	1.41401
O	-0.05568	0.35562	2.35976
N	-2.1383	-1.78374	-0.15126
H	2.31587	-2.19421	0.72246
C	2.86181	-0.47238	-0.13546
C	3.89064	1.30933	1.14534
C	3.87563	1.41856	-1.25576
C	4.22153	2.01598	-0.02892
H	4.16081	1.70354	2.11837
H	4.1295	1.90246	-2.19206
C	-2.92152	-0.58022	-0.11351
C	-4.01486	1.13036	1.20961
C	-3.98199	1.31107	-1.1875
C	-4.35734	1.86056	0.05329

H	-4.3048	1.48753	2.19115
H	-4.24186	1.81587	-2.11103
H	-2.33144	-2.30515	0.69914
C	3.20291	0.20176	-1.3061
H	2.94511	-0.21322	-2.2769
C	3.22119	0.09491	1.08766
H	2.9733	-0.42677	2.01033
N	4.84286	3.26681	0.02518
C	5.54978	3.58916	1.2533
H	6.0164	4.56892	1.14493
H	4.86132	3.6493	2.09978
H	6.33441	2.85863	1.50534
C	5.51737	3.70959	-1.18315
H	4.80602	3.85322	-2.00024
H	5.98997	4.67353	-0.99089
H	6.29229	3.0072	-1.52838
C	-2.36471	-2.68877	-1.31475
H	-3.33902	-2.49246	-1.77353
H	-2.38566	-3.70986	-0.92181
C	-1.24645	-2.57823	-2.32952
H	-1.38586	-3.31966	-3.12737
H	-1.2144	-1.58533	-2.79231
C	-3.30823	-0.06101	1.12213
H	-3.05072	-0.60207	2.03138
C	-3.2715	0.11762	-1.26743
H	-2.9897	-0.26034	-2.24684
N	-5.01748	3.08964	0.13749
C	-5.73673	3.35843	1.37128
H	-5.05204	3.41817	2.2209
H	-6.23336	4.32567	1.28661
H	-6.49861	2.59756	1.60246
C	-5.70253	3.54173	-1.0615
H	-6.20596	4.485	-0.84654
H	-4.99406	3.72847	-1.87228
H	-6.45383	2.82414	-1.42701
C	2.37025	-2.6113	-1.26994
C	3.67828	-2.97371	-1.59271
C	1.3034	-3.11359	-2.01498
C	3.91936	-3.83769	-2.66072
H	4.51919	-2.57693	-1.00562
C	1.54432	-3.97862	-3.08273
C	2.85204	-4.34061	-3.40578
H	4.95038	-4.12289	-2.91559
H	0.70293	-4.37482	-3.66973
H	3.04236	-5.02186	-4.24779

2b-IN3

Energy: -1726.655935 Hartree

H	0.27413	-1.04023	2.40939
N	-0.18408	-2.85053	0.60496
N	1.98086	-1.30747	0.38415
Mn	-0.08008	-0.72141	0.75423
C	-0.48105	-0.46785	-0.9477
O	-0.76725	-0.24593	-2.07486
H	0.11701	-3.22162	1.50537
C	0.2078	0.17151	3.77858
O	-0.95254	0.17439	4.03855
O	1.35345	0.45919	3.8906
C	0.13062	0.99574	0.97205
O	0.32585	2.15824	1.10115
N	-2.11379	-0.95208	1.39226
H	2.25085	-1.63897	1.31002
C	2.81587	-0.1918	0.03296
C	4.36462	1.5335	0.72428
C	3.45361	1.5604	-1.50354
C	4.27845	2.16567	-0.53125
H	5.00133	1.939	1.50207
H	3.36711	1.9921	-2.49401
C	-3.05138	-0.14143	0.66406
C	-4.04296	2.04251	0.31681
C	-4.64166	0.21514	-1.12506
C	-4.7775	1.57589	-0.79327
H	-4.13253	3.07292	0.64132
H	-5.20475	-0.20727	-1.94946
H	-2.06634	-0.60022	2.34644
C	2.72733	0.41268	-1.22123

H	2.08457	-0.01615	-1.98609
C	3.64387	0.37613	0.99651
H	3.72152	-0.08719	1.97792
N	4.95771	3.35472	-0.7964
C	6.07448	3.67774	0.07503
H	6.53003	4.60857	-0.26406
H	5.73805	3.83582	1.10266
H	6.85084	2.89705	0.08898
C	5.16721	3.69321	-2.19428
H	4.21701	3.8721	-2.70344
H	5.74383	4.61696	-2.25119
H	5.70874	2.91266	-2.7507
C	-2.39016	-2.41185	1.44904
H	-3.46261	-2.61095	1.35069
H	-2.08169	-2.7507	2.44321
C	-1.60472	-3.17813	0.40537
H	-1.80559	-4.2518	0.50362
H	-1.87171	-2.87413	-0.61278
C	-3.19572	1.20031	1.02144
H	-2.63689	1.58858	1.87124
C	-3.79008	-0.62489	-0.41295
H	-3.71671	-1.66654	-0.7127
N	-5.58426	2.43456	-1.5438
C	-6.01258	3.66152	-0.89304
H	-5.15924	4.30499	-0.6653
H	-6.66217	4.21407	-1.57288
H	-6.56339	3.48436	0.0439
C	-6.60261	1.81097	-2.37135
H	-7.17801	2.59047	-2.87188
H	-6.15062	1.19034	-3.14898
H	-7.29982	1.18058	-1.79738
C	1.89935	-2.43325	-0.52552
C	2.86033	-2.72583	-1.48576
C	0.75351	-3.2351	-0.41414
C	2.66223	-3.79811	-2.35599
H	3.74695	-2.10434	-1.56713
C	0.56012	-4.30364	-1.28063
C	1.51448	-4.57852	-2.26059
H	3.40895	-4.01526	-3.11345
H	-0.33624	-4.91081	-1.19583
H	1.35745	-5.40556	-2.94596

2b-IN3R

Energy: -1726.680709 Hartree

N	0.01606	-2.70603	-1.50604
N	2.12489	-1.65342	-0.07298
Mn	0.00285	-1.35788	0.08486
C	-0.01316	0.05427	-0.93907
O	-0.02157	1.03456	-1.60206
H	0.02667	-3.63598	-1.08541
C	0.3536	-3.22982	2.39481
O	0.37131	-4.2761	3.05666
O	-0.00428	-3.13232	1.16431
C	-0.01302	-0.27011	1.46531
O	-0.02984	0.51184	2.35243
N	-2.10803	-1.70705	-0.06757
H	2.34182	-2.15001	0.78799
C	2.87242	-0.42484	-0.06693
C	3.89035	1.36267	1.21273
C	3.88991	1.46115	-1.18962
C	4.22768	2.0642	0.03687
H	4.15411	1.76158	2.18553
H	4.14885	1.94125	-2.12641
C	-2.8929	-0.5032	-0.0448
C	-3.98498	1.22796	1.2514
C	-3.95194	1.37183	-1.14833
C	-4.32643	1.94092	0.08399
H	-4.27562	1.59997	2.22721
H	-4.21158	1.86212	-2.07965
H	-2.28607	-2.21606	0.79495
C	3.21923	0.24318	-1.23879
H	2.96788	-0.17686	-2.20906
C	3.22201	0.14798	1.15592
H	2.96711	-0.36994	2.0791
N	4.84744	3.31472	0.08931
C	5.54338	3.6466	1.32117

H	6.00889	4.62652	1.2101
H	4.84775	3.71096	2.16147
H	6.32724	2.91935	1.58435
C	5.52583	3.75631	-1.11729
H	4.81745	3.89651	-1.93756
H	5.99554	4.72164	-0.92518
H	6.30356	3.05476	-1.45751
C	-2.35136	-2.63382	-1.21255
H	-3.33041	-2.44272	-1.66249
H	-2.37007	-3.64716	-0.8007
C	-1.24312	-2.54207	-2.23957
H	-1.37829	-3.30856	-3.01349
H	-1.22231	-1.5644	-2.73388
H	0.67478	-2.27277	2.87377
C	-5.66274	3.61167	-1.05786
H	-6.16282	4.55963	-0.85617
H	-4.94981	3.78548	-1.86763
H	-6.41559	2.89309	-1.41791
C	-5.70324	3.46167	1.37854
H	-6.19721	4.42872	1.27815
H	-6.46713	2.70672	1.62168
H	-5.01832	3.53311	2.22707
C	-3.27876	0.0351	1.18259
H	-3.02373	-0.49195	2.10009
C	-3.24298	0.1767	-1.20943
H	-2.96293	-0.21687	-2.18306
N	-4.9848	3.17097	0.14932
C	2.37823	-2.56717	-1.19627
C	1.31131	-3.06452	-1.94513
C	3.6852	-2.93691	-1.51354
C	1.55135	-3.93194	-3.01059
C	3.92556	-3.8038	-2.57991
H	4.52623	-2.54487	-0.92353
C	2.85892	-4.30144	-3.32834
H	0.7104	-4.32449	-3.6005
H	4.95619	-4.09481	-2.83009
H	3.04794	-4.98533	-4.16849

2b-IN4

Energy: -1727.818535 Hartree

H	0.29757	-1.46444	2.05895
N	-0.19219	-2.59976	-0.43387
N	1.96674	-1.07227	-0.06105
Mn	-0.08077	-0.70764	0.53272
C	-0.49971	0.20317	-0.91346
O	-0.7974	0.87848	-1.83668
H	0.10767	-3.30652	0.2428
C	-0.0543	-4.10983	3.07207
O	-0.08366	-5.2845	3.4259
O	0.80634	-3.60664	2.23137
C	0.12175	0.79403	1.40298
O	0.30677	1.81698	1.96626
N	-2.12358	-1.16518	1.02487
H	2.28167	-1.72849	0.65437
C	2.78604	0.10947	0.01261
C	4.34316	1.48059	1.25622
C	3.3732	2.31401	-0.78286
H	4.99913	1.57939	2.11338
H	3.25888	3.07834	-1.5428
C	-3.04262	-0.12977	0.62626
C	-3.98451	2.06056	1.05823
C	-4.59022	0.87231	-0.94123
C	-4.70514	2.03311	-0.15445
H	-4.05966	2.9138	1.72274
H	-5.14413	0.78058	-1.86846
H	-2.11926	-1.20556	2.04061
H	-0.78867	-3.37283	3.46729
H	0.53396	-2.44012	2.09273
C	2.66256	1.13183	-0.92824
H	2.00712	1.00461	-1.78603
C	4.21918	2.53078	0.32593
C	3.63617	0.29385	1.09848
H	3.74412	-0.4976	1.83756
N	4.88215	3.74413	0.50418
C	6.01429	3.74524	1.41456
H	6.44849	4.74527	1.43854

H	5.69933	3.50308	2.43256
H	6.80206	3.03265	1.12482
C	5.05306	4.5789	-0.67327
H	4.08864	4.91567	-1.0612
H	5.61903	5.4687	-0.39628
H	5.58795	4.06897	-1.48924
C	-2.40025	-2.53302	0.51074
H	-3.47269	-2.67845	0.34382
H	-2.09306	-3.23312	1.2938
C	-1.61338	-2.82925	-0.74799
H	-1.80671	-3.85921	-1.07022
H	-1.88376	-2.15996	-1.57165
C	-3.16768	1.00369	1.43126
H	-2.61501	1.05691	2.36709
C	-3.77125	-0.18639	-0.559
H	-3.71484	-1.05672	-1.20603
N	-5.47797	3.12105	-0.56455
C	-6.4864	2.85937	-1.57703
H	-7.03198	3.78189	-1.77831
H	-6.02697	2.54112	-2.516
H	-7.21113	2.08695	-1.27558
C	-5.89872	4.05118	0.46991
H	-5.03975	4.55294	0.92193
H	-6.52335	4.82194	0.01723
H	-6.47369	3.56965	1.27608
C	1.8833	-1.77224	-1.33043
C	0.7425	-2.5627	-1.52709
C	2.84665	-1.67958	-2.32762
C	0.55607	-3.23263	-2.73046
C	2.65543	-2.35032	-3.53523
H	3.73005	-1.06878	-2.16828
C	1.51209	-3.11634	-3.73964
H	-0.33481	-3.83472	-2.88238
H	3.40339	-2.26566	-4.31742
H	1.35968	-3.62965	-4.68393

2b-TS4-5

Energy: -1727.804531 Hartree

H	-0.3081	-2.42236	1.4661
N	-0.21467	-2.58147	-1.2806
N	1.98236	-1.79621	0.22787
Mn	-0.08448	-1.16861	0.27491
C	0.17917	0.19931	-0.79625
O	0.37159	1.16531	-1.45168
H	-0.40267	-3.5057	-0.88961
C	-1.99596	-4.71504	1.28948
O	-2.60441	-5.77207	1.14949
O	-0.73899	-4.5366	0.99548
C	-0.01104	-0.088	1.64854
O	0.03352	0.65259	2.571
N	-2.227	-1.18997	-0.01077
H	2.10484	-2.25074	1.12809
C	2.90533	-0.69408	0.16681
C	4.11876	1.04686	1.33682
C	4.26227	0.89205	-1.05742
H	4.38795	1.50153	2.28341
H	4.64194	1.22712	-2.01586
C	-2.79366	0.1321	-0.04107
C	-3.67587	2.03226	1.17576
C	-3.44588	2.14492	-1.21469
C	-3.81154	2.77964	-0.01201
H	-3.96975	2.45613	2.12918
H	-3.55254	2.66105	-2.16192
H	-2.56617	-1.64758	0.83133
H	-2.50342	-3.81152	1.69587
H	-0.47923	-3.38442	1.20647
C	3.41492	-0.21146	-1.03626
H	3.15824	-0.68978	-1.97736
C	4.62341	1.56368	0.12556
C	3.27412	-0.05384	1.35029
H	2.89616	-0.42899	2.29923
N	5.4263	2.70643	0.10037
C	6.10038	3.05739	1.33919
H	6.71787	3.93959	1.16695
H	5.38051	3.31243	2.12065
H	6.74663	2.25253	1.72279

C	6.22908	2.91103	-1.09334
H	5.59652	3.06686	-1.97073
H	6.83156	3.81073	-0.96364
H	6.90574	2.06929	-1.30876
C	-2.5484	-2.06802	-1.1745
H	-3.43308	-1.6987	-1.70231
H	-2.79867	-3.0557	-0.77356
C	-1.36486	-2.19772	-2.10773
H	-1.57859	-2.93695	-2.89055
H	-1.1278	-1.24616	-2.59696
C	-3.17317	0.73858	1.15606
H	-3.0752	0.18558	2.08838
C	-2.93984	0.84933	-1.22629
H	-2.65666	0.40604	-2.17755
N	-4.26475	4.10092	0.00716
C	-4.77561	4.63153	-1.24533
H	-5.13112	5.64892	-1.07909
H	-3.98838	4.68216	-2.0016
H	-5.60569	4.03815	-1.65957
C	-5.01457	4.51625	1.18073
H	-4.39081	4.48703	2.0775
H	-5.33821	5.54891	1.04534
H	-5.90543	3.89627	1.36682
C	2.11785	-2.8163	-0.82188
C	1.02995	-3.12518	-1.6389
C	3.33401	-3.47534	-1.00123
C	1.15807	-4.09339	-2.63458
C	3.46262	-4.44316	-1.99782
H	4.1916	-3.23188	-0.35749
C	2.37489	-4.75236	-2.81437
H	0.30044	-4.33736	-3.2782
H	4.42175	-4.96233	-2.13927
H	2.47569	-5.51568	-3.59954

2b-IN5

Energy: -1727.803701 Hartree

H	0.29757	-1.46444	2.05895
N	-0.19219	-2.59976	-0.43387
N	1.96674	-1.07227	-0.06105
Mn	-0.08077	-0.70764	0.53272
C	-0.49971	0.20317	-0.91346
O	-0.7974	0.87848	-1.83668
H	0.10767	-3.30652	0.2428
C	-0.0543	-4.10983	3.07207
O	-0.08366	-5.2845	3.4259
O	0.80634	-3.60664	2.23137
C	0.12175	0.79403	1.40298
O	0.30677	1.81698	1.96626
N	-2.12358	-1.16518	1.02487
H	2.28167	-1.72849	0.65437
C	2.78604	0.10947	0.01261
C	4.34316	1.48059	1.25622
C	3.3732	2.31401	-0.78286
H	4.99913	1.57939	2.11338
H	3.25888	3.07834	-1.5428
C	-3.04262	-0.12977	0.62626
C	-3.98451	2.06056	1.05823
C	-4.59022	0.87231	-0.94123
C	-4.70514	2.03311	-0.15445
H	-4.05966	2.9138	1.72274
H	-5.14413	0.78058	-1.86846
H	-2.11926	-1.20556	2.04061
H	-0.78867	-3.37283	3.46729
H	0.53396	-2.44012	2.09273
C	2.66256	1.13183	-0.92824
H	2.00712	1.00461	-1.78603
C	4.21918	2.53078	0.32593
C	3.63617	0.29385	1.09848
H	3.74412	-0.4976	1.83756
N	4.88215	3.74413	0.50418
C	6.01429	3.74524	1.41456
H	6.44849	4.74527	1.43854
H	5.69933	3.50308	2.43256
H	6.80206	3.03265	1.12482
C	5.05306	4.5789	-0.67327
H	4.08864	4.91567	-1.0612

H 5.61903 5.4687 -0.39628
H 5.58795 4.06897 -1.48924
C -2.40025 -2.53302 0.51074
H -3.47269 -2.67845 0.34382
H -2.09306 -3.23312 1.2938
C -1.61338 -2.82925 -0.74799
H -1.80671 -3.85921 -1.07022
H -1.88376 -2.15996 -1.57165
C -3.16768 1.00369 1.43126
H -2.61501 1.05691 2.36709
C -3.77125 -0.18639 -0.559
H -3.71484 -1.05672 -1.20603
N -5.47797 3.12105 -0.56455
C -6.4864 2.85937 -1.57703
H -7.03198 3.78189 -1.77831
H -6.02697 2.54112 -2.516
H -7.21113 2.08695 -1.27558
C -5.89872 4.05118 0.46991
H -5.03975 4.55294 0.92193
H -6.52335 4.82194 0.01723
H -6.47369 3.56965 1.27608
C 1.8833 -1.77224 -1.33043
C 0.7425 -2.5627 -1.52709
C 2.84665 -1.67958 -2.32762
C 0.55607 -3.23263 -2.73046
C 2.65543 -2.35032 -3.53523
H 3.73005 -1.06878 -2.16828
C 1.51209 -3.11634 -3.73964
H -0.33481 -3.83472 -2.88238
H 3.40339 -2.26566 -4.31742
H 1.35968 -3.62965 -4.68393

2c-IN1

Energy: -1420.236884 Hartree

N -1.58673 -2.54812 0.29616
N -3.18318 -0.40088 -0.42167
Mn -1.05593 -0.78552 -0.75169
H -1.95242 -3.23503 -0.35987
C -0.66705 0.57635 -1.76611
O -0.39961 1.5025 -2.46021
N 0.8192 -1.86206 -0.93257
C -0.35085 -3.08226 0.87599
C 0.65423 -3.17395 -0.25101
C -3.39834 0.92995 0.03105
C -3.76551 3.10575 -0.56271
C -3.53416 2.58137 1.7502
H -3.93255 3.8157 -1.37006
H -3.50962 2.86634 2.79775
N -3.61324 1.82673 -0.93624
C 1.96305 -1.08338 -0.54465
C 2.52245 -0.19706 -1.46611
C 2.51553 -1.14669 0.73248
C 3.59445 0.61402 -1.12294
H 2.11149 -0.14594 -2.4723
C 3.58999 -0.33512 1.08446
H 2.1151 -1.83147 1.47472
C 4.14835 0.58058 0.17349
H 4.00283 1.27566 -1.87838
H 3.9928 -0.42628 2.08664
N 5.18911 1.43729 0.54237
C 5.98734 1.9927 -0.53799
H 5.38624 2.64386 -1.1772
H 6.78338 2.6038 -0.11115
H 6.44751 1.222 -1.17592
C 5.95765 1.05698 1.71535
H 6.75444 1.78581 1.86817
H 5.33427 1.06442 2.61301
H 6.4142 0.05849 1.62825
H 0.87989 -2.02772 -1.9322
C -0.52338 0.09192 0.69252
O -0.12837 0.74012 1.60435
H -3.5604 -0.46464 -1.3621
H -1.53209 -1.61811 -2.14376
C -3.36353 1.25298 1.39046
H -3.19161 0.48864 2.14149
C -3.73304 3.53757 0.75577

H	-3.86604	4.58671	0.99527
H	-0.02279	-2.38909	1.6592
H	-0.49694	-4.06845	1.33617
H	0.27807	-3.87574	-1.0018
H	1.61852	-3.55407	0.1023
C	-3.72914	-1.48658	0.40543
C	-5.06074	-1.44577	0.81971
C	-2.91601	-2.55759	0.77597
C	-5.57883	-2.47546	1.60492
H	-5.70133	-0.60089	0.52806
C	-3.43437	-3.58816	1.56059
C	-4.76552	-3.5472	1.97521
H	-6.62824	-2.44305	1.93203
H	-2.79314	-4.43269	1.85225
H	-5.17425	-4.35898	2.59427

2c-IN2

Energy: -1608.811702 Hartree

H	1.14651	-0.33462	2.1813
N	1.26508	-2.03085	0.09033
N	2.6324	0.25313	0.05984
Mn	0.54315	-0.08751	0.61175
C	-0.11302	0.25136	-0.99694
O	-0.58863	0.55014	-2.0391
H	1.7908	-2.38595	0.89061
C	1.67267	-1.58423	3.41848
O	0.8028	-1.46894	4.21459
O	2.6707	-1.99144	2.92052
C	0.09387	1.50297	1.1648
O	-0.15637	2.60409	1.52367
N	-1.16577	-1.21888	1.2609
H	3.12253	-0.04564	0.90351
C	2.84251	1.6523	-0.10585
C	3.47919	3.59813	0.89885
C	2.53578	3.69685	-1.28962
C	3.07218	4.36032	-0.18508
H	3.9132	4.07361	1.77535
H	2.20815	4.25023	-2.16444
H	3.17574	5.43947	-0.16845
N	3.38401	2.25928	0.95109
C	-2.40626	-0.75279	0.70001
C	-3.02726	0.35606	1.27644
C	-2.98859	-1.33032	-0.42533
C	-4.19169	0.88484	0.73989
H	-2.59104	0.81045	2.1638
C	-4.15624	-0.80371	-0.97019
H	-2.54417	-2.20281	-0.89548
C	-4.77988	0.32926	-0.41558
H	-4.64468	1.73801	1.23203
H	-4.57937	-1.29253	-1.84034
N	-5.91682	0.89536	-0.99575
C	-6.73133	1.74466	-0.14286
H	-6.18022	2.63619	0.1663
H	-7.60161	2.08053	-0.70782
H	-7.08544	1.23262	0.76538
C	-6.678	0.04923	-1.89848
H	-7.55105	0.60149	-2.24781
H	-6.0875	-0.21708	-2.77865
H	-7.02426	-0.88411	-1.42752
H	-1.18549	-1.0343	2.26044
C	2.41461	2.31723	-1.25861
H	1.98364	1.76501	-2.08763
C	0.07605	-2.87404	-0.11807
H	0.32699	-3.93817	-0.20163
H	-0.38745	-2.55982	-1.05941
C	-0.84469	-2.65808	1.06351
H	-1.75972	-3.24967	0.95481
H	-0.34222	-2.99897	1.97449
C	2.16051	-1.85394	-1.02049
C	2.88832	-0.65623	-1.03623
C	2.30714	-2.77155	-2.05352
C	3.78392	-0.39501	-2.06597
C	3.18423	-2.49414	-3.10226
H	1.73199	-3.69255	-2.04282
C	3.92426	-1.31594	-3.1041
H	4.35317	0.52989	-2.0679

H	3.28956	-3.20557	-3.91535
H	4.61337	-1.10459	-3.91561

2c-TS2-3

Energy: -1608.806584 Hartree

H	1.5411	-1.62369	1.34062
N	1.51266	-1.82826	-1.3045
N	2.90714	0.21511	-0.06716
Mn	0.87738	-0.51819	0.22821
C	0.11894	0.64914	-0.85965
O	-0.42909	1.46744	-1.51898
H	2.00801	-2.62423	-0.90398
C	3.01174	-2.71048	1.59012
O	2.59268	-3.74388	1.18633
O	3.77915	-1.93648	2.0586
C	0.42884	0.43617	1.61745
O	0.12502	1.07813	2.56725
N	-0.82572	-1.85631	0.1985
H	3.49142	-0.15868	0.67785
C	2.89258	1.62201	0.14835
C	3.02253	3.31335	1.6785
C	2.63373	3.87221	-0.60544
C	2.80298	4.28719	0.71439
H	3.17206	3.59327	2.71912
H	2.46604	4.59523	-1.3981
H	2.77123	5.33584	0.98828
N	3.07983	1.99925	1.41629
C	-2.08651	-1.1667	0.14191
C	-2.65196	-0.6934	1.32671
C	-2.75049	-0.91441	-1.05653
C	-3.83977	0.02351	1.31905
H	-2.15306	-0.89531	2.27251
C	-3.94214	-0.19596	-1.07193
H	-2.34848	-1.27413	-1.99959
C	-4.51078	0.30972	0.11216
H	-4.24639	0.35679	2.26703
H	-4.42811	-0.03275	-2.02709
N	-5.6743	1.08278	0.09203
C	-6.41342	1.18062	1.33953
H	-5.82553	1.69045	2.10676
H	-7.31209	1.77508	1.17109
H	-6.71736	0.20093	1.74017
C	-6.51295	0.96567	-1.08876
H	-7.39847	1.58816	-0.95589
H	-5.99215	1.32717	-1.97886
H	-6.84182	-0.06696	-1.28466
H	-0.76319	-2.31169	1.10396
C	2.67801	2.5183	-0.90211
H	2.53278	2.1637	-1.91753
C	0.29914	-2.33695	-1.95424
H	0.5187	-3.12197	-2.6895
H	-0.17354	-1.50111	-2.48236
C	-0.58521	-2.88133	-0.85464
H	-1.53247	-3.26003	-1.25219
H	-0.07286	-3.71858	-0.37053
C	2.41638	-1.03732	-2.15221
C	3.15872	0.00554	-1.59737
C	2.53185	-1.32946	-3.5112
C	4.0158	0.75648	-2.40156
C	3.38989	-0.57902	-4.31558
H	1.94698	-2.15147	-3.94863
C	4.13173	0.46391	-3.76104
H	4.60048	1.57889	-1.96443
H	3.48104	-0.80985	-5.38697
H	4.80762	1.0561	-4.3949

2c-IN3

Energy: -1608.826807 Hartree

H	1.14651	-0.33462	2.1813
N	1.26508	-2.03085	0.09033
N	2.6324	0.25313	0.05984
Mn	0.54315	-0.08751	0.61175
C	-0.11302	0.25136	-0.99694
O	-0.58863	0.55014	-2.0391
H	1.7908	-2.38595	0.89061
C	1.67267	-1.58423	3.41848

O	0.8028	-1.46894	4.21459
O	2.6707	-1.99144	2.92052
C	0.09387	1.50297	1.1648
O	-0.15637	2.60409	1.52367
N	-1.16577	-1.21888	1.2609
H	3.12253	-0.04564	0.90351
C	2.84251	1.6523	-0.10585
C	3.47919	3.59813	0.89885
C	2.53578	3.69685	-1.28962
C	3.07218	4.36032	-0.18508
H	3.9132	4.07361	1.77535
H	2.20815	4.25023	-2.16444
H	3.17574	5.43947	-0.16845
N	3.38401	2.25928	0.95109
C	-2.40626	-0.75279	0.70001
C	-3.02726	0.35606	1.27644
C	-2.98859	-1.33032	-0.42533
C	-4.19169	0.88484	0.73989
H	-2.59104	0.81045	2.1638
C	-4.15624	-0.80371	-0.97019
H	-2.54417	-2.20281	-0.89548
C	-4.77988	0.32926	-0.41558
H	-4.64468	1.73801	1.23203
H	-4.57937	-1.29253	-1.84034
N	-5.91682	0.89536	-0.99575
C	-6.73133	1.74466	-0.14286
H	-6.18022	2.63619	0.1663
H	-7.60161	2.08053	-0.70782
H	-7.08544	1.23262	0.76538
C	-6.678	0.04923	-1.89848
H	-7.55105	0.60149	-2.24781
H	-6.0875	-0.21708	-2.77865
H	-7.02426	-0.88411	-1.42752
H	-1.18549	-1.0343	2.26044
C	2.41461	2.31723	-1.25861
H	1.98364	1.76501	-2.08763
C	0.07605	-2.87404	-0.11807
H	0.32699	-3.93817	-0.20163
H	-0.38745	-2.55982	-1.05941
C	-0.84469	-2.65808	1.06351
H	-1.75972	-3.24967	0.95481
H	-0.34222	-2.99897	1.97449
C	2.16051	-1.85394	-1.02049
C	2.88832	-0.65623	-1.03623
C	2.30714	-2.77155	-2.05352
C	3.78392	-0.39501	-2.06597
C	3.18423	-2.49414	-3.10226
H	1.73199	-3.69255	-2.04282
C	3.92426	-1.31594	-3.1041
H	4.35317	0.52989	-2.0679
H	3.28956	-3.20557	-3.91535
H	4.61337	-1.10459	-3.91561

2c-IN3R

Energy: -1608.851663 Hartree

N	1.52904	-1.80519	-1.56327
N	3.01685	-0.04685	-0.06613
Mn	0.94388	-0.6376	0.063
C	0.3347	0.66589	-0.92691
O	-0.09658	1.5628	-1.56617
H	1.93446	-2.65405	-1.16668
C	1.47626	-2.52189	2.32571
O	1.94663	-3.47064	2.96663
O	1.72193	-2.26174	1.09097
C	0.4646	0.30146	1.47164
O	0.11849	0.97757	2.37655
N	-0.8246	-1.82701	-0.12541
H	3.38804	-0.4714	0.781
C	3.17516	1.36313	0.05156
C	3.44407	3.13564	1.46573
C	3.23131	3.56364	-0.86948
C	3.39163	4.05295	0.42533
H	3.57954	3.47316	2.49097
H	3.1906	4.23998	-1.71798
H	3.47851	5.11538	0.62377
N	3.34812	1.8085	1.2988

C	-2.0428	-1.06203	-0.09518
C	-2.6171	-0.74975	1.13702
C	-2.6461	-0.57844	-1.25365
C	-3.75719	0.03748	1.21642
H	-2.16282	-1.13294	2.04927
C	-3.78956	0.21018	-1.18198
H	-2.23008	-0.81016	-2.23054
C	-4.36582	0.55696	0.05517
H	-4.17591	0.24234	2.19506
H	-4.23144	0.55696	-2.10898
N	-5.47647	1.39928	0.13082
C	-6.24815	1.35504	1.36122
H	-5.65184	1.69375	2.21201
H	-7.09708	2.0334	1.26985
H	-6.63213	0.34985	1.59501
C	-6.2776	1.53328	-1.07394
H	-7.12719	2.18329	-0.86216
H	-5.70376	1.99982	-1.87856
H	-6.66241	0.57099	-1.44606
H	-0.7916	-2.38891	0.72227
C	3.12052	2.19529	-1.06898
H	2.98121	1.78406	-2.06373
C	0.31585	-2.16871	-2.30305
H	0.51497	-2.90028	-3.09649
H	-0.07816	-1.26079	-2.77312
C	-0.64739	-2.74611	-1.28906
H	-1.61357	-2.98516	-1.74361
H	-0.22888	-3.67632	-0.89335
H	0.77106	-1.81664	2.82902
C	3.61756	-0.69125	-1.24291
C	2.85695	-1.55861	-2.02753
C	4.94788	-0.43552	-1.57517
C	3.42681	-2.17057	-3.14377
C	5.51778	-1.04682	-2.69228
H	5.54745	0.2482	-0.95697
C	4.75755	-1.91435	-3.47649
H	2.82754	-2.85473	-3.76191
H	6.56658	-0.84468	-2.95425
H	5.20663	-2.39694	-4.35668

2c-IN4

Energy: -1609.980575 Hartree

H	0.86179	0.40567	2.18694
N	1.69976	-1.84522	1.13786
N	2.78288	0.41831	0.36308
Mn	0.64697	-0.06863	0.53085
C	0.50516	-0.41669	-1.19414
O	0.38474	-0.59237	-2.35517
H	2.1514	-1.5887	2.01342
C	-1.28294	0.54387	4.15761
O	-1.4025	1.66406	3.65832
O	-0.43774	-0.37872	3.8053
C	-0.13621	1.47574	0.30006
O	-0.64242	2.53504	0.17254
N	-1.02488	-1.32058	0.94503
H	3.0848	0.49516	1.33512
C	2.94197	1.6993	-0.24201
C	3.21964	3.93298	0.13254
C	2.81879	3.18924	-2.09699
H	3.40622	4.71997	0.85939
H	2.67995	3.37849	-3.15702
C	-2.25182	-0.93205	0.29854
C	-3.05223	0.03835	0.90361
C	-2.65049	-1.4496	-0.93197
C	-4.21158	0.49324	0.29357
H	-2.75683	0.45221	1.86604
C	-3.81273	-0.99696	-1.54935
H	-2.06519	-2.21709	-1.42873
C	-4.61634	-0.00046	-0.96431
H	-4.80484	1.24103	0.80747
H	-4.09027	-1.43673	-2.50041
N	-5.74924	0.49573	-1.60989
C	-6.73039	1.17639	-0.78184
H	-6.31048	2.08296	-0.33936
H	-7.5705	1.48033	-1.40721
H	-7.1172	0.54775	0.03516

C	-6.31808	-0.33148	-2.66007
H	-7.21188	0.15671	-3.04978
H	-5.6198	-0.44436	-3.49317
H	-6.59736	-1.33849	-2.31307
H	-1.16707	-1.25339	1.95522
H	-1.93213	0.22617	4.99894
H	0.25599	0.04198	2.87524
C	2.76246	1.89116	-1.61445
H	2.57252	1.05178	-2.27505
N	3.18007	2.68459	0.62511
C	3.04613	4.24012	-1.20834
H	3.0907	5.26896	-1.54736
C	-0.53802	-2.6886	0.63995
H	-0.33753	-2.75611	-0.43351
H	-1.29559	-3.44028	0.89147
C	0.7353	-2.93139	1.41295
H	0.53207	-2.90012	2.4869
H	1.14108	-3.92216	1.19242
C	3.32131	-0.77437	-0.26007
C	2.71385	-1.98252	0.12646
C	4.33854	-0.77144	-1.20292
C	3.09155	-3.17222	-0.47881
C	4.71694	-1.97127	-1.80846
H	4.8165	0.16233	-1.48304
C	4.08717	-3.15965	-1.45862
H	2.61253	-4.10574	-0.20208
H	5.50078	-1.96683	-2.55912
H	4.3715	-4.09001	-1.94014

2c-TS4-5

Energy: -1609.972432 Hartree

H	0.86072	0.40112	2.20348
N	1.70166	-1.84521	1.13553
N	2.77792	0.42074	0.36351
Mn	0.64832	-0.07288	0.52854
C	0.50633	-0.41769	-1.19547
O	0.38554	-0.5927	-2.35665
H	2.15191	-1.58827	2.01172
C	-1.28702	0.54074	4.15629
O	-1.40567	1.66246	3.65991
O	-0.43967	-0.37972	3.80441
C	-0.13749	1.47097	0.30059
O	-0.64558	2.52948	0.17516
N	-1.02084	-1.32268	0.94261
H	3.07919	0.49795	1.33579
C	2.93464	1.70277	-0.24041
C	3.20674	3.93671	0.1364
C	2.81048	3.19389	-2.0943
H	3.39061	4.7235	0.86413
H	2.67266	3.38371	-3.15435
C	-2.24842	-0.93423	0.29701
C	-3.05032	0.03345	0.90433
C	-2.64598	-1.44913	-0.93497
C	-4.21008	0.48842	0.2951
H	-2.75585	0.44524	1.8679
C	-3.8086	-0.99645	-1.55155
H	-2.0593	-2.21433	-1.43363
C	-4.61376	-0.00253	-0.96418
H	-4.80448	1.23406	0.81079
H	-4.08522	-1.4341	-2.50385
N	-5.74706	0.49381	-1.60888
C	-6.72958	1.1708	-0.77941
H	-6.31116	2.07672	-0.3342
H	-7.56982	1.47532	-1.40434
H	-7.11591	0.53932	0.03563
C	-6.31424	-0.33121	-2.66165
H	-7.20836	0.15698	-3.05062
H	-5.6153	-0.44101	-3.4946
H	-6.59257	-1.33949	-2.31757
H	-1.16248	-1.2555	1.95295
H	-1.9396	0.22024	4.99408
H	0.25702	0.04131	2.8818
C	2.75643	1.89527	-1.61288
H	2.5693	1.05601	-2.27442
N	3.16945	2.6878	0.62784
C	3.03428	4.24452	-1.20447

H	3.07696	5.27374	-1.5426
C	-0.5346	-2.69087	0.63712
H	-0.33475	-2.75834	-0.43643
H	-1.29215	-3.44238	0.88914
C	0.73914	-2.93346	1.40935
H	0.53628	-2.90421	2.48342
H	1.14639	-3.92318	1.18697
C	3.32151	-0.76963	-0.25987
C	2.7173	-1.97974	0.12529
C	4.34015	-0.763	-1.2011
C	3.09971	-3.16815	-0.47946
C	4.72331	-1.96155	-1.80629
H	4.81559	0.17234	-1.48024
C	4.09686	-3.15204	-1.4577
H	2.62338	-4.10329	-0.2036
H	5.50828	-1.95443	-2.55573
H	4.38505	-4.08135	-1.93896

2c-IN5

Energy: -1609.971996 Hartree

H	0.86179	0.40567	2.18694
N	1.69976	-1.84522	1.13786
N	2.78288	0.41831	0.36308
Mn	0.64697	-0.06863	0.53085
C	0.50516	-0.41669	-1.19414
O	0.38474	-0.59237	-2.35517
H	2.1514	-1.5887	2.01342
C	-1.28294	0.54387	4.15761
O	-1.4025	1.66406	3.65832
O	-0.43774	-0.37872	3.8053
C	-0.13621	1.47574	0.30006
O	-0.64242	2.53504	0.17254
N	-1.02488	-1.32058	0.94503
H	3.0848	0.49516	1.33512
C	2.94197	1.6993	-0.24201
C	3.21964	3.93298	0.13254
C	2.81879	3.18924	-2.09699
H	3.40622	4.71997	0.85939
H	2.67995	3.37849	-3.15702
C	-2.25182	-0.93205	0.29854
C	-3.05223	0.03835	0.90361
C	-2.65049	-1.4496	-0.93197
C	-4.21158	0.49324	0.29357
H	-2.75683	0.45221	1.86604
C	-3.81273	-0.99696	-1.54935
H	-2.06519	-2.21709	-1.42873
C	-4.61634	-0.00046	-0.96431
H	-4.80484	1.24103	0.80747
H	-4.09027	-1.43673	-2.50041
N	-5.74924	0.49573	-1.60989
C	-6.73039	1.17639	-0.78184
H	-6.31048	2.08296	-0.33936
H	-7.5705	1.48033	-1.40721
H	-7.1172	0.54775	0.03516
C	-6.31808	-0.33148	-2.66007
H	-7.21188	0.15671	-3.04978
H	-5.6198	-0.44436	-3.49317
H	-6.59736	-1.33849	-2.31307
H	-1.16707	-1.25339	1.95522
H	-1.93213	0.22617	4.99894
H	0.25599	0.04198	2.87524
C	2.76246	1.89116	-1.61445
H	2.57252	1.05178	-2.27505
N	3.18007	2.68459	0.62511
C	3.04613	4.24012	-1.20834
H	3.0907	5.26896	-1.54736
C	-0.53802	-2.6886	0.63995
H	-0.33753	-2.75611	-0.43351
H	-1.29559	-3.44028	0.89147
C	0.7353	-2.93139	1.41295
H	0.53207	-2.90012	2.4869
H	1.14108	-3.92216	1.19242
C	3.32131	-0.77437	-0.26007
C	2.71385	-1.98252	0.12646
C	4.33854	-0.77144	-1.20292
C	3.09155	-3.17222	-0.47881

C	4.71694	-1.97127	-1.80846
H	4.8165	0.16233	-1.48304
C	4.08717	-3.15965	-1.45862
H	2.61253	-4.10574	-0.20208
H	5.50078	-1.96683	-2.55912
H	4.3715	-4.09001	-1.94014

2d-IN1

Energy: -1436.296544 Hartree

N	-1.87684	2.53565	0.08749
N	0.52619	1.96642	-0.8754
Mn	-1.16222	1.03385	-0.8211
H	-2.41585	3.07726	-0.55704
C	-0.5622	-0.43996	-1.91675
O	-0.1694	-1.40473	-2.63397
N	-2.99782	0.37705	-0.61281
C	1.74545	1.13825	-0.47558
C	3.57088	-0.38395	-1.14544
C	3.35505	0.23156	1.18144
H	4.09903	-0.89268	-1.92372
H	3.71907	0.19135	2.19028
C	-3.02262	-0.96525	0.00792
C	-3.09031	-3.2635	-0.44702
C	-3.26251	-2.44005	1.80016
C	-3.22301	-3.52159	0.91144
H	-3.07632	-4.06516	-1.1574
H	-3.39174	-2.6174	2.84714
H	-3.45102	0.25714	-1.49714
C	-0.62869	-0.08012	0.67684
O	-0.22825	-0.78835	1.64144
H	0.63665	2.26514	-1.8139
H	-1.60222	1.62449	-2.0974
C	2.22723	1.05005	0.8618
H	1.74846	1.60673	1.64122
C	3.99826	-0.52954	0.18522
C	2.4505	0.42444	-1.46961
H	2.14014	0.50073	-2.48822
N	5.10521	-1.48794	0.52551
C	6.0489	-1.61483	-0.6041
H	6.83805	-2.29085	-0.33474
H	5.53306	-1.99575	-1.46009
H	6.46606	-0.65805	-0.83468
C	5.85382	-1.02914	1.7099
H	5.19586	-0.98111	2.55105
H	6.64815	-1.72061	1.91829
H	6.26707	-0.06159	1.52265
H	-3.29365	-4.52913	1.26601
N	-2.98073	-2.00144	-0.85417
N	-3.13775	-1.17899	1.33213
C	-2.78505	2.02203	1.11451
H	-3.24987	2.79368	1.69459
H	-2.33257	1.332	1.78926
C	-3.77427	1.37153	0.17834
H	-4.62131	0.91253	0.6616
H	-4.12602	2.15105	-0.46213
C	0.42691	3.13471	0.01927
C	-0.81066	3.40007	0.55728
C	1.51238	3.96014	0.30688
C	-1.00339	4.44931	1.46239
C	1.31013	5.10956	1.09143
H	2.48191	3.733	-0.07974
C	0.0569	5.34728	1.68237
H	-1.93878	4.58373	1.96068
H	2.11138	5.80775	1.23696
H	-0.09044	6.21492	2.29527

2d-IN2

Energy: -1624.870750 Hartree

N	1.77693	1.81774	0.303
N	-0.81133	1.26204	0.32579
Mn	0.84478	-0.10864	0.48977
H	1.72545	2.29478	1.20281
C	-0.0176	-1.58425	0.81955
O	-0.65216	-2.55674	1.05133
N	2.89702	-0.69144	0.95235
C	-2.08554	0.62078	0.15594
C	-4.13635	-0.20176	1.13995
C	-3.61376	-0.71362	-1.1546
H	-4.78527	-0.25178	2.00668
H	-3.85034	-1.17458	-2.1066
C	3.28086	-1.7173	0.05988
C	3.17926	-3.91087	-0.46899
C	4.1774	-2.34131	-1.91559

C	3.79543	-3.65431	-1.68803
H	2.8907	-4.92165	-0.18914
H	4.70544	-2.06005	-2.82441
H	2.81035	-1.0778	1.88778
C	0.91887	-0.49415	-1.23742
O	0.92145	-0.84321	-2.36916
H	-0.774	1.67462	1.26009
H	0.70303	0.15237	2.15734
C	-2.42748	-0.00365	-1.0436
H	-1.75959	0.06268	-1.89919
C	-4.4929	-0.85043	-0.0584
C	-2.95153	0.51852	1.24036
H	-2.69351	1.00432	2.17969
N	-5.65064	-1.62019	-0.15244
C	-6.67758	-1.38847	0.84864
H	-7.53357	-2.02791	0.63082
H	-6.31987	-1.65171	1.84738
H	-7.02425	-0.34393	0.87731
C	-6.15556	-1.89183	-1.48764
H	-5.44638	-2.49255	-2.06251
H	-7.07716	-2.46869	-1.40552
H	-6.36969	-0.97744	-2.06208
H	3.98925	-4.44022	-2.40715
N	2.91805	-2.95398	0.42611
N	3.9377	-1.35346	-1.04325
C	3.19219	1.57344	-0.01916
H	3.79784	2.48541	0.04265
H	3.24077	1.19382	-1.04368
C	3.71168	0.54975	0.96532
H	4.76173	0.32113	0.76385
H	3.63918	0.94992	1.98105
C	-0.3896	2.2545	-0.64116
C	0.98336	2.54121	-0.65466
C	-1.24221	2.8895	-1.53481
C	1.50167	3.4421	-1.57511
C	-0.71773	3.79087	-2.46245
H	-2.30452	2.66575	-1.52009
C	0.64641	4.06133	-2.48827
H	2.56754	3.64966	-1.58687
H	-1.38383	4.2769	-3.16856
H	1.05201	4.75606	-3.21714
C	0.88739	1.36236	3.54254
O	0.1183	2.20021	3.20276
O	1.69303	0.79691	4.19963

2d-TS2-3

Energy: -1624.865839 Hartree

N	1.84058	1.82009	1.02176
N	-0.76447	1.28171	0.90015
Mn	0.90435	-0.102	0.92721
H	1.76775	2.1253	1.99119
C	0.03061	-1.60243	1.04555
O	-0.61549	-2.59322	1.12756
N	2.9608	-0.76307	1.26417
C	-2.01358	0.66345	0.54864
C	-4.098	-0.34663	1.24859
C	-3.44315	-0.42695	-1.06611
H	-4.79026	-0.56089	2.05472
H	-3.61862	-0.70654	-2.09855
C	3.344	-1.61894	0.20876
C	3.23607	-3.68755	-0.69308
C	4.25022	-1.8968	-1.84056
C	3.86172	-3.2274	-1.84585
H	2.94116	-4.72993	-0.59407
H	4.78453	-1.4648	-2.68425
H	2.87315	-1.30523	2.11748
C	0.99137	-0.24489	-0.84124
O	1.02029	-0.43937	-2.01
H	-0.79637	1.54478	1.88422
H	0.81753	-0.0103	2.64327
C	-2.27937	0.26377	-0.76125
H	-1.57232	0.49612	-1.55347
C	-4.3777	-0.76926	-0.06542
C	-2.93565	0.35615	1.54469
H	-2.74165	0.66387	2.57008
N	-5.51631	-1.51727	-0.36238
C	-6.60837	-1.45488	0.59407
H	-7.44165	-2.04912	0.21803
H	-6.31376	-1.88003	1.55666
H	-6.96704	-0.42928	0.77215
C	-5.93563	-1.55314	-1.75316
H	-5.18242	-2.03855	-2.37854
H	-6.85007	-2.14206	-1.8302
H	-6.13116	-0.5536	-2.17129

H	4.0577	-3.87823	-2.68871
N	2.97228	-2.89848	0.35206
N	4.00987	-1.0739	-0.81132
C	3.25979	1.63163	0.68554
H	3.86283	2.52042	0.90746
H	3.32442	1.42951	-0.38757
C	3.77256	0.45754	1.48916
H	4.8244	0.26761	1.25787
H	3.6936	0.68241	2.55642
C	-0.30924	2.41567	0.1209
C	1.06466	2.69054	0.18191
C	-1.14081	3.19828	-0.67051
C	1.60336	3.7273	-0.57016
C	-0.59629	4.23631	-1.42716
H	-2.20423	2.98324	-0.71194
C	0.76976	4.49534	-1.38404
H	2.67002	3.92699	-0.52834
H	-1.24693	4.8376	-2.05456
H	1.1934	5.2969	-1.98112
C	1.0593	0.38581	3.9191
O	0.06588	1.20894	4.08928
O	2.13221	-0.30709	4.16839

2d-IN3

Energy: -1624.885398 Hartree

N	1.77693	1.81774	0.303
N	-0.81133	1.26204	0.32579
Mn	0.84478	-0.10864	0.48977
H	1.72545	2.29478	1.20281
C	-0.0176	-1.58425	0.81955
O	-0.65216	-2.55674	1.05133
N	2.89702	-0.69144	0.95235
C	-2.08554	0.62078	0.15594
C	-4.13635	-0.20176	1.13995
C	-3.61376	-0.71362	-1.1546
H	-4.78527	-0.25178	2.00668
H	-3.85034	-1.17458	-2.1066
C	3.28086	-1.7173	0.05988
C	3.17926	-3.91087	-0.46899
C	4.1774	-2.34131	-1.91559
C	3.79543	-3.65431	-1.68803
H	2.8907	-4.92165	-0.18914
H	4.70544	-2.06005	-2.82441
H	2.81035	-1.0778	1.88778
C	0.91887	-0.49415	-1.23742
O	0.92145	-0.84321	-2.36916
H	-0.774	1.67462	1.26009
H	0.70303	0.15237	2.15734
C	-2.42748	-0.00365	-1.0436
H	-1.75959	0.06268	-1.89919
C	-4.4929	-0.85043	-0.0584
C	-2.95153	0.51852	1.24036
H	-2.69351	1.00432	2.17969
N	-5.65064	-1.62019	-0.15244
C	-6.67758	-1.38847	0.84864
H	-7.53357	-2.02791	0.63082
H	-6.31987	-1.65171	1.84738
H	-7.02425	-0.34393	0.87731
C	-6.15556	-1.89183	-1.48764
H	-5.44638	-2.49255	-2.06251
H	-7.07716	-2.46869	-1.40552
H	-6.36969	-0.97744	-2.06208
H	3.98925	-4.44022	-2.40715
N	2.91805	-2.95398	0.42611
N	3.9377	-1.35346	-1.04325
C	3.19219	1.57344	-0.01916
H	3.79784	2.48541	0.04265
H	3.24077	1.19382	-1.04368
C	3.71168	0.54975	0.96532
H	4.76173	0.32113	0.76385
H	3.63918	0.94992	1.98105
C	-0.3896	2.2545	-0.64116
C	0.98336	2.54121	-0.65466
C	-1.24221	2.8895	-1.53481
C	1.50167	3.4421	-1.57511
C	-0.71773	3.79087	-2.46245
H	-2.30452	2.66575	-1.52009
C	0.64641	4.06133	-2.48827
H	2.56754	3.64966	-1.58687
H	-1.38383	4.2769	-3.16856
H	1.05201	4.75606	-3.21714
C	0.88739	1.36236	3.54254
O	0.1183	2.20021	3.20276
O	1.69303	0.79691	4.19963

2d-IN3R

Energy: -1624.913941 Hartree

N	-1.87684	2.53565	0.08749
N	0.52619	1.96642	-0.8754
Mn	-1.16222	1.03385	-0.8211
H	-2.41585	3.07726	-0.55704
C	-0.5622	-0.43996	-1.91675
O	-0.1694	-1.40473	-2.63397
N	-2.99782	0.37705	-0.61281
C	1.74545	1.13825	-0.47558
C	3.57088	-0.38395	-1.14544
C	3.35505	0.23156	1.18144
H	4.09903	-0.89268	-1.92372
H	3.71907	0.19135	2.19028
C	-3.02262	-0.96525	0.00792
C	-3.09031	-3.2635	-0.44702
C	-3.26251	-2.44005	1.80016
C	-3.22301	-3.52159	0.91144
H	-3.07632	-4.06516	-1.1574
H	-3.39174	-2.6174	2.84714
H	-3.45102	0.25714	-1.49714
C	-0.62869	-0.08012	0.67684
O	-0.22825	-0.78835	1.64144
H	0.63665	2.26514	-1.8139
C	2.22723	1.05005	0.8618
H	1.74846	1.60673	1.64122
C	3.99826	-0.52954	0.18522
C	2.4505	0.42444	-1.46961
H	2.14014	0.50073	-2.48822
N	5.10521	-1.48794	0.52551
C	6.0489	-1.61483	-0.6041
H	6.83805	-2.29085	-0.33474
H	5.53306	-1.99575	-1.46009
H	6.46606	-0.65805	-0.83468
C	5.85382	-1.02914	1.7099
H	5.19586	-0.98111	2.55105
H	6.64815	-1.72061	1.91829
H	6.26707	-0.06159	1.52265
H	-3.29365	-4.52913	1.26601
N	-2.98073	-2.00144	-0.85417
N	-3.13775	-1.17899	1.33213
C	-2.78505	2.02203	1.11451
H	-3.24987	2.79368	1.69459
H	-2.33257	1.332	1.78926
C	-3.77427	1.37153	0.17834
H	-4.62131	0.91253	0.6616
H	-4.12602	2.15105	-0.46213
C	0.42691	3.13471	0.01927
C	-0.81066	3.40007	0.55728
C	1.51238	3.96014	0.30688
C	-1.00339	4.44931	1.46239
C	1.31013	5.10956	1.09143
H	2.48191	3.733	-0.07974
C	0.0569	5.34728	1.68237
H	-1.93878	4.58373	1.96068
H	2.11138	5.80775	1.23696
H	-0.09044	6.21492	2.29527
O	-1.70865	1.76736	-2.40612
C	-2.2864	0.82574	-3.19414
O	-2.35616	-0.32133	-2.75491
H	-2.62677	1.23874	-4.15874

2d-IN4

Energy: -1626.045253 Hartree

H	0.86851	0.49003	2.07922
N	1.85161	1.57832	-0.28533
N	-0.77082	1.33794	0.10434
Mn	0.78248	-0.10919	0.44555
C	0.62095	-0.87143	-1.13641
O	0.47958	-1.45004	-2.15587
H	1.96442	2.22689	0.49864
C	2.56634	2.35999	3.40229
O	3.21189	3.27198	3.91073
O	1.59113	2.52138	2.55279
C	-0.1702	-1.39743	1.15033
O	-0.85744	-2.24124	1.60705
N	2.80203	-0.82721	0.81634
H	-0.66477	1.94952	0.91479
C	-2.09582	0.77434	0.14078
C	-4.10611	0.29352	1.39585
C	-3.80853	-0.64336	-0.80228
H	-4.68309	0.44396	2.30095
H	-4.15108	-1.24059	-1.6395

C	2.95926	-2.07154	0.15983
C	2.56888	-4.29518	0.18633
C	3.51135	-3.23269	-1.69462
C	3.03686	-4.40051	-1.11806
H	2.22111	-5.17097	0.72957
H	3.93388	-3.2326	-2.69712
H	2.81614	-0.98103	1.82102
H	2.78324	1.29674	3.65033
H	1.1936	1.41437	2.2428
C	-2.57425	-0.01843	-0.90212
H	-1.98111	-0.14804	-1.80381
C	-4.602	-0.52042	0.35874
H	3.05255	-5.34631	-1.64489
C	-2.87371	0.92643	1.28433
H	-2.51046	1.54362	2.10357
N	2.52688	-3.13505	0.84841
N	3.49098	-2.05132	-1.06295
N	-5.81318	-1.19762	0.48165
C	-6.73218	-0.72067	1.50062
H	-7.64271	-1.31925	1.46274
H	-6.30593	-0.83852	2.49998
H	-7.00747	0.3375	1.37188
C	-6.44912	-1.63982	-0.74808
H	-5.83805	-2.38612	-1.26148
H	-7.40045	-2.11257	-0.50257
H	-6.64323	-0.81672	-1.45277
C	3.73516	0.26567	0.43631
H	4.71398	-0.14234	0.17115
H	3.85801	0.88697	1.32792
C	3.18511	1.10426	-0.69507
H	3.87219	1.93211	-0.90577
H	3.06764	0.51148	-1.60672
C	-0.36438	2.07157	-1.08188
C	1.01726	2.18591	-1.28785
C	-1.25396	2.62961	-1.99094
C	1.50399	2.8323	-2.4172
C	-0.76246	3.27571	-3.12539
H	-2.32314	2.54129	-1.82399
C	0.60854	3.3699	-3.34218
H	2.57538	2.90744	-2.57722
H	-1.45909	3.70021	-3.84151
H	0.98839	3.86437	-4.23075

2d-TS4-5

Energy: -1626.033195 Hartree

H	1.63001	1.41203	1.43116
N	1.64709	1.50038	-1.31843
N	-0.6669	1.86609	0.17306
Mn	0.86985	0.36297	0.26974
C	-0.01623	-0.76496	-0.75362
O	-0.65667	-1.5533	-1.35714
H	2.24107	2.24245	-0.94522
C	4.24617	2.51414	1.37801
O	5.32569	3.09135	1.27633
O	3.11529	2.95978	0.91195
C	0.28187	-0.4943	1.68077
O	-0.12631	-1.07455	2.62579
N	2.81485	-0.61883	-0.00012
H	-0.5654	2.35151	1.06001
C	-1.99989	1.32333	0.12709
C	-3.88848	0.39435	1.32746
C	-3.93988	0.51671	-1.07288
H	-4.34179	0.1531	2.28216
H	-4.43229	0.36758	-2.027
C	2.57442	-2.01109	-0.03744
C	2.1953	-3.90545	1.13134
C	2.03456	-3.8554	-1.2199
C	1.89673	-4.58073	-0.04601
H	2.16422	-4.41384	2.09247
H	1.86797	-4.32003	-2.18955
H	3.31358	-0.39625	0.85704
H	4.16993	1.53346	1.90024
H	2.2746	2.115	1.14557
C	-2.6749	1.09582	-1.07001
H	-2.22477	1.36642	-2.0211
C	-4.57458	0.13239	0.12344
H	1.60179	-5.62254	-0.04821
C	-2.62708	0.972	1.32261
H	-2.12005	1.16101	2.2667
N	2.54802	-2.61669	1.15648
N	2.38546	-2.56302	-1.23722
N	-5.81848	-0.50087	0.11834
C	-6.57726	-0.46213	1.35711
H	-7.53388	-0.96178	1.20005

H	-6.05738	-0.99916	2.15417
H	-6.77554	0.56162	1.71073
C	-6.62346	-0.34682	-1.08144
H	-6.13802	-0.81167	-1.94317
H	-7.5777	-0.85356	-0.93372
H	-6.82592	0.70582	-1.33338
C	3.49398	-0.01526	-1.1793
H	4.10593	-0.76268	-1.68936
H	4.16275	0.75744	-0.78845
C	2.49306	0.61104	-2.12317
H	3.01515	1.14983	-2.92425
H	1.85132	-0.14813	-2.58203
C	-0.31064	2.8029	-0.90228
C	0.80829	2.55766	-1.69873
C	-1.09123	3.9369	-1.12648
C	1.14685	3.44663	-2.7187
C	-0.75327	4.82572	-2.14735
H	-1.97325	4.13025	-0.49896
C	0.36565	4.58086	-2.94335
H	2.02914	3.25373	-3.34611
H	-1.36915	5.71953	-2.32416
H	0.63276	5.2816	-3.74765

2d-IN5

Energy: -1626.033131 Hartree

H	0.86851	0.49003	2.07922
N	1.85161	1.57832	-0.28533
N	-0.77082	1.33794	0.10434
Mn	0.78248	-0.10919	0.44555
C	0.62095	-0.87143	-1.13641
O	0.47958	-1.45004	-2.15587
H	1.96442	2.22689	0.49864
C	2.56634	2.35999	3.40229
O	3.21189	3.27198	3.91073
O	1.59113	2.52138	2.55279
C	-0.1702	-1.39743	1.15033
O	-0.85744	-2.24124	1.60705
N	2.80203	-0.82721	0.81634
H	-0.66477	1.94952	0.91479
C	-2.09582	0.77434	0.14078
C	-4.10611	0.29352	1.39585
C	-3.80853	-0.64336	-0.80228
H	-4.68309	0.44396	2.30095
H	-4.15108	-1.24059	-1.6395
C	2.95926	-2.07154	0.15983
C	2.56888	-4.29518	0.18633
C	3.51135	-3.23269	-1.69462
C	3.03686	-4.40051	-1.11806
H	2.22111	-5.17097	0.72957
H	3.93388	-3.2326	-2.69712
H	2.81614	-0.98103	1.82102
H	2.78324	1.29674	3.65033
H	1.1936	1.41437	2.2428
C	-2.57425	-0.01843	-0.90212
H	-1.98111	-0.14804	-1.80381
C	-4.602	-0.52042	0.35874
H	3.05255	-5.34631	-1.64489
C	-2.87371	0.92643	1.28433
H	-2.51046	1.54362	2.10357
N	2.52688	-3.13505	0.84841
N	3.49098	-2.05132	-1.06295
N	-5.81318	-1.19762	0.48165
C	-6.73218	-0.72067	1.50062
H	-7.64271	-1.31925	1.46274
H	-6.30593	-0.83852	2.49998
H	-7.00747	0.3375	1.37188
C	-6.44912	-1.63982	-0.74808
H	-5.83805	-2.38612	-1.26148
H	-7.40045	-2.11257	-0.50257
H	-6.64323	-0.81672	-1.45277
C	3.73516	0.26567	0.43631
H	4.71398	-0.14234	0.17115
H	3.85801	0.88697	1.32792
C	3.18511	1.10426	-0.69507
H	3.87219	1.93211	-0.90577
H	3.06764	0.51148	-1.60672
C	-0.36438	2.07157	-1.08188
C	1.01726	2.18591	-1.28785
C	-1.25396	2.62961	-1.99094
C	1.50399	2.8323	-2.4172
C	-0.76246	3.27571	-3.12539
H	-2.32314	2.54129	-1.82399
C	0.60854	3.3699	-3.34218
H	2.57538	2.90744	-2.57722

H	-1.45909	3.70021	-3.84151
H	0.98839	3.86437	-4.23075

2e-IN1

Energy: -1334.521952 Hartree

N	-0.00018	2.4975	0.13064
N	2.16267	0.98283	-0.73208
Mn	-0.00021	0.60747	-0.81194
H	0.00004	3.21491	-0.59139
C	-0.00022	-0.80943	-1.83005
O	-0.00048	-1.75689	-2.54197
N	-2.16267	0.98265	-0.73223
C	2.78159	-0.13568	-0.13089
C	3.54133	-2.24896	-0.37571
C	3.49617	-1.19456	1.73093
H	3.76949	-3.08362	-1.03503
H	3.683	-1.16194	2.8024
C	-2.78127	-0.13603	-0.13081
C	-3.54068	-2.24946	-0.37532
C	-3.49573	-1.19474	1.73116
C	-3.76143	-2.3384	0.99388
H	-3.76877	-3.0842	-1.03457
H	-3.68274	-1.16195	2.80258
H	-2.42074	1.01398	-1.71318
C	0.00002	-0.39818	0.65602
O	0.00005	-1.18141	1.5457
H	2.42029	1.01429	-1.71313
H	-0.00042	1.48232	-2.24165
C	3.7622	-2.33803	0.99349
H	-4.14485	-3.24051	1.45394
N	-3.05722	-1.15029	-0.96123
N	-3.01245	-0.07295	1.18218
C	-1.25116	2.61262	0.88422
H	-1.3891	3.61388	1.31338
H	-1.22259	1.88664	1.70302
C	-2.37239	2.3006	-0.08014
H	-3.34477	2.33156	0.41778
H	-2.37731	3.04219	-0.88428
H	4.14598	-3.24006	1.45339
N	3.01261	-0.07279	1.18214
N	3.05767	-1.14982	-0.96147
C	2.36983	2.28717	-0.08656
C	1.28186	2.98841	0.43407
C	3.65403	2.82371	0.00557
C	1.47807	4.22623	1.04608
C	3.85059	4.06143	0.61861
H	4.51166	2.27107	-0.40456
C	2.76288	4.76279	1.13871
H	0.62049	4.7793	1.45591
H	4.8633	4.48403	0.69147
H	2.91734	5.73875	1.62135

2e-IN2

Energy: -1523.094923 Hartree

H	-0.03727	0.22647	2.11574
N	0.00531	-1.93637	0.5387
N	1.90599	-0.09964	0.37575
Mn	-0.25063	0.17609	0.44977
C	-0.46829	0.23424	-1.31206
O	-0.63321	0.37355	-2.47403
H	0.27479	-2.16771	1.49613
C	-0.00176	-0.70649	3.67672
O	-0.94151	-0.23541	4.22356
O	0.95045	-1.39652	3.51016
C	-0.36026	1.91502	0.55481
O	-0.38789	3.09516	0.62915
N	-2.29203	-0.36484	0.9049
H	2.11752	-0.27363	1.3584
C	-3.13224	0.17741	-0.09641
C	-4.19465	2.00063	-0.89951
C	-4.1382	-0.02317	-2.10625
C	-4.52513	1.30726	-2.05767
H	-4.50213	3.03437	-0.75808
H	-4.39501	-0.65188	-2.95624
H	-2.46348	0.12224	1.7801
H	-5.07665	1.77302	-2.8647
N	-3.44588	-0.61375	-1.12325
N	-3.50156	1.44908	0.10117
C	-2.33086	-1.83683	1.10553
H	-3.33275	-2.22404	0.90236
H	-2.11066	-2.00683	2.16397
C	-1.30412	-2.54389	0.25068
H	-1.32075	-3.61873	0.46588
H	-1.51274	-2.40017	-0.8133

C	2.4851	1.13872	0.00534
N	2.90732	1.87667	1.03761
N	2.49193	1.45737	-1.28998
C	3.32315	3.10906	0.72126
C	2.93072	2.69089	-1.56552
C	3.34605	3.58034	-0.58333
H	3.65938	3.72883	1.54907
H	2.94678	2.9692	-2.61695
H	3.68482	4.58003	-0.82452
C	2.10975	-1.28784	-0.4244
C	1.09844	-2.25269	-0.33998
C	3.22432	-1.50486	-1.22343
C	1.18379	-3.41839	-1.09123
C	3.30425	-2.67351	-1.98028
H	4.01266	-0.76077	-1.26943
C	2.28564	-3.61998	-1.92236
H	0.39221	-4.15933	-1.03212
H	4.16708	-2.83822	-2.61781
H	2.34764	-4.52455	-2.51925

2e-TS2-3

Energy: -1523.091822 Hartree

H	0.1182	2.25413	-0.24796
N	-0.02826	1.29006	2.09878
N	2.12595	0.69951	0.46547
Mn	-0.01933	0.594	0.10786
C	-0.15819	-1.13032	0.50224
O	-0.24201	-2.29797	0.66922
H	0.04557	2.30557	2.08356
C	0.66425	3.36913	-1.56587
O	-0.10141	4.26673	-1.43812
O	1.58713	2.77378	-2.02217
C	-0.00092	0.2092	-1.59999
O	0.01341	-0.04063	-2.75646
N	-2.15246	1.01334	0.34296
H	2.40805	1.42134	-0.19509
C	-2.86934	-0.13041	-0.07667
C	-3.70161	-1.3252	-1.80272
C	-3.75992	-2.14703	0.40552
C	-4.02072	-2.36568	-0.93882
H	-3.91906	-1.39311	-2.86638
H	-4.02092	-2.89022	1.15603
H	-2.30928	1.76285	-0.32497
H	-4.47331	-3.2836	-1.2924
N	-3.19094	-1.02363	0.86079
N	-3.13092	-0.18982	-1.38842
C	-2.38143	1.48194	1.73627
H	-3.38738	1.2143	2.06813
H	-2.3118	2.57294	1.70591
C	-1.32891	0.93725	2.67521
H	-1.46649	1.35662	3.68033
H	-1.3863	-0.15369	2.74604
C	2.70052	-0.53231	0.08115
N	3.01322	-0.63427	-1.21729
N	2.85434	-1.45595	1.03196
C	3.44418	-1.83834	-1.60477
C	3.28731	-2.6484	0.60297
C	3.57871	-2.90786	-0.72768
H	3.70034	-1.94097	-2.65702
H	3.41021	-3.41597	1.3643
H	3.91907	-3.8805	-1.06023
C	2.36566	0.96471	1.89134
C	1.28843	1.17826	2.75183
C	3.67001	1.00276	2.38407
C	1.51562	1.43046	4.10461
C	3.89739	1.25404	3.73742
H	4.51914	0.83429	1.70604
C	2.82049	1.46802	4.59768
H	0.66662	1.59941	4.7828
H	4.9259	1.28357	4.12572
H	2.99937	1.66682	5.66434

2e-IN3

Energy: -1523.109973 Hartree

H	-0.03727	0.22647	2.11574
N	0.00531	-1.93637	0.5387
N	1.90599	-0.09964	0.37575
Mn	-0.25063	0.17609	0.44977

C	-0.46829	0.23424	-1.31206
O	-0.63321	0.37355	-2.47403
H	0.27479	-2.16771	1.49613
C	-0.00176	-0.70649	3.67672
O	-0.94151	-0.23541	4.22356
O	0.95045	-1.39652	3.51016
C	-0.36026	1.91502	0.55481
O	-0.38789	3.09516	0.62915
N	-2.29203	-0.36484	0.9049
H	2.11752	-0.27363	1.3584
C	-3.13224	0.17741	-0.09641
C	-4.19465	2.00063	-0.89951
C	-4.1382	-0.02317	-2.10625
C	-4.52513	1.30726	-2.05767
H	-4.50213	3.03437	-0.75808
H	-4.39501	-0.65188	-2.95624
H	-2.46348	0.12224	1.7801
H	-5.07665	1.77302	-2.8647
N	-3.44588	-0.61375	-1.12325
N	-3.50156	1.44908	0.10117
C	-2.33086	-1.83683	1.10553
H	-3.33275	-2.22404	0.90236
H	-2.11066	-2.00683	2.16397
C	-1.30412	-2.54389	0.25068
H	-1.32075	-3.61873	0.46588
H	-1.51274	-2.40017	-0.8133
C	2.4851	1.13872	0.00534
N	2.90732	1.87667	1.03761
N	2.49193	1.45737	-1.28998
C	3.32315	3.10906	0.72126
C	2.93072	2.69089	-1.56552
C	3.34605	3.58034	-0.58333
H	3.65938	3.72883	1.54907
H	2.94678	2.9692	-2.61695
H	3.68482	4.58003	-0.82452
C	2.10975	-1.28784	-0.4244
C	1.09844	-2.25269	-0.33998
C	3.22432	-1.50486	-1.22343
C	1.18379	-3.41839	-1.09123
C	3.30425	-2.67351	-1.98028
H	4.01266	-0.76077	-1.26943
C	2.28564	-3.61998	-1.92236
H	0.39221	-4.15933	-1.03212
H	4.16708	-2.83822	-2.61781
H	2.34764	-4.52455	-2.51925

2e-IN3R

Energy: -1523.133607 Hartree

N	-0.00257	1.6272	-1.7171
N	-2.13976	0.83237	-0.17516
Mn	-0.00042	0.56712	0.07454
C	0.04167	-1.01919	-0.67084
O	0.06669	-2.11556	-1.10884
H	-0.03046	2.61137	-1.44685
C	-0.31297	2.79096	2.04448
O	-0.30109	3.93097	2.525
O	-0.02108	2.49264	0.82761
C	0.00181	-0.26027	1.63444
O	0.00783	-0.87265	2.64145
N	2.12827	0.9284	-0.12389
H	-2.38121	1.42274	0.61719
C	2.8225	-0.2922	0.04753
C	3.64465	-1.82424	1.48726
C	3.64689	-2.195	-0.84086
C	3.92394	-2.67963	0.42842
H	3.87379	-2.10677	2.51232
H	3.87381	-2.78222	-1.72815
H	2.30521	1.52715	0.67901
H	4.35676	-3.65979	0.58478
N	3.10109	-0.99035	-1.05388
N	3.0989	-0.61625	1.31582
C	2.3602	1.66637	-1.39732
H	3.34761	1.42915	-1.79926
H	2.34323	2.72891	-1.14032
C	1.26918	1.37357	-2.40166
H	1.3942	2.00336	-3.29148

H	1.28657	0.3259	-2.7183
H	-0.60226	1.92709	2.68968
C	-2.7759	-0.42189	-0.01722
N	-3.06143	-0.75802	1.24608
N	-2.99709	-1.1334	-1.12263
C	-3.54859	-1.99195	1.40864
C	-3.4855	-2.36418	-0.91856
C	-3.7627	-2.86125	0.34579
H	-3.78398	-2.28487	2.42934
H	-3.66495	-2.96254	-1.80923
H	-4.14812	-3.86204	0.4952
C	-2.36958	1.54403	-1.44071
C	-1.28687	1.91866	-2.23686
C	-3.67002	1.84468	-1.84568
C	-1.50461	2.59438	-3.43737
C	-3.88806	2.51973	-3.047
H	-4.52349	1.54932	-1.21839
C	-2.80563	2.89472	-3.84278
H	-0.65123	2.89026	-4.06465
H	-4.91354	2.75627	-3.36624
H	-2.97707	3.42745	-4.78941

2e-IN4

Energy: -1524.26897 Hartree

H	-0.15019	-0.64948	2.05973
N	-0.03228	-1.84287	-0.46977
N	1.90489	-0.2032	0.34217
Mn	-0.24939	0.05221	0.4706
C	-0.3803	0.92874	-1.05945
O	-0.4893	1.58181	-2.03394
H	0.15206	-2.52835	0.26839
C	-0.63463	-3.28223	3.03236
O	-0.70874	-4.44921	3.40758
O	0.35292	-2.78053	2.34717
C	-0.35017	1.577	1.3326
O	-0.37153	2.614	1.89307
N	-2.32425	-0.56521	0.52224
H	2.1084	-0.8066	1.13964
H	-2.53869	-0.57892	1.51593
H	-1.44204	-2.55308	3.26974
H	0.08544	-1.60183	2.13253
C	-2.39849	-1.93653	-0.04881
H	-3.39089	-2.12189	-0.46711
H	-2.24991	-2.62542	0.78736
C	-1.32946	-2.16568	-1.09164
H	-1.37338	-3.20357	-1.44102
H	-1.46602	-1.504	-1.95193
C	-3.11112	0.43662	-0.09876
N	-3.38693	0.27706	-1.3928
N	-3.46819	1.45016	0.69829
C	-4.02868	1.30301	-1.96816
C	-4.10991	2.4517	0.08885
C	-4.40069	2.44139	-1.27006
H	-4.25516	1.19379	-3.02657
H	-4.40737	3.28615	0.71985
H	-4.91126	3.26623	-1.75098
C	2.50709	1.06041	0.58088
N	2.90719	1.24861	1.8417
N	2.54798	1.92379	-0.43391
C	3.34418	2.48375	2.11863
C	3.00538	3.14151	-0.12039
C	3.40556	3.48786	1.16342
H	3.6641	2.66075	3.14259
H	3.04883	3.86096	-0.93509
H	3.76029	4.4827	1.40164
C	2.14045	-0.90137	-0.90618
C	1.11706	-1.75625	-1.33171
C	3.30556	-0.77085	-1.65041
C	1.24458	-2.44909	-2.53012
C	3.42814	-1.46426	-2.85334
H	4.10143	-0.12006	-1.30295
C	2.39921	-2.29092	-3.29546
H	0.44522	-3.10305	-2.86502
H	4.3312	-1.35227	-3.44483
H	2.49426	-2.82295	-4.23682

2e-TS4-5

Energy: -1524.259465 Hartree

H	0.61576	1.76318	-1.22972
N	0.45004	1.63356	1.38105
N	2.07433	-0.23575	0.18445
Mn	0.0383	0.4242	-0.28118
C	-0.73141	-0.89829	0.60063
O	-1.29038	-1.81228	1.095
H	0.95201	2.46219	1.06207
C	3.10145	3.13378	-0.97319
O	4.23128	3.61699	-0.93446
O	2.77759	2.03603	-1.5894
C	-0.21588	-0.4531	-1.78314
O	-0.38556	-1.04002	-2.79186
N	-1.76467	1.65132	-0.3001
H	2.61875	0.09591	-0.61011
H	-1.71346	2.11268	-1.20383
H	2.25088	3.64	-0.46088
H	1.57427	1.83343	-1.37906
C	-1.64379	2.6383	0.80551
H	-2.63258	2.9606	1.14092
H	-1.12554	3.50509	0.38462
C	-0.83257	2.06964	1.94571
H	-0.70172	2.82233	2.73308
H	-1.32479	1.19549	2.38319
C	-2.90315	0.80858	-0.28707
N	-3.45952	0.55453	0.89739
N	-3.27684	0.34361	-1.48502
C	-4.4633	-0.33285	0.87689
C	-4.28344	-0.53514	-1.46451
C	-4.91024	-0.93311	-0.28977
H	-4.92438	-0.55721	1.8364
H	-4.60106	-0.92436	-2.4292
H	-5.71834	-1.6539	-0.28944
C	2.08038	-1.65066	0.18962
N	2.27763	-2.22329	-1.00388
N	1.87436	-2.254	1.36111
C	2.17135	-3.55559	-1.0187
C	1.77303	-3.58834	1.30446
C	1.893	-4.3005	0.12076
H	2.32628	-4.03919	-1.98065
H	1.59624	-4.09658	2.25006
H	1.7996	-5.3788	0.08963
C	2.51656	0.43428	1.41587
C	1.6665	1.32847	2.06726
C	3.78591	0.17605	1.93317
C	2.08605	1.96475	3.23532
C	4.20537	0.8117	3.10211
H	4.45595	-0.52881	1.41999
C	3.35575	1.70604	3.75314
H	1.41632	2.67004	3.74847
H	5.2061	0.60759	3.50997
H	3.68631	2.20779	4.67416

2e-IN5

Energy: -1524.258272 Hartree

H	-0.15019	-0.64948	2.05973
N	-0.03228	-1.84287	-0.46977
N	1.90489	-0.2032	0.34217
Mn	-0.24939	0.05221	0.4706
C	-0.3803	0.92874	-1.05945
O	-0.4893	1.58181	-2.03394
H	0.15206	-2.52835	0.26839
C	-0.63463	-3.28223	3.03236
O	-0.70874	-4.44921	3.40758
O	0.35292	-2.78053	2.34717
C	-0.35017	1.577	1.3326
O	-0.37153	2.614	1.89307
N	-2.32425	-0.56521	0.52224
H	2.1084	-0.8066	1.13964
H	-2.53869	-0.57892	1.51593
H	-1.44204	-2.55308	3.26974
H	0.08544	-1.60183	2.13253
C	-2.39849	-1.93653	-0.04881
H	-3.39089	-2.12189	-0.46711
H	-2.24991	-2.62542	0.78736

C	-1.32946	-2.16568	-1.09164
H	-1.37338	-3.20357	-1.44102
H	-1.46602	-1.504	-1.95193
C	-3.11112	0.43662	-0.09876
N	-3.38693	0.27706	-1.3928
N	-3.46819	1.45016	0.69829
C	-4.02868	1.30301	-1.96816
C	-4.10991	2.4517	0.08885
C	-4.40069	2.44139	-1.27006
H	-4.25516	1.19379	-3.02657
H	-4.40737	3.28615	0.71985
H	-4.91126	3.26623	-1.75098
C	2.50709	1.06041	0.58088
N	2.90719	1.24861	1.8417
N	2.54798	1.92379	-0.43391
C	3.34418	2.48375	2.11863
C	3.00538	3.14151	-0.12039
C	3.40556	3.48786	1.16342
H	3.6641	2.66075	3.14259
H	3.04883	3.86096	-0.93509
H	3.76029	4.4827	1.40164
C	2.14045	-0.90137	-0.90618
C	1.11706	-1.75625	-1.33171
C	3.30556	-0.77085	-1.65041
C	1.24458	-2.44909	-2.53012
C	3.42814	-1.46426	-2.85334
H	4.10143	-0.12006	-1.30295
C	2.39921	-2.29092	-3.29546
H	0.44522	-3.10305	-2.86502
H	4.3312	-1.35227	-3.44483
H	2.49426	-2.82295	-4.23682

Group-3

3a-IN1

Energy: -942.278868 Hartree

N	1.62975	1.64197	-1.41654
N	3.48778	-0.46129	-1.10481
Mn	1.30983	-0.41824	-0.96188
H	1.58393	1.74525	-2.42912
C	0.95171	-2.11285	-0.79323
O	0.6553	-3.25664	-0.68843
N	-0.69423	0.33701	-1.24316
C	0.54368	2.36417	-0.80579
C	0.6305	3.65835	-0.3084
C	-0.67653	1.68629	-0.70803
C	-0.46906	4.2675	0.28702
H	1.56852	4.2023	-0.37696
C	-1.78911	2.29083	-0.14456
C	-1.70259	3.59387	0.39102
H	-0.35894	5.27384	0.67322
H	-2.71819	1.73503	-0.10521
C	2.99528	1.96551	-0.97696
H	3.00141	1.97264	0.11701
H	3.32643	2.94978	-1.33085
C	3.91778	0.89668	-1.51846
H	3.88562	0.90592	-2.61159
H	4.9489	1.08459	-1.20657
N	-2.79448	4.17574	1.02418
C	-4.10254	3.60069	0.7612
H	-4.16105	2.57198	1.1265
H	-4.85624	4.17881	1.29598
H	-4.36381	3.59685	-0.30788
C	-2.77738	5.61989	1.18348
H	-1.95415	5.93472	1.82963
H	-2.68299	6.15655	0.22744
H	-3.70502	5.93258	1.66332
H	-0.92468	0.35341	-2.2161
C	1.28193	-0.19346	0.79962
O	1.25983	-0.1463	1.98391
H	1.33713	-0.67269	-2.62517
H	3.88427	-0.68935	-0.21555
H	3.77226	-1.13309	-1.78873
H	-1.35019	-0.22618	-0.74063

3a-IN2

Energy: -1130.855289 Hartree

H	-2.50812	0.07096	-1.12506
N	-0.64671	-1.16995	0.37394
N	-2.91988	-0.02196	1.46295
Mn	-1.53617	0.77057	0.07924
C	-0.56125	1.57147	1.30726
O	0.05423	2.17755	2.12175
H	-1.02031	-1.79122	-0.34423
C	-3.29237	-1.51094	-1.74415
O	-2.33748	-2.07667	-2.16238
O	-4.42404	-1.29616	-1.47083
C	-2.34725	2.25145	-0.31655
O	-2.91581	3.25647	-0.59951
N	0.0367	0.70957	-1.36569
H	-3.87585	0.04261	1.12642
C	0.76692	-0.99125	0.17472
C	1.76832	-1.65882	0.8662
C	1.12666	0.0005	-0.74268
C	3.10749	-1.34093	0.65485
H	1.50935	-2.41645	1.60082
C	2.45406	0.32071	-0.97267
C	3.48307	-0.35613	-0.2802
H	3.86069	-1.86498	1.23094
H	2.67971	1.10987	-1.68171
C	-1.10122	-1.64515	1.68944
H	-0.59763	-1.04341	2.45508
H	-0.8513	-2.69849	1.86571
C	-2.59793	-1.44185	1.73765
H	-3.07172	-2.04969	0.95912
H	-3.00404	-1.76933	2.69967
N	4.82059	-0.07013	-0.53573
C	5.11652	1.20561	-1.16568
H	4.74519	2.06649	-0.58917
H	6.19695	1.30455	-1.27215
H	4.68509	1.25924	-2.16828
C	5.78933	-0.49453	0.46061
H	5.59816	-0.06611	1.45626
H	5.80134	-1.58279	0.55833
H	6.78455	-0.18605	0.13923
H	-0.32772	0.17448	-2.15269
H	-2.88809	0.50002	2.3334
H	0.36387	1.59296	-1.74598

3a-TS2-3

Energy: -1130.850815 Hartree

H	-1.42862	-0.60862	2.10635
N	-1.41413	1.8305	0.93549
N	-3.3771	-0.10553	0.38351
Mn	-1.21532	-0.24613	0.45674
C	-0.94665	0.04792	-1.2661
O	-0.74928	0.16995	-2.428
H	-1.52601	1.93408	1.94465
C	-2.24216	-0.00478	3.64715
O	-1.44522	0.7911	4.02018
O	-3.22748	-0.6635	3.63746
C	-0.98943	-1.95957	0.24157
O	-0.78617	-3.11963	0.11029
N	0.79046	0.31046	1.04322
H	-3.6565	-0.68261	1.1734
C	-0.18459	2.46054	0.52221
C	-0.08533	3.75936	0.03977
C	0.97065	1.67278	0.57581
C	1.13563	4.26863	-0.39038
H	-0.97497	4.37909	-0.02979
C	2.19921	2.17694	0.18194
C	2.31005	3.49386	-0.31257
H	1.16585	5.27524	-0.79018
H	3.06769	1.53213	0.23874
C	-2.65155	2.28108	0.27668
H	-2.46112	2.29585	-0.80196
H	-2.94283	3.29416	0.58037
C	-3.75389	1.31248	0.64675
H	-3.9507	1.38735	1.72079
H	-4.68303	1.5664	0.12667
N	3.54638	4.00302	-0.69201
C	4.59616	3.04009	-0.98244

H	4.30671	2.30996	-1.75405
H	5.48007	3.57559	-1.3289
H	4.88176	2.485	-0.08518
C	3.54422	5.18312	-1.53972
H	3.00047	5.03214	-2.48453
H	3.0952	6.03557	-1.02423
H	4.57463	5.4503	-1.77487
H	0.68165	0.32236	2.05725
H	-3.7427	-0.53361	-0.44298
H	1.47931	-0.37381	0.80396

3a-IN3

Energy: -1130.872616 Hartree

H	-2.50812	0.07096	-1.12506
N	-0.64671	-1.16995	0.37394
N	-2.91988	-0.02196	1.46295
Mn	-1.53617	0.77057	0.07924
C	-0.56125	1.57147	1.30726
O	0.05423	2.17755	2.12175
H	-1.02031	-1.79122	-0.34423
C	-3.29237	-1.51094	-1.74415
O	-2.33748	-2.07667	-2.16238
O	-4.42404	-1.29616	-1.47083
C	-2.34725	2.25145	-0.31655
O	-2.91581	3.25647	-0.59951
N	0.0367	0.70957	-1.36569
H	-3.87585	0.04261	1.12642
C	0.76692	-0.99125	0.17472
C	1.76832	-1.65882	0.8662
C	1.12666	0.0005	-0.74268
C	3.10749	-1.34093	0.65485
H	1.50935	-2.41645	1.60082
C	2.45406	0.32071	-0.97267
C	3.48307	-0.35613	-0.2802
H	3.86069	-1.86498	1.23094
H	2.67971	1.10987	-1.68171
C	-1.10122	-1.64515	1.68944
H	-0.59763	-1.04341	2.45508
H	-0.8513	-2.69849	1.86571
C	-2.59793	-1.44185	1.73765
H	-3.07172	-2.04969	0.95912
H	-3.00404	-1.76933	2.69967
N	4.82059	-0.07013	-0.53573
C	5.11652	1.20561	-1.16568
H	4.74519	2.06649	-0.58917
H	6.19695	1.30455	-1.27215
H	4.68509	1.25924	-2.16828
C	5.78933	-0.49453	0.46061
H	5.59816	-0.06611	1.45626
H	5.80134	-1.58279	0.55833
H	6.78455	-0.18605	0.13923
H	-0.32772	0.17448	-2.15269
H	-2.88809	0.50002	2.3334
H	0.36387	1.59296	-1.74598

3a-IN3R

Energy: -1130.893853 Hartree

N	1.23404	1.9359	-1.07485
N	3.33265	0.1523	-0.57229
Mn	1.20022	-0.10825	-0.57073
C	1.0089	0.16608	1.14357
O	0.87751	0.3298	2.30744
H	1.26508	1.93668	-2.09623
C	1.11294	-1.83971	-0.29096
O	1.00342	-2.99749	-0.09188
N	-0.85445	0.28611	-1.02577
H	3.75323	-0.48381	-1.21919
C	-0.00411	2.50972	-0.61705
C	-0.16789	3.82466	-0.20143
C	-1.10582	1.64805	-0.58511
C	-1.40273	4.2763	0.25145
H	0.67757	4.5068	-0.22176
C	-2.34885	2.0907	-0.16346
C	-2.51943	3.41661	0.29076
H	-1.49106	5.3051	0.57992
H	-3.17878	1.39436	-0.16952

C	2.49553	2.49624	-0.56799
H	2.40921	2.57234	0.52128
H	2.69538	3.50042	-0.9606
C	3.6028	1.55183	-0.99031
H	3.66665	1.54023	-2.08306
H	4.57085	1.89225	-0.60915
N	-3.7436	3.84387	0.78753
C	-4.91185	3.0393	0.47509
H	-4.82746	2.03931	0.90891
H	-5.7917	3.51062	0.91308
H	-5.08086	2.92682	-0.60651
C	-3.97436	5.27721	0.84768
H	-3.28291	5.75846	1.54403
H	-3.86873	5.76973	-0.13047
H	-4.98467	5.4566	1.21571
H	-1.01054	0.20806	-2.01042
O	1.29615	-0.28358	-2.65199
C	2.12823	-1.016	-3.2898
O	3.08745	-1.65882	-2.8245
H	1.94396	-1.05151	-4.38484
H	-1.44548	-0.35091	-0.53092
H	3.6959	-0.00524	0.34599

3a-IN4

Energy: -1132.030425 Hartree

H	-2.62376	0.32963	0.69737
N	-0.75012	0.39428	-1.16967
N	-2.96412	-1.26764	-1.21155
Mn	-1.64452	-0.81423	0.36413
C	-0.62366	-2.20591	0.0571
O	0.0242	-3.18196	-0.11247
H	-1.14356	1.3346	-1.09685
C	-2.32118	4.26341	0.59033
O	-2.17857	5.47877	0.49777
O	-2.52447	3.46324	-0.41926
C	-2.43412	-1.67016	1.65764
O	-2.97216	-2.2512	2.5394
N	-0.12186	0.24556	1.40618
H	-3.93903	-1.15194	-0.95279
C	0.65676	0.43496	-0.86859
C	1.6794	0.4691	-1.80662
C	0.98752	0.35712	0.48725
C	3.01084	0.4164	-1.40369
H	1.4434	0.50735	-2.86659
C	2.3067	0.31501	0.90524
C	3.35704	0.35241	-0.03912
H	3.78206	0.41789	-2.16467
H	2.51002	0.23665	1.96779
C	-1.16796	-0.17991	-2.45913
H	-0.63741	-1.1296	-2.59469
H	-0.92157	0.47156	-3.30592
C	-2.6606	-0.39797	-2.37187
H	-3.1601	0.56237	-2.20697
H	-3.04929	-0.81919	-3.30405
N	4.68519	0.34634	0.37324
C	4.96238	-0.1485	1.7115
H	4.60536	-1.17718	1.87157
H	6.03954	-0.12961	1.8782
H	4.50464	0.48998	2.47111
C	5.68499	-0.00039	-0.62247
H	5.52336	-0.99456	-1.0661
H	5.70246	0.73205	-1.43327
H	6.6686	0.0104	-0.15237
H	-0.48304	1.17144	1.63271
H	-2.28785	3.75347	1.57789
H	-2.54587	1.06298	0.3779
H	0.19294	-0.1596	2.28325
H	-2.85959	-2.23907	-1.48927

3a-TS4-5

Energy: -1132.020639 Hartree

H	-1.48511	-1.1479	2.18389
N	-1.68555	1.40208	1.57457
N	-3.50189	-0.60287	0.78434
Mn	-1.35647	-0.45226	0.58685
C	-1.4022	0.26219	-1.02073

O	-1.44235	0.70448	-2.11521
H	-1.60357	1.246	2.57965
C	0.76136	-2.77949	2.95683
O	1.72139	-3.37812	3.43979
O	0.27132	-1.66463	3.41485
C	-1.01448	-2.01591	-0.12541
O	-0.75995	-3.05394	-0.62921
N	0.65272	0.24162	1.00717
H	-3.7401	-1.42965	1.29395
C	-0.63755	2.28384	1.12262
C	-0.78504	3.64788	0.91064
C	0.58472	1.68112	0.80671
C	0.25956	4.40286	0.38611
H	-1.73407	4.13012	1.12782
C	1.64095	2.42134	0.30461
C	1.50198	3.80977	0.08683
H	0.09672	5.45874	0.20619
H	2.56699	1.91206	0.06658
C	-3.07104	1.79896	1.27313
H	-3.09939	2.11615	0.22517
H	-3.40557	2.6429	1.88781
C	-3.95934	0.60222	1.52964
H	-3.91319	0.34268	2.59096
H	-5.00231	0.83274	1.28974
N	2.56968	4.55883	-0.39074
C	3.64556	3.83691	-1.04975
H	3.29668	3.2259	-1.89624
H	4.37653	4.55467	-1.42201
H	4.16437	3.17657	-0.34984
C	2.26494	5.86275	-0.95447
H	1.56265	5.81121	-1.80013
H	1.83554	6.52552	-0.19924
H	3.19001	6.32204	-1.30309
H	0.90464	0.03169	1.95188
H	0.23496	-3.17782	2.06009
H	-0.68787	-1.35935	2.7048
H	1.305	-0.16962	0.37045
H	-3.92675	-0.62564	-0.12063

3a-IN5

Energy: -1132.019313 Hartree

H	-2.75099	0.47824	0.74067
N	-0.75012	0.39428	-1.16967
N	-2.96412	-1.26764	-1.21155
Mn	-1.64452	-0.81423	0.36413
C	-0.62366	-2.20591	0.0571
O	0.0242	-3.18196	-0.11247
H	-1.14356	1.3346	-1.09685
C	-2.37941	3.32634	0.93448
O	-2.23681	4.5417	0.84192
O	-2.5827	2.52618	-0.0751
C	-2.43412	-1.67016	1.65764
O	-2.97216	-2.2512	2.5394
N	-0.12186	0.24556	1.40618
H	-3.93903	-1.15194	-0.95279
C	0.65676	0.43496	-0.86859
C	1.6794	0.4691	-1.80662
C	0.98752	0.35712	0.48725
C	3.01084	0.4164	-1.40369
H	1.4434	0.50735	-2.86659
C	2.3067	0.31501	0.90524
C	3.35704	0.35241	-0.03912
H	3.78206	0.41789	-2.16467
H	2.51002	0.23665	1.96779
C	-1.16796	-0.17991	-2.45913
H	-0.63741	-1.1296	-2.59469
H	-0.92157	0.47156	-3.30592
C	-2.6606	-0.39797	-2.37187
H	-3.1601	0.56237	-2.20697
H	-3.04929	-0.81919	-3.30405
N	4.68519	0.34634	0.37324
C	4.96238	-0.1485	1.7115
H	4.60536	-1.17718	1.87157
H	6.03954	-0.12961	1.8782
H	4.50464	0.48998	2.47111
C	5.68499	-0.00039	-0.62247

H	5.52336	-0.99456	-1.0661
H	5.70246	0.73205	-1.43327
H	6.6686	0.0104	-0.15237
H	-0.48304	1.17144	1.63271
H	-2.34609	2.8164	1.92204
H	-2.65213	1.40902	0.33519
H	0.19294	-0.1596	2.28325
H	-2.85959	-2.23907	-1.48927

3b-IN1

Energy: -1671.956225 Hartree

N	1.62975	1.64197	-1.41654
N	3.48778	-0.46129	-1.10481
Mn	1.30983	-0.41824	-0.96188
H	1.58393	1.74525	-2.42912
C	0.95171	-2.11285	-0.79323
O	0.6553	-3.25664	-0.68843
N	-0.69423	0.33701	-1.24316
C	0.54368	2.36417	-0.80579
C	0.6305	3.65835	-0.3084
C	-0.67653	1.68629	-0.70803
C	-0.46906	4.2675	0.28702
H	1.56852	4.2023	-0.37696
C	-1.78911	2.29083	-0.14456
C	-1.70259	3.59387	0.39102
H	-0.35894	5.27384	0.67322
H	-2.71819	1.73503	-0.10521
C	4.01164	-0.97183	0.10268
C	4.44678	-2.79399	1.3655
C	4.77043	-0.61421	2.19924
H	4.48841	-3.87782	1.44959
H	5.07348	0.09434	2.9674
C	2.99528	1.96551	-0.97696
H	3.00141	1.97264	0.11701
H	3.32643	2.94978	-1.33085
C	3.91778	0.89668	-1.51846
H	3.88562	0.90592	-2.61159
H	4.9489	1.08459	-1.20657
N	-2.79448	4.17574	1.02418
C	-4.10254	3.60069	0.7612
H	-4.16105	2.57198	1.1265
H	-4.85624	4.17881	1.29598
H	-4.36381	3.59685	-0.30788
C	-2.77738	5.61989	1.18348
H	-1.95415	5.93472	1.82963
H	-2.68299	6.15655	0.22744
H	-3.70502	5.93258	1.66332
C	-1.71878	-0.56892	-0.80233
C	-2.39744	-1.34556	-1.73681
C	-1.9807	-0.76713	0.55377
C	-3.31235	-2.31157	-1.3337
H	-2.20457	-1.19957	-2.79769
C	-2.9019	-1.72056	0.96282
H	-1.46432	-0.16754	1.29885
C	-3.58051	-2.53442	0.03056
H	-3.82219	-2.88956	-2.09584
H	-3.08806	-1.83326	2.02481
N	-4.45961	-3.53427	0.44603
C	-5.40182	-4.02961	-0.54269
H	-4.88248	-4.51959	-1.37004
H	-6.04433	-4.77621	-0.07485
H	-6.04081	-3.23838	-0.96463
C	-5.00343	-3.41457	1.78832
H	-5.7006	-4.23513	1.96056
H	-4.21566	-3.49402	2.54135
H	-5.53763	-2.46636	1.95464
H	-0.72543	0.38904	-2.26018
C	1.28193	-0.19346	0.79962
O	1.25983	-0.1463	1.98391
H	3.64193	-1.13588	-1.84694
H	1.33713	-0.67269	-2.62517
C	4.38921	-0.08748	1.02832
H	4.38649	0.96671	0.84507
C	4.79805	-1.97985	2.43586
C	4.05584	-2.30886	0.18384
H	3.79716	-2.9444	-0.63718
N	5.17739	-2.53198	3.74435
C	5.47764	-3.96408	3.6035
H	5.87921	-4.33641	4.52275
H	4.57966	-4.49505	3.36556
H	6.19259	-4.1032	2.81967
C	6.36673	-1.82907	4.24664
H	6.0904	-0.84772	4.57144

H	6.78185	-2.37327	5.06909
H	7.09364	-1.75457	3.46501

3b-IN2

Energy: -1860.532152 Hartree

H	0.24741	-0.05364	2.73847
N	0.19773	-2.29849	1.21797
N	2.5297	-0.7462	1.58026
Mn	0.51001	-0.17975	1.05889
C	0.79523	-0.20921	-0.68396
O	1.00431	-0.13967	-1.84901
H	-0.06914	-2.53968	2.17162
C	-0.40002	-0.89083	4.27175
O	-1.51092	-1.13896	3.93626
O	0.56456	-0.82871	4.95447
C	0.64648	1.55375	1.08425
O	0.68369	2.7395	1.10186
N	-1.63755	-0.38599	0.91138
H	2.6738	-0.2792	2.47117
C	-0.89087	-2.60572	0.32523
C	-1.01397	-3.78281	-0.40073
C	-1.8571	-1.606	0.15952
C	-2.07351	-3.96706	-1.28389
H	-0.26115	-4.55993	-0.30111
C	-2.93461	-1.78863	-0.69116
C	-3.06853	-2.98112	-1.43439
H	-2.11784	-4.88521	-1.85736
H	-3.65985	-0.99014	-0.78969
C	3.50792	-0.24923	0.65029
C	4.80627	1.63169	-0.15781
C	4.9189	-0.47719	-1.30341
C	5.31835	0.86956	-1.22867
H	5.1037	2.66611	-0.02814
H	5.30342	-1.12108	-2.08615
C	1.50591	-2.91532	0.94371
H	1.71819	-2.77801	-0.1222
H	1.51406	-3.99159	1.15558
C	2.52088	-2.2126	1.81982
H	2.24283	-2.35905	2.86857
H	3.5206	-2.63829	1.68094
N	-4.16254	-3.17127	-2.27096
C	-4.8854	-1.98304	-2.69429
H	-4.2391	-1.24056	-3.18745
H	-5.66874	-2.27612	-3.39327
H	-5.37216	-1.49386	-1.84647
C	-4.05359	-4.21509	-3.27556
H	-3.2034	-4.0677	-3.95866
H	-3.9454	-5.19772	-2.80963
H	-4.96975	-4.23413	-3.86609
C	-2.3031	0.81731	0.49891
C	-2.93931	1.61093	1.44894
C	-2.23342	1.27036	-0.81926
C	-3.49003	2.83896	1.09971
H	-2.99953	1.2681	2.48026
C	-2.79221	2.48835	-1.17747
H	-1.73821	0.66451	-1.57423
C	-3.42128	3.31675	-0.22312
H	-3.981	3.42222	1.87013
H	-2.72824	2.79774	-2.21443
N	-3.92865	4.56823	-0.57234
C	-4.89366	5.1594	0.33867
H	-4.43952	5.36842	1.31052
H	-5.23157	6.11075	-0.07329
H	-5.77556	4.52194	0.50685
C	-4.21353	4.79143	-1.97988
H	-4.64244	5.78688	-2.09945
H	-3.29922	4.75844	-2.57734
H	-4.91961	4.05789	-2.3988
H	-1.85386	-0.56189	1.89423
C	4.02748	-1.02371	-0.38413
H	3.74905	-2.06794	-0.49133
C	3.91661	1.08175	0.75299
H	3.53073	1.69606	1.56406
N	6.16509	1.43576	-2.18527
C	6.96085	0.51184	-2.97546
H	6.32465	-0.12296	-3.59728
H	7.60376	1.08277	-3.64639
H	7.59769	-0.14439	-2.36171
C	6.8691	2.64646	-1.79687
H	6.17001	3.46364	-1.6042
H	7.49329	2.51497	-0.8992
H	7.51347	2.95919	-2.6192

3b-TS2-3

Energy: -1860.526284 Hartree

H	1.30653	1.06669	2.10549
N	1.67498	-1.40569	1.23469
N	3.41113	0.71245	0.55735
Mn	1.23442	0.55225	0.48311
C	1.13841	0.07673	-1.22678
O	1.04825	-0.16522	-2.37926
H	1.63629	-1.36035	2.25259
C	1.03997	0.73265	3.89248
O	0.35497	-0.2408	3.84279
O	1.65858	1.61754	4.37195
C	0.79912	2.20634	0.10901
O	0.46764	3.31673	-0.1138
N	-0.70669	-0.25265	0.98469
H	3.54902	1.51557	1.16169
C	0.616	-2.25721	0.7536
C	0.76759	-3.58557	0.37892
C	-0.64034	-1.65432	0.61396
C	-0.30425	-4.30708	-0.13678
H	1.73954	-4.06393	0.46219
C	-1.72511	-2.36627	0.1324
C	-1.57914	-3.71681	-0.25823
H	-0.1421	-5.33137	-0.45059
H	-2.67947	-1.8628	0.03897
C	3.88861	1.02671	-0.73615
C	4.2294	2.61584	-2.3012
C	4.6451	0.33635	-2.7463
C	4.61748	1.64182	-3.21669
H	4.21705	3.66956	-2.57359
H	4.96997	-0.48478	-3.38259
C	3.05073	-1.7212	0.82469
H	3.05193	-1.8847	-0.25687
H	3.43952	-2.62362	1.31365
C	3.92139	-0.53356	1.17847
H	3.90533	-0.36901	2.2603
H	4.95641	-0.71685	0.87598
N	-2.65975	-4.42537	-0.73838
C	-3.87111	-3.71212	-1.08219
H	-3.7011	-2.92235	-1.8299
H	-4.59777	-4.41584	-1.48689
H	-4.32033	-3.24289	-0.19969
C	-2.4419	-5.73462	-1.31327
H	-1.77288	-5.71166	-2.18674
H	-2.00678	-6.41947	-0.57753
H	-3.39895	-6.15149	-1.62585
C	-1.83278	0.52512	0.54667
C	-2.54775	1.28796	1.46566
C	-2.17374	0.60455	-0.80417
C	-3.57617	2.12661	1.05245
H	-2.29668	1.23371	2.52356
C	-3.20826	1.42554	-1.22528
H	-1.62217	0.01646	-1.53329
C	-3.92875	2.22481	-0.30892
H	-4.11067	2.70037	1.80077
H	-3.45407	1.44955	-2.28087
N	-4.93021	3.07658	-0.73398
C	-5.78759	3.6945	0.25291
H	-5.2111	4.33273	0.93158
H	-6.5173	4.32689	-0.25254
H	-6.3328	2.96123	0.86703
C	-5.41642	2.96604	-2.09152
H	-6.20455	3.70206	-2.24963
H	-4.62053	3.17488	-2.81472
H	-5.82416	1.96974	-2.32174
H	-0.63995	-0.18785	2.0012
C	4.28781	0.00855	-1.49932
H	4.31914	-0.99794	-1.13753
C	3.86561	2.32736	-1.05004
H	3.57672	3.0833	-0.35003
N	5.00505	1.98113	-4.59348
C	6.27447	1.31711	-4.92296
H	6.0957	0.27903	-5.1109
H	6.69788	1.76974	-5.79517
H	6.95383	1.41924	-4.10262
C	5.16858	3.43721	-4.71177
H	4.22496	3.91614	-4.55329
H	5.86923	3.77817	-3.97846
H	5.53059	3.67719	-5.68966

3b-IN3

Energy: -1860.548756 Hartree

H	0.24741	-0.05364	2.73847
N	0.19773	-2.29849	1.21797

N	2.5297	-0.7462	1.58026
Mn	0.51001	-0.17975	1.05889
C	0.79523	-0.20921	-0.68396
O	1.00431	-0.13967	-1.84901
H	-0.06914	-2.53968	2.17162
C	-0.40002	-0.89083	4.27175
O	-1.51092	-1.13896	3.93626
O	0.56456	-0.82871	4.95447
C	0.64648	1.55375	1.08425
O	0.68369	2.7395	1.10186
N	-1.63755	-0.38599	0.91138
H	2.6738	-0.2792	2.47117
C	-0.89087	-2.60572	0.32523
C	-1.01397	-3.78281	-0.40073
C	-1.8571	-1.606	0.15952
C	-2.07351	-3.96706	-1.28389
H	-0.26115	-4.55993	-0.30111
C	-2.93461	-1.78863	-0.69116
C	-3.06853	-2.98112	-1.43439
H	-2.11784	-4.88521	-1.85736
H	-3.65985	-0.99014	-0.78969
C	3.50792	-0.24923	0.65029
C	4.80627	1.63169	-0.15781
C	4.9189	-0.47719	-1.30341
C	5.31835	0.86956	-1.22867
H	5.1037	2.66611	-0.02814
H	5.30342	-1.12108	-2.08615
C	1.50591	-2.91532	0.94371
H	1.71819	-2.77801	-0.1222
H	1.51406	-3.99159	1.15558
C	2.52088	-2.2126	1.81982
H	2.24283	-2.35905	2.86857
H	3.5206	-2.63829	1.68094
N	-4.16254	-3.17127	-2.27096
C	-4.8854	-1.98304	-2.69429
H	-4.2391	-1.24056	-3.18745
H	-5.66874	-2.27612	-3.39327
H	-5.37216	-1.49386	-1.84647
C	-4.05359	-4.21509	-3.27556
H	-3.2034	-4.0677	-3.95866
H	-3.9454	-5.19772	-2.80963
H	-4.96975	-4.23413	-3.86609
C	-2.3031	0.81731	0.49891
C	-2.93931	1.61093	1.44894
C	-2.23342	1.27036	-0.81926
C	-3.49003	2.83896	1.09971
H	-2.99953	1.2681	2.48026
C	-2.79221	2.48835	-1.17747
H	-1.73821	0.66451	-1.57423
C	-3.42128	3.31675	-0.22312
H	-3.981	3.42222	1.87013
H	-2.72824	2.79774	-2.21443
N	-3.92865	4.56823	-0.57234
C	-4.89366	5.1594	0.33867
H	-4.43952	5.36842	1.31052
H	-5.23157	6.11075	-0.07329
H	-5.77556	4.52194	0.50685
C	-4.21353	4.79143	-1.97988
H	-4.64244	5.78688	-2.09945
H	-3.29922	4.75844	-2.57734
H	-4.91961	4.05789	-2.3988
H	-1.85386	-0.56189	1.89423
C	4.02748	-1.02371	-0.38413
H	3.74905	-2.06794	-0.49133
C	3.91661	1.08175	0.75299
H	3.53073	1.69606	1.56406
N	6.16509	1.43576	-2.18527
C	6.96085	0.51184	-2.97546
H	6.32465	-0.12296	-3.59728
H	7.60376	1.08277	-3.64639
H	7.59769	-0.14439	-2.36171
C	6.8691	2.64646	-1.79687
H	6.17001	3.46364	-1.6042
H	7.49329	2.51497	-0.8992
H	7.51347	2.95919	-2.6192

3b-IN3R

Energy: -1860.574962 Hartree

N	-1.3941	-1.77812	-1.16199
N	-3.36687	0.12299	-0.58678
Mn	-1.20832	0.23644	-0.58025
C	-1.03583	-0.09435	1.12821
O	-0.9272	-0.27453	2.29157
H	-1.396	-1.73761	-2.18298

C	-0.98487	1.94757	-0.24679
O	-0.78041	3.08522	-0.01402
N	0.80573	-0.29724	-1.05911
H	-3.53375	0.86915	-1.27161
C	-0.21248	-2.45612	-0.69875
C	-0.15605	-3.78406	-0.2982
C	0.95153	-1.68187	-0.64068
C	1.03484	-4.33382	0.16535
H	-1.05066	-4.39926	-0.33748
C	2.15127	-2.22244	-0.20967
C	2.21307	-3.56296	0.2303
H	1.04002	-5.37011	0.48179
H	3.03182	-1.59146	-0.19449
C	-3.86656	0.49008	0.67776
C	-4.34	2.16277	2.12226
C	-4.52674	-0.09841	2.75699
C	-4.61467	1.23579	3.12117
H	-4.4278	3.2307	2.31044
H	-4.76255	-0.89015	3.46508
C	-2.70816	-2.26173	-0.71104
H	-2.66876	-2.38079	0.37563
H	-2.97028	-3.22927	-1.15566
C	-3.7277	-1.21571	-1.10883
H	-3.75222	-1.12021	-2.19898
H	-4.72701	-1.50383	-0.77229
N	3.39392	-4.08701	0.73959
C	4.62478	-3.37365	0.44585
H	4.61556	-2.37476	0.89038
H	5.46104	-3.91767	0.88502
H	4.81154	-3.26327	-0.63303
C	3.51255	-5.53403	0.79293
H	2.77023	-5.96496	1.46951
H	3.39242	-6.01118	-0.19123
H	4.4971	-5.79277	1.18292
C	1.78101	0.66614	-0.6258
C	2.2924	1.58261	-1.53973
C	2.13841	0.78197	0.71772
C	3.13855	2.60478	-1.12625
H	2.01991	1.50207	-2.59006
C	2.9913	1.79303	1.13559
H	1.74982	0.07436	1.44571
C	3.50118	2.74582	0.22748
H	3.51876	3.29366	-1.8716
H	3.25562	1.84089	2.18565
N	4.30774	3.79747	0.65796
C	5.10658	4.4681	-0.35349
H	4.47103	4.96298	-1.09205
H	5.70888	5.23998	0.12642
H	5.78275	3.78557	-0.8911
C	4.96759	3.63953	1.94301
H	5.58391	4.51867	2.13355
H	4.23879	3.57225	2.75435
H	5.61186	2.74811	1.99045
H	0.7418	-0.26089	-2.07853
O	-1.30991	0.5115	-2.64792
C	-2.09361	1.33884	-3.22855
O	-2.99754	2.02122	-2.71057
H	-1.92113	1.42612	-4.32223
C	-4.16252	-0.49436	1.5299
H	-4.11151	-1.52577	1.24975
C	-3.96839	1.81035	0.88894
H	-3.767	2.53289	0.12587
N	-5.00634	1.64813	4.47671
C	-5.45432	3.048	4.45281
H	-5.87654	3.30362	5.40217
H	-4.6197	3.68617	4.25018
H	-6.19279	3.17317	3.68868
C	-6.10191	0.7911	4.95223
H	-5.71966	-0.1813	5.18292
H	-6.53581	1.22137	5.83058
H	-6.8477	0.70941	4.18933

IN4

Energy: -1861.712099 Hartree

H	-0.38183	-0.62953	2.88168
N	-0.49062	1.83333	1.98182
N	-2.63318	-0.0058	1.91857
Mn	-0.60092	-0.14806	1.21823
C	-1.02311	0.35217	-0.41471
O	-1.35028	0.63469	-1.51448
H	-0.13918	1.78049	2.9382
C	1.86191	-2.37564	3.32969
O	2.87105	-3.00464	3.64374
O	1.5987	-1.15619	3.70077

C	-0.5838	-1.81596	0.69042
O	-0.54455	-2.93379	0.30496
N	1.5045	0.35199	1.01199
H	-2.69833	-0.76312	2.59298
C	0.45539	2.52653	1.14441
C	0.35747	3.8594	0.77017
C	1.49577	1.75223	0.61866
C	1.27074	4.41663	-0.12031
H	-0.4571	4.4694	1.15056
C	2.42539	2.29742	-0.24975
C	2.33367	3.65183	-0.63901
H	1.14411	5.45191	-0.4134
H	3.21123	1.66101	-0.63823
C	-3.61399	-0.2412	0.88985
C	-4.73049	-1.83962	-0.54986
C	-5.17446	0.50824	-0.8015
C	-5.40093	-0.80861	-1.24141
H	-4.8936	-2.87727	-0.81751
H	-5.68732	1.33962	-1.27129
C	-1.8703	2.34698	2.01773
H	-2.16978	2.55026	0.98389
H	-1.95145	3.28253	2.58355
C	-2.73362	1.27993	2.6547
H	-2.3748	1.08913	3.67051
H	-3.77513	1.61063	2.72876
N	3.27944	4.20447	-1.49467
C	4.05875	3.28169	-2.30419
H	3.43276	2.61348	-2.91522
H	4.70322	3.85451	-2.97111
H	4.70647	2.65919	-1.68141
C	2.92634	5.45073	-2.15286
H	2.01401	5.37088	-2.76318
H	2.77529	6.25008	-1.42343
H	3.74878	5.75307	-2.80132
C	2.32949	-0.54175	0.23743
C	3.42634	-1.16894	0.82046
C	2.03526	-0.80833	-1.10023
C	4.19586	-2.07757	0.10185
H	3.69035	-0.94387	1.85103
C	2.80988	-1.69572	-1.83097
H	1.19699	-0.30905	-1.57947
C	3.89829	-2.37695	-1.2416
H	5.03836	-2.54498	0.59843
H	2.55477	-1.86652	-2.8705
N	4.62941	-3.32151	-1.95586
C	5.9317	-3.68603	-1.42574
H	5.8371	-4.15754	-0.44441
H	6.39405	-4.41347	-2.09346
H	6.61227	-2.82665	-1.32493
C	4.55851	-3.2565	-3.40611
H	5.20882	-4.02459	-3.82564
H	3.54497	-3.45905	-3.76093
H	4.871	-2.28111	-3.80904
H	1.82154	0.29416	1.9822
H	1.08252	-2.83616	2.68154
H	0.51613	-0.84902	3.21851
N	-6.23341	-1.08356	-2.32758
C	-6.741	-2.44094	-2.43652
H	-7.39469	-2.50419	-3.30713
H	-5.92722	-3.15407	-2.58923
H	-7.31201	-2.76167	-1.55132
C	-7.19381	-0.05466	-2.68893
H	-6.68879	0.85427	-3.02524
H	-7.80162	-0.41465	-3.51968
H	-7.86622	0.21796	-1.86063
C	-3.853	-1.55588	0.4853
H	-3.34364	-2.37147	0.99421
C	-4.29471	0.78591	0.24134
H	-4.15676	1.82151	0.53642

3b-TS4-5

Energy: -1861.695506 Hartree

H	-0.40556	-0.78106	2.88103
N	-0.63397	1.69643	2.0623
N	-2.68885	-0.2295	1.95377
Mn	-0.65036	-0.26132	1.23288
C	-1.0909	0.25662	-0.39113
O	-1.42388	0.53795	-1.48914
H	-0.26377	1.62493	3.01039
C	1.9213	-2.43024	3.27533
O	2.96324	-3.01628	3.56495
O	1.60321	-1.2337	3.67567
C	-0.5524	-1.91214	0.65826
O	-0.46223	-3.01469	0.2424

N	1.42292	0.34727	1.03898
H	-2.7358	-1.03654	2.56907
C	0.26742	2.46129	1.2382
C	0.09844	3.79671	0.90086
C	1.34236	1.75634	0.68535
C	0.97737	4.42416	0.02232
H	-0.74493	4.3529	1.30044
C	2.23857	2.37151	-0.17098
C	2.0763	3.73025	-0.52061
H	0.79546	5.45893	-0.24203
H	3.05292	1.78667	-0.58114
C	-3.60445	-0.43118	0.88075
C	-4.54328	-1.90198	-0.56082
C	-4.9123	0.4238	-0.75639
C	-5.10684	-0.85189	-1.31208
H	-4.71364	-2.93955	-0.83404
H	-5.38034	1.30315	-1.19023
C	-2.03382	2.14693	2.13851
H	-2.36861	2.37072	1.12135
H	-2.14459	3.05316	2.74558
C	-2.84507	1.02114	2.73806
H	-2.47948	0.80021	3.74503
H	-3.89937	1.30195	2.81144
N	2.98911	4.35507	-1.36199
C	3.81376	3.49833	-2.19843
H	3.22244	2.81758	-2.82987
H	4.42728	4.12361	-2.84707
H	4.49297	2.89127	-1.5942
C	2.57157	5.60095	-1.98188
H	1.65942	5.49452	-2.58836
H	2.38811	6.37134	-1.2289
H	3.37368	5.96027	-2.62668
C	2.29345	-0.4804	0.24056
C	3.42261	-1.06433	0.80677
C	2.01334	-0.7238	-1.1045
C	4.24059	-1.9074	0.06263
H	3.67421	-0.85634	1.84398
C	2.83506	-1.5458	-1.86
H	1.1498	-0.25641	-1.57067
C	3.95987	-2.18255	-1.28967
H	5.10735	-2.34294	0.54624
H	2.58922	-1.69982	-2.90439

N	4.7433	-3.06245	-2.03009
C	6.06554	-3.36526	-1.51034
H	6.00053	-3.87229	-0.54445
H	6.56995	-4.0423	-2.20019
H	6.69342	-2.47023	-1.38187
C	4.66556	-2.96281	-3.47798
H	5.35785	-3.68083	-3.91837
H	3.66458	-3.21388	-3.83735
H	4.92056	-1.96064	-3.85479
H	1.74252	0.27948	2.00793
H	1.15819	-2.91451	2.62501
H	0.49897	-0.96678	3.20548
N	-5.79965	-1.0621	-2.48929
C	-6.28727	-2.40948	-2.73695
H	-6.80507	-2.42358	-3.69609
H	-5.45767	-3.1177	-2.8007
H	-6.98213	-2.76306	-1.96092
C	-6.68122	0.00623	-2.93166
H	-6.11547	0.91873	-3.13498
H	-7.16063	-0.29728	-3.8623
H	-7.46498	0.24467	-2.19749
C	-3.7933	-1.70254	0.5218
H	-3.36741	-2.51608	1.07104
C	-4.16448	0.64041	0.32966
H	-4.02172	1.61975	0.73637

3b-IN5

Energy: -1861.695875 Hartree

H	-0.38183	-0.62953	2.88168
N	-0.49062	1.83333	1.98182
N	-2.63318	-0.0058	1.91857
Mn	-0.60092	-0.14806	1.21823
C	-1.02311	0.35217	-0.41471
O	-1.35028	0.63469	-1.51448
H	-0.13918	1.78049	2.9382
C	1.86191	-2.37564	3.32969
O	2.87105	-3.00464	3.64374
O	1.5987	-1.15619	3.70077
C	-0.5838	-1.81596	0.69042
O	-0.54455	-2.93379	0.30496
N	1.5045	0.35199	1.01199
H	-2.69833	-0.76312	2.59298

C	0.45539	2.52653	1.14441
C	0.35747	3.8594	0.77017
C	1.49577	1.75223	0.61866
C	1.27074	4.41663	-0.12031
H	-0.4571	4.4694	1.15056
C	2.42539	2.29742	-0.24975
C	2.33367	3.65183	-0.63901
H	1.14411	5.45191	-0.4134
H	3.21123	1.66101	-0.63823
C	-3.61399	-0.2412	0.88985
C	-4.73049	-1.83962	-0.54986
C	-5.17446	0.50824	-0.8015
C	-5.40093	-0.80861	-1.24141
H	-4.8936	-2.87727	-0.81751
H	-5.68732	1.33962	-1.27129
C	-1.8703	2.34698	2.01773
H	-2.16978	2.55026	0.98389
H	-1.95145	3.28253	2.58355
C	-2.73362	1.27993	2.6547
H	-2.3748	1.08913	3.67051
H	-3.77513	1.61063	2.72876
N	3.27944	4.20447	-1.49467
C	4.05875	3.28169	-2.30419
H	3.43276	2.61348	-2.91522
H	4.70322	3.85451	-2.97111
H	4.70647	2.65919	-1.68141
C	2.92634	5.45073	-2.15286
H	2.01401	5.37088	-2.76318
H	2.77529	6.25008	-1.42343
H	3.74878	5.75307	-2.80132
C	2.32949	-0.54175	0.23743
C	3.42634	-1.16894	0.82046
C	2.03526	-0.80833	-1.10023
C	4.19586	-2.07757	0.10185
H	3.69035	-0.94387	1.85103
C	2.80988	-1.69572	-1.83097
H	1.19699	-0.30905	-1.57947
C	3.89829	-2.37695	-1.2416
H	5.03836	-2.54498	0.59843
H	2.55477	-1.86652	-2.8705
N	4.62941	-3.32151	-1.95586
C	5.9317	-3.68603	-1.42574
H	5.8371	-4.15754	-0.44441
H	6.39405	-4.41347	-2.09346
H	6.61227	-2.82665	-1.32493
C	4.55851	-3.2565	-3.40611
H	5.20882	-4.02459	-3.82564
H	3.54497	-3.45905	-3.76093
H	4.871	-2.28111	-3.80904
H	1.82154	0.29416	1.9822
H	1.08252	-2.83616	2.68154
H	0.51613	-0.84902	3.21851
N	-6.23341	-1.08356	-2.32758
C	-6.741	-2.44094	-2.43652
H	-7.39469	-2.50419	-3.30713
H	-5.92722	-3.15407	-2.58923
H	-7.31201	-2.76167	-1.55132
C	-7.19381	-0.05466	-2.68893
H	-6.68879	0.85427	-3.02524
H	-7.80162	-0.41465	-3.51968
H	-7.86622	0.21796	-1.86063
C	-3.853	-1.55588	0.4853
H	-3.34364	-2.37147	0.99421
C	-4.29471	0.78591	0.24134
H	-4.15676	1.82151	0.53642

3c-IN1

Energy: -1554.127658 Hartree

N	1.62975	1.64197	-1.41654
N	3.48778	-0.46129	-1.10481
Mn	1.30983	-0.41824	-0.96188
H	1.58393	1.74525	-2.42912
C	0.95171	-2.11285	-0.79323
O	0.6553	-3.25664	-0.68843
N	-0.69423	0.33701	-1.24316
C	0.54368	2.36417	-0.80579
C	0.6305	3.65835	-0.3084
C	-0.67653	1.68629	-0.70803
C	-0.46906	4.2675	0.28702
H	1.56852	4.2023	-0.37696
C	-1.78911	2.29083	-0.14456
C	-1.70259	3.59387	0.39102
H	-0.35894	5.27384	0.67322
H	-2.71819	1.73503	-0.10521

C	4.01164	-0.97183	0.10268
C	4.44678	-2.79399	1.3655
C	4.77043	-0.61421	2.19924
H	4.48841	-3.87782	1.44959
H	5.07348	0.09434	2.9674
C	2.99528	1.96551	-0.97696
H	3.00141	1.97264	0.11701
H	3.32643	2.94978	-1.33085
C	3.91778	0.89668	-1.51846
H	3.88562	0.90592	-2.61159
H	4.9489	1.08459	-1.20657
N	4.05584	-2.30886	0.18384
N	-2.79448	4.17574	1.02418
C	-4.10254	3.60069	0.7612
H	-4.16105	2.57198	1.1265
H	-4.85624	4.17881	1.29598
H	-4.36381	3.59685	-0.30788
C	-2.77738	5.61989	1.18348
H	-1.95415	5.93472	1.82963
H	-2.68299	6.15655	0.22744
H	-3.70502	5.93258	1.66332
C	-1.71878	-0.56892	-0.80233
C	-2.39744	-1.34556	-1.73681
C	-1.9807	-0.76713	0.55377
C	-3.31235	-2.31157	-1.3337
H	-2.20457	-1.19957	-2.79769
C	-2.9019	-1.72056	0.96282
H	-1.46432	-0.16754	1.29885
C	-3.58051	-2.53442	0.03056
H	-3.82219	-2.88956	-2.09584
H	-3.08806	-1.83326	2.02481
N	-4.45961	-3.53427	0.44603
C	-5.40182	-4.02961	-0.54269
H	-4.88248	-4.51959	-1.37004
H	-6.04433	-4.77621	-0.07485
H	-6.04081	-3.23838	-0.96463
C	-5.00343	-3.41457	1.78832
H	-5.7006	-4.23513	1.96056
H	-4.21566	-3.49402	2.54135
H	-5.53763	-2.46636	1.95464
H	-0.72543	0.38904	-2.26018
C	1.28193	-0.19346	0.79962
O	1.25983	-0.1463	1.98391
H	3.64193	-1.13588	-1.84694
H	1.33713	-0.67269	-2.62517
C	4.38921	-0.08748	1.02832
H	4.38649	0.96671	0.84507
C	4.79805	-1.97985	2.43586
H	5.07417	-2.38174	3.3883

3c-IN2

Energy: -1742.702013 Hartree

H	-1.42862	-0.60862	2.10635
N	-1.41413	1.8305	0.93549
N	-3.3771	-0.10553	0.38351
Mn	-1.21532	-0.24613	0.45674
C	-0.94665	0.04792	-1.2661
O	-0.74928	0.16995	-2.428
H	-1.52601	1.93408	1.94465
C	-2.24216	-0.00478	3.64715
O	-1.44522	0.7911	4.02018
O	-3.22748	-0.6635	3.63746
C	-0.98943	-1.95957	0.24157
O	-0.78617	-3.11963	0.11029
N	0.79046	0.31046	1.04322
H	-3.6565	-0.68261	1.1734
C	-0.18459	2.46054	0.52221
C	-0.08533	3.75936	0.03977
C	0.97065	1.67278	0.57581
C	1.13563	4.26863	-0.39038
H	-0.97497	4.37909	-0.02979
C	2.19921	2.17694	0.18194
C	2.31005	3.49386	-0.31257
H	1.16585	5.27524	-0.79018
H	3.06769	1.53213	0.23874
C	-3.89771	-0.71511	-0.79339
C	-4.63075	-2.64985	-1.76231
C	-4.52732	-0.67745	-3.09526
C	-4.81512	-2.03705	-2.99398
H	-4.856	-3.70641	-1.63401
H	-4.66362	-0.14928	-4.03417
H	-5.1782	-2.60538	-3.84298
C	-2.65155	2.28108	0.27668
H	-2.46112	2.29585	-0.80196

H	-2.94283	3.29416	0.58037
C	-3.75389	1.31248	0.64675
H	-3.9507	1.38735	1.72079
H	-4.68303	1.5664	0.12667
N	-4.18917	-2.0131	-0.66812
N	3.54638	4.00302	-0.69201
C	4.59616	3.04009	-0.98244
H	4.30671	2.30996	-1.75405
H	5.48007	3.57559	-1.3289
H	4.88176	2.485	-0.08518
C	3.54422	5.18312	-1.53972
H	3.00047	5.03214	-2.48453
H	3.0952	6.03557	-1.02423
H	4.57463	5.4503	-1.77487
C	1.78065	-0.67315	0.6993
C	2.25933	-1.54092	1.67607
C	2.19756	-0.84899	-0.62078
C	3.12884	-2.575	1.34743
H	1.94442	-1.41206	2.70966
C	3.0749	-1.87027	-0.95371
H	1.83807	-0.17765	-1.39648
C	3.55041	-2.77621	0.0189
H	3.48102	-3.22424	2.14068
H	3.38603	-1.96354	-1.98788
N	4.38124	-3.84078	-0.3284
C	5.13969	-4.45744	0.74632
H	4.47585	-4.92184	1.47979
H	5.76574	-5.24668	0.32904
H	5.78896	-3.74586	1.27962
C	5.09939	-3.73114	-1.58704
H	5.73397	-4.60958	-1.7085
H	4.40921	-3.70903	-2.43392
H	5.73566	-2.83445	-1.64413
H	0.68165	0.32236	2.05725
C	-4.06045	0.00349	-1.98048
H	-3.81731	1.05946	-2.03531

3c-TS2-3

Energy: -1742.696852 Hartree

H	1.30653	1.06669	2.10549
N	1.67498	-1.40569	1.23469
N	3.41113	0.71245	0.55735
Mn	1.23442	0.55225	0.48311
C	1.13841	0.07673	-1.22678
O	1.04825	-0.16522	-2.37926
H	1.63629	-1.36035	2.25259
C	1.03997	0.73265	3.89248
O	0.35497	-0.2408	3.84279
O	1.65858	1.61754	4.37195
C	0.79912	2.20634	0.10901
O	0.46764	3.31673	-0.1138
N	-0.70669	-0.25265	0.98469
H	3.54902	1.51557	1.16169
C	0.616	-2.25721	0.7536
C	0.76759	-3.58557	0.37892
C	-0.64034	-1.65432	0.61396
C	-0.30425	-4.30708	-0.13678
H	1.73954	-4.06393	0.46219
C	-1.72511	-2.36627	0.1324
C	-1.57914	-3.71681	-0.25823
H	-0.1421	-5.33137	-0.45059
H	-2.67947	-1.8628	0.03897
C	3.88861	1.02671	-0.73615
C	4.2294	2.61584	-2.3012
C	4.6451	0.33635	-2.7463
C	4.61748	1.64182	-3.21669
H	4.21705	3.66956	-2.57359
H	4.96997	-0.48478	-3.38259
H	4.90295	1.89174	-4.23078
C	3.05073	-1.7212	0.82469
H	3.05193	-1.8847	-0.25687
H	3.43952	-2.62362	1.31365
C	3.92139	-0.53356	1.17847
H	3.90533	-0.36901	2.2603
H	4.95641	-0.71685	0.87598
N	3.86561	2.32736	-1.05004
N	-2.65975	-4.42537	-0.73838
C	-3.87111	-3.71212	-1.08219
H	-3.7011	-2.92235	-1.8299
H	-4.59777	-4.41584	-1.48689
H	-4.32033	-3.24289	-0.19969
C	-2.4419	-5.73462	-1.31327
H	-1.77288	-5.71166	-2.18674
H	-2.00678	-6.41947	-0.57753

H	-3.39895	-6.15149	-1.62585
C	-1.83278	0.52512	0.54667
C	-2.54775	1.28796	1.46566
C	-2.17374	0.60455	-0.80417
C	-3.57617	2.12661	1.05245
H	-2.29668	1.23371	2.52356
C	-3.20826	1.42554	-1.22528
H	-1.62217	0.01646	-1.53329
C	-3.92875	2.22481	-0.30892
H	-4.11067	2.70037	1.80077
H	-3.45407	1.44955	-2.28087
N	-4.93021	3.07658	-0.73398
C	-5.78759	3.6945	0.25291
H	-5.2111	4.33273	0.93158
H	-6.5173	4.32689	-0.25254
H	-6.3328	2.96123	0.86703
C	-5.41642	2.96604	-2.09152
H	-6.20455	3.70206	-2.24963
H	-4.62053	3.17488	-2.81472
H	-5.82416	1.96974	-2.32174
H	-0.63995	-0.18785	2.0012
C	4.28781	0.00855	-1.49932
H	4.31914	-0.99794	-1.13753

3c-IN3

Energy: -1742.717669 Hartree

H	-1.42862	-0.60862	2.10635
N	-1.41413	1.8305	0.93549
N	-3.3771	-0.10553	0.38351
Mn	-1.21532	-0.24613	0.45674
C	-0.94665	0.04792	-1.2661
O	-0.74928	0.16995	-2.428
H	-1.52601	1.93408	1.94465
C	-2.24216	-0.00478	3.64715
O	-1.44522	0.7911	4.02018
O	-3.22748	-0.6635	3.63746
C	-0.98943	-1.95957	0.24157
O	-0.78617	-3.11963	0.11029
N	0.79046	0.31046	1.04322
H	-3.6565	-0.68261	1.1734
C	-0.18459	2.46054	0.52221
C	-0.08533	3.75936	0.03977
C	0.97065	1.67278	0.57581
C	1.13563	4.26863	-0.39038
H	-0.97497	4.37909	-0.02979
C	2.19921	2.17694	0.18194
C	2.31005	3.49386	-0.31257
H	1.16585	5.27524	-0.79018
H	3.06769	1.53213	0.23874
C	-3.89771	-0.71511	-0.79339
C	-4.63075	-2.64985	-1.76231
C	-4.52732	-0.67745	-3.09526
C	-4.81512	-2.03705	-2.99398
H	-4.856	-3.70641	-1.63401
H	-4.66362	-0.14928	-4.03417
H	-5.1782	-2.60538	-3.84298
C	-2.65155	2.28108	0.27668
H	-2.46112	2.29585	-0.80196
H	-2.94283	3.29416	0.58037
C	-3.75389	1.31248	0.64675
H	-3.9507	1.38735	1.72079
H	-4.68303	1.5664	0.12667
N	-4.18917	-2.0131	-0.66812
N	3.54638	4.00302	-0.69201
C	4.59616	3.04009	-0.98244
H	4.30671	2.30996	-1.75405
H	5.48007	3.57559	-1.3289
H	4.88176	2.485	-0.08518
C	3.54422	5.18312	-1.53972
H	3.00047	5.03214	-2.48453
H	3.0952	6.03557	-1.02423
H	4.57463	5.4503	-1.77487
C	1.78065	-0.67315	0.6993
C	2.25933	-1.54092	1.67607
C	2.19756	-0.84899	-0.62078
C	3.12884	-2.575	1.34743
H	1.94442	-1.41206	2.70966
C	3.0749	-1.87027	-0.95371
H	1.83807	-0.17765	-1.39648
C	3.55041	-2.77621	0.0189
H	3.48102	-3.22424	2.14068
H	3.38603	-1.96354	-1.98788
N	4.38124	-3.84078	-0.3284
C	5.13969	-4.45744	0.74632

H	4.47585	-4.92184	1.47979
H	5.76574	-5.24668	0.32904
H	5.78896	-3.74586	1.27962
C	5.09939	-3.73114	-1.58704
H	5.73397	-4.60958	-1.7085
H	4.40921	-3.70903	-2.43392
H	5.73566	-2.83445	-1.64413
H	0.68165	0.32236	2.05725
C	-4.06045	0.00349	-1.98048
H	-3.81731	1.05946	-2.03531

3c-IN3R

Energy: -1742.744891 Hartree

N	-1.3941	-1.77812	-1.16199
N	-3.36687	0.12299	-0.58678
Mn	-1.20832	0.23644	-0.58025
C	-1.03583	-0.09435	1.12821
O	-0.9272	-0.27453	2.29157
H	-1.396	-1.73761	-2.18298
C	-0.98487	1.94757	-0.24679
O	-0.78041	3.08522	-0.01402
N	0.80573	-0.29724	-1.05911
H	-3.53375	0.86915	-1.27161
C	-0.21248	-2.45612	-0.69875
C	-0.15605	-3.78406	-0.2982
C	0.95153	-1.68187	-0.64068
C	1.03484	-4.33382	0.16535
H	-1.05066	-4.39926	-0.33748
C	2.15127	-2.22244	-0.20967
C	2.21307	-3.56296	0.2303
H	1.04002	-5.37011	0.48179
H	3.03182	-1.59146	-0.19449
C	-3.86656	0.49008	0.67776
C	-4.34	2.16277	2.12226
C	-4.52674	-0.09841	2.75699
C	-4.61467	1.23579	3.12117
H	-4.4278	3.2307	2.31044
H	-4.76255	-0.89015	3.46508
H	-4.90317	1.53952	4.11967
C	-2.70816	-2.26173	-0.71104
H	-2.66876	-2.38079	0.37563
H	-2.97028	-3.22927	-1.15566
C	-3.7277	-1.21571	-1.10883
H	-3.75222	-1.12021	-2.19898
H	-4.72701	-1.50383	-0.77229
N	-3.96839	1.81035	0.88894
N	3.39392	-4.08701	0.73959
C	4.62478	-3.37365	0.44585
H	4.61556	-2.37476	0.89038
H	5.46104	-3.91767	0.88502
H	4.81154	-3.26327	-0.63303
C	3.51255	-5.53403	0.79293
H	2.77023	-5.96496	1.46951
H	3.39242	-6.01118	-0.19123
H	4.4971	-5.79277	1.18292
C	1.78101	0.66614	-0.6258
C	2.2924	1.58261	-1.53973
C	2.13841	0.78197	0.71772
C	3.13855	2.60478	-1.12625
H	2.01991	1.50207	-2.59006
C	2.9913	1.79303	1.13559
H	1.74982	0.07436	1.44571
C	3.50118	2.74582	0.22748
H	3.51876	3.29366	-1.8716
H	3.25562	1.84089	2.18565
N	4.30774	3.79747	0.65796
C	5.10658	4.4681	-0.35349
H	4.47103	4.96298	-1.09205
H	5.70888	5.23998	0.12642
H	5.78275	3.78557	-0.8911
C	4.96759	3.63953	1.94301
H	5.58391	4.51867	2.13355
H	4.23879	3.57225	2.75435
H	5.61186	2.74811	1.99045
H	0.7418	-0.26089	-2.07853
O	-1.30991	0.5115	-2.64792
C	-2.09361	1.33884	-3.22855
O	-2.99754	2.02122	-2.71057
H	-1.92113	1.42612	-4.32223
C	-4.16252	-0.49436	1.5299
H	-4.11151	-1.52577	1.24975

3c-IN4

Energy: -1743.883436 Hartree

H	-1.48511	-1.1479	2.18389
N	-1.68555	1.40208	1.57457
N	-3.50189	-0.60287	0.78434
Mn	-1.35647	-0.45226	0.58685
C	-1.4022	0.26219	-1.02073
O	-1.44235	0.70448	-2.11521
H	-1.60357	1.246	2.57965
C	0.76136	-2.77949	2.95683
O	1.72139	-3.37812	3.43979
O	0.27132	-1.66463	3.41485
C	-1.01448	-2.01591	-0.12541
O	-0.75995	-3.05394	-0.62921
N	0.65272	0.24162	1.00717
H	-3.60208	-1.42341	1.37576
C	-0.63755	2.28384	1.12262
C	-0.78504	3.64788	0.91064
C	0.58472	1.68112	0.80671
C	0.25956	4.40286	0.38611
H	-1.73407	4.13012	1.12782
C	1.64095	2.42134	0.30461
C	1.50198	3.80977	0.08683
H	0.09672	5.45874	0.20619
H	2.56699	1.91206	0.06658
C	-4.15046	-0.89716	-0.45181
C	-4.79592	-2.53726	-1.90483
C	-5.19301	-0.26538	-2.50288
H	-4.8573	-3.60412	-2.10826
H	-5.56696	0.4934	-3.18359
C	-3.07104	1.79896	1.27313
H	-3.09939	2.11615	0.22517
H	-3.40557	2.6429	1.88781
C	-3.95934	0.60222	1.52964
H	-3.91319	0.34268	2.59096
H	-5.00231	0.83274	1.28974
N	2.56968	4.55883	-0.39074
C	3.64556	3.83691	-1.04975
H	3.29668	3.2259	-1.89624
H	4.37653	4.55467	-1.42201
H	4.16437	3.17657	-0.34984
C	2.26494	5.86275	-0.95447
H	1.56265	5.81121	-1.80013
H	1.83554	6.52552	-0.19924
H	3.19001	6.32204	-1.30309
C	1.75054	-0.45576	0.3821
C	2.7328	-1.05948	1.16146
C	1.84096	-0.55161	-1.00681
C	3.76505	-1.78401	0.57622
H	2.69669	-0.96021	2.24358
C	2.87853	-1.25373	-1.60025
H	1.09858	-0.06335	-1.63283
C	3.8565	-1.91406	-0.82313
H	4.50479	-2.24118	1.22323
H	2.91971	-1.29573	-2.68258
N	4.8541	-2.6792	-1.4186
C	6.01719	-2.99599	-0.60808
H	5.73994	-3.60852	0.25356
H	6.71836	-3.5767	-1.20794
H	6.53858	-2.10143	-0.23443
C	5.13796	-2.42441	-2.82085
H	5.96072	-3.06649	-3.13641
H	4.27665	-2.66772	-3.44781
H	5.41884	-1.37908	-3.02017
H	0.71616	0.06357	2.01201
H	0.23496	-3.17782	2.06009
H	-0.68787	-1.35935	2.7048
C	-4.62099	0.11082	-1.2954
H	-4.53586	1.15768	-1.02404
N	-4.24364	-2.20019	-0.73124
C	-5.28054	-1.61727	-2.82497
H	-5.71799	-1.95138	-3.75908

3c-TS4-5

Energy: -1743.865588 Hartree

H	-0.40556	-0.78106	2.88103
N	-0.63397	1.69643	2.0623
N	-2.68885	-0.2295	1.95377
Mn	-0.65036	-0.26132	1.23288
C	-1.0909	0.25662	-0.39113
O	-1.42388	0.53795	-1.48914
H	-0.26377	1.62493	3.01039
C	1.9213	-2.43024	3.27533
O	2.96324	-3.01628	3.56495
O	1.60321	-1.2337	3.67567

C	-0.5524	-1.91214	0.65826
O	-0.46223	-3.01469	0.2424
N	1.42292	0.34727	1.03898
H	-2.7358	-1.03654	2.56907
C	0.26742	2.46129	1.2382
C	0.09844	3.79671	0.90086
C	1.34236	1.75634	0.68535
C	0.97737	4.42416	0.02232
H	-0.74493	4.3529	1.30044
C	2.23857	2.37151	-0.17098
C	2.0763	3.73025	-0.52061
H	0.79546	5.45893	-0.24203
H	3.05292	1.78667	-0.58114
C	-3.60445	-0.43118	0.88075
C	-4.54328	-1.90198	-0.56082
C	-4.9123	0.4238	-0.75639
H	-4.71364	-2.93955	-0.83404
H	-5.38034	1.30315	-1.19023
C	-2.03382	2.14693	2.13851
H	-2.36861	2.37072	1.12135
H	-2.14459	3.05316	2.74558
C	-2.84507	1.02114	2.73806
H	-2.47948	0.80021	3.74503
H	-3.89937	1.30195	2.81144
N	2.98911	4.35507	-1.36199
C	3.81376	3.49833	-2.19843
H	3.22244	2.81758	-2.82987
H	4.42728	4.12361	-2.84707
H	4.49297	2.89127	-1.5942
C	2.57157	5.60095	-1.98188
H	1.65942	5.49452	-2.58836
H	2.38811	6.37134	-1.2289
H	3.37368	5.96027	-2.62668
C	2.29345	-0.4804	0.24056
C	3.42261	-1.06433	0.80677
C	2.01334	-0.7238	-1.1045
C	4.24059	-1.9074	0.06263
H	3.67421	-0.85634	1.84398
C	2.83506	-1.5458	-1.86
H	1.1498	-0.25641	-1.57067
C	3.95987	-2.18255	-1.28967
H	5.10735	-2.34294	0.54624
H	2.58922	-1.69982	-2.90439
N	4.7433	-3.06245	-2.03009
C	6.06554	-3.36526	-1.51034
H	6.00053	-3.87229	-0.54445
H	6.56995	-4.0423	-2.20019
H	6.69342	-2.47023	-1.38187
C	4.66556	-2.96281	-3.47798
H	5.35785	-3.68083	-3.91837
H	3.66458	-3.21388	-3.83735
H	4.92056	-1.96064	-3.85479
H	1.74252	0.27948	2.00793
H	1.15819	-2.91451	2.62501
H	0.49897	-0.96678	3.20548
C	-4.16448	0.64041	0.32966
H	-4.02172	1.61975	0.73637
N	-3.7933	-1.70254	0.5218
C	-5.10684	-0.85189	-1.31208
H	-5.63783	-1.0099	-2.2275

3c-IN5

Energy: -1743.866137 Hartree

H	-1.48511	-1.1479	2.18389
N	-1.68555	1.40208	1.57457
N	-3.50189	-0.60287	0.78434
Mn	-1.35647	-0.45226	0.58685
C	-1.4022	0.26219	-1.02073
O	-1.44235	0.70448	-2.11521
H	-1.60357	1.246	2.57965
C	0.76136	-2.77949	2.95683
O	1.72139	-3.37812	3.43979
O	0.27132	-1.66463	3.41485
C	-1.01448	-2.01591	-0.12541
O	-0.75995	-3.05394	-0.62921
N	0.65272	0.24162	1.00717
H	-3.60208	-1.42341	1.37576
C	-0.63755	2.28384	1.12262
C	-0.78504	3.64788	0.91064
C	0.58472	1.68112	0.80671
C	0.25956	4.40286	0.38611
H	-1.73407	4.13012	1.12782
C	1.64095	2.42134	0.30461
C	1.50198	3.80977	0.08683

H	0.09672	5.45874	0.20619
H	2.56699	1.91206	0.06658
C	-4.15046	-0.89716	-0.45181
C	-4.79592	-2.53726	-1.90483
C	-5.19301	-0.26538	-2.50288
H	-4.8573	-3.60412	-2.10826
H	-5.56696	0.4934	-3.18359
C	-3.07104	1.79896	1.27313
H	-3.09939	2.11615	0.22517
H	-3.40557	2.6429	1.88781
C	-3.95934	0.60222	1.52964
H	-3.91319	0.34268	2.59096
H	-5.00231	0.83274	1.28974
N	2.56968	4.55883	-0.39074
C	3.64556	3.83691	-1.04975
H	3.29668	3.2259	-1.89624
H	4.37653	4.55467	-1.42201
H	4.16437	3.17657	-0.34984
C	2.26494	5.86275	-0.95447
H	1.56265	5.81121	-1.80013
H	1.83554	6.52552	-0.19924
H	3.19001	6.32204	-1.30309
C	1.75054	-0.45576	0.3821
C	2.7328	-1.05948	1.16146
C	1.84096	-0.55161	-1.00681
C	3.76505	-1.78401	0.57622
H	2.69669	-0.96021	2.24358
C	2.87853	-1.25373	-1.60025
H	1.09858	-0.06335	-1.63283
C	3.8565	-1.91406	-0.82313
H	4.50479	-2.24118	1.22323
H	2.91971	-1.29573	-2.68258
N	4.8541	-2.6792	-1.4186
C	6.01719	-2.99599	-0.60808
H	5.73994	-3.60852	0.25356
H	6.71836	-3.5767	-1.20794
H	6.53858	-2.10143	-0.23443
C	5.13796	-2.42441	-2.82085
H	5.96072	-3.06649	-3.13641
H	4.27665	-2.66772	-3.44781
H	5.41884	-1.37908	-3.02017
H	0.71616	0.06357	2.01201
H	0.23496	-3.17782	2.06009
H	-0.68787	-1.35935	2.7048
C	-4.62099	0.11082	-1.2954
H	-4.53586	1.15768	-1.02404
N	-4.24364	-2.20019	-0.73124
C	-5.28054	-1.61727	-2.82497
H	-5.71799	-1.95138	-3.75908

3d-IN1

Energy: -1570.186136 Hartree

N	-0.94051	1.48166	0.84867
N	1.27364	0.60473	1.64396
Mn	-0.07334	-0.15266	0.48705
C	-0.62118	-1.5241	1.73516
O	-1.00936	-2.43005	2.50785
H	-0.62426	2.2179	0.24974
C	1.42	-0.68275	-0.64764
O	2.27245	-0.98537	-1.29537
N	-1.48382	-0.38377	-0.89416
H	1.89642	1.05858	0.97778
C	-2.34469	1.17484	0.69038
C	-3.35283	1.6181	1.54401
C	-2.61716	0.34304	-0.34536
C	-4.66372	1.27615	1.22588
H	-3.13299	2.1839	2.42922
C	-3.9014	0.18281	-0.80285
C	-4.93144	0.64507	-0.00096
H	-5.4661	1.4825	1.90922
H	-4.08133	-0.28686	-1.74698
C	1.97323	-0.38992	2.48538
C	3.58722	-2.00655	2.93832
C	1.84874	-1.56031	4.53044
C	2.96736	-2.29462	4.16387
H	4.46169	-2.54588	2.6493
H	1.3515	-1.75086	5.46457
H	3.34848	-3.06321	4.80631
C	-0.72708	1.78023	2.20454
H	-1.26403	1.01726	2.72981
H	-1.10313	2.74386	2.50155
C	0.72656	1.60298	2.56708
H	1.35576	2.46728	2.50496
H	0.66761	1.27161	3.58195

N 1.39578 -0.61342 3.70272
N 3.07366 -1.054 2.13025
N -6.31146 0.4966 -0.38377
C -6.78817 1.74841 -1.02314
H -6.22959 1.9355 -1.92346
H -7.8258 1.65332 -1.26222
H -6.65213 2.57034 -0.34585
C -7.04319 0.20993 0.84717
H -6.66056 -0.69827 1.27665
H -6.9048 1.01918 1.53622
H -8.081 0.09538 0.63444
C -1.84235 -1.80773 -1.10768
C -0.94439 -2.66902 -1.76852
C -3.06974 -2.3165 -0.64127
C -1.25965 -4.0293 -1.93346
H -0.01244 -2.28659 -2.14922
C -3.39119 -3.67303 -0.82247
H -3.77458 -1.6589 -0.16441
C -2.48556 -4.52975 -1.46592
H -0.56462 -4.68968 -2.43005
H -4.33539 -4.05636 -0.46783
N -2.81227 -5.95444 -1.64507
C -1.99934 -6.50495 -2.73176
H -0.97033 -6.51366 -2.43703
H -2.32033 -7.5037 -2.93985
H -2.1138 -5.90283 -3.60761
C -4.23823 -6.07904 -1.97499
H -4.44984 -7.09196 -2.24494
H -4.82961 -5.80368 -1.12702
H -4.47301 -5.43382 -2.79654
H -1.19526 0.02295 -1.76782
H 0.29138 1.05285 -0.27099

3d-IN2

Energy: -1758.761489 Hartree

H 1.30653 1.06669 2.10549
N 1.67498 -1.40569 1.23469
N 3.41113 0.71245 0.55735
Mn 1.23442 0.55225 0.48311
C 1.13841 0.07673 -1.22678
O 1.04825 -0.16522 -2.37926
H 1.63629 -1.36035 2.25259
C 1.03997 0.73265 3.89248
O 0.35497 -0.2408 3.84279
O 1.65858 1.61754 4.37195
C 0.79912 2.20634 0.10901
O 0.46764 3.31673 -0.1138
N -0.70669 -0.25265 0.98469
H 3.54902 1.51557 1.16169
C 0.616 -2.25721 0.7536
C 0.76759 -3.58557 0.37892
C -0.64034 -1.65432 0.61396
C -0.30425 -4.30708 -0.13678
H 1.73954 -4.06393 0.46219
C -1.72511 -2.36627 0.1324
C -1.57914 -3.71681 -0.25823
H -0.1421 -5.33137 -0.45059
H -2.67947 -1.8628 0.03897
C 3.88861 1.02671 -0.73615
C 4.2294 2.61584 -2.3012
C 4.6451 0.33635 -2.7463
C 4.61748 1.64182 -3.21669
H 4.21705 3.66956 -2.57359
H 4.96997 -0.48478 -3.38259
H 4.90295 1.89174 -4.23078
C 3.05073 -1.7212 0.82469
H 3.05193 -1.8847 -0.25687
H 3.43952 -2.62362 1.31365
C 3.92139 -0.53356 1.17847
H 3.90533 -0.36901 2.2603
H 4.95641 -0.71685 0.87598
N 4.28781 0.00855 -1.49932
N 3.86561 2.32736 -1.05004
N -2.65975 -4.42537 -0.73838
C -3.87111 -3.71212 -1.08219
H -3.7011 -2.92235 -1.8299
H -4.59777 -4.41584 -1.48689
H -4.32033 -3.24289 -0.19969
C -2.4419 -5.73462 -1.31327
H -1.77288 -5.71166 -2.18674
H -2.00678 -6.41947 -0.57753
H -3.39895 -6.15149 -1.62585
C -1.83278 0.52512 0.54667
C -2.54775 1.28796 1.46566

C	-2.17374	0.60455	-0.80417
C	-3.57617	2.12661	1.05245
H	-2.29668	1.23371	2.52356
C	-3.20826	1.42554	-1.22528
H	-1.62217	0.01646	-1.53329
C	-3.92875	2.22481	-0.30892
H	-4.11067	2.70037	1.80077
H	-3.45407	1.44955	-2.28087
N	-4.93021	3.07658	-0.73398
C	-5.78759	3.6945	0.25291
H	-5.2111	4.33273	0.93158
H	-6.5173	4.32689	-0.25254
H	-6.3328	2.96123	0.86703
C	-5.41642	2.96604	-2.09152
H	-6.20455	3.70206	-2.24963
H	-4.62053	3.17488	-2.81472
H	-5.82416	1.96974	-2.32174
H	-0.63995	-0.18785	2.0012
H	-5.4661	1.4825	1.90922
H	-4.08133	-0.28686	-1.74698
C	1.97323	-0.38992	2.48538
C	3.58722	-2.00655	2.93832
C	1.84874	-1.56031	4.53044
C	2.96736	-2.29462	4.16387
H	4.46169	-2.54588	2.6493
H	1.3515	-1.75086	5.46457
H	3.34848	-3.06321	4.80631
C	-0.72708	1.78023	2.20454
H	-1.26403	1.01726	2.72981
H	-1.10313	2.74386	2.50155
C	0.72656	1.60298	2.56708
H	1.35576	2.46728	2.50496
H	0.66761	1.27161	3.58195
N	1.39578	-0.61342	3.70272
N	3.07366	-1.054	2.13025
N	-6.31146	0.4966	-0.38377
C	-6.78817	1.74841	-1.02314
H	-6.22959	1.9355	-1.92346
H	-7.8258	1.65332	-1.26222
H	-6.65213	2.57034	-0.34585
C	-7.04319	0.20993	0.84717
H	-6.66056	-0.69827	1.27665
H	-6.9048	1.01918	1.53622
H	-8.081	0.09538	0.63444
C	-1.84235	-1.80773	-1.10768
C	-0.94439	-2.66902	-1.76852
C	-3.06974	-2.3165	-0.64127
C	-1.25965	-4.0293	-1.93346
H	-0.01244	-2.28659	-2.14922
C	-3.39119	-3.67303	-0.82247
H	-3.77458	-1.6589	-0.16441
C	-2.48556	-4.52975	-1.46592
H	-0.56462	-4.68968	-2.43005
H	-4.33539	-4.05636	-0.46783
N	-2.81227	-5.95444	-1.64507
C	-1.99934	-6.50495	-2.73176
H	-0.97033	-6.51366	-2.43703
H	-2.32033	-7.5037	-2.93985
H	-2.1138	-5.90283	-3.60761
C	-4.23823	-6.07904	-1.97499

3d-TS2-3

Energy: -1758.755567 Hartree

H	0.72926	1.20429	-0.32198
N	-0.94051	1.48166	0.84867
N	1.27364	0.60473	1.64396
Mn	-0.07334	-0.15266	0.48705
C	-0.62118	-1.5241	1.73516
O	-1.00936	-2.43005	2.50785
H	-0.62426	2.2179	0.24974
C	0.76093	3.06442	-1.03528
O	-0.43404	3.04566	-1.54685
O	1.88868	3.60705	-0.68353
C	1.42	-0.68275	-0.64764
O	2.27245	-0.98537	-1.29537
N	-1.48382	-0.38377	-0.89416
H	1.89642	1.05858	0.97778
C	-2.34469	1.17484	0.69038
C	-3.35283	1.6181	1.54401
C	-2.61716	0.34304	-0.34536
C	-4.66372	1.27615	1.22588
H	-3.13299	2.1839	2.42922
C	-3.9014	0.18281	-0.80285
C	-4.93144	0.64507	-0.00096

H	-4.44984	-7.09196	-2.24494
H	-4.82961	-5.80368	-1.12702
H	-4.47301	-5.43382	-2.79654
H	-1.19526	0.02295	-1.76782

3d-IN3

Energy: -1758.776747 Hartree

H	1.30653	1.06669	2.10549
N	1.67498	-1.40569	1.23469
N	3.41113	0.71245	0.55735
Mn	1.23442	0.55225	0.48311
C	1.13841	0.07673	-1.22678
O	1.04825	-0.16522	-2.37926
H	1.63629	-1.36035	2.25259
C	1.03997	0.73265	3.89248
O	0.35497	-0.2408	3.84279
O	1.65858	1.61754	4.37195
C	0.79912	2.20634	0.10901
O	0.46764	3.31673	-0.1138
N	-0.70669	-0.25265	0.98469
H	3.54902	1.51557	1.16169
C	0.616	-2.25721	0.7536
C	0.76759	-3.58557	0.37892
C	-0.64034	-1.65432	0.61396
C	-0.30425	-4.30708	-0.13678
H	1.73954	-4.06393	0.46219
C	-1.72511	-2.36627	0.1324
C	-1.57914	-3.71681	-0.25823
H	-0.1421	-5.33137	-0.45059
H	-2.67947	-1.8628	0.03897
C	3.88861	1.02671	-0.73615
C	4.2294	2.61584	-2.3012
C	4.6451	0.33635	-2.7463
C	4.61748	1.64182	-3.21669
H	4.21705	3.66956	-2.57359
H	4.96997	-0.48478	-3.38259
H	4.90295	1.89174	-4.23078
C	3.05073	-1.7212	0.82469
H	3.05193	-1.8847	-0.25687
H	3.43952	-2.62362	1.31365
C	3.92139	-0.53356	1.17847
H	3.90533	-0.36901	2.2603
H	4.95641	-0.71685	0.87598
N	4.28781	0.00855	-1.49932
N	3.86561	2.32736	-1.05004
N	-2.65975	-4.42537	-0.73838
C	-3.87111	-3.71212	-1.08219
H	-3.7011	-2.92235	-1.8299
H	-4.59777	-4.41584	-1.48689
H	-4.32033	-3.24289	-0.19969
C	-2.4419	-5.73462	-1.31327
H	-1.77288	-5.71166	-2.18674
H	-2.00678	-6.41947	-0.57753
H	-3.39895	-6.15149	-1.62585
C	-1.83278	0.52512	0.54667
C	-2.54775	1.28796	1.46566
C	-2.17374	0.60455	-0.80417
C	-3.57617	2.12661	1.05245
H	-2.29668	1.23371	2.52356
C	-3.20826	1.42554	-1.22528
H	-1.62217	0.01646	-1.53329
C	-3.92875	2.22481	-0.30892
H	-4.11067	2.70037	1.80077
H	-3.45407	1.44955	-2.28087
N	-4.93021	3.07658	-0.73398
C	-5.78759	3.6945	0.25291
H	-5.2111	4.33273	0.93158
H	-6.5173	4.32689	-0.25254
H	-6.3328	2.96123	0.86703
C	-5.41642	2.96604	-2.09152
H	-6.20455	3.70206	-2.24963
H	-4.62053	3.17488	-2.81472
H	-5.82416	1.96974	-2.32174
H	-0.63995	-0.18785	2.0012

3d-IN3R

Energy: -1758.803345 Hartree

N	-0.8959	1.62911	0.75596
N	1.31825	0.75218	1.55124
Mn	-0.02873	-0.00521	0.39433
C	-0.57657	-1.37665	1.64244
O	-0.93271	-2.20781	2.35134
H	-0.57965	2.36535	0.15702

C	1.46461	-0.5353	-0.74036
O	2.31706	-0.83792	-1.38809
N	-1.43921	-0.23632	-0.98688
H	1.94103	1.20603	0.88506
C	-2.30008	1.32229	0.59766
C	-3.30822	1.76554	1.4513
C	-2.57255	0.49049	-0.43808
C	-4.61911	1.4236	1.13316
H	-3.08838	2.33135	2.3365
C	-3.85679	0.33026	-0.89557
C	-4.88683	0.79252	-0.09368
H	-5.42149	1.62995	1.81651
H	-4.03672	-0.13941	-1.8397
C	2.01784	-0.24247	2.39266
C	3.63183	-1.8591	2.84561
C	1.89335	-1.41286	4.43772
C	3.01197	-2.14717	4.07115
H	4.5063	-2.39843	2.55658
H	1.39611	-1.60341	5.37186
H	3.39309	-2.91576	4.71359
C	-0.68247	1.92768	2.11182
H	-1.21942	1.16471	2.63709
H	-1.05852	2.89131	2.40883
C	0.77117	1.75043	2.47436
H	1.40037	2.61473	2.41224
H	0.71222	1.41906	3.48923
N	1.44039	-0.46597	3.61
N	3.11827	-0.90655	2.03753
N	-6.26685	0.64405	-0.47649
C	-6.74356	1.89586	-1.11586
H	-6.18498	2.08294	-2.01618
H	-7.78119	1.80077	-1.35494
H	-6.60752	2.71779	-0.43857
C	-6.99858	0.35738	0.75445
H	-6.61595	-0.55082	1.18393
H	-6.86019	1.16663	1.4435
H	-8.03639	0.24283	0.54172
C	-1.79774	-1.66028	-1.2004
C	-0.89978	-2.52157	-1.86124
C	-3.02513	-2.16905	-0.73399
C	-1.21504	-3.88185	-2.02618
H	0.03217	-2.13914	-2.24194
C	-3.34658	-3.52558	-0.91519
H	-3.72997	-1.51145	-0.25713
C	-2.44095	-4.3823	-1.55864
H	-0.52001	-4.54223	-2.52277
H	-4.29078	-3.90891	-0.56055
N	-2.76766	-5.80699	-1.73779
C	-1.95473	-6.3575	-2.82448
H	-0.92572	-6.36621	-2.52975
H	-2.27572	-7.35625	-3.03257
H	-2.06919	-5.75538	-3.70033
C	-4.19362	-5.9316	-2.0677
H	-4.40523	-6.94451	-2.33766
H	-4.785	-5.65623	-1.21974
H	-4.4284	-5.28637	-2.88926
H	-1.15065	0.1704	-1.86054
O	0.4358	1.53022	-0.57117
C	1.73445	1.8511	-0.7991
O	2.59601	1.09713	-0.34876
H	1.83951	2.78242	-1.38089

3d-IN4

Energy: -1759.936080 Hartree

N	-1.61277	1.61408	0.66144
N	-3.37445	-0.55045	0.42872
Mn	-1.19784	-0.45029	0.48208
H	-1.67907	1.82223	1.66953
C	-0.77221	-2.14895	0.47742
O	-0.42846	-3.27908	0.46678
N	0.73694	0.38531	0.91472
C	-0.47837	2.31308	0.11814
C	-0.53058	3.54895	-0.51462
C	0.76091	1.6796	0.25153
C	0.6245	4.14007	-1.01543
H	-1.48312	4.05897	-0.6278
C	1.92504	2.26795	-0.21528
C	1.87811	3.50934	-0.88516
H	0.54293	5.09907	-1.51315
H	2.8645	1.74679	-0.07521
C	-3.74841	-1.25122	-0.74138
C	-4.00402	-3.25063	-1.75883
C	-4.25691	-1.22703	-2.94005
C	-4.23771	-2.61282	-2.97135

H	-4.0188	-4.33572	-1.68448
H	-4.47512	-0.64817	-3.83511
H	-4.42033	-3.16758	-3.88314
C	-2.9214	1.85825	0.0364
H	-2.80741	1.75246	-1.04688
H	-3.29921	2.86708	0.24535
C	-3.88863	0.83598	0.58842
H	-4.01247	0.99135	1.66346
H	-4.86877	0.93551	0.11503
N	-4.02438	-0.52305	-1.82435
N	-3.76248	-2.58524	-0.62552
N	3.03046	4.06981	-1.42313
C	4.30458	3.5676	-0.93855
H	4.43272	2.51196	-1.19241
H	5.10906	4.11951	-1.42497
H	4.42241	3.67161	0.15076
C	2.99976	5.492	-1.71912
H	2.26485	5.71597	-2.49636
H	2.76231	6.1087	-0.83924
H	3.97613	5.79467	-2.09784
C	1.82587	-0.52616	0.68408
C	2.40255	-1.19379	1.75993
C	2.24637	-0.83973	-0.60861
C	3.37449	-2.16687	1.55701
H	2.08393	-0.95511	2.77257
C	3.22347	-1.80165	-0.81787
H	1.81105	-0.3251	-1.46134
C	3.80225	-2.50748	0.25901
H	3.80243	-2.65689	2.42381
H	3.53221	-2.00716	-1.83638
N	4.73843	-3.51667	0.04188
C	5.5551	-3.91014	1.17696
H	4.94022	-4.33561	1.97402
H	6.25428	-4.68357	0.85782
H	6.13264	-3.07536	1.60333
C	5.43928	-3.50211	-1.23141
H	6.1665	-4.31452	-1.24305
H	4.75081	-3.67089	-2.06304
H	5.97292	-2.55768	-1.41867
H	0.65514	0.5385	1.92046
C	-0.98173	-0.39927	-1.26517
O	-0.81608	-0.43989	-2.43445
H	-3.60566	-1.11654	1.24083
H	-1.30538	-0.6163	2.2126
H	-1.94826	-0.20486	2.84637
O	-2.68483	0.15848	3.74983
C	-2.41148	1.37331	4.09787
O	-1.64104	2.15474	3.52557
H	-2.95152	1.69399	5.0094

3d-TS4-5

Energy: -1759.927507 Hartree

N	-1.61458	1.61394	0.6459
N	-3.36658	-0.5536	0.43349
Mn	-1.1984	-0.44294	0.48601
H	-1.68151	1.83338	1.65353
C	-0.77079	-2.14435	0.48391
O	-0.42669	-3.27301	0.4698
N	0.73146	0.38798	0.91145
C	-0.48026	2.31038	0.09924
C	-0.53148	3.54272	-0.54039
C	0.75861	1.67855	0.23992
C	0.62501	4.13121	-1.04086
H	-1.48387	4.05176	-0.65914
C	1.9242	2.26405	-0.22637
C	1.87859	3.50171	-0.90334
H	0.5447	5.08737	-1.54421
H	2.86332	1.74382	-0.08073
C	-3.73967	-1.27189	-0.72723
C	-3.99325	-3.28554	-1.71608
C	-4.24953	-1.27882	-2.92538
C	-4.22952	-2.6649	-2.93693
H	-4.00606	-4.36946	-1.62651
H	-4.46854	-0.71274	-3.82834
H	-4.41279	-3.23253	-3.84062
C	-2.92252	1.85161	0.01613
H	-2.80667	1.7376	-1.06619
H	-3.30118	2.86175	0.21663
C	-3.88864	0.83261	0.57529
H	-4.01739	0.99873	1.64797
H	-4.86679	0.92177	0.09607
N	-4.01635	-0.55908	-1.81983
N	-3.75124	-2.60381	-0.59249

N	3.03201	4.05956	-1.44113
C	4.3052	3.56052	-0.95089
H	4.43433	2.50355	-1.1987
H	5.11057	4.11007	-1.43845
H	4.42039	3.67055	0.13809
C	3.00135	5.47959	-1.74721
H	2.26877	5.6976	-2.52835
H	2.76067	6.10224	-0.87244
H	3.97874	5.78023	-2.12489
C	1.82041	-0.52603	0.68716
C	2.39346	-1.18915	1.76769
C	2.24358	-0.84565	-0.60306
C	3.36519	-2.16371	1.57169
H	2.07222	-0.94567	2.77838
C	3.22023	-1.80946	-0.80544
H	1.81128	-0.33425	-1.45925
C	3.79585	-2.51062	0.27622
H	3.79067	-2.65012	2.4417
H	3.53141	-2.01997	-1.82219
N	4.73186	-3.52105	0.06627
C	5.5457	-3.90987	1.20499
H	4.92871	-4.33132	2.00256
H	6.24515	-4.68513	0.89101
H	6.12268	-3.07345	1.62881
C	5.43405	-3.51521	-1.20634
H	6.16106	-4.32786	-1.21177
H	4.74637	-3.68935	-2.03753
H	5.96809	-2.57219	-1.39929
H	0.65304	0.54953	1.91648
C	-0.98208	-0.40578	-1.25623
O	-0.81469	-0.45049	-2.42438
H	-3.59762	-1.10981	1.25279
H	-1.27011	-0.64728	2.26145
H	-1.90977	-0.18263	2.81686
O	-2.71408	0.26163	3.73833
C	-2.41139	1.46921	4.06091
O	-1.61829	2.22442	3.47504
H	-2.93966	1.83239	4.96574

3d-IN5

Energy: -1759.927679 Hartree

N	-1.61277	1.61408	0.66144
N	-3.37445	-0.55045	0.42872
Mn	-1.19784	-0.45029	0.48208
H	-1.67907	1.82223	1.66953
C	-0.77221	-2.14895	0.47742
O	-0.42846	-3.27908	0.46678
N	0.73694	0.38531	0.91472
C	-0.47837	2.31308	0.11814
C	-0.53058	3.54895	-0.51462
C	0.76091	1.6796	0.25153
C	0.6245	4.14007	-1.01543
H	-1.48312	4.05897	-0.6278
C	1.92504	2.26795	-0.21528
C	1.87811	3.50934	-0.88516
H	0.54293	5.09907	-1.51315
H	2.8645	1.74679	-0.07521
C	-3.74841	-1.25122	-0.74138
C	-4.00402	-3.25063	-1.75883
C	-4.25691	-1.22703	-2.94005
C	-4.23771	-2.61282	-2.97135
H	-4.0188	-4.33572	-1.68448
H	-4.47512	-0.64817	-3.83511
H	-4.42033	-3.16758	-3.88314
C	-2.9214	1.85825	0.0364
H	-2.80741	1.75246	-1.04688
H	-3.29921	2.86708	0.24535
C	-3.88863	0.83598	0.58842
H	-4.01247	0.99135	1.66346
H	-4.86877	0.93551	0.11503
N	-4.02438	-0.52305	-1.82435
N	-3.76248	-2.58524	-0.62552
N	3.03046	4.06981	-1.42313
C	4.30458	3.5676	-0.93855
H	4.43272	2.51196	-1.19241
H	5.10906	4.11951	-1.42497
H	4.42241	3.67161	0.15076
C	2.99976	5.492	-1.71912
H	2.26485	5.71597	-2.49636
H	2.76231	6.1087	-0.83924
H	3.97613	5.79467	-2.09784
C	1.82587	-0.52616	0.68408
C	2.40255	-1.19379	1.75993
C	2.24637	-0.83973	-0.60861

C	3.37449	-2.16687	1.55701
H	2.08393	-0.95511	2.77257
C	3.22347	-1.80165	-0.81787
H	1.81105	-0.3251	-1.46134
C	3.80225	-2.50748	0.25901
H	3.80243	-2.65689	2.42381
H	3.53221	-2.00716	-1.83638
N	4.73843	-3.51667	0.04188
C	5.5551	-3.91014	1.17696
H	4.94022	-4.33561	1.97402
H	6.25428	-4.68357	0.85782
H	6.13264	-3.07536	1.60333
C	5.43928	-3.50211	-1.23141
H	6.1665	-4.31452	-1.24305
H	4.75081	-3.67089	-2.06304
H	5.97292	-2.55768	-1.41867
H	0.65514	0.5385	1.92046
C	-0.98173	-0.39927	-1.26517
O	-0.81608	-0.43989	-2.43445
H	-3.60566	-1.11654	1.24083
H	-1.30538	-0.6163	2.2126
H	-1.94826	-0.20486	2.84637
O	-2.68483	0.15848	3.74983
C	-2.41148	1.37331	4.09787
O	-1.64104	2.15474	3.52557
H	-2.95152	1.69399	5.0094

3e-IN1

Energy: -1468.412009 Hartree

N	1.62975	1.64197	-1.41654
N	3.48778	-0.46129	-1.10481
Mn	1.30983	-0.41824	-0.96188
H	1.58393	1.74525	-2.42912
C	0.95171	-2.11285	-0.79323
O	0.6553	-3.25664	-0.68843
N	-0.69423	0.33701	-1.24316
C	0.54368	2.36417	-0.80579
C	0.6305	3.65835	-0.3084
C	-0.67653	1.68629	-0.70803
C	-0.46906	4.2675	0.28702
H	1.56852	4.2023	-0.37696
C	-1.78911	2.29083	-0.14456
C	-1.70259	3.59387	0.39102
H	-0.35894	5.27384	0.67322
H	-2.71819	1.73503	-0.10521
C	4.01164	-0.97183	0.10268
C	4.44678	-2.79399	1.3655
C	4.77043	-0.61421	2.19924
C	4.79805	-1.97985	2.43586
H	4.48841	-3.87782	1.44959
H	5.07348	0.09434	2.9674
H	5.10235	-2.39008	3.39065
C	2.99528	1.96551	-0.97696
H	3.00141	1.97264	0.11701
H	3.32643	2.94978	-1.33085
C	3.91778	0.89668	-1.51846
H	3.88562	0.90592	-2.61159
H	4.9489	1.08459	-1.20657
N	4.38921	-0.08748	1.02832
N	4.05584	-2.30886	0.18384
N	-2.79448	4.17574	1.02418
C	-4.10254	3.60069	0.7612
H	-4.16105	2.57198	1.1265
H	-4.85624	4.17881	1.29598
H	-4.36381	3.59685	-0.30788
C	-2.77738	5.61989	1.18348
H	-1.95415	5.93472	1.82963
H	-2.68299	6.15655	0.22744
H	-3.70502	5.93258	1.66332
C	-1.71878	-0.56892	-0.80233
C	-3.26228	-2.25861	-1.35363
C	-2.85289	-1.66996	0.93879
C	-3.49213	-2.43822	-0.01253
H	-3.77212	-2.83659	-2.11577
H	-3.03905	-1.78267	2.00078
H	-0.72543	0.38904	-2.26018
C	1.28193	-0.19346	0.79962
O	1.25983	-0.1463	1.98391
H	3.64193	-1.13588	-1.84694
H	1.33713	-0.67269	-2.62517
N	-2.37551	-1.32047	-1.70662
N	-1.9716	-0.76024	0.50662
H	-4.18144	-3.19288	0.30409

3e-IN2

Energy: -1656.987131 Hartree

H	1.27883	1.09225	1.93664
N	0.05331	-1.19859	1.62965
N	2.7936	-0.83831	1.07033
Mn	1.03268	0.2864	0.46112
C	0.86264	-0.55178	-1.09462
O	0.81738	-1.05634	-2.16211
H	-0.03382	-0.81735	2.57222
C	0.69784	2.53551	2.85009
O	-0.32191	2.12253	3.30304
O	1.57221	3.30959	2.64727
C	1.79252	1.66996	-0.28666
O	2.26905	2.64314	-0.76105
N	-1.05164	0.94825	0.51462
H	3.34808	-0.13193	1.54413
C	-1.25757	-1.38518	1.06228
C	-1.96782	-2.57932	1.0751
C	-1.84018	-0.26624	0.46099
C	-3.22785	-2.65942	0.49389
H	-1.5365	-3.46143	1.53967
C	-3.11058	-0.31724	-0.08854
C	-3.82614	-1.53362	-0.10873
H	-3.74884	-3.60912	0.51572
H	-3.53164	0.58437	-0.51601
C	3.49255	-1.27494	-0.07952
C	4.90535	-0.74193	-1.75888
C	3.84381	-2.84368	-1.6641
C	4.68211	-1.97946	-2.35058
H	5.58309	-0.01986	-2.20875
H	3.64483	-3.84672	-2.03554
H	5.15525	-2.26048	-3.28312
C	0.94353	-2.36962	1.68084
H	0.92691	-2.84512	0.69605
H	0.61853	-3.10575	2.42575
C	2.33046	-1.87675	2.02464
H	2.31724	-1.39971	3.00855
H	3.03786	-2.70971	2.05871
N	3.24409	-2.51143	-0.513
N	4.32174	-0.37016	-0.61583
N	-5.06759	-1.61878	-0.72522
C	-5.7768	-0.37582	-0.97544
H	-5.22196	0.26015	-1.6698
H	-6.73596	-0.60459	-1.44022
H	-5.96504	0.20392	-0.05919
C	-5.90976	-2.74862	-0.37046
H	-5.45117	-3.69252	-0.67581
H	-6.11944	-2.80551	0.70817
H	-6.85804	-2.66078	-0.90065
H	-1.11814	1.37556	1.43953
C	-1.20043	1.96786	-0.45647
N	-1.07935	3.20768	0.03308
N	-1.37676	1.60097	-1.72673
C	-1.0678	4.18216	-0.88401
C	-1.3773	2.60611	-2.61022
C	-1.20362	3.93372	-2.2426
H	-0.95743	5.19594	-0.50595
H	-1.52249	2.32733	-3.65166
H	-1.19261	4.73212	-2.97398

3e-TS2-3

Energy: -1656.980099 Hartree

H	1.30653	1.06669	2.10549
N	1.67498	-1.40569	1.23469
N	3.41113	0.71245	0.55735
Mn	1.23442	0.55225	0.48311
C	1.13841	0.07673	-1.22678
O	1.04825	-0.16522	-2.37926
H	1.63629	-1.36035	2.25259
C	1.03997	0.73265	3.89248
O	0.35497	-0.2408	3.84279
O	1.65858	1.61754	4.37195
C	0.79912	2.20634	0.10901
O	0.46764	3.31673	-0.1138
N	-0.70669	-0.25265	0.98469
H	3.54902	1.51557	1.16169
C	0.616	-2.25721	0.7536
C	0.76759	-3.58557	0.37892
C	-0.64034	-1.65432	0.61396
C	-0.30425	-4.30708	-0.13678
H	1.73954	-4.06393	0.46219
C	-1.72511	-2.36627	0.1324
C	-1.57914	-3.71681	-0.25823
H	-0.1421	-5.33137	-0.45059

H	-2.67947	-1.8628	0.03897
C	3.88861	1.02671	-0.73615
C	4.2294	2.61584	-2.3012
C	4.6451	0.33635	-2.7463
C	4.61748	1.64182	-3.21669
H	4.21705	3.66956	-2.57359
H	4.96997	-0.48478	-3.38259
H	4.90295	1.89174	-4.23078
C	3.05073	-1.7212	0.82469
H	3.05193	-1.8847	-0.25687
H	3.43952	-2.62362	1.31365
C	3.92139	-0.53356	1.17847
H	3.90533	-0.36901	2.2603
H	4.95641	-0.71685	0.87598
N	4.28781	0.00855	-1.49932
N	3.86561	2.32736	-1.05004
N	-2.65975	-4.42537	-0.73838
C	-3.87111	-3.71212	-1.08219
H	-3.7011	-2.92235	-1.8299
H	-4.59777	-4.41584	-1.48689
H	-4.32033	-3.24289	-0.19969
C	-2.4419	-5.73462	-1.31327
H	-1.77288	-5.71166	-2.18674
H	-2.00678	-6.41947	-0.57753
H	-3.39895	-6.15149	-1.62585
H	-0.63995	-0.18785	2.0012
C	-1.85867	0.543	0.5366
N	-2.80689	0.82192	1.47238
N	-1.86466	0.92314	-0.77037
C	-3.85755	1.55305	1.04492
C	-2.92888	1.65285	-1.16547
C	-3.97115	2.00213	-0.28508
H	-4.63193	1.78406	1.79804
H	-2.94179	1.96566	-2.22482
H	-4.82937	2.5949	-0.61886

3e-IN3

Energy: -1657.000275 Hartree

H	1.27883	1.09225	1.93664
N	0.05331	-1.19859	1.62965
N	2.7936	-0.83831	1.07033
Mn	1.03268	0.2864	0.46112
C	0.86264	-0.55178	-1.09462
O	0.81738	-1.05634	-2.16211
H	-0.03382	-0.81735	2.57222
C	0.69784	2.53551	2.85009
O	-0.32191	2.12253	3.30304
O	1.57221	3.30959	2.64727
C	1.79252	1.66996	-0.28666
O	2.26905	2.64314	-0.76105
N	-1.05164	0.94825	0.51462
H	3.34808	-0.13193	1.54413
C	-1.25757	-1.38518	1.06228
C	-1.96782	-2.57932	1.0751
C	-1.84018	-0.26624	0.46099
C	-3.22785	-2.65942	0.49389
H	-1.5365	-3.46143	1.53967
C	-3.11058	-0.31724	-0.08854
C	-3.82614	-1.53362	-0.10873
H	-3.74884	-3.60912	0.51572
H	-3.53164	0.58437	-0.51601
C	3.49255	-1.27494	-0.07952
C	4.90535	-0.74193	-1.75888
C	3.84381	-2.84368	-1.6641
C	4.68211	-1.97946	-2.35058
H	5.58309	-0.01986	-2.20875
H	3.64483	-3.84672	-2.03554
H	5.15525	-2.26048	-3.28312
C	0.94353	-2.36962	1.68084
H	0.92691	-2.84512	0.69605
H	0.61853	-3.10575	2.42575
C	2.33046	-1.87675	2.02464
H	2.31724	-1.39971	3.00855
H	3.03786	-2.70971	2.05871
N	3.24409	-2.51143	-0.513
N	4.32174	-0.37016	-0.61583
N	-5.06759	-1.61878	-0.72522
C	-5.7768	-0.37582	-0.97544
H	-5.22196	0.26015	-1.6698
H	-6.73596	-0.60459	-1.44022
H	-5.96504	0.20392	-0.05919
C	-5.90976	-2.74862	-0.37046
H	-5.45117	-3.69252	-0.67581
H	-6.11944	-2.80551	0.70817

H	-6.85804	-2.66078	-0.90065
H	-1.11814	1.37556	1.43953
C	-1.20043	1.96786	-0.45647
N	-1.07935	3.20768	0.03308
N	-1.37676	1.60097	-1.72673
C	-1.0678	4.18216	-0.88401
C	-1.3773	2.60611	-2.61022
C	-1.20362	3.93372	-2.2426
H	-0.95743	5.19594	-0.50595
H	-1.52249	2.32733	-3.65166
H	-1.19261	4.73212	-2.97398

3e-IN3R

Energy: -1657.028551 Hartree

N	1.23404	1.9359	-1.07485
N	3.33265	0.1523	-0.57229
Mn	1.20022	-0.10825	-0.57073
C	1.0089	0.16608	1.14357
O	0.87751	0.3298	2.30744
H	1.26508	1.93668	-2.09623
C	1.11294	-1.83971	-0.29096
O	1.00342	-2.99749	-0.09188
N	-0.85445	0.28611	-1.02577
H	3.55242	-0.50649	-1.32728
C	-0.00411	2.50972	-0.61705
C	-0.16789	3.82466	-0.20143
C	-1.10582	1.64805	-0.58511
C	-1.40273	4.2763	0.25145
H	0.67757	4.5068	-0.22176
C	-2.34885	2.0907	-0.16346
C	-2.51943	3.41661	0.29076
H	-1.49106	5.3051	0.57992
H	-3.17878	1.39436	-0.16952
C	3.94921	-0.30778	0.62329
C	4.77007	-2.11285	1.76097
C	4.73754	0.03771	2.77439
C	5.04156	-1.31387	2.87073
H	4.98832	-3.17867	1.74593
H	4.94926	0.67742	3.6259
H	5.47816	-1.77538	3.74938
C	2.49553	2.49624	-0.56799
H	2.40921	2.57234	0.52128
H	2.69538	3.50042	-0.9606
C	3.6028	1.55183	-0.99031
H	3.66665	1.54023	-2.08306
H	4.57085	1.89225	-0.60915
N	4.23221	-1.61494	0.63951
N	-3.7436	3.84387	0.78753
C	-4.91185	3.0393	0.47509
H	-4.82746	2.03931	0.90891
H	-5.7917	3.51062	0.91308
H	-5.08086	2.92682	-0.60651
C	-3.97436	5.27721	0.84768
H	-3.28291	5.75846	1.54403
H	-3.86873	5.76973	-0.13047
H	-4.98467	5.4566	1.21571
H	-0.79324	0.27179	-2.04576
O	1.29615	-0.28358	-2.65199
C	2.12823	-1.016	-3.2898
O	3.08745	-1.65882	-2.8245
H	1.94396	-1.05151	-4.38484
C	-1.77234	-0.77872	-0.59623
N	-2.56352	-1.32566	-1.5593
N	-1.74147	-1.11381	0.72267
C	-3.40016	-2.30162	-1.14858
C	-2.58993	-2.09286	1.10046
C	-3.45554	-2.73146	0.19144
H	-4.04598	-2.75111	-1.92388
H	-2.57141	-2.37114	2.16932
H	-4.13937	-3.52477	0.5114
N	4.19875	0.51296	1.6505

3e-IN4

Energy: -1658.161754 Hartree

N	0.10661	-1.69995	0.57591
N	2.80554	-0.99536	0.36658
Mn	1.00505	0.20962	0.49968
H	0.04412	-1.96203	1.57304
C	1.69222	1.82338	0.56802
O	2.10041	2.93007	0.60317
N	-1.07047	0.65554	0.96302
C	-1.22428	-1.56519	0.04505
C	-1.92239	-2.56396	-0.62385
C	-1.85088	-0.33237	0.2406

C	-3.21059	-2.33569	-1.0933
H	-1.45842	-3.53273	-0.78581
C	-3.14709	-0.09772	-0.18746
C	-3.85102	-1.09526	-0.89463
H	-3.71948	-3.13542	-1.61803
H	-3.59744	0.86723	0.01094
C	3.51989	-0.59328	-0.7885
C	4.90715	0.91667	-1.73239
C	3.89871	-0.79309	-3.00343
C	4.70665	0.33309	-2.97763
H	5.56479	1.77502	-1.61491
H	3.72306	-1.33747	-3.92886
H	5.176	0.72171	-3.87265
C	1.00973	-2.64186	-0.10438
H	0.97447	-2.43477	-1.17838
H	0.71367	-3.68623	0.05457
C	2.39802	-2.42443	0.45161
H	2.41334	-2.68048	1.51403
H	3.12524	-3.05785	-0.06248
N	3.30092	-1.28215	-1.90862
N	4.32505	0.4622	-0.61861
N	-5.12171	-0.84833	-1.39918
C	-5.85113	0.27961	-0.8455
H	-5.33864	1.22137	-1.05828
H	-6.83277	0.33076	-1.31675
H	-5.99266	0.20778	0.2435
C	-5.93643	-2.00333	-1.73759
H	-5.48749	-2.57644	-2.55265
H	-6.08915	-2.6846	-0.88716
H	-6.91179	-1.65871	-2.08121
H	-1.10232	0.47416	1.96658
C	0.79339	0.37887	-1.24503
O	0.68326	0.55594	-2.40543
H	3.33646	-0.72393	1.19036
H	1.17868	0.18397	2.22925
H	1.49409	-0.54826	2.79784
O	1.91699	-1.32733	3.6559
C	1.0094	-2.19277	3.96564
O	-0.07722	-2.36732	3.39787
H	1.28404	-2.81727	4.83762
C	-1.28934	2.04165	0.75712
N	-1.2148	2.78232	1.86722
N	-1.47707	2.45638	-0.49659
C	-1.28141	4.10552	1.67242
C	-1.55433	3.7834	-0.64717
C	-1.44486	4.66999	0.41607
H	-1.21184	4.72554	2.5629
H	-1.70916	4.14156	-1.66255
H	-1.49754	5.74191	0.27289

3e-TS[4-5]

Energy: -1658.152613 Hartree

N	-1.61277	1.61408	0.66144
N	-3.37445	-0.55045	0.42872
Mn	-1.19784	-0.45029	0.48208
H	-1.67907	1.82223	1.66953
C	-0.77221	-2.14895	0.47742
O	-0.42846	-3.27908	0.46678
N	0.73694	0.38531	0.91472
C	-0.47837	2.31308	0.11814
C	-0.53058	3.54895	-0.51462
C	0.76091	1.6796	0.25153
C	0.6245	4.14007	-1.01543
H	-1.48312	4.05897	-0.6278
C	1.92504	2.26795	-0.21528
C	1.87811	3.50934	-0.88516
H	0.54293	5.09907	-1.51315
H	2.8645	1.74679	-0.07521
C	-3.74841	-1.25122	-0.74138
C	-4.00402	-3.25063	-1.75883
C	-4.25691	-1.22703	-2.94005
C	-4.23771	-2.61282	-2.97135
H	-4.0188	-4.33572	-1.68448
H	-4.47512	-0.64817	-3.83511
H	-4.42033	-3.16758	-3.88314
C	-2.9214	1.85825	0.0364
H	-2.80741	1.75246	-1.04688
H	-3.29921	2.86708	0.24535
C	-3.88863	0.83598	0.58842
H	-4.01247	0.99135	1.66346
H	-4.86877	0.93551	0.11503

N	-4.02438	-0.52305	-1.82435
N	-3.76248	-2.58524	-0.62552
N	3.03046	4.06981	-1.42313
C	4.30458	3.5676	-0.93855
H	4.43272	2.51196	-1.19241
H	5.10906	4.11951	-1.42497
H	4.42241	3.67161	0.15076
C	2.99976	5.492	-1.71912
H	2.26485	5.71597	-2.49636
H	2.76231	6.1087	-0.83924
H	3.97613	5.79467	-2.09784
H	0.65514	0.5385	1.92046
C	-0.98173	-0.39927	-1.26517
O	-0.81608	-0.43989	-2.43445
H	-3.60566	-1.11654	1.24083
H	-1.30538	-0.6163	2.2126
H	-1.94826	-0.20486	2.84637
O	-2.68483	0.15848	3.74983
C	-2.41148	1.37331	4.09787
O	-1.64104	2.15474	3.52557
H	-2.95152	1.69399	5.0094
C	1.84959	-0.54602	0.67905
N	2.67859	-0.78304	1.73223
N	1.94222	-1.08044	-0.56936
C	3.69463	-1.64013	1.49933
C	2.96884	-1.93328	-0.76904
C	3.88994	-2.25395	0.24692
H	4.37238	-1.83823	2.34882
H	3.05148	-2.37172	-1.77957
H	4.71885	-2.9478	0.07139

3e-IN5

Energy: -1658.15 Hartree

N	0.10661	-1.69995	0.57591
N	2.80554	-0.99536	0.36658
Mn	1.00505	0.20962	0.49968
H	0.04412	-1.96203	1.57304
C	1.69222	1.82338	0.56802
O	2.10041	2.93007	0.60317
N	-1.07047	0.65554	0.96302
C	-1.22428	-1.56519	0.04505
C	-1.92239	-2.56396	-0.62385
C	-1.85088	-0.33237	0.2406
C	-3.21059	-2.33569	-1.0933
H	-1.45842	-3.53273	-0.78581
C	-3.14709	-0.09772	-0.18746
C	-3.85102	-1.09526	-0.89463
H	-3.71948	-3.13542	-1.61803
H	-3.59744	0.86723	0.01094
C	3.51989	-0.59328	-0.7885
C	4.90715	0.91667	-1.73239
C	3.89871	-0.79309	-3.00343
C	4.70665	0.33309	-2.97763
H	5.56479	1.77502	-1.61491
H	3.72306	-1.33747	-3.92886
H	5.176	0.72171	-3.87265
C	1.00973	-2.64186	-0.10438
H	0.97447	-2.43477	-1.17838
H	0.71367	-3.68623	0.05457
C	2.39802	-2.42443	0.45161
H	2.41334	-2.68048	1.51403
H	3.12524	-3.05785	-0.06248
N	3.30092	-1.28215	-1.90862
N	4.32505	0.4622	-0.61861
N	-5.12171	-0.84833	-1.39918
C	-5.85113	0.27961	-0.8455
H	-5.33864	1.22137	-1.05828
H	-6.83277	0.33076	-1.31675
H	-5.99266	0.20778	0.2435
C	-5.93643	-2.00333	-1.73759
H	-5.48749	-2.57644	-2.55265
H	-6.08915	-2.6846	-0.88716
H	-6.91179	-1.65871	-2.08121
H	-1.10232	0.47416	1.96658
C	0.79339	0.37887	-1.24503

O	0.68326	0.55594	-2.40543	N	-1.2148	2.78232	1.86722
H	3.33646	-0.72393	1.19036	N	-1.47707	2.45638	-0.49659
H	1.17868	0.18397	2.22925	C	-1.28141	4.10552	1.67242
H	1.49409	-0.54826	2.79784	C	-1.55433	3.7834	-0.64717
O	1.91699	-1.32733	3.6559	C	-1.44486	4.66999	0.41607
C	1.0094	-2.19277	3.96564	H	-1.21184	4.72554	2.5629
O	-0.07722	-2.36732	3.39787	H	-1.70916	4.14156	-1.66255
H	1.28404	-2.81727	4.83762	H	-1.49754	5.74191	0.27289
C	-1.28934	2.04165	0.75712				

Table of Electronic Energies of the transition states and intermediates in Hartree-

GROUP-1					
CO2 to Formate formation					
	1a	1b	1c	1d	1e
IN1	-656.000078	-1385.675418	-1267.84614	-1283.90552	-1182.13442
IN2	-844.574929	-1574.251535	-1456.42122	-1472.48019	-1370.70982
TS[2-3]	-844.567253	-1574.244115	-1456.41633	-1472.47551	-1370.70496
Imaginary Frequency	-483.34	-439	-426.72	-467.48	-506.48
IN3	-844.589418	-1574.267304	-1456.4364	-1472.49372	-1370.72091
IN3A	-844.612632	-1574.290234	-1456.45959	-1472.51776	-1370.74576

Formate to Formic Acid formation					
IN4	-845.747504	-1575.422955	-1457.59749	-1473.64871	-1371.8767
TS[4-5]	-845.738665	-1575.414517	-1457.58068	-1473.64243	-1371.86951
Imaginary Frequency	-971.5	-1013.38	-967.46	-999.06	-956.92
IN5	-845.737855	-1575.413492	-1457.58317	-1473.64314	-1371.87307

GROUP-2					
CO2 to Formate formation					
	2a	2b	2c	2d	2e
IN1	-808.3919	-1538.07	-1420.237	-1436.29654	-1334.522
IN2	-996.965204	-1726.64	-1608.812	-1624.87075	-1523.095
TS[2-3]	-996.960621	-1726.64	-1608.807	-1624.86584	-1523.092
Imaginary Frequency	-397.71	-469.48	-438.63	-415.61	-451.41
IN3	-996.982986	-1726.66	-1608.827	-1624.8854	-1523.11
IN3A	-997.007483	-1726.68	-1608.852	-1624.91394	-1523.134
Formate to Formic Acid formation					
IN4	-998.147463	-1727.82	-1727.819	-1626.04525	-1524.269
TS[4-5]	-998.130637	-1727.8	-1727.805	-1626.0332	-1524.259
Imaginary Frequency	-971.85	-983.98	-1067.73	-971.92	-988.93
IN5	-998.127869	-1727.8	-1727.806	-1626.03313	-1524.258

GROUP-3					
CO2 to Formate formation					
	3a	3b	3c	3d	3e
IN1	-942.278868	-1671.956225	-1554.12766	-1570.18614	-1468.412
IN2	-1130.855289	-1860.532152	-1742.71226	-1758.76149	-1656.987
TS[2-3]	-1130.850815	-1860.526284	-1742.69685	-1758.75547	-1656.98
Imaginary Frequency	-390.31	-386.55	-417.92	-371.45	-451.41
IN3	-1130.872616	-1860.548756	-1742.71767	-1758.77675	-1657
IN3A	-1130.893853	-1860.574962	-1742.74489	-1758.80335	-1657.029
Formate to Formic Acid formation					
IN4	-1132.030425	-1861.712099	-1743.88344	-1759.93608	-1658.162
TS[4-5]	-1132.020639	-1861.695506	-1743.86559	-1759.92751	-1658.153

**Imaginary Frequency**	-969.92	-994.91	-971.96	-1056.85	-988.93
**IN5**	-1132.019313	-1861.698507	-1743.86614	-1759.92768	-1658.153
